# Efficient biological carbon export to the mesopelagic ocean induced by submesoscale fronts

**DOI:** 10.1038/s41467-024-44846-7

**Published:** 2024-01-17

**Authors:** Mingxian Guo, Xiaogang Xing, Peng Xiu, Giorgio Dall’Olmo, Weifang Chen, Fei Chai

**Affiliations:** 1 State Key Laboratory of Tropical Oceanography, South China Sea Institute of Oceanology, Chinese Academy of Sciences, Guangzhou, China; 2 State Key Laboratory of Satellite Ocean Environment Dynamics, Second Institute of Oceanography, Ministry of Natural Resources, Hangzhou, China; 3 State Key Laboratory of Marine Environmental Science & College of Ocean and Earth Sciences, Xiamen University, Xiamen, China; 4 Istituto Nazionale di Oceanografia e di Geofisica Sperimentale—OGS, Trieste, Italy

**Keywords:** Marine biology, Marine chemistry

## Abstract

Oceanic submesoscale processes are ubiquitous in the North Pacific Subtropical Gyre (NPSG), where the biological carbon pump is generally ineffective. Due to difficulties in collecting continuous observations, however, it remains uncertain whether episodic submesoscale processes can drive significant changes in particulate organic carbon (POC) export into the mesopelagic ocean. Here we present observations from high-frequency Biogeochemical-Argo floats in the NPSG, which captured the enhanced POC export fluxes during the intensifying stages of a submesoscale front and a cyclonic eddy compared to their other life stages. A higher percentage of POC export flux was found to be transferred to the base of mesopelagic layer at the front compared to that at the intensifying eddy and the mean of previous studies (37% vs. ~10%), suggesting that the POC export efficiency was significantly strengthened by submesoscale dynamics. Such findings highlight the importance of submesoscale fronts for carbon export and sequestration in subtropical gyres.

## Abstract (web summary)

Submesoscale processes are ubiquitous in the North Pacific Subtropical Gyre. The authors show that carbon export efficiency is significantly strengthened under the influence of these episodic features by using high-frequency BGC-Argo observations.

## Introduction

Biological pumping of particulate organic carbon (POC), one of the primary mechanisms that draws atmospheric CO_2_ to the deep sea, has been reported to be ineffective within oligotrophic gyres, such as the North Pacific Subtropical Gyre (NPSG)^1–3^. In these regions, weak vertical nutrient supply limits biological productivity^4^ and consequently reduces carbon export^5,6^. Furthermore, the downward POC flux attenuates rapidly in the mesopelagic layer due to the dominance of small-size sinking particles^7^, considerable fragmentation^8^, and relatively fast remineralization compared to that at high latitudes^9–11^.

Subtropical gyres have abundant mesoscale eddies characterized by small Rossby numbers (R_0_) and large Richardson numbers (R_i_), as well as submesoscale fronts characterized by O(1) R_0_ and R_i_^12–16^. Mesoscale eddies and submesoscale fronts can impact biological pumps in various ways^16–24^. Generally, mesoscale eddies experience different life stages, such as the intensifying, mature, and decaying stages. The stage during eddy formation and intensification is typically associated with upward isopycnal displacement in cyclonic eddies (CEs) and downward displacement in anticyclonic eddies (ACEs). The upward isopycnal displacement during a CE intensifying stage can increase nutrient supply and primary production^25–28^, enhancing POC sinking in subtropical gyres (referred to as the biological gravitational pump)^29–31^. Due to the increase in the lateral buoyancy gradient^32^, intensifying submesoscale fronts are often accompanied by vertical velocities as high as 10–100 m d^−1^, which is an order of magnitude greater than the vertical velocities induced by mesoscale eddies^14,15^. These vertical motions can inject high quantities of POC produced in the euphotic zone into the mesopelagic layer (referred to as subduction pump)^17,19,22,24^, resulting in carbon sequestration^17,19,20,23^. The vertical motions induced by submesoscale fronts may also drive the vertical nutrient supply and stimulate the growth of large phytoplankton communities^32–36^; these large phytoplankton may sink fast and affect the carbon export efficiency by creating particles with fast sinking velocities through food web dynamics^16,36,37^. At the global scale, submesoscale fronts have the potential to contribute to POC export^38,39^, which would help to reconcile the imbalance between the upper layer carbon supply and deep ocean carbon consumption^9,17,40^.

Prior studies have captured anomalous features of elevated POC at depth, which are interpreted as evidence of the subduction pump^19,22,23^. Despite their potential importance in shaping POC export, direct observations of the downward POC flux and its attenuation in response to submesoscale fronts are scarce^17,19–21,23,41^. This is largely attributable to their relatively small spatiotemporal scales and the difficulty in obtaining subsurface observations, as well as the possible decoupling between physical and biogeochemical processes at the front^41^. Mesoscale features have been suggested to be able to change the strength of oceanic carbon export locally^29–31,42,43^, whereas the effects of submesoscale features are still unclear. Using high-resolution simulations, Omand et al.^19^ showed that the subduction pump contributes up to 50% of the spring POC flux in the North Atlantic and Southern Ocean. However, Llort et al.^23^ showed that the contribution ranged between 1% in spring and 14–19% in summer in the Southern Ocean. By using a model covering a subpolar and a subtropical gyre, Resplandy et al.^18^ suggested that the subduction pump contributes less than 5% to the annual carbon export due to the strong compensation between upward and downward fluxes at the basin scale. Given the large uncertainty among the estimations from different studies, more high-resolution field observations are required. There are challenges when estimating the POC export flux associated with episodic submesoscale features by either sediment traps or radioactive pairs (mainly the ^234^Th-^238^U pair). Biogeochemical-Argo (BGC-Argo) floats can fill observational gaps over small spatial and temporal scales^20,23,28,44,45^. By tracking the particle accumulation rates in the water column over sustained periods, BGC-Argo data have been used to estimate the downward POC flux^8,45–48^.

BGC-Argo data include variables that may change in both space and time, especially when sampled near mesoscale and submesoscale features. Thus, careful analysis is required to reasonably delineate the temporal rates and/or the spatial variability of measured variables. The BGC-Argo has been used to identify elevated POC in the mesopelagic layer and estimate the carbon flux associated with submesoscale features^20,23^. However, the transfer efficiency of POC export associated with submesoscale features has rarely been investigated.

In this study, we show the capability of using BGC-Argo floats to estimate the efficiency of POC export in response to submesoscale fronts and eddies. We deployed two high-frequency sampling floats that profiled twice a day in the western NPSG. These two floats captured the intensifying stages of a mesoscale eddy and a submesoscale front, which were used to estimate vertically resolved POC export fluxes and transfer efficiencies. Further data synthesis extrapolating the results to the entire subtropical gyre revealed the important role that submesoscale fronts may play in strengthening the biological POC pump in the mesopelagic ocean.

## Results

### Site characterization and tracer distributions

**Fig. 1:**
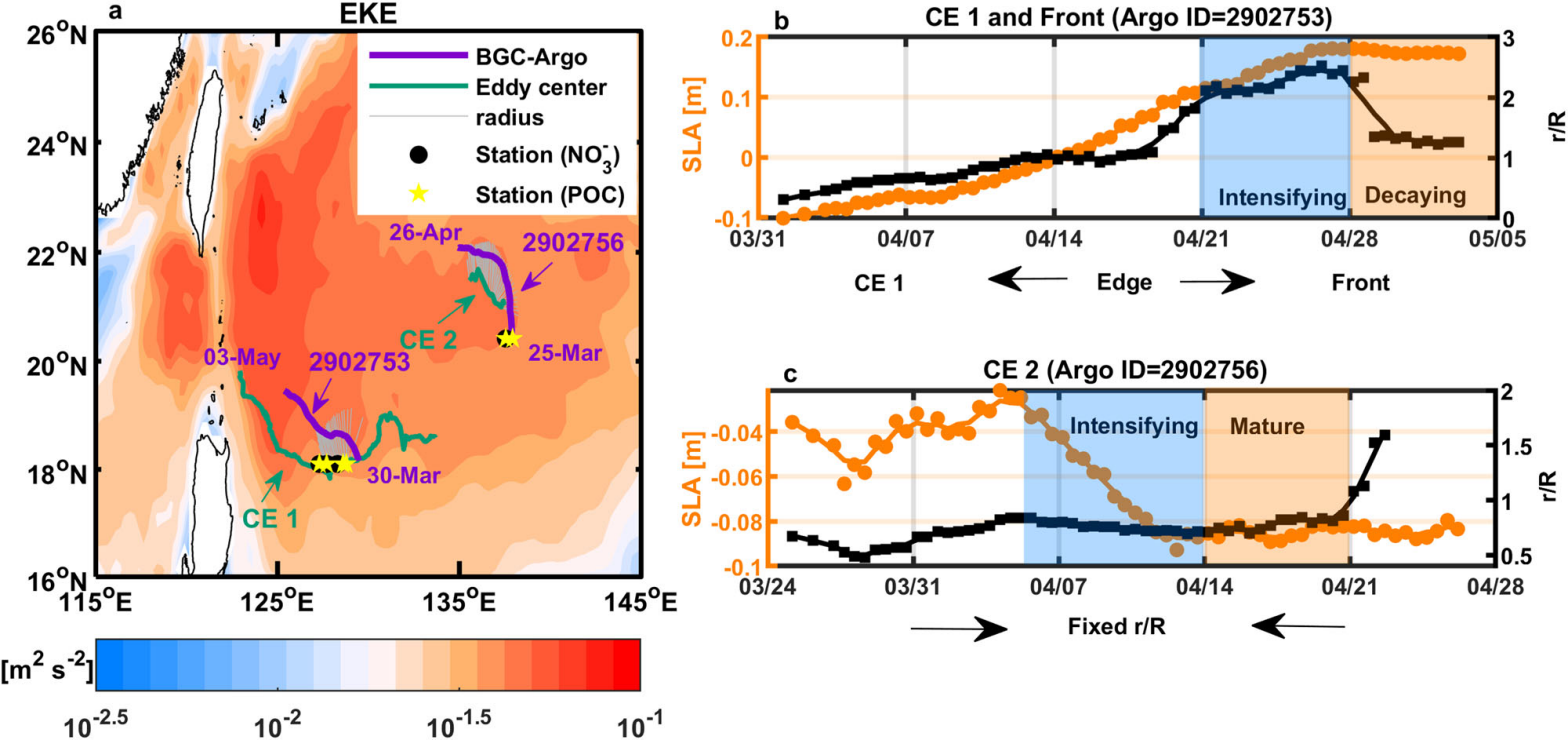
Locations of the Biogeochemical-Argo (BGC-Argo) floats and their positions relative to the eddies. **a** The trajectories of BGC-Argo floats (WMO numbers 2902753 and 2902756) in purple and the trajectories of the center of mesoscale eddies (identified as cyclonic eddy 1 (CE 1) and cyclonic eddy 2 (CE 2)) in green. The gray lines indicate the eddy radius. The background color is the mean eddy kinetic energy (EKE (m^2^ s^-2^)) from 1993 to 2020. Black dots and yellow pentagrams represent in-situ sampling locations of nitrate (NO_3_^-^) and particulate organic carbon (POC), respectively. **b** Along-track sea level anomaly (SLA; m) in orange and the relative distance from the center of CE 1 normalized by eddy radius, r/R, in black for float 2902753. The blue and orange areas correspond to the intensifying and decaying stages of the submesoscale front, respectively. **c** The same as **b** but for float 2902756 in CE 2. In **c**, between the two vertical dashed lines, the float was at a relatively fixed r/R ~ 0.7. The blue and orange areas correspond to the intensifying and mature stages of CE 2. Source data are provided as a Source Data file.

**Fig. 2:**
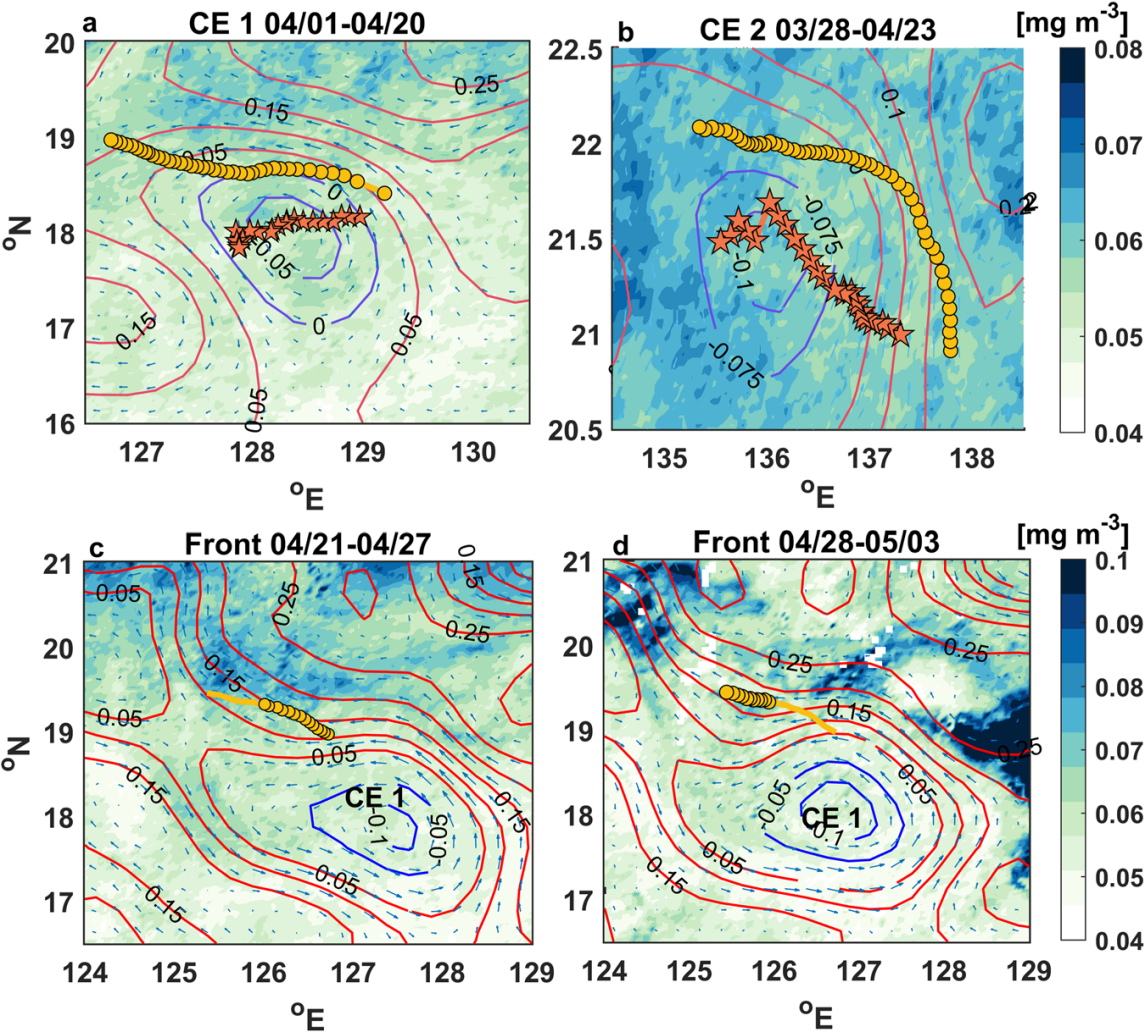
The Biogeochemical-Argo (BGC-Argo) and eddy trajectories. **a** The locations of (yellow dots) the BGC-Argo float (WMO number 2902753) and (orange pentagrams) the center of cyclonic eddy 1 (CE 1) during 1–20 April 2019. **b** The locations of (yellow dots) the BGC-Argo float (WMO number 2902756) and (orange pentagrams) the center of cyclonic eddy 2 (CE 2). **c** The locations of the BGC-Argo floats along the front during 21–27 April 2019. The yellow line is the float trajectory from 21 April to 3 May 2019. **d** The same as **c** but during 28 April-3 May 2019. Background color and isolines represent sea surface chlorophyll concentrations and sea level anomalies, respectively. The arrows are the geostrophic velocity anomalies. Source data are provided as a Source Data file.

Two BGC-Argo floats (WMO numbers 2902753 and 2902756; Fig. 1a, Table S1 and Text S1) were deployed in two cyclonic eddies (identified as CE 1 and CE 2; Fig. 1a, Table S2 and Text S2) to collect temperature, salinity, nitrate, chlorophyll-*a* (Chl), dissolved oxygen (DO), particulate backscattering coefficients at 700 nm (b_bp_(700)) and photosynthetically available radiation (PAR). From 30 March to 20 April 2019, float 2902753 moved gradually from the CE 1 core to the CE 1 edge with the relative position r/R (r and R were the distance of the float to the eddy center and the eddy radius, respectively) increasing from ~0 to 2 (Fig. 1b and Fig. 2a). During this time, the sea level anomaly (SLA) at the float position increased gradually from −0.1 m to 0.1 m, reflecting the SLA change from the CE 1 center to its periphery (Fig. 1b). From 21 April to 3 May 2019, this float moved along one side of a submesoscale front and experienced the intensifying and decaying stages of the front (Fig. 1b). Float 2902756 was deployed and trapped in the core region of CE 2 and experienced the intensifying and mature stages of the eddy until 22 April 2019 (Figs. 1c and 2b).

**Fig. 3:**
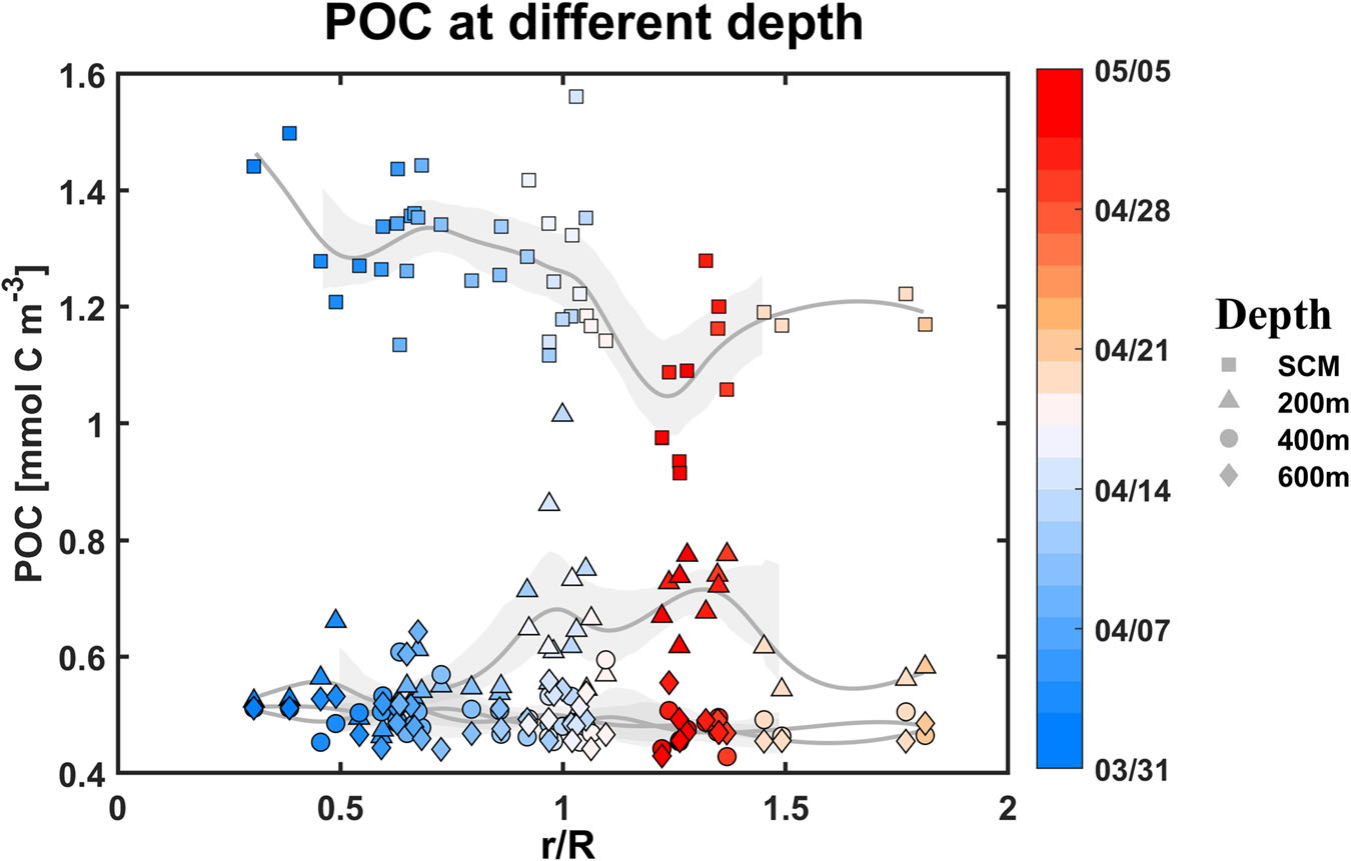
Radial distribution of particulate organic carbon (POC) concentration in CE 1 at the subsurface chlorophyll maximum depth (*Z*_SCM_), 200 m, 400 m and 600 m. Colors indicate the sampling dates. The square, triangle, dot and diamond symbols represent the depths of the SCM, 200 m 400 m and 600 m, respectively. The solid curves and shades are the regression results and the 95% confidence interval at each depth, respectively. Source data are provided as a Source Data file.

The euphotic depth (*Z*_Eu_) was on average ~159 m within CE 1 and changed slightly along the radial direction (Fig. S1). The floats may have captured the spatial variability of tracers for CE 1 as the location of the floats changed from inside to outside the eddy. The satellite-derived sea surface Chl was averaged over the float sampling period and then interpolated onto the relative locations of the float in CE 1 to compare with that measured by the float. The float-measured Chl was in agreement with that derived from satellites in the radial direction (Fig. S2a), with a correlation of 0.39 (*p* = 0.01). In contrast, the temporal evolution of satellite-derived Chl averaged inside CE 1 was different from that of float-measured Chl (Fig. S2b), as indicated by a low correlation (R = −0.18, *p* = 0.24). Therefore, the float-measured tracers during this period can be used to represent the spatial variability of CE 1 along the radial direction. The radial POC distributions of CE 1 were constructed from BGC-Argo measurements (Fig. 3). From the center to the edge, the POC distributions in CE 1 decreased at the subsurface chlorophyll maximum depth (*Z*_SCM_; on average 117.0 ± 9.9 m; Fig. S1) and increased at 200 m depth. The radial POC distributions at 400 m and 600 m changed slightly compared with those at 200 m. As the float moved to the edge of CE 1, the temperature displayed an increasing trend, while nitrate, Chl and POC showed a decreasing trend (Fig. S3 and Table S3). This result suggested that nutrients in the euphotic zone were enhanced by the uplift of isotherms within this cyclonic eddy^28^, leading to higher POC concentrations in the CE 1 center than at its edge (Fig. 3). The POC at 200 m reached its peaks at the edge of r/R = ~ 1 and 1.3, which was likely due to subduction of POC from the productive layer by the front^17,19,20,23^. This observation further supported that the radial distributions of POC measured by the float reflected the spatial variability of the eddy.

### POC flux by an intensifying cyclonic eddy (CE 2)

We defined the intensifying and mature stages of CE 2 based on the temporal change of SLA averaged inside CE 2. Data from float 2902756 were then classified into intensifying-stage and mature-stage measurements by comparing the change of SLA near the float with that averaged inside CE 2. From 6 April to 14 April 2019, the CE 2 intensified with gradually decreasing SLA values (Fig. S4). The SLA near the float also decreased gradually from −0.03 m to -0.09 m until 14 April, almost synchronously with the SLA decrease inside CE 2 (Fig. S4). Additionally, the SLA change of the float was positively correlated with the change in temperature and negatively correlated with the changes in NO_3_^−^, Chl and POC at the *Z*_SCM_ (Fig. S5 and Table S3), implying that the float captured the eddy’s intensifying stage. In stages other than the intensifying stage, the correlations between changes in SLA and changes in temperature, NO_3_^−^, Chl, and POC at the *Z*_SCM_ were generally low or even insignificant (Fig. S5). From April 15 to April 22, the SLA near the float and that averaged inside CE 2 remained at extremely low values, indicating that the eddy had reached a mature stage. Except for a fluctuation in the relative position (r/R) during the first few days, the float’s r/R value inside CE 2 remained approximately 0.7 until 22 April 2019 (Fig. 1c). We thus used the float measurements during 6–14 April to describe the eddy’s intensifying stage and measurements during 15–22 April to describe the eddy’s mature stage.

Since float 2902756 traveled in a quasi-Lagrangian manner inside the eddy during the intensifying and mature eddy stages, we assumed that the variability measured by the float was mostly attributable to temporal changes of the eddy. The POC concentration was first integrated from the base of the mesopelagic layer (900 m) to depth z (integrated POC; noted as *P*(z) in Eq. (3)), and then the rate at which POC changed over time (denoted as *E*(*z*)) was computed by Eq. (4). With multiple sampling profiles, a linear least squared method (slope of the regression line) was used to estimate *E*(*z*) during the intensifying (denoted as *E*_EI_(*z*)) and mature (denoted as *E*_EM_(*z*)) stages.

**Fig. 4:**
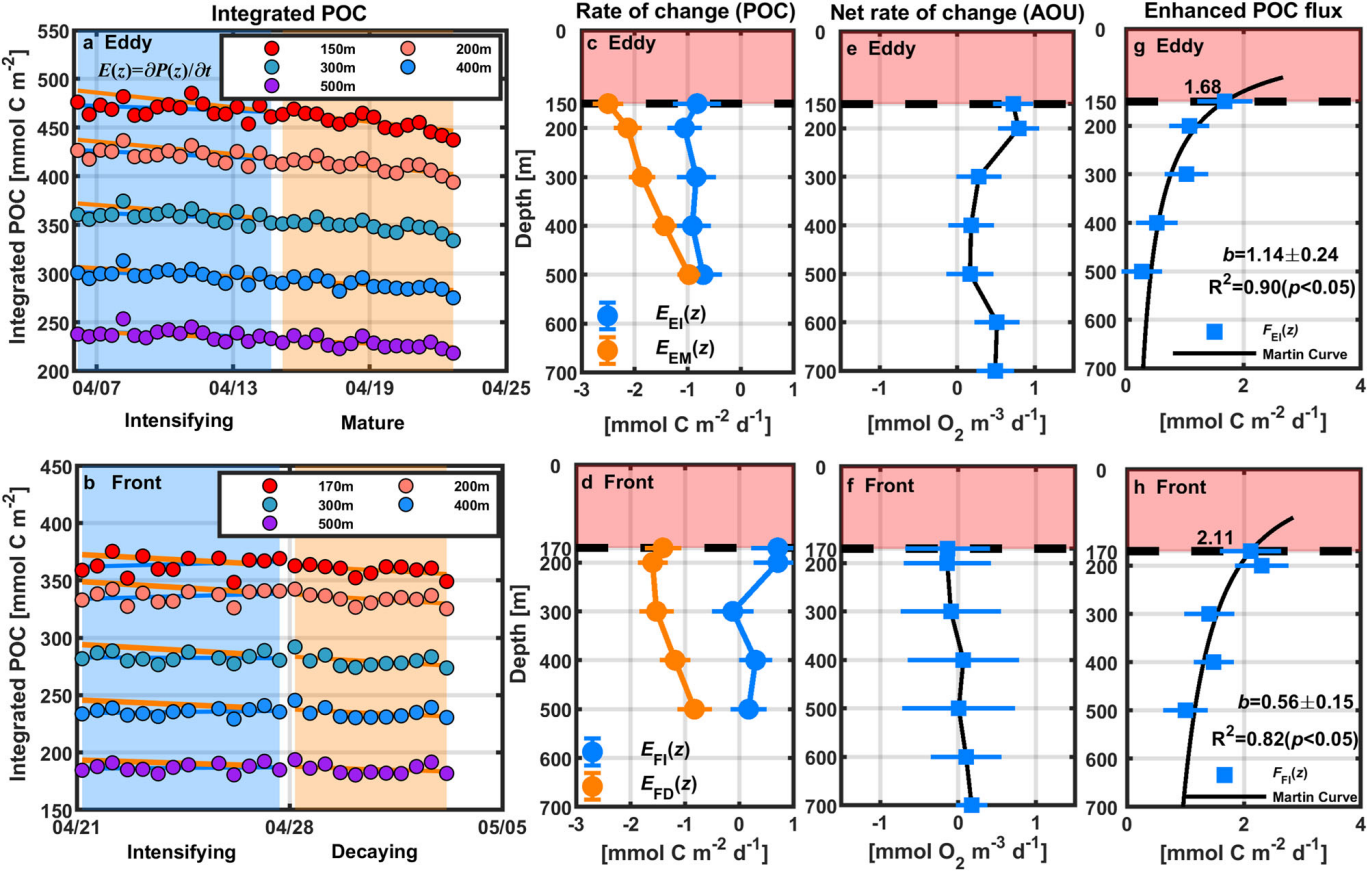
Vertical particulate organic carbon (POC) export fluxes induced by an intensifying cyclonic eddy and a submesoscale front. **a**, **b** Integrated POC from the base of the mesopelagic layer (900 m) to depth z, with z = [150 200 300 400 500] m for CE 2 and z = [170 200 300 400 500] m for the front. **c**, **d** Changing rate of the integrated POC over time. **e**, **f** Net rate of change of the averaged AOU. **g**, **h** Enhanced POC export fluxes. (**a**, **c**, **e** and **g**) are in CE 2, and (**b**, **d**, **f** and **h**) are at the front. The blue and orange solid lines in **a**, **b** represent linear regression lines of the POC inventories during the intensifying and mature (or decaying) stages, respectively. The blue and orange areas in **a**, **b** correspond to the intensifying and mature (or decaying) stages, respectively. The black dashed lines and error bars in **c**–**h** correspond to the euphotic zone depth and one standard deviation, respectively. The red areas in **c**–**h** represent the euphotic zone. The *b* values in **g**–**h** are shown as the mean ± 1 standard deviation. Source data are provided as a Source Data file.

Negative *E*_EM_(*z*) values were present at all depths (Fig. 4a, c), which suggested that the POC removal process was dominant for the POC concentration change during the mature stage of the eddy, likely caused by microbial degradation in the mesopelagic layer^9,49^. Consistent with other observational studies^35^, the rate of change of POC decreased gradually with depth in the mesopelagic layer. However, there was a significant difference between the *E*_EI_(*z*) and *E*_EM_ (*z*) profiles (Fig. 4c). The *E*_EI_(*z*) during the intensifying stage of the eddy was larger than the *E*_EM_(*z*), and their difference decreased with depth. This suggested a decrease in the rate of change of POC in the mature stage with respect to the intensifying stage, which could be induced by changes in the POC export flux from the top and/or the POC consumption rate in the water column. In addition, the net rate of change of apparent oxygen utilization (AOU) was positive in the upper mesopelagic layer because AOU changed more during the intensifying stage than during the mature stage (Fig. 4e). This may imply enhanced POC consumption by microbial activity during the intensifying stage. The POC inventory change in the water column is the combined result of the POC flux supplied from the top and the POC consumption in the water column. It is thus very likely that the POC export flux was higher during the intensifying stage than during the mature stage, given that both the rate of change of POC and the average AOU were larger during the intensifying stage than during the mature stage.

The enhanced net POC flux during the eddy’s intensifying stage was then calculated using Eq. (5). Note that this flux should be smaller than the actual POC export flux, as POC consumption also increased during the intensifying stage compared with the mature stage (presented as a positive net rate of change of AOU) (Fig. 4e). An enhanced net POC flux of 1.68 ± 0.3 mmol C m^−2^ d^−1^ at *Z*_Eu_ was obtained, which was comparable to the estimates of POC export flux in the NPSG by sediment traps (1.92 mmol C m^−2^ d^−1^ by Grabowski et al.^50^ and 2.5 ± 0.7 mmol C m^−2^ d^−1^ by Zhou et al.^31^) and smaller than the estimates of POC export flux in the subpolar northwest Pacific Ocean using the radioactive technique (3.25 and 11.08 mmol C m^−2^ d^−1^ by Buesseler and Boyd^51^; Table S4). For eddies, significant POC export is not sustained throughout their lifespan and is often associated with the physical dynamics of eddies^42,52^. Observations have shown that intensifying eddies exhibit a large biological response, while eddies in the mature stage do not show a significant biological response or high export flux^29,52^. We assumed that the magnitude of the export flux during the eddy’s mature stage was low, so the enhanced net POC flux was considered as the approximation of the actual POC flux here. The enhanced POC flux may underestimate the actual POC flux during the eddy’s intensifying stage as *F*_EI_(*z*) was a flux anomaly relative to the eddy’s mature stage. Because the Martin curve has long been used as the standard for quantifying POC attenuation across the global ocean, *F*_EI_(*z*) values at different depths in the mesopelagic layer were fitted to the Martin curve (Eq. (8)) to estimate the attenuation coefficient *b*, yielding a value of 1.14 ± 0.24, which was similar to those estimated in subtropical gyres and higher than those estimated at high latitudes^10,39,50,51^ (Fig. 4g and Table S4).

### POC flux by an intensifying submesoscale front

The sea surface temperature (SST) distribution revealed structures of a submesoscale thermal front with an SST gradient magnitude (GM) of ~0.2 °C km^−1^ outside of CE 1 from 21 April to 3 May 2019 (Fig. S6a–d). During this period, float 2902753 drifted along the warm (upwelling) side of the front (Fig. S6). This was further supported by a Lagrangian particle tracking experiment, which suggested that the float drifted in a quasi-Lagrangian manner along one side of the front (Text S3). The front exhibited a higher temperature in the upper 300 m and lower salinity in the upper 150 m compared to the CE 1 core, indicating that similar water masses were sampled in the frontal region. The T-S diagram demonstrated coherent physical characteristics from 21 April to 3 May 2019 (Fig. S7). All these results suggested that the float sampled similar water masses along the warm side of the front.

The float presented a small Richardson number R_i_ (R_i_≈2) in the frontal region, indicating ageostrophic motions at play to a depth of ~250 m (Fig. S8b). The ageostrophic motions at the front can also be indicated by the increased finite-size Lyapunov exponent (FSLE) distributions at the front (Fig. S8c). We defined different stages of the front by examining changes of GM at the float locations. From 21 April to 27 April, the GM was elevated and larger than the background value, indicating that the float was sampling the front’s intensifying stage. From 28 April to 3 May, the GM decreased to the background value, indicative of the front’s decaying stage (Fig. S6e). In addition, the R_i_ between the MLD and 250 m depth was smaller during the intensifying stage than during the decaying stage (Fig. S8b). Consistent with the change of R_i_, the magnitude of the lateral buoyancy frequency (M^2^) increased during the intensifying stage and decreased during the decaying stage (see the M^2^ envelope in Fig. S8c). During the intensifying stage, submesoscale dynamics were prone to generating secondary circulations across the front, which in turn changed the temperature and salinity distributions along the front. Due to the heterogeneous evolution and distribution of the front over time, M^2^ increased in magnitude along the front. The temporal changes in R_i_ and M^2^ suggested that submesoscale motions were more active during the intensifying stage than during the decaying stage. During the intensifying stage, the POC inventory in the euphotic zone was elevated (Fig. S9b). Moreover, large particles were detected by spikes of backscattering coefficients in the mesopelagic layer^8^ (Fig. S9c).

Given the two distinct periods sampled by the float, the rate of change of integrated POC during the intensifying (denoted as *E*_FI_(*z*)) and decaying (denoted as *E*_FD_(*z*)) stages of the front were calculated (Fig. 4b, d, f, h). The distribution of *E*_FD_(*z*) was similar to that of *E*_EM_(*z*), which decreased with depth (Fig. 4b, d). Following the same method applied to the eddy, the enhanced net POC flux induced by front intensification (denoted as *F*_FI_(*z*)) was estimated by Eq. (6). In contrast to the eddy, the net rate of change of AOU was very small in the mesopelagic layer (Fig. 4f). The small net rate of change of AOU implies relatively stable POC consumption rates during the intensifying and decaying stages. The enhanced net POC flux (*F*_FI_(*z*)) was thus mostly contributed by the increased POC export flux during the front intensifying stage (Fig. 4h). The increased POC export flux at the base of the euphotic zone was then approximately estimated as 2.11 ± 0.4 mmol C m^−2^ d^−1^, higher than that in the eddy’s intensifying stage. Similar results have been reported in the NPSG, which showed a higher POC flux at a submesoscale front than in a cyclonic eddy nearby (2.2 mmol C m^−2^ d^−1^ versus 1.2 mmol C m^−2^ d^−1^ in Guidi et al. ^41^). In addition, an attenuation coefficient *b* of 0.56 ± 0.15 was estimated by fitting *F*_FI_(*z*) to Eq. (8), which was smaller than that for the intensifying cyclonic eddy, implying deeper transfer of POC in the frontal region.

## Discussion

Previous studies have suggested that the POC flux was low and attenuated quickly within subtropical gyres^10,11^. However, the fast-varying nature of carbon export induced by submesoscale processes makes it difficult to evaluate by ship-based measurements or moored traps. With the flexible design of the high-frequency BGC-Argo sampling schemes, we demonstrated that there were additional POC export fluxes during the intensifying stages of eddies and fronts relative to their mature and decaying stages, respectively. As mesoscale and submesoscale features are ubiquitous in the gyres, their episodic intensifying processes might enhance the basin-scale POC export.

The POC export efficiency was found to be lower in the intensifying eddy (T_eff_ = 12%) than in the intensifying front (T_eff_ = 37%). The T_eff_ of the intensifying eddy was comparable to previous estimates in the NPSG (T_eff_ = 10% on average from Marsay et al.^10^, Buesseler and Boyd^51^ and Grabowski et al.^50^), whereas the T_eff_ of the intensifying front was much higher, suggesting efficient carbon export in frontal regions. This high transfer efficiency at the front is comparable with the regional results of Guidi et al.^41^ conducted in a frontal region (Table S4).

Two mechanisms affecting POC export at fronts have been shown to be important in previous studies^14–24,35^. The first mechanism is through biological carbon pump. Strong submesoscale flows can enhance the vertical nutrient supply^14,15,33^, alter the phytoplankton community structure^32,35^, and generate particles sinking to depth^26,41^. Some of the particles generated through this mechanism have the potential to penetrate the pycnocline and reach the mesopelagic layer via gravitational sinking^16,36,37^. The second mechanism is through physical subduction, by which particles are carried downward with the water mass^17–23^. For this mechanism, the depth the particle can reach is largely determined by the subduction velocity and the ventilation depth^17–23^.

According to the statistical relationship of Marsay et al.^10^, the coefficient *b* in the NPSG should be limited to a narrow range between 1.54–1.83, as the median temperature recorded by BGC-Argo floats ranged from 20–25 °C. However, our study obtained a smaller *b* value (0.56 ± 0.15) that was outside of the range given by Marsay et al.^10^ that used a different method to estimate *b* and did not explicitly consider submesoscale dynamics. The coefficient *b* was controlled not only by the remineralization rate but also by the particle properties that are further linked with the phytoplankton community structure in the euphotic zone^38,39,53,54^. Intense frontal dynamics, such as frontogenesis, have been shown to be able to stimulate the growth of large phytoplankton communities, through which food web dynamics can generate large and rapidly sinking particles^32,36^. The slow attenuation was likely caused by large particles sinking out of the euphotic zone during front intensification. Although this study lacked POC size structure analysis, a signal of fast sinking by large particles was reflected by the b_bp_(700) spikes during the front intensifying stage (Fig. S9c). Another mechanism suggested by a previous study^41^ was the surface convergence caused by horizontal stirring at the front, which in turn increased the concentrations of buoyant nitrogen fixer (*Trichodesmium* spp.) and chlorophyll at the surface and stimulated downward particle transport. As the surface chlorophyll concentration was relatively lower at the float-sampled frontal region than in other regions (Fig. 2), this convergence-driven mechanism may not be dominant here.

The high transfer efficiency of POC at the front could also result from the physical subduction of POC by intense submesoscale vertical velocities. Two lines of evidence have revealed that the subduction pump contributed to carbon export at the front. Firstly, frontal dynamics were responsible for the elevated POC at 200 m depth at the CE 1 edge of r/R = 1 and 1.3 (Fig. 3). At the positions of r/R = 1 and 1.3, the float was right on the filaments of high FSLE accompanied by high M^2^ and small R_i_ in the vertical direction, suggestive of ongoing submesoscale vertical motions (Fig S8). Concurrent with the physical dynamics, there were patches of positive spiciness and POC anomalies between *Z*_Eu_ and ~250 m (Fig. S10), which was evidence of subduction pump as suggested in previous studies^17,19,20,23,55^. The patches of positive spiciness and POC anomalies were stronger in the front’s intensifying stage than in the decaying stage (Fig. S10). Secondly, the POC fluxes between *Z*_Eu_ and ~250 m influenced by the subduction pump were higher than those at *Z*_Eu_ and those below 250 m (Fig. S11). We tested different ways to fit the POC flux attenuation coefficient (*b*) with and without considering the POC flux in the subduction layer (*Z*_Eu_ ~ 250 m). The *b* value was approximately 0.65 excluding the POC flux in the subduction layer, which was higher than that considering the subduction layer (*b* = 0.56) but was still lower than those estimated in eddies and normal conditions. This suggests that submesoscale dynamics affect POC export flux not only through physical subduction but also through biological processes by modulating particle properties (e.g., size, sinking speed, and composition). These results emphasize the complexity of submesoscale dynamics at play in enhancing POC transfer efficiency. Overall, we showed that physical subduction was mostly limited to depths of <250 m. Below 250 m, POC export in the region could be largely attributable to the gravitational sinking of particles.

**Fig. 5:**
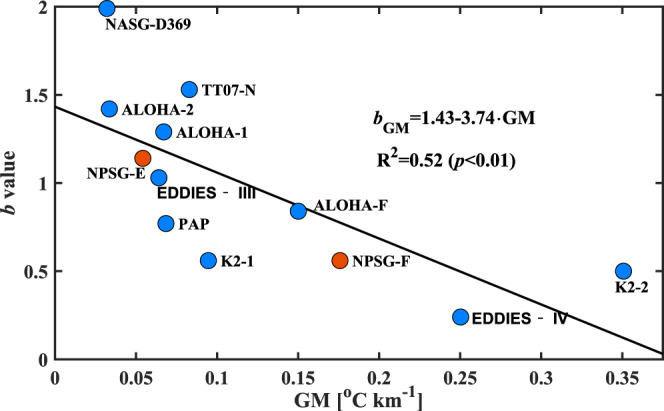
Relationship between the sea surface temperature (SST) gradient magnitude (GM) and the attenuation coefficient (*b*) obtained by compiling previous data in subtropical gyres (blue points, Table S4, Fig. S12). Red points are estimates from this study. Source data are provided as a Source Data file.

Our results demonstrated that the POC flux attenuation in the cyclonic eddy was comparable to the regional mean condition or at least within the range of subtropical gyres, which was different from that estimated at the submesoscale front. The impact of fronts on POC transfer efficiency at the basin scale and global scale is still an open question. We combined previous studies that have estimated POC attenuation coefficients in the North Pacific and North Atlantic gyres (Table S4, Fig. S12). A strong correlation between the *b* value and GM was found (R^2^ = 0.52, *p* < 0.01; Fig. 5). The derived relationship between the flux attenuation coefficient and the front intensity could be applied to large-scale applications. Estimations based on this relationship suggest that the average POC export flux at 1000 m may be underestimated by ~20–58% in the NPSG if the contribution of submesoscale fronts is ignored (Text S4). The estimations suggest that submesoscale fronts are important in setting the spatial and temporal variations in POC transfer efficiency. Note that the estimation in this study is based on the relationship between the *b* value and GM. In much of the world’s ocean, temperature variations play a leading role in controlling the spatial near-surface density gradients, while salinity plays a secondary role^56^. Therefore, the satellite-derived SST gradient as an indicator of submesoscale fronts has been suggested to be useful to investigate the spatiotemporal variation in submesoscale fronts. However, in regions that are strongly influenced by freshwater runoff and rain or have close-to-freezing surface temperatures, salinity variations govern the near-surface density gradients. The GM method cannot identify density fronts governed by salinity variations^57^. It may thus introduce uncertainties in the identified front and the estimated *b* value.

Previous carbon cycle studies have suggested that the attenuation of the POC flux is determined by the rate of POC production in the euphotic zone, the sinking velocity of particles, and the rate of POC consumption in the mesopelagic layer^5–10,38,39,49–51,53,54,58^. Our estimation suggests that submesoscale fronts increase the POC transfer efficiency in the NPSG through biological gravitational pump and physical subduction. However, it should be kept in mind that other processes that can potentially modulate the *b* value (e.g., microbial POC consumption^49,58^, POC fragmentation by zooplankton^8^) were not explicitly included in the estimation. Quantifying the mesopelagic fragmentation processes revealed that roughly half of the observed flux attenuation was due to the fragmentation of large particles into small particles^8^. Ngyuen et al. showed that microbial dynamics may play a significant role in shaping the POC flux profiles observed in global oceans^58^. However, how these dynamics respond to submesoscale fronts is still unclear. Future work including dynamics such as microbial consumption and zooplankton disaggregation of the particles is needed, and may allow for better constraints on the relative contributions of submesoscale fronts to the basin-scale carbon transfer efficiency.

Submesoscale processes are ubiquitous in the ocean. This study demonstrated the unique application of autonomous floats for studying these features by sampling at high frequencies, highlighting the importance of resolving their life stages to better understand the roles in affecting the biological carbon pump. Although great efforts have been made to constrain the uncertainties in carbon sink and source estimations^9,40^, the upper-layer carbon export is still reported to be up to two to three times less than the mesopelagic carbon demand by heterotrophic metabolism^9,40^, highlighting a requirement to reassess the unaccounted pathways that deliver carbon to deep waters^17^. This study showed a higher carbon transfer efficiency along the front compared to the mean of previous studies (T_eff_=37% vs. T_eff_ = 10%), suggesting that submesoscale dynamics could potentially result in closer agreement between sources and sinks of carbon and need to be considered when addressing the imbalance in mesopelagic carbon budgets.

## Methods

### BGC-Argo measurement and calibration

Data were collected by sensors equipped on the BGC-Argo floats from depths between ~900 m and the surface. The floats profiled twice a day. The floats sampled the first 30 m at a 0.2 m resolution, between 30–400 m at a 1 m resolution and below 400 m at a 5 m resolution. All profile data were interpolated at a 5 m resolution. Before deployment, the dark currents of the chlorophyll fluorometer and backscattering sensor were corrected (reassigned) based on the dark-current measurements, by masking the optical windows with black tape. The dark-corrected Chl and bbp profiles were smoothed using a 5-point moving median filter to minimize high frequency noise^44^. The spike of b_bp_(700), a qualitative proxy index for large particles, was calculated as the difference between the raw and smoothed b_bp_(700)^8^. The nitrate distributions from the BGC-Argo floats were calibrated using ship-based nitrate measurements taken along the BGC-Argo trajectories (Text S5 and Fig. S13). The ship-based POC concentrations were also measured during float deployment. Then, a localized statistical relationship between the b_bp_(700) values measured by the BGC-Argo floats and the ship-based collection of POC concentrations was derived (Text S5, Fig. S14; a total of 32 data points, R^2^ = 0.39, *p* < 0.01):

1 $${{{{{\rm{POC}}}}}}\,({{{{{\rm{mmol}}}}}}\; {{{\rm{C}}}}\,{{{{{{\rm{m}}}}}}}^{-3})={2083.17\cdot {{{{{\rm{b}}}}}}}_{{{{{{\rm{bp}}}}}}}(700)+0.06$$

The mixed layer depth (MLD) was computed as the depth where the potential density was different from the sea surface density by 0.03σ_θ_ following Jing et al.^59^. The *Z*_Eu_ was calculated as the depth where PAR was 1% of its surface value^60^. The AOU was calculated as the difference between the saturated and observed DO:

2 $${{{{{\mathrm{AOU}}}}}}={{{{{{\mathrm{DO}}}}}}}_{{{{{{\mathrm{sat}}}}}}}-{{{{{\mathrm{DO}}}}}}$$

where DO_sat_ is the saturated DO computed using the measured temperature and salinity. To identify subsurface features resulting from subduction pumps, spiciness that describes thermohaline perturbations not included in potential density was estimated using the BGC-Argo-derived temperature and salinity data. As suggested by Omand et al.^19^, subsurface water masses originating from the euphotic zone were identified as subsurface anomalies in vertical POC and spiciness distributions.

### Other data

Ship-based nitrate and POC measurements were collected at 10 stations near the BGC-Argo trajectories for nitrate calibration and POC calculations (Text S5 and Table S5). Nitrate was measured onboard using a Four-channel Continuous Flow Technicon AA3 Auto-Analyzer (Bran-Lube, GmbH). The POC data were determined using an Elemental vario EL cube CHNS Analyzer, of which the uncertainty was <10%^42^.

Daily SLA and sea surface Chl were obtained from the Copernicus Marine Environment Monitoring Service (CMEMS) and the Ocean Color Climate Change Initiative (OC-CCI) project, respectively. The dataset from the Mesoscale Eddy Trajectory Atlas was identified and tracked using the methods provided by Pegliasco et al.^12^ and obtained from the Archiving, Validation, and Interpretation of Satellite Oceanographic Data (AVISO) service. Satellite-derived SST data at a resolution of ~4.5 km (available from the CMEMS) were used to determine the SST gradients of the oceanic fronts.

### The rate of change of integrated POC

Since the float drifted along the front or in a relatively fixed position of CE 2, we expected that the float sampled similar water masses over time. The POC concentrations derived from the float estimations were integrated from the base of the mesopelagic layer (900 m) to an upper mesopelagic depth z:

3 $$P(z)={\int_{900m}^{z}}{{{{{\rm{POC}}}}}}\cdot {dz}$$

where *P*(*z*) is the integrated POC, *z* = [*Z*_Eu_, 200 m, 300 m, 400 m, 500 m]. The rate of change of integrated POC (denoted as *E*(*z*)) was thus considered as the net rate at which POC accumulated between depth z and the base of the mesopelagic layer:

4 $$E(z)=\partial P(z)/\partial t$$

where *E*(*z*) can be interpreted as the balance between the POC flux supply from depth z and the POC consumption between depth z and the base of the mesopelagic layer, given that the POC flux at the base of the mesopelagic layer is generally negligible^10,46^. The *E*(*z*) was calculated using a linear least squared method (slope of the regression line). Because the balance between supply and consumption is the driver of *E*(*z*) changes, we estimated *E*(*z*) during the intensifying and mature (decaying) stages of the eddy (front). The *E*(*z*) during the intensifying and mature stages of the eddy were denoted as *E*_EI_(*z*) and *E*_EM_(*z*), respectively, and those during the intensifying and decaying stages of the front were denoted as *E*_FI_(*z*) and *E*_FD_(*z*), respectively. The uncertainty of the linear least squared estimations was shown in Table S6. The enhanced net POC fluxes during the intensifying stages of the eddy (denoted as *F*_EI_(*z*)) and the front (denoted as *F*_FI_(*z*)) were calculated as:

5 $${F}_{{{{{{\rm{EI}}}}}}}(z)={E}_{{{{{{\rm{EI}}}}}}}(z)-{E}_{{{{{{\rm{EM}}}}}}}(z)$$

6 $${F}_{{{{{{\rm{FI}}}}}}}(z)={E}_{{{{{{\rm{FI}}}}}}}(z)-{E}_{{{{{{\rm{FD}}}}}}}(z)$$

A Lagrangian particle tracking experiment was conducted to test whether the float drifted along the front (Text S3). To further verify that the float sampled similar water masses, the physical characteristics along the front were analyzed (Fig. S7).

The AOU multiplied by the Redfield ratios represents the heterotrophic respiration of the total organic carbon (TOC), 50–90% of which is dissolved organic carbon^48,61,62^. Because the ratio of the POC to TOC and the Redfield ratios in the study region were not measured, we did not use the AOU to reconstruct the remineralized POC and only used the change of AOU over time to demonstrate possible changes in remineralization between different stages. The average AOU from the base of the mesopelagic layer (900 m) to an upper mesopelagic depth z was calculated as

7 $${{{{{\rm{average}}}}}}\,{{{{{\rm{AOU}}}}}}=\frac{1}{(900 \, m-z)}{\int_{900 \, m}^{z}}{{{\rm{AOU}}}}\cdot {dz}\,$$

where *z* = [*Z*_Eu_, 200 m, 300 m, 400 m, 500 m]. The net rate of change of AOU defined as the difference of the temporal rate of AOU between the intensifying and mature (decaying) stages of the eddy (front) was thus estimated using the averaged AOU.

### POC flux attenuation

The attenuation of POC flux with depth is quantified by a Martin curve^63^:

8 $$F\left(z\right)=F({Z}_{{{{{{\rm{Eu}}}}}}})\cdot {\left(\frac{z}{{Z}_{{{{{{\rm{Eu}}}}}}}}\right)}^{-b}$$

where *F*(*z*) is the POC flux at depth *z*, normalized to flux $F({Z}_{{{{{{\rm{Eu}}}}}}})$ at euphotic depth *Z*_Eu_, and *b* is the attenuation coefficient.

### The SST gradient

We use the SST gradient to indicate the strength of front. The SST gradient magnitude (GM) is calculated following Belkin and O’Reilly^64^:

9 $${{{{{\rm{GM}}}}}}=\sqrt{{\left({G}_{x}\right)}^{2}+{\left({G}_{y}\right)}^{2}}$$

where *G*_*x*_ and *G*_*y*_ are the zonal and meridional SST gradients that can be quantified as:

10 $${G}_{x}=\frac{1}{{d}_{x}}\left[\begin{array}{ccc}-1 & 0 & 1\\ -2 & 0 & 2\\ -1 & 0 & 1\end{array}\right] * {{{{{\rm{SST}}}}}}$$

11 $${G}_{y}=\frac{1}{{d}_{y}}\left[\begin{array}{ccc}1 & 2 & 1\\ 0 & 0 & 0\\ -1 & -2 & -1\end{array}\right] * {{{{{\rm{SST}}}}}}$$

where *d*_*x*_, *d*_*y*_ and ∗ are the zonal and meridional distances between successive pixels within the SST data and the convolution sign, respectively. Using satellite-derived SST data, the GM at the front where the BGC-Argo passed through (Fig. S6c–e) and at the locations of the sampling site for in-situ POC flux measurements (Fig. 5) were calculated. To extrapolate the relationship between the *b* value and GM for the NPSG, the spatial distributions of GM during 2003–2019 were also calculated (Text S6).

### The FSLE field

We use the finite-size Lyapunov exponent (FSLE) to illustrate the frontal region encountered by the BGC-Argo. Daily FSLEs with a spatial resolution of 0.04° were obtained from AVISO. The FSLE is defined as the inverse time of separation of two particles from their initial distance δ_0_ to final distance δ_f_^65^. The particles were advected by altimetry velocities and their trajectories were computed by backward-time integration of the altimetry velocities. The FSLE has been suggested to be particularly suited to diagnose filamentary processes generated by mesoscale horizontal shear and strain deformation^65^. Large backward FSLE values represent regions of strong submesoscale transport^66^. As such, the FSLE has been employed to delineate submesoscale structures^32,66^. The FSLE time series was extracted along the path of float 2902753 to capture the strain field and was then compared to the lateral buoyancy frequency (M^2^) sampled by the float.

### Reporting summary

Further information on research design is available in the Nature Portfolio Reporting Summary linked to this article.

### Supplementary information

Supplementary Information

Peer Review File

Reporting Summary

### Source data

Source Data

## Data Availability

The BGC‐Argo data used in this study can be downloaded from the BGC-Argo FTP server (ftp://ftp.ifremer.fr/ifremer/argo/dac/csio/2902753/ and ftp://ftp.ifremer.fr/ifremer/argo/dac/csio/2902756/). Daily SST and SLA data were downloaded from the EU Copernicus Marine Environment Monitoring Service (CMEMS; 10.48670/moi-00168 and 10.48670/moi-00148). The eddy trajectory information was downloaded from AVISO’s Mesoscale Eddy Trajectory Atlas (https://www.aviso.altimetry.fr/en/data/products/value-added-products/global-mesoscale-eddy-trajectory-product.html). The FSLE data was download via https://tds.aviso.altimetry.fr/thredds/fsle/dataset-duacs-dt-global-allsat-madt-fsle.html. The ocean color data were downloaded from the OceanColor-CCI website (https://www.oceancolor.org). The data used in this study are available in the ZENDO database that can be accessed through 10.5281/zenodo.10294902. Source data are provided with this paper.

## Code availability

The MATLAB 2022 is used for plotting. The code used for analysis is available at ZENODO via 10.5281/zenodo.10294902. The MATLAB code of GRAMM and M_MAP is obtained from https://github.com/piermorel/gramm and https://www.eoas.ubc.ca/~rich/map.html. The TRACMASS code for Lagrangian particle tracking is available at https://www.tracmass.org/index.html.
