# Supplementary material for: Efficient biological carbon export to the mesopelagic ocean induced by submesoscale fronts
Source: Nat Commun. 2024 Jan 17;15:580. doi: 10.1038/s41467-024-44846-7 (PMC10794176; doi:10.1038/s41467-024-44846-7)
Supplement: Supplementary file 1 — Supplementary Information [file 41467_2024_44846_MOESM1_ESM.pdf]

Supplementary Information:

Efficient biological carbon export to the mesopelagic ocean induced by submesoscale fronts

Mingxian Guo[1§], Xiaogang Xing[2§], Peng Xiu[3*], Giorgio Dall'Olmo[4], Weifang Chen[3], Fei Chai[3]

[1]State Key Laboratory of Tropical Oceanography, South China Sea Institute of Oceanology, Chinese Academy of Sciences, Guangzhou, China

[2]State Key Laboratory of Satellite Ocean Environment Dynamics, Second Institute of Oceanography, Ministry of Natural Resources, Hangzhou, China

[3]State Key Laboratory of Marine Environmental Science & College of Ocean and Earth Sciences, Xiamen University, Xiamen, China

[4]Istituto Nazionale di Oceanografia e di Geofisica Sperimentale - OGS, Trieste, Italy

*Corresponding author: Peng Xiu (pxiu@xmu.edu.cn)

[§]M.G. and X.X. contributed equally to this work

Text S1. BGC-Argo deployment

The eddy field was monitored by satellite-derived SLAs and sea surface chlorophyll concentrations so that BGC-Argo floats could be deployed in the center of the eddy. Float 2902753 was deployed in cyclonic eddy CE 1, which lasted until 21 April 2019. From 21 April to 3 May, this float propagated along one side of a submesoscale front. Float 2902756 was deployed in cyclonic eddy CE 2 on 25 March and remained inside CE 2 until 22 April 2019. At the beginning of the deployment, the two floats profiled once a day at a parking depth of 200 m. From 3 April to 3 May 2019 (28 March to 26 April 2019), float 2902753 (2902756) collected profiles twice a day to cover the depth from the sea surface to ~1000 m. The average sampling distances during the floats inside CE 1 and CE 2 were 6.45 km and 6.2 km, respectively.

Text S2. Eddy tracking results

Based on SLAs, eddies were tracked, and their trajectory products were downloaded from AVISO+ with the algorithm of Pegliasco et al.[1] for the delayed-time product. Eddy information includes the location of the eddy center, the outline of the eddy edge, the radius R and the amplitude. Using this eddy dataset, the parameters of the eddies sampled by the BGC-Argo floats were computed (Table S2). According to the satellite-derived eddy identification and tracking dataset, the characteristics of eddies CE 1 and CE 2, including the eddy radius, amplitude and propagation speed, were averaged over the period during which they were sampled by the floats, as well as over the whole lifespan of the eddy.

Text S3. Lagrangian particle tracking experiment at the front

To investigate whether the float drifted along the front, a numerical particle tracking experiment was conducted using the TRACMASS Lagrangian trajectory code[2,3]. The particle was released at the same location as the float on 21 April 2019 and transported purely by surface current. Here, we run the code with the current velocity derived from AVISO. The Lagrangian particle trajectory derived from the particle tracking model demonstrated that the virtual particle starting at the location of the float moved along the front (Fig. S15). The float moved slower than the particle but mainly followed its path, suggesting that the float drifted in a quasi-Lagrangian manner during the analysis period. The difference in moving velocity is likely that the particle was only transported by the surface current, whereas the float was moved by both the surface and deep currents.

Text S4. POC transfer efficiency affected by fronts in subtropical gyres

To illustrate the potential implications of our findings, we combined previous studies that have estimated POC attenuation coefficients in the North Pacific and North Atlantic gyres (Table S4, Fig. S12). In much of the world's ocean, temperature variations play a leading role in controlling the spatial near-surface density gradients, while salinity plays a secondary role[4]. Sea-surface temperature gradients derived from satellites at a 4-km resolution have been used to study fronts and filaments at scales of O(1 to 10) km[5,6]. A strong correlation between the b value and GM was found (b_{GM}=-3.74·GM+1.43; R^2=0.52, p<0.01, Fig. 5). This statistical relationship implies that a strong front is linked with high POC transfer efficiency to the mesopelagic layer, which could be used to estimate the impact of fronts on carbon export in subtropical gyres.

At the global scale, water temperature has been proposed as the key factor controlling the variation in b from low to high latitudes through its influence on microbial remineralization[7,8]. According to Marsay et al.[7], mesopelagic POC flux attenuation is correlated with water column temperature (b_T=0.062·T+0.303, where T is the median temperature of the upper 500 m). For the NPSG, there is substantial submesoscale variability in b_{GM} compared to b_T or constant b values in the gyre, and the b_{GM} generally deceased from low to high latitudes in a way that was similar to b_T (Fig. S16a and c).

The mesopelagic POC transfer efficiency (T_{eff}), which is the POC flux at 1000 m relative to the flux at the base of the euphotic zone, was estimated using b_{GM} and b_T (Text S6). Compared to the T_{eff} estimated from b_T ($T_{eff}(b_T)$), the spatial variability in T_{eff} estimated from b_{GM} ($T_{eff}(b_{GM})$) was large (Fig. S16b and d), which reflected the influence of frontal dynamics on enhancing local carbon transfer efficiency[9]. Overall, b_{GM} was smaller than b_T in the NPSG (on average 1.16±0.04 versus 1.64±0.02) during 2003-2019 (Fig. S16e). The mean b_{GM} value was more consistent with the mean observational b of 1.19 (Table S4). Both b_{GM} and b_T were lower in winter than in summer, which is in agreement with the seasonal b value in the NPSG estimated using POC flux profiles recorded at ALOHA (b=0.81±0.19 in winter versus b=1.13±0.39 in

summer; Fig. S17). The lower *b* value in winter than in summer is likely attributable to stronger submesoscale activities or lower remineralization rates in winter than in summer[7,10]. As a result, the T_{eff} in winter was higher than that in summer, as estimated from both b_{GM} and b_T. Due to the lower value of b_{GM}, the $T_{eff}(b_{GM})$ at 1000 m was much higher than the $T_{eff}(b_T)$ (on average 12.4±1.2% versus 4.7±0.1%) during 2003-2019 (Fig. S16f). The elevated $T_{eff}(b_{GM})$ is consistent with the BGC-Argo observations in this study and the observations of Guidi et al.[11]. For the entire NPSG, estimations of POC export flux at 1000 m by using b_T or a constant *b* (1.19) may underestimate the flux estimated with b_{GM} by ~58% and ~20% on average, respectively.

Text S5. Calibrating the BGC-Argo derived data

The BGC-Argo float was equipped with an SBE41CP CTD for measuring temperature, salinity, and depth; an ECO Triplet optical sensor for measuring chlorophyll-a concentration (Chl; units: mg m^{-3}) and the particulate backscattering coefficient at 700 nm ($b_{bp}(700)$; units: m^{-1}); an Aanderaa optode sensor (model 4330) for measuring dissolved oxygen concentrations (O_2; μmol kg^{-1}); and a Satlantic OCR Radiometer for measuring the photosynthetically available radiation (PAR; units: μmol quanta m^{-2} s^{-1}).

When the float was deployed, seawater samples were collected at 10 locations, 6 of which (marked as black dots in Fig. 1a; Table S5) were used for nitrate determination, and the others (marked as yellow pentagrams in Fig. 1a) were used for POC determination. Nitrate from discrete samples were analyzed onboard with a Four-channel Continuous Flow Technicon AA3 Auto-Analyzer (Bran-Lube, GmbH) at depths of 0-200 m with an interval of 10 m and 200-500 m with an interval of 100 m. The POC concentrations were determined by analyzing 8 L of seawater collected at intervals of 30 m between depths ranging from 30 to 180 m, as well as at a depth of 200 m using an Elemental vario EL cube CHNS Analyzer[12]. The uncertainty for POC data is < 10%.

The shipboard nitrate concentration was used to calibrate the nitrate estimations derived from BGC-Argo float data. By matching each shipboard sampling station with the nearest float sampling location from the BGC-Argo trajectories, the vertical distributions of nitrate were compared between the shipboard and BGC-Argo measurements (Fig. S13a and b). The nitrate concentrations in the upper layer were below the limit of quantification (BLQ), which is 0.1 μmol L^{-1}. From 100 m to 300 m depth, the ship-based nitrate concentration increased from 0.1 μmol L^{-1} to ~9 μmol L^{-1}. The measurements from BGC-Argo floats captured the vertical structure of nitrate distributions in the NPSG well, despite a slight mismatch of the values below 300 m for both floats (Fig. S13a and b). The X-Y scatter plots for both BGC-Argo floats demonstrated a high correlation between the shipboard and BGC-Argo derived nitrate

concentrations (R^2=0.98 for float 2902753 and R^2=0.96 for float 2902756; Fig. S13c and d). The linear regression relationships for both BGC-Argo floats (Y=0.92·X+0.01 for float 2902753 and Y=1.13·X-1.1 for float 2902756) suggested an overestimation for float 2902753 and an underestimation for float 2902756, which were corrected for all the float profiles.

The POC concentration was estimated by converting the BGC-Argo derived $b_{bp}(700)$ into the POC concentration based on empirical equations[13–16]. In subtropical gyres, the relationship between POC and $b_{bp}(700)$ is usually described as follows[16]:

$$\text{POC (mmol C m}^{-3}\text{)}=\frac{1}{12}\cdot\left(53607\cdot b_{bp}\cdot(700/555)+2.5\right) \quad \text{(S1)}$$

This relationship was shown to overestimate the POC in our study region compared to in situ measurements (Fig. S14). We thus derived another relationship between POC and $b_{bp}(700)$ by applying a linear fitting method to the in situ measured POC and the BGC-Argo derived $b_{bp}(700)$:

$$\text{POC (mmol C m}^{-3}\text{)}=2083.17\cdot b_{bp}+0.06 \quad \text{(S2)}$$

Eq. (S2) was shown to better estimate the POC distribution than Eq. (S1) in the NPSG, as the mean square error (MSE) was much smaller than that from Eq. (S2).

Text S6. Estimating the transfer efficiency of POC flux in the NPSG

The relationships between the *b* value and the SST gradient (b_{GM}) and the temperature (b_T) were applied to the NPSG to estimate the POC transfer efficiency. The relationship between b_{GM} and GM was shown in Fig. 5:

$$b_{GM}=-3.74\cdot GM+1.43 \quad (S3)$$

The b_{GM} accounts for the influence of front on POC transfer efficiency. On the other hand, Marsay et al.[7] suggested that the attenuation coefficient can be estimated based on the relationship between the *b* value and upper water column temperature:

$$b_T=0.062\cdot T+0.303 \quad (S4)$$

where T is the median temperature of the upper 500 m.

The daily SST values were obtained from the CMEMS at a spatial resolution of ~4.5 km to calculate the spatial distributions of GM following the method provided by Belkin and O'Reilly[17]. The b_{GM} from 2003 to 2019 was then obtained using Eq. (S3). The b_T in the NPSG was obtained using the upper water column (500 m) median temperature provided by the World Ocean Atlas climatological data.

The transfer efficiency of POC flux to the mesopelagic layer was estimated from b_{GM} ($T_{\text{eff}}(b_{GM})$) and from b_T ($T_{\text{eff}}(b_T)$) as

$$T_{\text{eff}}(b_{GM}) = \left(\frac{1000\,m}{Z_{\text{Eu}}}\right)^{-b_{GM}} \cdot 100 \quad (S5)$$

$$T_{\text{eff}}(b_T) = \left(\frac{1000\,m}{Z_{\text{Eu}}}\right)^{-b_T} \cdot 100 \quad (S6)$$

Table S1. The operation details of sampling when BGC-Argo encountered mesoscale eddies

BGC-Argo	**2902753**	**2902756**
Eddy encountered	CE 1	CE 2
First date	2019/04/03	2019/03/28
Last date	2019/05/03	2019/04/26
Time interval (day)	0.5±0.0	0.5±0.0
Distance (km)	6.45±2.03	6.2±1.3
Propagation speed (m/s)	0.15±0.05	0.14±0.03
Sampling depth(m)	971.4±16.0	979.6±14.3
Vertical resolution (m)	2	2

Table S2. Eddy characteristics

	CE 1	CE 2
First detection	2019/02/10	2019/03/24
Last detection	2019/06/27	2019/04/22
Total duration [days]	138	30
Age at time of BGC-Argo deployment [days]	48	3
	On average during the period sampled by the floats	
Amplitude [m]	0.14±0.03	0.09±0.04
Radius [m]	108.5±20.9	89.7±22.1
Propagation speed [m s^{-1}]	0.11±0.05	0.10±0.05
	On average during eddy whole lifespan	
Amplitude [m]	0.15±0.08	0.09±0.04
Radius [km]	114.5±37.2	91.4±22.2
Propagation speed [m s^{-1}]	0.09±0.10	0.10±0.05

Table S3. Correlation coefficients (CR) of temperature, NO_3^-, DO, Chl and POC with r/R at both the sea surface and Z_{SCM} for CE 1 and with SLA during the intensifying stage (Intensification) and the whole observational period for CE 2 (ALL).

Argo	Eddy ID				Temperature [°C]	NO_3^- [mmol N m^{-3}]	DO [μmol kg^{-1}]	Chl [mg m^{-3}]	POC [mmol C m^{-3}]
2902753	CE 1	r/R(<2)	surface	CR	0.89	-0.57	-0.91	0.09	-0.35
				p value	<0.001	<0.001	<0.001	0.56	0.02
			Z_{SCM}	CR	0.66	-0.58	0.3	-0.47	-0.74
				p value	<0.001	<0.001	0.04	<0.001	<0.001
2902756	CE 2	SLA	ALL	CR	0.69	-0.63	0.34	-0.19	0.03
				P value	<0.001	<0.001	0.01	0.13	0.84
			Intensification	CR	0.98	-0.91	0.65	-0.90	-0.87
				p value	<0.001	<0.001	<0.001	<0.001	<0.001

Table S4. Compilation of POC export fluxes in the subtropical North Pacific and North Atlantic gyres

Method	Region	Date	Z_{Eu} [m]	$F(Z_{Eu})$ [mmolC m^{-2}d^{-1}]	F(1000 m) [a] [mmolC m^{-2}d^{-1}]	T_{eff} $\frac{F(1000m)}{F(Z_{Eu})}$	Martin b	Site Name	reference
^{234}Th	47°N 160°E	31-Jul-05	50	11.08	2.01	0.18	0.57	K2-1	Marsay et al.[7]
^{234}Th	47°N 160°E	9-Aug-05	50	3.25	0.75	0.23	0.49	K2-2	Marsay et al.[7]
	Average		50	7.17	1.38	0.21	0.53		
^{234}Th	22°N 158°W	Jun/Jul 2004	125	1.25	0.11	0.08	1.17	ALOHA	Buesseler and Boyd[18]
Sediment Trap	22°N 158°W	1-Sep-13	175	1.91	0.40	0.21	0.89	ALOHA	Grabowski et al.[19]
Sediment Trap	22°N 158°W	26-Jun-04	125	1.5	0.10	0.07	1.29	ALOHA-1	Wiedmann et al.[20]
Sediment Trap	22°N 158°W	4-Jul-04	125	1.3	0.07	0.05	1.42	ALOHA-2	Wiedmann et al.[20]
Sediment Trap	24.11° N 157.74° W	6-Aug-08	150	2.2	0.45		0.84	ALOHA-F	Guidi et al.[11]
	12°N 140°W	5-Feb-92					1.53	TT07-N	Wiedmann et al.[20]

	Average		140	1.63	0.23	0.10	1.19		
BGC-Argo	NPSG (eddy)	Apr-19	149.0 ±7.5	1.68	0.19	0.12	1.14	NPSG-E	This study
BGC-Argo	NPSG (front)	Apr-19	169.1 ±2.9	2.11	0.78	0.37	0.56	NPSG-F	This study
trap	48.92° N 16.39° W	3-Aug-09					0.77	PAP	Wiedmann et al.[20]
trap	26.5°N 31°W	25-Aug-11					1.99	NASG-D369	Wiedmann et al.[20]
^{234}Th	30.9°N 66.7°W	10-Jul-05	140	1.17	0.15	0.13	1.03	EDDIES-III	Buesseler et al.[21]
^{234}Th	30.18° N 68.67° W	19-Aug-05	140	2.25	1.40	0.62	0.24	EDDIES-IV	Buesseler et al.[21]
	Average		140	1.71	0.78	0.38	1.01		

[a] $F_{(1000\ m)}$ is derived from the fitted Martin curve.

Table S5. In situ sampling locations and dates

	BGC-Argo	Station	date	Latitude	Longitude	Note
POC	2902756	S23	2019/03/24	20.42	137.55	Sampling depths in 30:30:180 200 m
	2902753	Z2	2019/04/03	18.10	128.30	
	2902753	A36	2019/04/11	18.10	127.30	
	2902753	5A35	2019/04/14	18.10	127.70	
Nitrate	2902756	S23	2019/03/24	20.42	137.55	Sampling depths in 0:10:200 300 400 500 m
	2902756	S24	2019/03/24	20.42	137.93	
	2902753	2A34	2019/04/03	18.10	128.60	
	2902753	Z3	2019/04/04	18.10	128.78	
	2902753	A36	2019/04/11	18.10	127.30	
	2902753	5A35	2019/04/14	18.10	127.70	

Table S6. Linear least squared estimations for the rate of change of integrated POC (unit: mmol C m^{-2} d^{-1})

	Depth	Intensifying				Mature (Decaying)			
		Rate	Standard deviation	R^2	*p*	Rate	Standard deviation	R^2	*p*
Eddy	Z_{Eu}	-0.82	0.28	0.50	<0.001	-2.50	0.13	0.94	<0.001
	200 m	-1.06	0.16	0.80	<0.001	-2.13	0.11	0.94	<0.001
	300 m	-0.84	0.20	0.61	<0.001	-1.86	0.09	0.95	<0.001
	400 m	-0.91	0.19	0.68	<0.001	-1.43	0.07	0.94	<0.001
	500 m	-0.71	0.19	0.52	<0.001	-0.98	0.06	0.91	<0.001
Front	Z_{Eu}	0.71	0.33	0.33	0.04	-1.40	0.18	0.95	<0.001
	200 m	0.71	0.29	0.39	0.02	-1.59	0.12	0.97	<0.001
	300 m	-0.12	0.23	0.03	0.58	-1.52	0.17	0.95	<0.001
	400 m	0.30	0.15	0.31	0.05	-1.18	0.13	0.94	<0.001
	500 m	0.17	0.18	0.10	0.30	-0.82	0.17	0.90	<0.001

Table S7. Linear least squared estimations for the rate of change of AOU (unit: mmol O_2 m^{-3} d^{-1})

Depth	Eddy		Front	
	Rate	Standard deviation	Rate	Standard deviation
Z_{Eu}	0.72	0.15	-0.14	0.44
200 m	0.79	0.16	-0.14	0.45
300 m	0.28	0.18	-0.09	0.54
400 m	0.18	0.19	0.07	0.61
500 m	0.17	0.20	0.01	0.62
600 m	0.51	0.19	0.10	0.35
700 m	0.49	0.13	0.17	0.10
800 m	0.21	0.07	-0.23	0.11

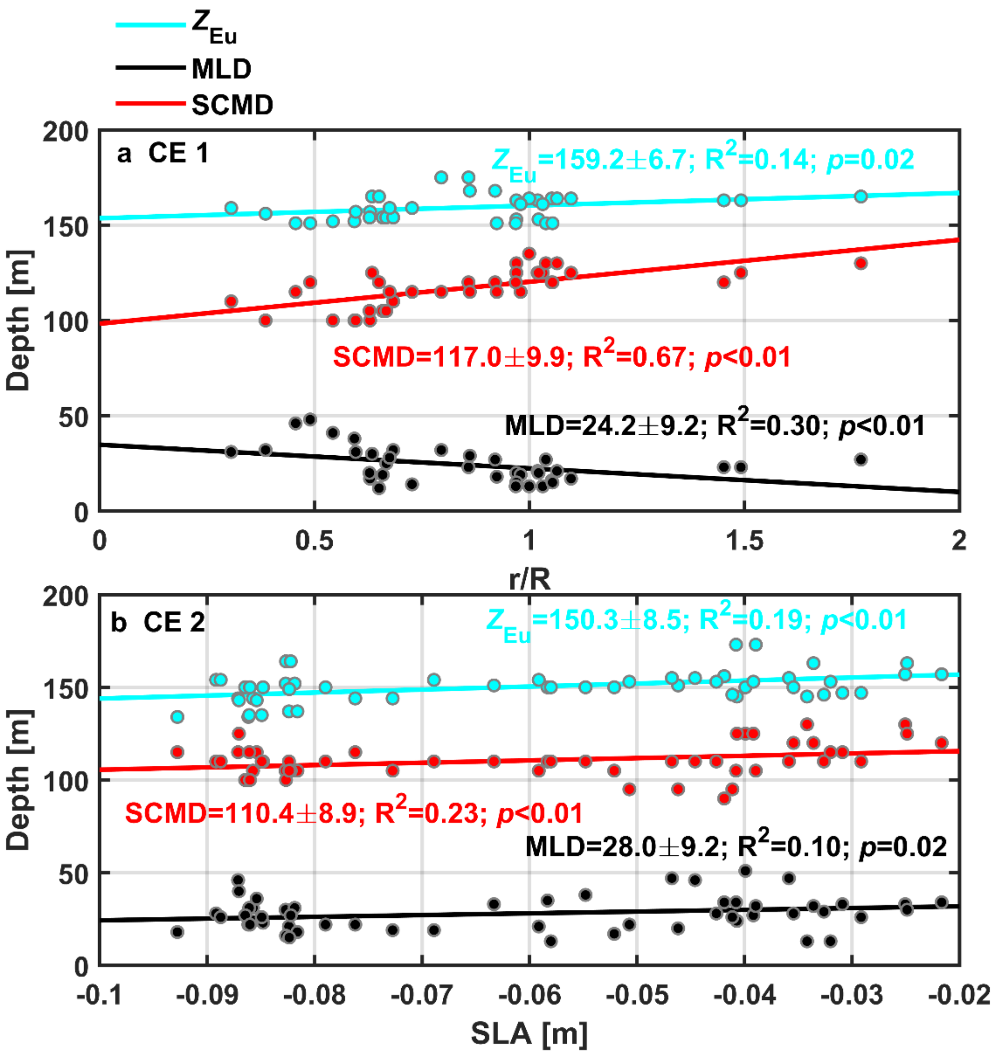

Figure S1. Scatterplots of Z_{Eu}, MLD and Z_{SCM} versus r/R in **a** CE 1 and versus SLA in **b** CE 2. Source data are provided as a Source Data file.

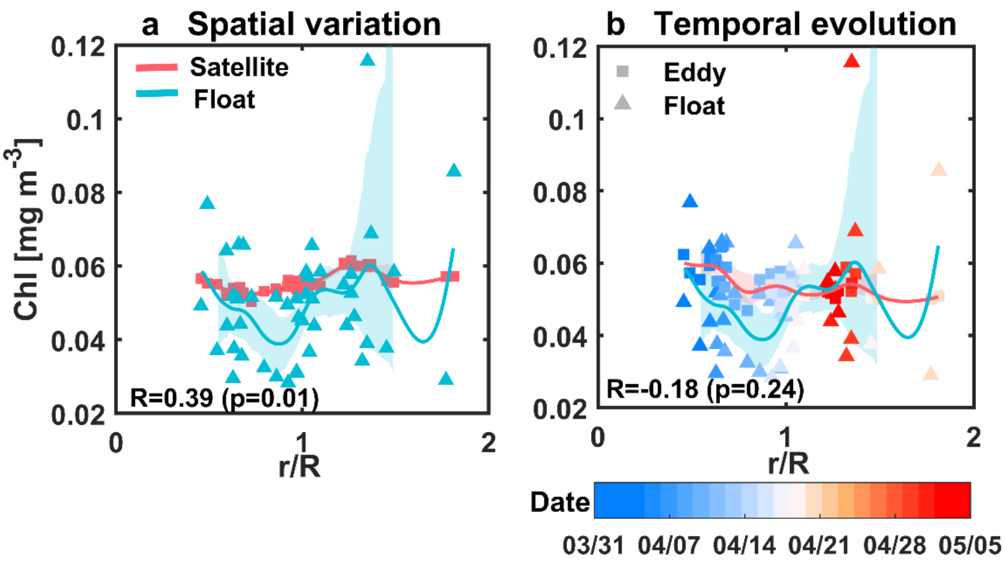

Figure S2. Comparison of sea surface Chl between satellite and BGC-Argo observations in both spatial and temporal variability. **a** Radial distribution of sea surface Chl at the float locations derived from both the BGC-Argo and satellite observations. The BGC-Argo results were directly obtained from the surface Chl measured by the float. The satellite-derived Chl values were calculated by averaging the Chl from OC-CCI over the float sampling period and then interpolating to the relative locations of the float in CE 1. Because the eddy propagated over time, the location of the float in the eddy was constructed by calculating the azimuthal and radial positions of the float relative to the eddy center. **b** Satellite-derived Chl averaged inside CE 1 and sea surface Chl derived from BGC-Argo observations. The BGC-Argo results were directly obtained from the surface Chl measured by the float. The satellite-derived Chl values were calculated by averaging the satellite-derived Chl values inside the eddy at the time when the float was at the position of r/R. The color in **b** indicates date.

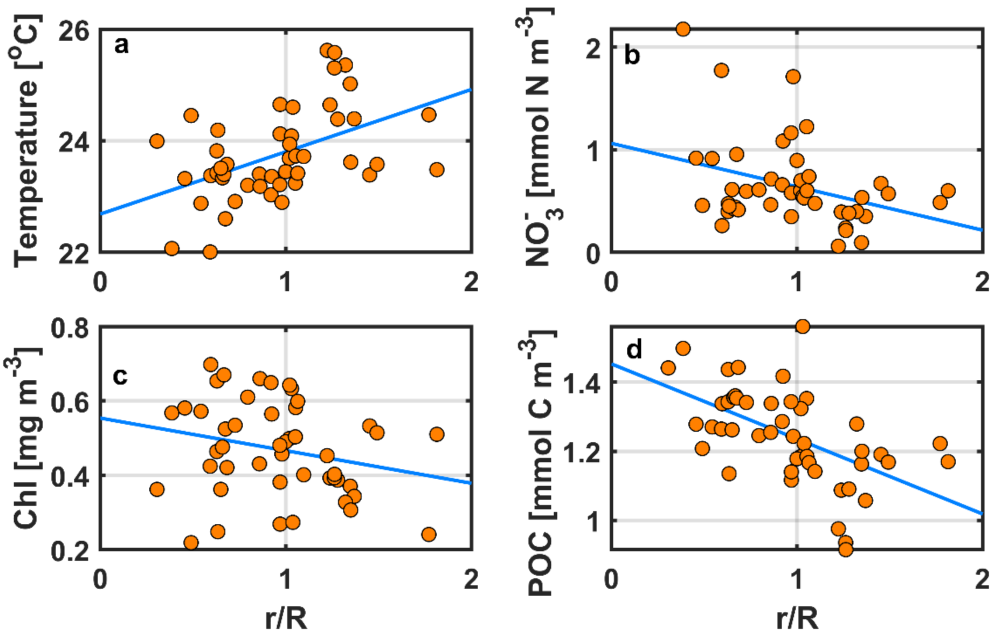

Figure S3. Radial distributions of **a** temperature, **b** NO_3^-, **c** Chl, and **d** POC in CE 1 at the depth of Z_{SCM}. The solid lines indicate the linear regression lines. Source data are provided as a Source Data file.

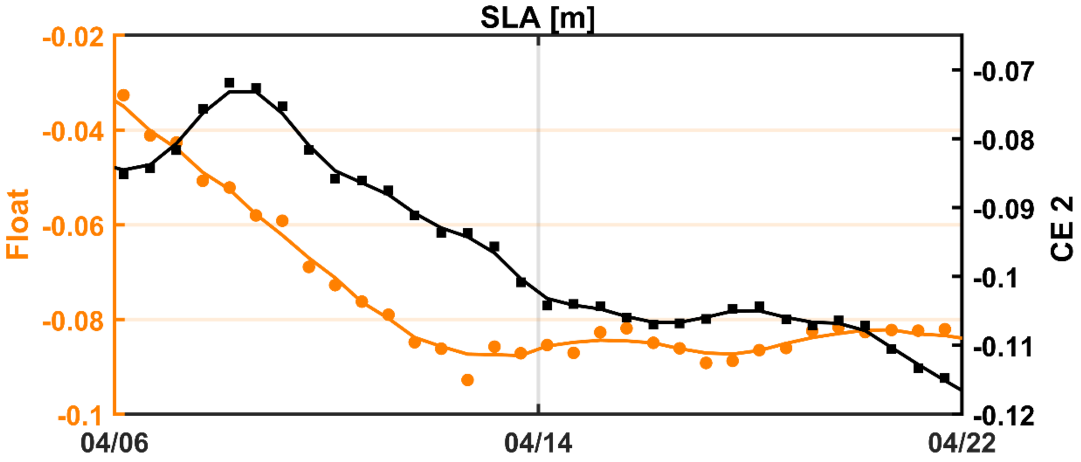

Figure S4. Time series of sea level anomaly (SLA) along (orange) the float trajectory and (black) averaged inside CE 2. Source data are provided as a Source Data file.

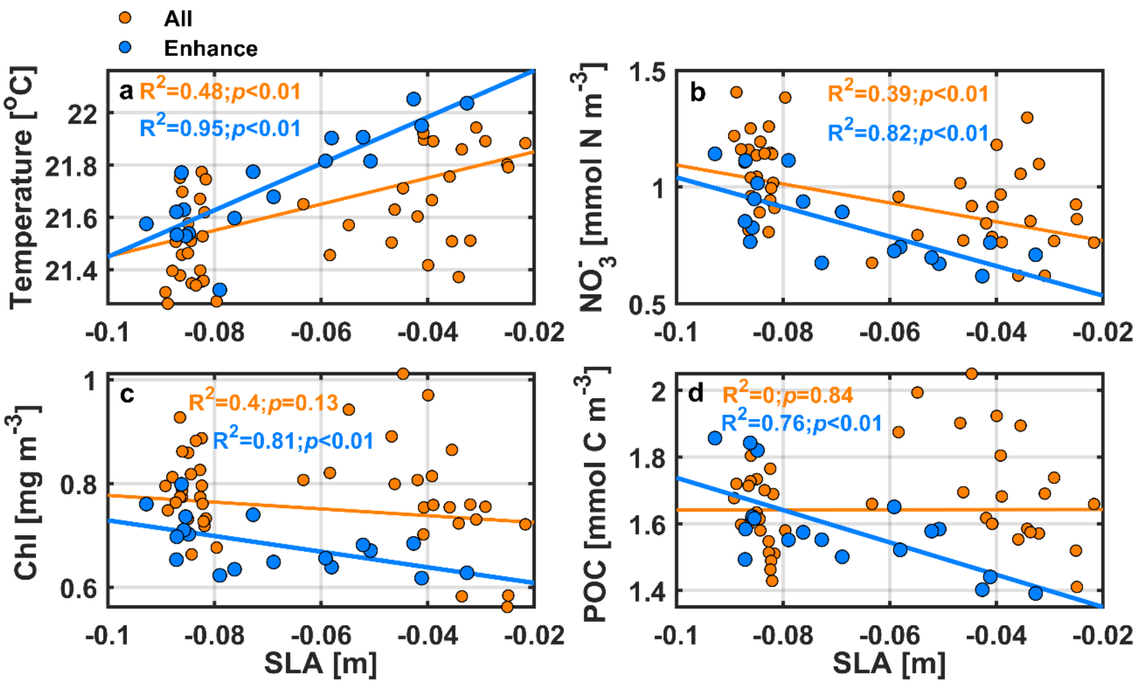

Figure S5. Scatterplots of **a** temperature, **b** NO_3^-, **c** Chl, and **d** POC at the depth of Z_{SCM} versus SLA during (blue) the eddy intensification stage and during (orange) the other observational period in CE 2. The solid lines represent the linear regression lines. Source data are provided as a Source Data file.

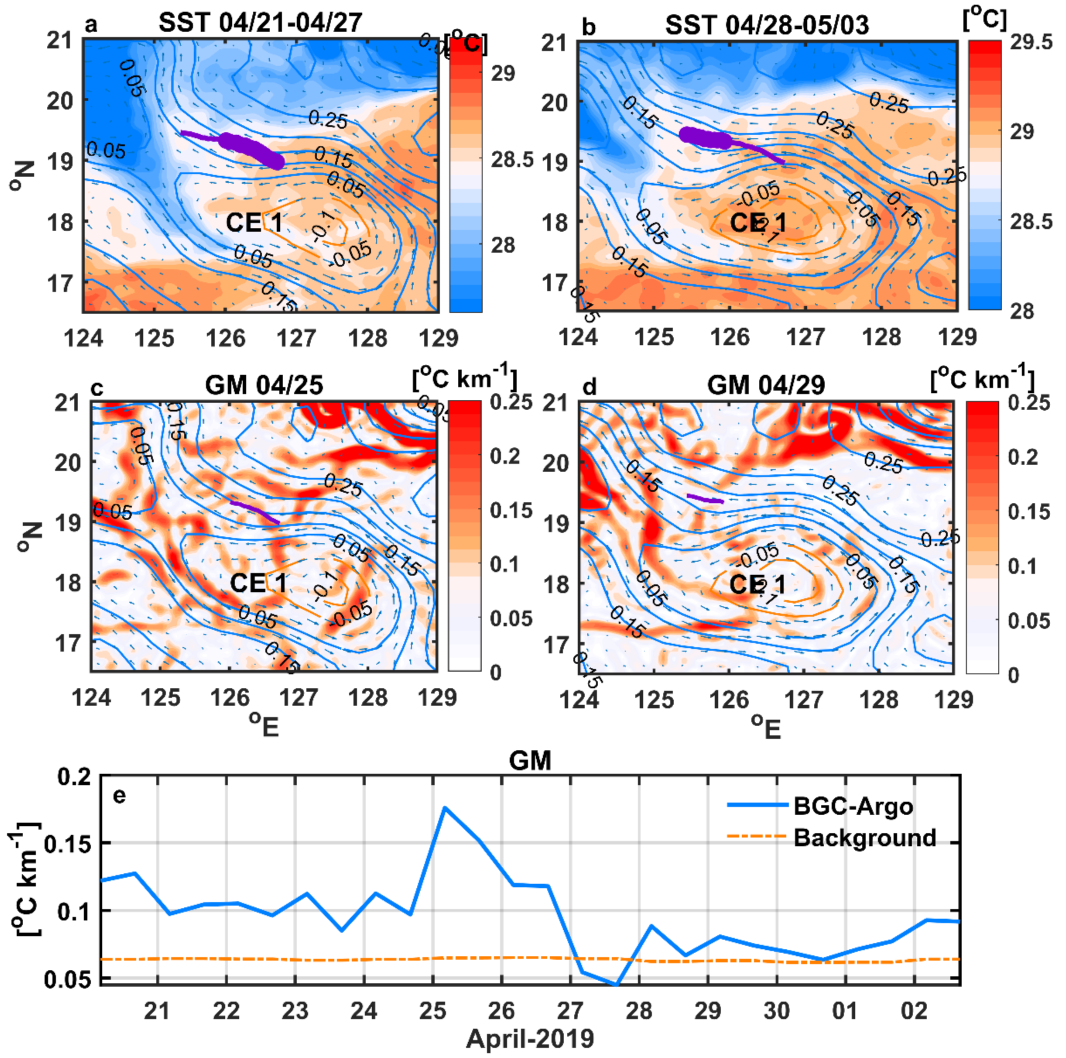

Figure S6. The SST gradient magnitude (GM), lateral buoyancy frequency (M^2) and normalized strain rate (S/f) at the front. **a** Weekly averaged sea-surface temperature (background color) and the locations of the BGC-Argo floats in the eddy during 21-27 April 2019 (purple points). The purple line is the float trajectory from 21 April to 3 May 2019. **b** The same as **a** but during 28 April-3 May 2019. **c** The GM distribution on 25 April 2019. The purple line is the float trajectory during 21-27 April 2019. **d** The same as **c** but on 29 April 2019. The purple line is the float trajectory during 28 April-3 May 2019. **e** The GM at the BGC-Argo float location during 21 April-3 May 2019.

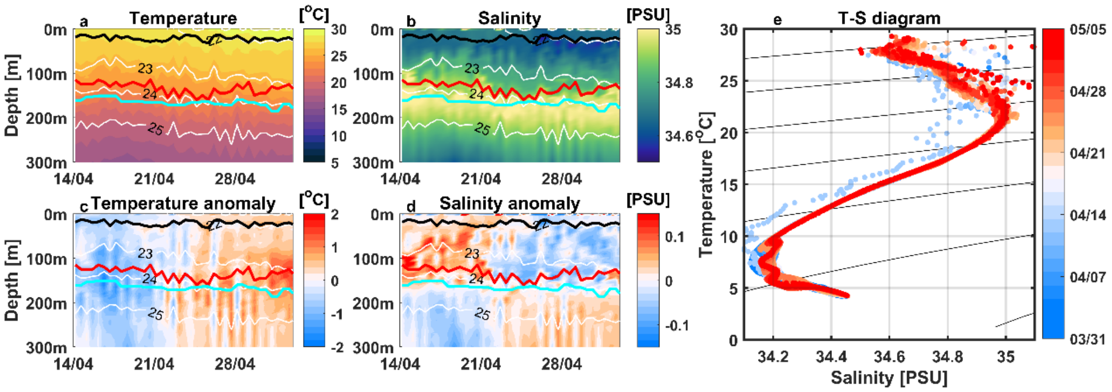

Figure S7. Physical characteristics of the float measurements. During 21 April-3 May 2019, the float was on one side of the front. Time series of **a** temperature, **b** salinity, **c** temperature anomaly and **d** salinity anomaly measured by float 2902753 from 1 April to 3 May 2019. The black, red and cyan lines indicate the mixed layer depth (MLD), the depth of subsurface chlorophyll maximum (Z_{SCM}) and the euphotic zone (Z_{Eu}), respectively. The white lines are the isopycnals of 23, 24 and 25 kg m^{-3}, respectively. The temperature and salinity anomalies were defined as the instantaneous values subtracting the full time mean. **e** Temperature-salinity diagram with data points from float 2902753 from 1 April to 3 May 2019. The color indicates the sampling date.

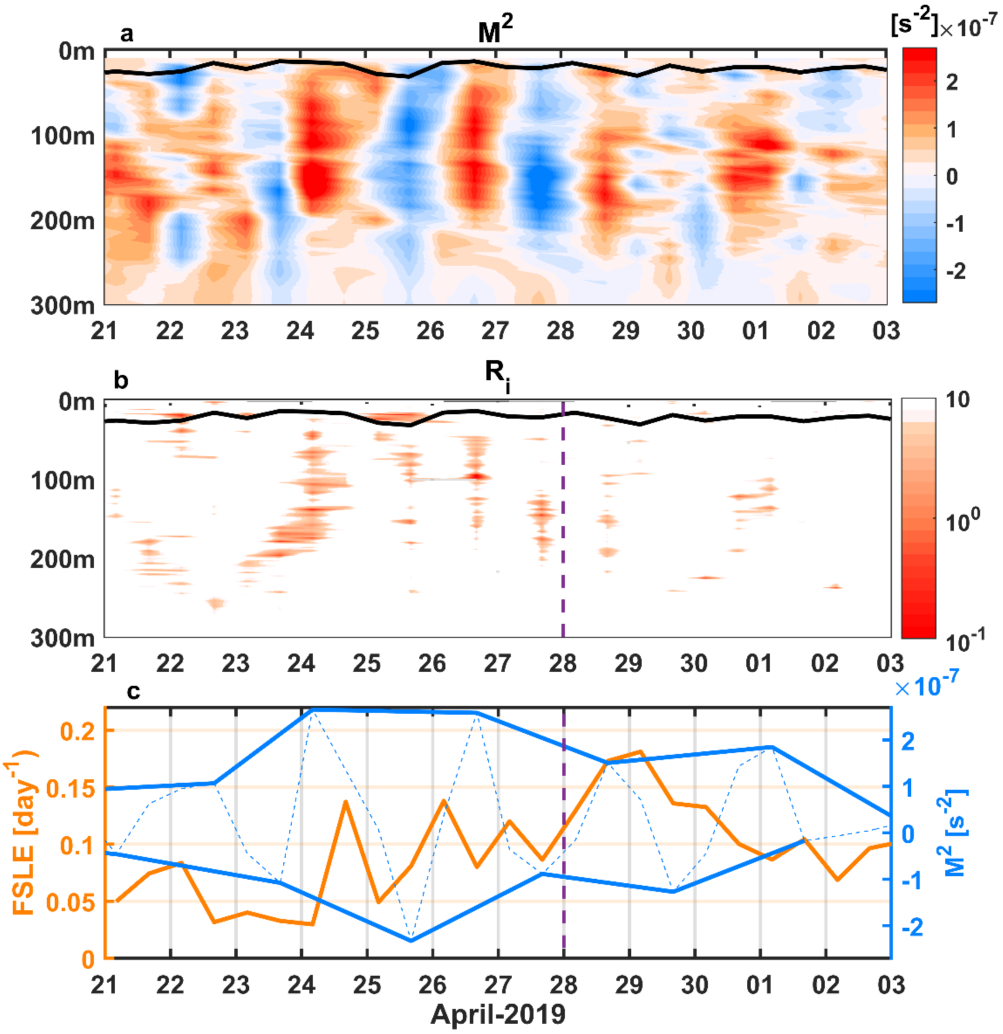

Figure S8. The lateral buoyancy frequency (M^2), Richardson number (R_i) and finite-size Lyapunov exponent (FSLE) at the front. **a** BGC-Argo-derived lateral buoyancy frequency during 21 April-3 May 2019. $M^2 = -\partial b/\partial s$, where s is the along-track direction. The black line represents the mixed layer depth. **b** BGC-Argo-derived Richardson number during 21 April-3 May 2019. $R_i = f^2 b_z / b_s^2$, where f and b are the Coriolis parameter and buoyancy, respectively. The black line represents the mixed layer depth. **c** Surface FSLE in the frontal region (orange line) and the lateral buoyancy frequency (blue dashed line) at 100 m during 21 April-3 May 2019. Blue solid lines are the buoyancy frequency envelope. Purple dash lines indicate the shift from the intensifying to decaying stage.

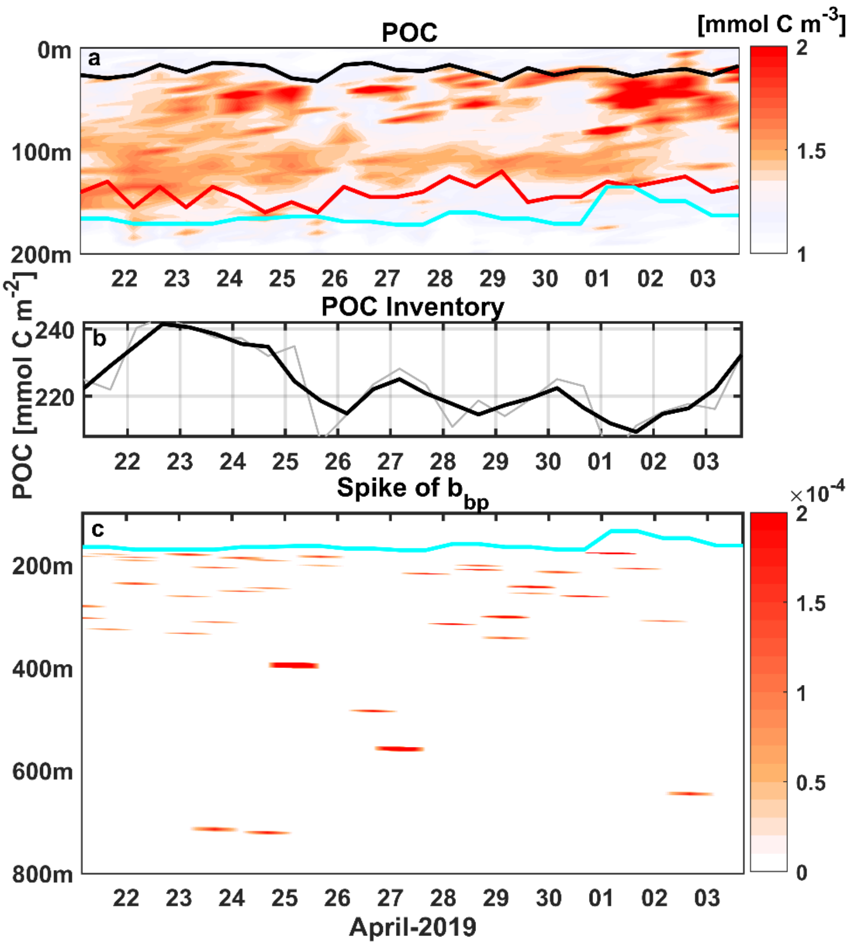

Figure S9. The POC and spike of $b_{bp}(700)$ at the front. **a** BGC-Argo derived POC distribution during 21 April-3 May 2019. The black, red and cyan lines are the MLD, Z_{SCM} and Z_{Eu}, respectively. **b** Depth-integrated POC in the euphotic zone during 21 April-3 May 2019. Gray and black lines in **b** represent the time series of the integrated POC without and with a 5-point moving filter, respectively. **c** Spikes of $b_{bp}(700)$ during 21 April-3 May 2019. The cyan line indicates Z_{Eu}.

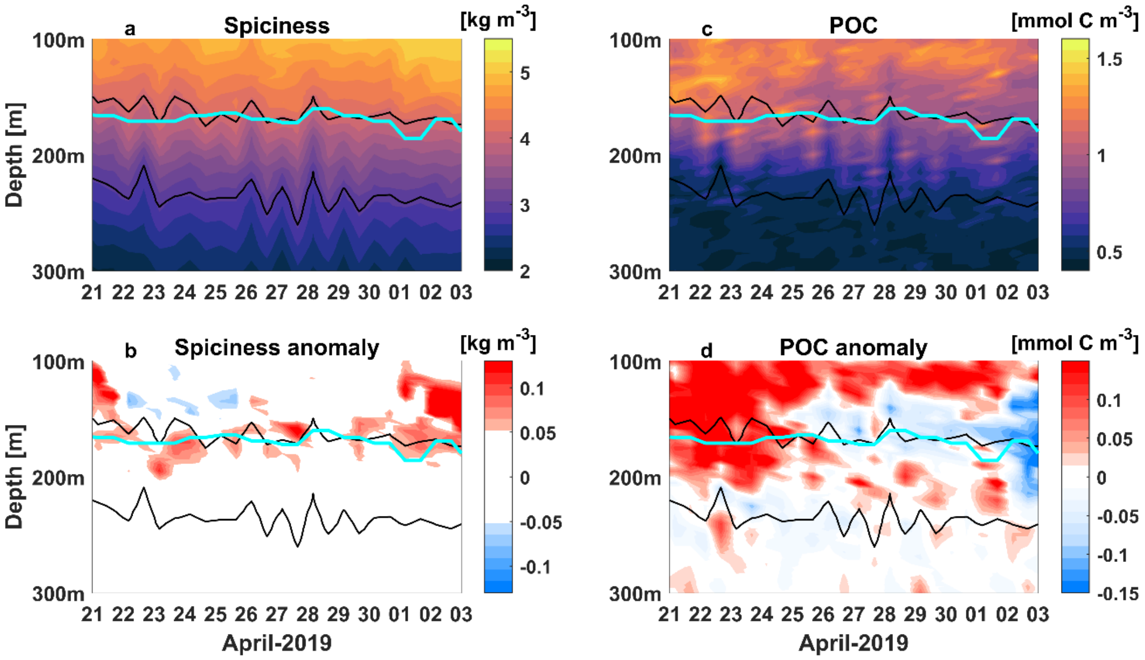

Figure S10. Vertical distribution of spiciness and POC. Time series of **a** spiciness and **c** POC distributions measured by float 2902753 from 21 April to 3 May 2019. Time series of **b** spiciness anomaly and **d** POC anomaly measured by float 2902753 from 21 April to 3 May 2019. The cyan line indicates the euphotic zone depth. The black lines are the isopycnals of 24 and 25 kg m^{-3}. The spiciness and POC anomalies were defined as the instantaneous values subtracting the annual mean at the same isopycnal layer.

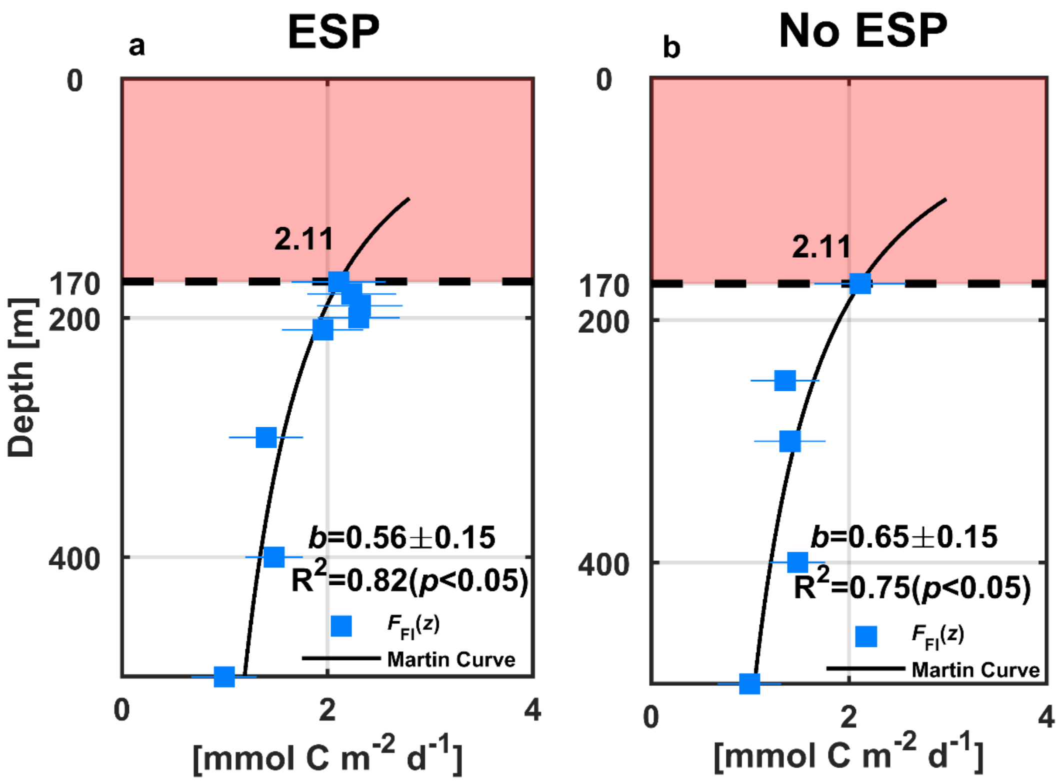

Figure S11. POC flux profiles. **a** The POC flux profile with the subduction pump (ESP) between Z_{Eu} and 250 m accounted for when estimating the b value. **b** The POC flux profile without the ESP when estimating the b value. The black dashed lines and error bars correspond to the euphotic zone depth and one standard deviation, respectively. The red areas represent the euphotic zone. Source data are provided as a Source Data file.

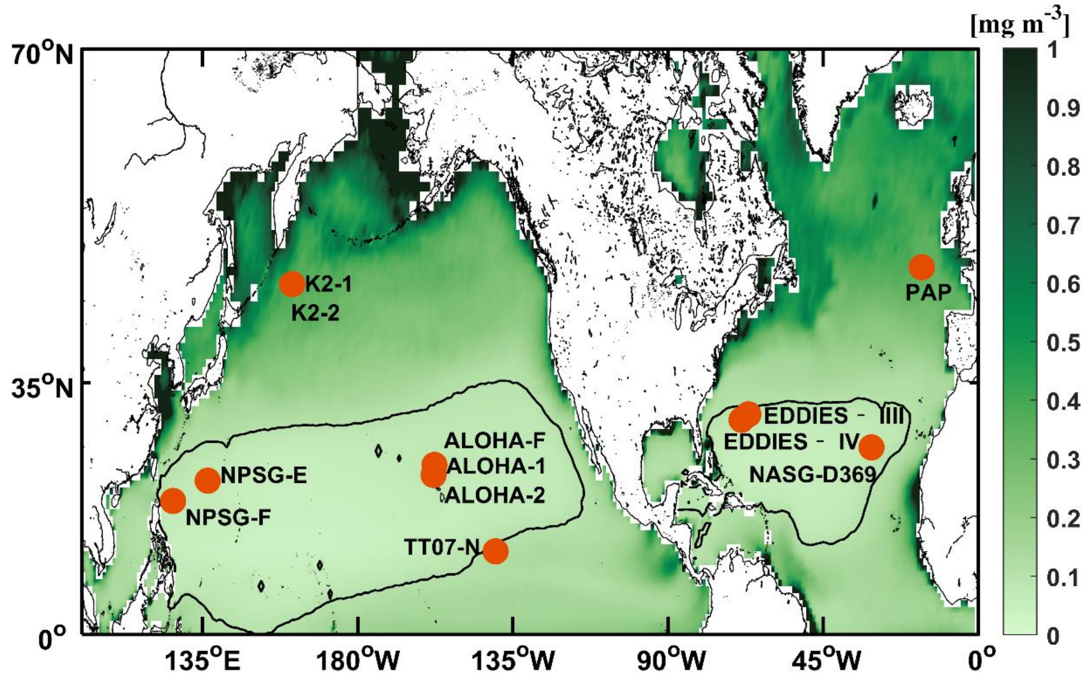

Figure S12. Locations of the POC flux data from previous studies (Fig. 5, Table S4) in subtropical gyres. The background color is the mean chlorophyll concentration from satellite data, and the black contour is the chlorophyll concentration of 0.1 mg m^{-3}.

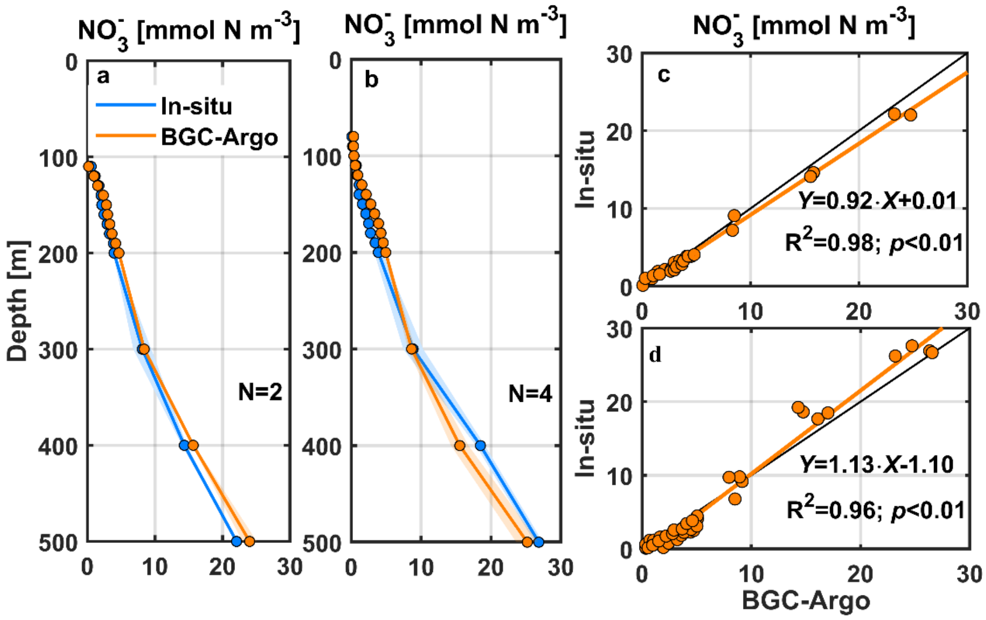

Figure S13. Calibration of the BGC-Argo derived nitrate concentrations. **a-b** Vertical profiles and **c-d** scatterplot showing the comparison between the in situ and BGC-Argo derived nitrate concentrations. **a** and **c** are results for float 2902753, and **b** and **d** are results for float 2902756. Solid lines represent average results, and shades represent their standard deviations. The number of in situ sampling stations N is indicated in **a** and **b**. The orange lines in **c** and **d** are the linear regression lines.

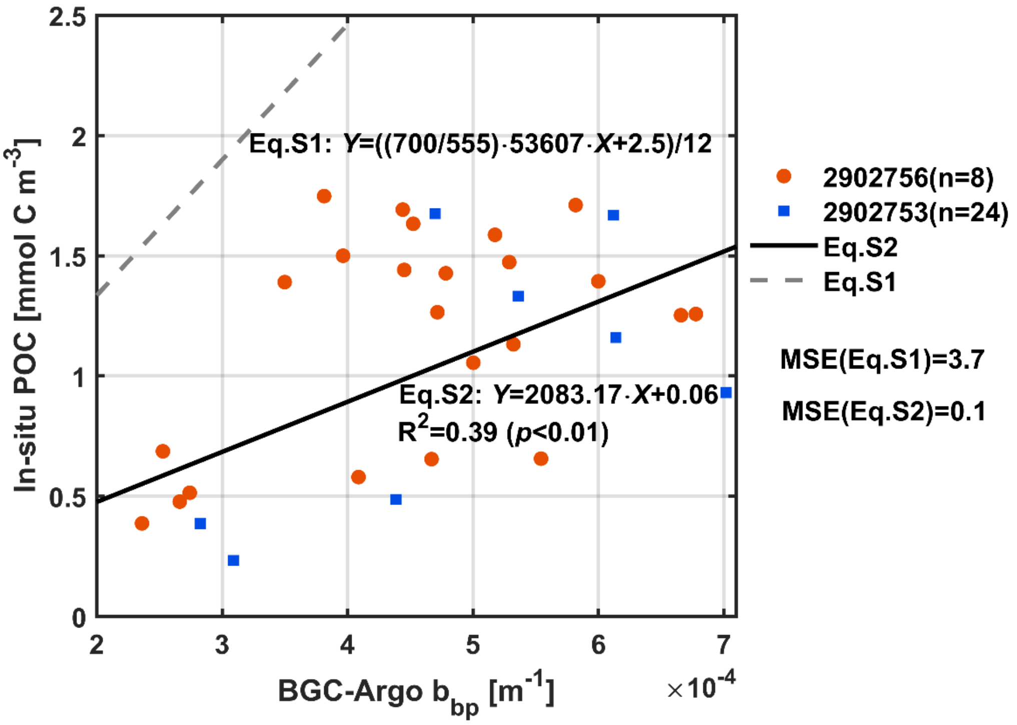

Figure S14. Relationship between BGC-Argo derived $b_{bp}(700)$ and in situ measured POC concentrations. Scatterplot of BGC-Argo derived b_{bp} versus in situ POC. Eq. (S1) (gray dashed line) was from Stramski et al.[16], and Eq. (S2) (black solid line) was derived from the linear regression of BGC-Argo derived b_{bp} versus in situ measured POC. Orange and blue colors indicate data points from floats 2902753 and 2902756, respectively. The mean square error (MSE) for Eq. (S1) and Eq. (S2) are 3.7 and 0.1 $mmol^2$ C m^{-6}, respectively.

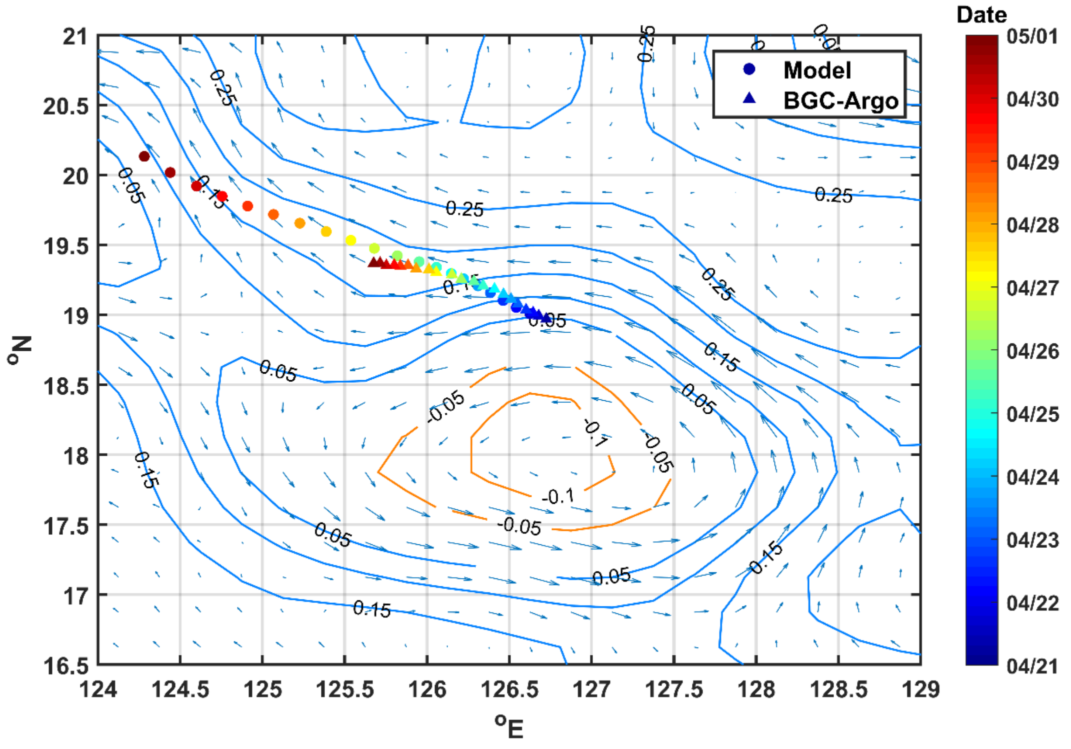

Figure S15. Trajectories of the (triangle) BGC-Argo and (dots) the virtual particle over the period when the float was at the front (21 April-3 May 2019). The particle was released at the same position as the float on 21 April 2019 and transported by the surface current velocity derived from AVISO. The particle trajectory was tracked using the TRACMASS code. Color indicates the date. Blue and orange lines are the contour lines of the sea level anomaly (SLA). Arrows represent the sea surface current velocity anomaly. Source data are provided as a Source Data file.

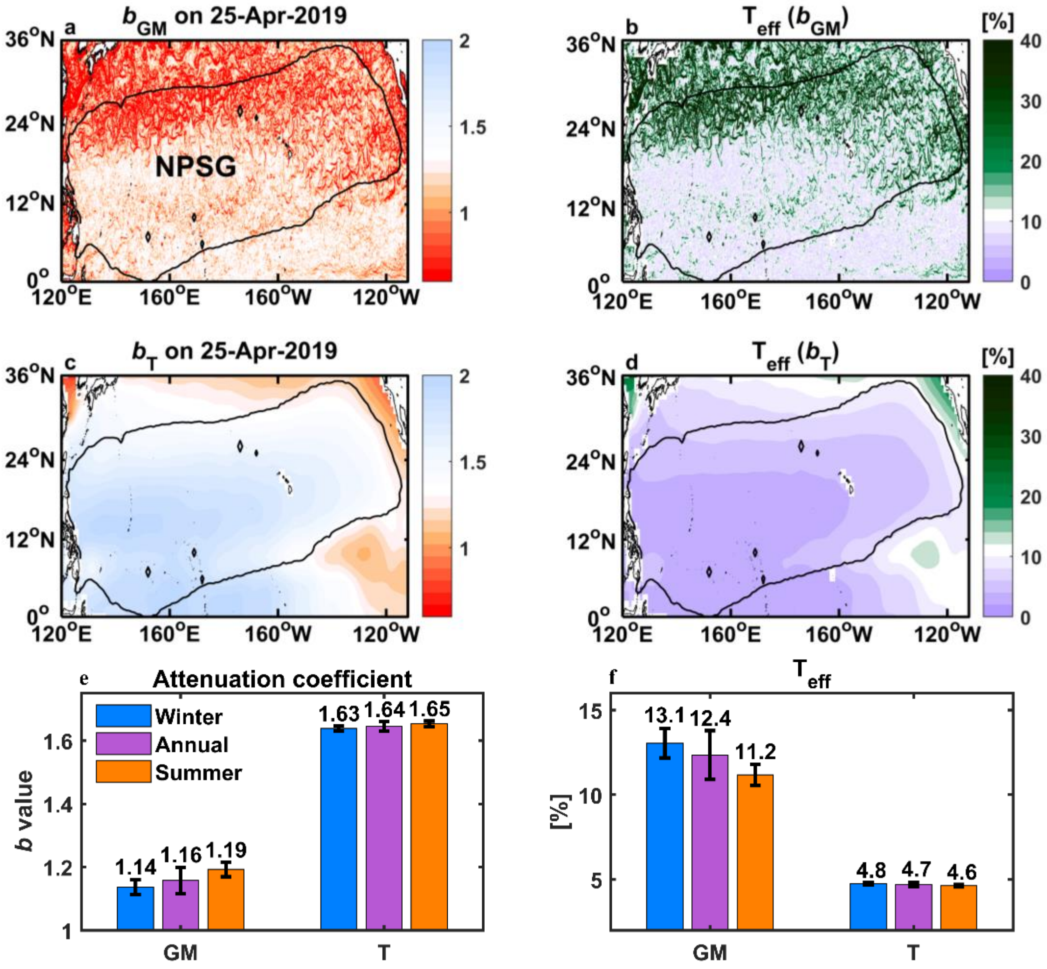

Figure S16. Transfer efficiency of POC flux at 1000 m estimated based on attenuation coefficient *b*. **a-b** Spatial distributions of attenuation coefficient *b* derived from GM (b_{GM}) on 25 April 2019 (**a**) and its transfer efficiency of POC flux at 1000 m ($T_{eff}(b_{GM})$) (**b**). **c-d** Spatial distributions of the attenuation coefficient *b* derived from the median temperature of the upper 500 m (b_T) on 25 April 2019 (**c**) and its transfer efficiency of POC flux at 1000 m ($T_{eff}(b_T)$) (**d**). **e-f** Bar plots quantifying the *b* value (**e**) and the transfer efficiency of the POC flux T_{eff} at 1000 m (**f**) during 2003-2019 in the NPSG. The black contour in **a-d** is the Chl of 0.1 mg m^{-3}, which represents the outline of the NPSG. Bars are plotted as the mean ± 1 standard deviation. Source data are provided as a Source Data file.

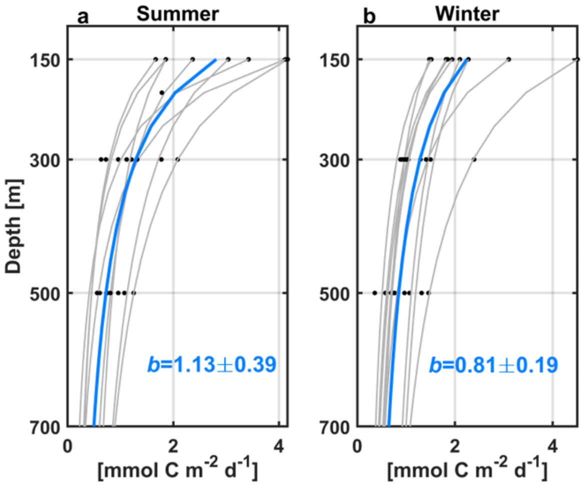

c

Summer	*b*	R^2	winter	*b*	R^2
1	1.08	0.93	1	0.93	0.98
2	1	1	2	0.93	1
3	0.83	1	3	0.94	1
4	1.63	0.99	4	0.61	0.96
5	0.66	0.95	5	0.81	1
6	1.17	0.98	6	0.91	0.93
7	0.89	0.96	7	0.53	0.99
8	1.77	0.94	8	1.11	0.98
			9	0.54	0.93
			10	0.78	1
Mean *b*	1.13±0.39			0.81±0.19	

Figure S17. The seasonal *b* value at the ALOHA station. **a** POC flux profiles and the corresponding Martin curves in summer. Black dots are observational profiles from sediment traps. Gray lines are the fitted Martin curves. The blue line is the Martin curve derived from the averaged *b* value and averaged POC flux at 150 m. **b** is the same as **a** but in winter. **c** The *b* value and R^2 for each observational POC flux profile in both summer and winter. We picked up all observational POC flux profiles with at least three vertical sampling points for Martin curve fitting. The *b* values in summer are significantly different from those in winter, as h=1 and p=0.036 in the two-sample t test result. The *b* values in **a-b** are shown as the mean ± 1 standard deviation. Source data are provided as a Source Data file.

References:

1. Pegliasco, C., Busché, C. & Faugère, Y. Mesoscale Eddy Trajectory Atlas META3.2 Delayed-Time all satellites: version META3.2 DT allsat. AVISO https://doi.org/10.24400/527896/A01-2022.005.220209 (2022).
2. Blanke, B. & Raynaud, S. Kinematics of the Pacific Equatorial Undercurrent: a Eulerian and Lagrangian approach from GCM results. *J. Phys. Oceanogr.* **27**, 1038–1053 (1997)
3. Döös, K. Inter-ocean exchange of water masses. *J. Geophys. Res. Oceans* **100**, 13499–13514 (1995)
4. Johnson, G. C., Schmidtko, S. & Lyman, J. M. Relative contributions of temperature and salinity to seasonal mixed layer density changes and horizontal density gradients. *J. Geophys. Res. Oceans* **117**, C04015 (2012).
5. Rivière, P., Jaud, T., Siegelman, L., Klein, P., Cotté, C., Le Sommer, J., Dencausse, G. & Guinet, C. Sub-mesoscale fronts modify elephant seals foraging behavior. *Limnol. Oceanogr. Lett.* **4**, 193-204 (2019)
6. Dong, J., & Zhong, Y. Submesoscale fronts observed by satellites over the northern South China Sea shelf. Dyn. *Atmospheres Oceans* **91**, 101161 (2020).
7. Marsay, C. M., Sanders, R. J., Henson, S. A., Pabortsava, K., Achterberg, E. P. & Lampitt. R. S. Attenuation of sinking particulate organic carbon flux through the mesopelagic ocean. *Proc. Natl. Acad. Sci. USA* **112**, 1089–1094 (2015).
8. DeVries, T. & Weber., T. The export and fate of organic matter in the ocean: new constraints from combining satellite and oceanographic tracer observations. *Glob. Biogeochem. Cycles* **31**, 535–555 (2017).
9. Stukel, M. R. et al. Mesoscale ocean fronts enhance carbon export due to gravitational sinking and subduction. *Proc. Natl. Acad. Sci. USA* **114**(6), 1252–1257 (2017).
10. Qiu, B., Chen, S., Klein, P., Sasaki, H. & Sasai. Y. Seasonal Mesoscale and Submesoscale Eddy Variability along the North Pacific Subtropical Countercurrent. *J. Phys. Oceanog.* **44**(12), 3079–3098 (2014).
11. Guidi, L. et al. Does eddy-eddy interaction control surface phytoplankton distribution and carbon export in the North Pacific Subtropical Gyre? *J. Geophys. Res.* **117**, G02024 (2012).
12. Zhou, K., Dai, M. Xiu, P., Wang, L., Hu, J. & Benitez-Nelson, C. R. Transient enhancement and decoupling of carbon and opal export in cyclonic eddies. *J. Geophys. Res. Oceans* **125**, e2020JC016372 (2020).
13. Dall'Olmo, G. & Mork., K. A. Carbon export by small particles in the Norwegian Sea. *Geophys. Res. Lett.* **41**, 2921–2927 (2014).
14. Estapa, M. L., Feen, M. L. & Breves., E. Direct observations of biological carbon export from profiling floats in the subtropical North Atlantic. *Global Biogeochem. Cycles* **33**, 282–300 (2019).
15. Kheireddine, M. et al. Organic carbon export and loss rates in the Red Sea. *Global Biogeochem. Cycles* **34**, e2020GB006650 (2020).
16. Stramski, D. et al. Relationships between the surface concentration of

particulate organic carbon and optical properties in the eastern South Pacific and eastern Atlantic Oceans. *Biogeosciences* **5**(1), 171–201 (2008).

17. Belkin, I. M. & O'Reilly., J. E. An algorithm for oceanic front detection in chlorophyll and SST satellite imagery. *J. Mar. Sys.* **78**, 319-326 (2009).
18. Buesseler, K. O. & Boyd, P. W. Shedding light on processes that control particle export and flux attenuation in the twilight zone of the open ocean. *Limnol. Oceanogr.* **54**, 1210–1232 (2009).
19. Grabowski, E., Letelier, R. M., Laws, E. A. & Karl, D. M. Coupling carbon and energy fluxes in the North Pacific Subtropical Gyre. *Nat. Commun.* **10**(1), 1895 (2019).
20. Wiedmann, I. et al. Arctic observations identify phytoplankton community composition as driver of carbon flux attenuation. *Geophys. Res. Lett.* **47**, e2020GL087465 (2020).
21. Buesseler, K. O., Boyd, P. W., Black, E. E. & Siegel, D. A. Metrics that matter for assessing the ocean biological carbon pump. *Proc. Natl. Acad. Sci.* **117**, 201918114 (2020).
