# Supplementary material for: Efficient biological carbon export to the mesopelagic ocean induced by submesoscale fronts
Source: Nat Commun. 2024 Jan 17;15:580. doi: 10.1038/s41467-024-44846-7 (PMC10794176; doi:10.1038/s41467-024-44846-7)
Supplement: Supplementary file 3 — Reporting Summary [file 41467_2024_44846_MOESM3_ESM.pdf]

nature portfolio

Corresponding author(s): Peng Xiu

Last updated by author(s): Jan 2, 2024

Reporting Summary

Nature Portfolio wishes to improve the reproducibility of the work that we publish. This form provides structure for consistency and transparency in reporting. For further information on Nature Portfolio policies, see our Editorial Policies and the Editorial Policy Checklist.

Statistics

For all statistical analyses, confirm that the following items are present in the figure legend, table legend, main text, or Methods section.

n/a	Confirmed	
☐	☒	The exact sample size (*n*) for each experimental group/condition, given as a discrete number and unit of measurement
☒	☐	A statement on whether measurements were taken from distinct samples or whether the same sample was measured repeatedly
☒	☐	The statistical test(s) used AND whether they are one- or two-sided<br>*Only common tests should be described solely by name; describe more complex techniques in the Methods section.*
☒	☐	A description of all covariates tested
☐	☒	A description of any assumptions or corrections, such as tests of normality and adjustment for multiple comparisons
☐	☒	A full description of the statistical parameters including central tendency (e.g. means) or other basic estimates (e.g. regression coefficient) AND variation (e.g. standard deviation) or associated estimates of uncertainty (e.g. confidence intervals)
☒	☐	For null hypothesis testing, the test statistic (e.g. *F*, *t*, *r*) with confidence intervals, effect sizes, degrees of freedom and *P* value noted<br>*Give P values as exact values whenever suitable.*
☒	☐	For Bayesian analysis, information on the choice of priors and Markov chain Monte Carlo settings
☒	☐	For hierarchical and complex designs, identification of the appropriate level for tests and full reporting of outcomes
☒	☐	Estimates of effect sizes (e.g. Cohen's *d*, Pearson's *r*), indicating how they were calculated

Our web collection on statistics for biologists contains articles on many of the points above.

Software and code

Policy information about availability of computer code

Data collection	BGC-Argo and satellite data were collected and analyzed using MATLAB 2022.
Data analysis	BGC-Argo and satellite data were collected and analyzed using MATLAB 2022. The main scripts for data processing and plotting are available at zenodo (https://doi.org/10.5281/zenodo.10294902). The TRACMASS code for Lagrangian particle tracking is available at https://www.tracmass.org/index.html.

For manuscripts utilizing custom algorithms or software that are central to the research but not yet described in published literature, software must be made available to editors and reviewers. We strongly encourage code deposition in a community repository (e.g. GitHub). See the Nature Portfolio guidelines for submitting code & software for further information.

Data

Policy information about availability of data

All manuscripts must include a data availability statement. This statement should provide the following information, where applicable:

- Accession codes, unique identifiers, or web links for publicly available datasets
- A description of any restrictions on data availability
- For clinical datasets or third party data, please ensure that the statement adheres to our policy

The BGC-Argo data was obtained from the BGC-Argo FTP server (ftp://ftp.ifremer.fr/ifremer/argo/dac/csio/). Daily SST and SLA data were downloaded from the EU Copernicus Marine Environment Monitoring Service (CMEMS; https://doi.org/10.48670/moi-00168 and https://doi.org/10.48670/moi-00148). The eddy trajectory

information was downloaded from AVISO's Mesoscale Eddy Trajectory Atlas (https://www.aviso.altimetry.fr/en/data/products/value-added-products/global-mesoscale-eddy-trajectory-product.html). The ocean color data were downloaded from the OceanColor-CCI website (https://www.oceancolor.org). In situ measurement data are available at zenodo (https://doi.org/10.5281/zenodo.10294902).

Research involving human participants, their data, or biological material

Policy information about studies with human participants or human data. See also policy information about sex, gender (identity/presentation), and sexual orientation and race, ethnicity and racism.

Reporting on sex and gender	N/A
Reporting on race, ethnicity, or other socially relevant groupings	N/A
Population characteristics	N/A
Recruitment	N/A
Ethics oversight	N/A

Note that full information on the approval of the study protocol must also be provided in the manuscript.

Field-specific reporting

Please select the one below that is the best fit for your research. If you are not sure, read the appropriate sections before making your selection.

☐ Life sciences ☐ Behavioural & social sciences ☒ Ecological, evolutionary & environmental sciences

For a reference copy of the document with all sections, see nature.com/documents/nr-reporting-summary-flat.pdf

Ecological, evolutionary & environmental sciences study design

All studies must disclose on these points even when the disclosure is negative.

Study description	Analysis of POC transfer efficiency in response to intensified mesoscale eddy and submesoscale dynamics using observations of BGC-Argo floats and satellites.
Research sample	Two BGC-Argo floats were deployed in two cyclonic eddies in the North Pacific subtropical gyres to collect biogeochemical profiles. The BGC-Argo data is available at ftp://ftp.ifremer.fr/ifremer/argo/dac/csio/. Satellite data for analysis were obtained from https://www.aviso.altimetry.fr/, https://www.oceancolor.org and https://marine.copernicus.eu/. In situ measured POC and nitrate were used for calibration of the BGC-Argo data and available at ZENODO via (https://doi.org/10.5281/zenodo.10294902).
Sampling strategy	There are challenges when estimating the POC export flux associated with episodic submesoscale features by either sediment traps or radioactive pairs. To capture the characteristics of vertical carbon transfer efficiency induced by mesoscale and submesoscale dynamics, we deployed two high-frequency sampling floats that profiled twice a day. Biogeochemical profiles are obtained autonomously by the BGC-Argo floats and stored in the BGC-Argo FTP server, available at ftp://ftp.ifremer.fr/ifremer/argo/dac/csio/.
Data collection	Two BGC-Argo floats were deployed in two cyclonic eddies in the North Pacific subtropical gyre during April 2019. Once deployed, the floats park at ~1000 m between profiles. Profile measurements are made when the float ascends to the surface, and data are transmitted via a satellite network before the float descends back to its park depth. Data is received, stored, and available at ftp://ftp.ifremer.fr/ifremer/argo/dac/csio/.
Timing and spatial scale	BGC-Argo data sampling twice a day inside two cyclonic eddies corresponds to the period from 1 April to 3 May 2019. The average sampling distances during the floats in the eddies were 6.45 km and 6.2 km, respectively.
Data exclusions	No data was excluded.
Reproducibility	This research is not based on an experiment. Data analyses were replicated multiple times to ensure consistency and remove computational coding errors.
Randomization	The data presented in this study is not part of an experiment, so data randomization is not necessary in our study.
Blinding	This study is based on observations from BGC-Argo floats and satellites. Since the data presented in this study are not part of an experiment, blinding was not necessary.

Did the study involve field work? ☒ Yes ☐ No

Field work, collection and transport

Field conditions	The BGC-Argo deployment and in situ measurements of POC and nitrate were performed in April 2019 in the North Pacific subtropical gyre, which is spring in the North Pacific Ocean, a time of the year when air temperature starts to increase. Rainfall occurred during the cruise.
Location	The locations were saved in the Source Data file and shown in Fig. 1.
Access & import/export	All samples were collected at the open ocean of the North Pacific subtropical gyre, so no permits are needed.
Disturbance	Disturbance is minimal because all the water masses for analysis were sampled using CTD.

Reporting for specific materials, systems and methods

We require information from authors about some types of materials, experimental systems and methods used in many studies. Here, indicate whether each material, system or method listed is relevant to your study. If you are not sure if a list item applies to your research, read the appropriate section before selecting a response.

Materials & experimental systems

n/a	Involved in the study	
☒	☐	Antibodies
☒	☐	Eukaryotic cell lines
☒	☐	Palaeontology and archaeology
☒	☐	Animals and other organisms
☒	☐	Clinical data
☒	☐	Dual use research of concern
☒	☐	Plants

Methods

n/a	Involved in the study	
☒	☐	ChIP-seq
☒	☐	Flow cytometry
☒	☐	MRI-based neuroimaging
