# Supplementary material for: Efficient biological carbon export to the mesopelagic ocean induced by submesoscale fronts
Source: Nat Commun. 2024 Jan 17;15:580. doi: 10.1038/s41467-024-44846-7 (PMC10794176; doi:10.1038/s41467-024-44846-7)
Supplement: Supplementary file 2 — Peer Review File [file 41467_2024_44846_MOESM2_ESM.pdf]

nature portfolio

Peer Review File

Efficient biological carbon export to the mesopelagic ocean induced by submesoscale fronts

Open Access This file is licensed under a Creative Commons Attribution 4.0 International License, which permits use, sharing, adaptation, distribution and reproduction in any medium or format, as long as you give appropriate credit to the original author(s) and the source, provide a link to the Creative Commons license, and indicate if changes were made. In the cases where the authors are anonymous, such as is the case for the reports of anonymous peer reviewers, author attribution should be to 'Anonymous Referee' followed by a clear attribution to the source work. The images or other third party material in this file are included in the article's Creative Commons license, unless indicated otherwise in a credit line to the material. If material is not included in the article's Creative Commons license and your intended use is not permitted by statutory regulation or exceeds the permitted use, you will need to obtain permission directly from the copyright holder. To view a copy of this license, visit http://creativecommons.org/licenses/by/4.0/.

REVIEWER COMMENTS

Reviewer #1 (Remarks to the Author):

This study presents an observation-based analysis of the relationship between particulate organic carbon (POC) and eddy and frontal dynamics. The authors have leveraged observations from floats with the objective to bring new insights into carbon export dynamics at fine scale. This is a valuable effort; yet there are major issues that need to be addressed before it can be published. The main concerns fall in 4 categories:

I. Gaps in context and references to prior work in introduction and discussion of the results, specifically the potential impact of frontal subduction (comment #1)

II. Missing or weak justification of the hypotheses used to interpret the data that account for the compounding effects of spatial and temporal variability, specifically the justification to use a "mostly spatial" or a "mostly temporal" framework depending on the eddy/front considered (comments #2-5)

III. Shortcomings with the conclusions and generalizations of the results that link fronts to POC export and attenuation coefficient b, which is the main point of the paper (comments # 6-10).

IV. Imprecisions on the approach that need clarification, issues with text and figures. (comments #11-28).

Detailed comments and suggestions on how to address them are provided below. The two reviewers that contributed to this review believe the authors can improve the justification of their approach, as well as the interpretation and generalization so the manuscript can be acceptable for publication.

1. Gaps in context and references. The sentence of L71 is not true: "In this study, for the first time, we show the capability of using BGC-Argo floats equipped with optical sensors to estimate the carbon export flux and associated vertical attenuation in response to submesoscale fronts"

The paper is lacking key references, in particular prior studies identifying and quantifying the role of subduction by fronts, including with BGC-Argo floats. The manuscript leads us to believe that there were no attempts to quantify this subduction pump before. Yet observation based (using float AOU, spiciness) and high resolution modeling papers have been published on this topic (see list of reference below). They need to be included to provide the adequate context in the introduction but also in the discussion. Note that the process of export by subduction along fronts explained in this study can be called differently in the literature: eddy pump, eddy subduction pump etc. Although the term "eddy pump" was often used, it does not usually refer to "Ekman pumping" in eddies but the subduction by fast velocities associated with meso and submesoscale fronts, similarly to what is considered in this manuscript.

Sentences that critically need these references include L56-67 (almost all sentences in this paragraph are missing critical references), L71 which needs to integrate these prior work. In discussion, please consider

how the impact of subduction would influence your results and discuss considering these prior works.

Review about export pumps including the physical injection pump or eddy pump:
Boyd, P.W., Claustre, H., Levy, M., Siegel, D.A., Weber, T., 2019. Multi-faceted particle pumps drive carbon sequestration in the ocean. Nature 568, 327. https://doi.org/10.1038/s41586-019-1098-2

Papers estimating the role the "eddy subduction pump" using BGC-Argo, cruises and models:
Erickson, Z.K., Thompson, A.F., 2018. The Seasonality of Physically Driven Export at Submesoscales in the Northeast Atlantic Ocean. Global Biogeochemical Cycles 32. https://doi.org/10.1029/2018GB005927
Lacour, L., Llort, J., Briggs, N., Strutton, P.G., Boyd, P.W., 2023. Seasonality of downward carbon export in the Pacific Southern Ocean revealed by multi-year robotic observations. Nat Commun 14, 1278. https://doi.org/10.1038/s41467-023-36954-7
Llort, J., Langlais C., Matear R., Moreau S., Lenton A., Strutton Peter G., 2018. Evaluating Southern Ocean Carbon Eddy-Pump From Biogeochemical-Argo Floats. Journal of Geophysical Research: Oceans 123, 971–984. https://doi.org/10.1002/2017JC012861
McGillicuddy, D.J., Resplandy, L., Lévy, M., 2019. Estimating particle export flux from satellite observations: Challenges associated with spatial and temporal decoupling of production and export. Journal of Marine Research 77, 247–258. https://doi.org/10.1357/002224019828474331
Omand, M.M., D'Asaro, E.A., Lee, C.M., Perry, M.J., Briggs, N., Cetinić, I., Mahadevan, A., 2015. Eddy-driven subduction exports particulate organic carbon from the spring bloom. Science 348, 222–225. https://doi.org/10.1126/science.1260062
Resplandy, L., Lévy, M., McGillicuddy Jr., D.J., 2019. Effects of Eddy-Driven Subduction on Ocean Biological Carbon Pump. Global Biogeochemical Cycles 33, 1071–1084. https://doi.org/10.1029/2018GB006125
Stukel, M.R., Song, H., Goericke, R., Miller, A.J., 2017. The role of subduction and gravitational sinking in particle export, carbon sequestration, and the remineralization length scale in the California Current Ecosystem: Subduction and sinking particle export in the CCE. Limnology and Oceanography 63, 363–383. https://doi.org/10.1002/lno.10636

2. Missing justification of the hypotheses, specifically the justification to use a "mostly spatial" or a "mostly temporal" framework to interpret the float data.
The authors alternatively interpret the observed changes along the float trajectories as "mostly spatial changes" (CE1 and ACE1, Figure 2), or "mostly temporal changes" i.e. pseudo lagrangian (CE2 and front, Figure 3). The hypothesis of "mostly temporal" is justified in the text for CE2 (L128 and 141). However, the justification to interpret as "mostly spatial" the changes along CE1 and ACE1 is missing, and the hypothesis to interpret as "mostly temporal" the changes attributed to frontal dynamics seems inadequate (see details in comments 3 and 4 below). We encourage the authors to explain clearly and

early these different strategies to orient and prepare the reader.

3. The authors need to demonstrate why it is justified to ignore temporal variations when analyzing CE1 and ACE1. Figure 2 interprets all POC data as spatial changes (using radial structure of the eddies CE1 and ACE1). The approach is attractive, but the text and figures should clearly show that this is a valid hypothesis. Specifically, how much of the signal that is interpreted as spatial variability across the eddy is in fact introduced by the temporal evolution along the trajectory of the float? At least, the authors could indicate the day of sampling for each data point on Figure 2? [Colors could indicate the day of sampling while symbols could indicate the depth]. This would allow the reader to see how much of the variations attributed to the spatial structure of the eddy could be introduced by temporal changes.

4. The hypothesis of "mostly temporal" is highly disputable for the front. It is ok for eddy CE2 because the float seems to stay stuck close to the center of the eddy, and most changes can be interpreted as temporal changes. In the case of the front, however, the float seems to be sampling different parts of the front. The associated changes in SLA, POC, AOU etc. are therefore likely to come from spatial variations across the front and not temporal variations. The authors could demonstrate that it is indeed temporal, however it is unlikely to be successful and convincing given the complexity of the dynamics in the frontal structure. This shortcoming has major implications for the result interpretation and the conclusions of the paper as the authors interpret these changes as temporal changes while they are likely spatial changes. This section of the results and associated conclusions needs to be revised.

5. The sampling context should be better presented. In Figure 1, eddies and float trajectories appear completely disconnected. This is likely because the black lines show the center of the eddy and not their span. Could you indicate the radius of the eddies around the trajectories so we can see that they were indeed in the eddies? Also we encourage the authors to include a figure similar to Figure S4a to show the float relative to the eddy/front. Maybe choose snapshots that show the overlap with the front as a function of time.

6. Generalization between front and carbon export. L214-222. "A strong correlation between the b value and GM was found …. This statistical relationship implies that a strong front is linked with high POC transfer efficiency to the mesopelagic layer, which could be used as a parameterization to account for the impact of fronts on carbon export in large-scale models, once the front intensity at the surface is known". This conclusion seems not backed up by the results. More complete models seem to show that b can vary according to many other biophysical parameters, making this generalization highly debatable (see for instance Ngyuen et al. 2022: Microbes contribute to setting the ocean carbon flux by altering the fate of sinking particulates). This is one of the main points of the paper; the caveats need to be discussed properly.

7. Conclusion about seasonality and export. line 234: You did not take into account that the seasonality of mesoscale and frontal dynamics is different from the seasonality of POC content in the water column. Consequently, the amount of POC transported by the same front strength varies depending on the season. This could completely reverse your conclusion about the seasonal evolution of the parameter b.

Please revise or strengthen these points.

8. Missing consideration of subduction #1. The POC export depth by fronts also depends on the penetration depth of the vertical velocities associated with the fronts (subduction, see comment #1 for a list of papers that describe this). Shallow fronts do not allow POC to be exported below the pycnocline and would limit the frontal effect on b, further weakening your conclusion and the link established between fronts and POC export/attenuation coefficient. Please consider this effect of subduction in introduction and importantly in your results and interpretation. This is one of the weakest point of the paper. See also comment 9 below.

9. Missing consideration of subduction #2. L118: “The POC distributions attenuated faster in the CE 1 center than at its edge, which is likely due to the higher remineralization of the newly produced and easily degradable organic matter inside the eddy core8,36,37.” Is this really what is happening here? Couldn’t it be that intense vertical velocities associated with the frontal dynamics at the boundary of the eddies are transporting/subducting material down?

10. Generalization to the subtropical Pacific. The generalization to the basin is very hand wavy and unconvincing, and probably not needed here. The results can stand alone without this. The author could in fact point out that the impact at the basin scale and global scale is still an open question. The conclusion, abstract (and maybe title) should be modified to reflect the changes in conclusions.

Imprecisions on the approach that need clarification, issues with text and figures.

Section 2.1

11. Line 97 and onward. This paragraph about the position of the floats is a little tedious to read. Maybe the authors could either streamline or fold in some results in there as they describe the trajectory.

12. Line 107: “The euphotic depth (ZEu) was on average ~159 m within CE 1 and 161 m in ACE” is this difference really significant?

13. line 115: Correlations are reversed: change increasing to decreasing and vice versa.

14. Line 116: “This result suggested that nutrients in the euphotic zone were enhanced by the uplift of isotherms within this cyclonic eddy17,” Don’t you mean that uplift occurs at the edge of CE instead of “within”?

15. line 123: Trends are reversed: change positive to negative and vice versa.

16. Text on the figure was blurry, small and hard to read in my version.

17. Zscm is used to discuss the changes in the different properties measured. Could you explain why? How to separate the effect of the eddy dynamics and the change in sampling depth due to variations in Zscm? Is the assumption that Zscm follows a density layer? Maybe using density instead of depth would be more insightful?

Section 2.2

18. Please state clearly that you attribute change in integrated content over time to change in flux from the top, and detail and justify your assumptions.

19. More arguments could be provided to justify the reason for comparing the attenuation of a flux anomaly (FEI(z)) calculated with the float with the attenuation of the total POC flux from the literature.
20. Write P(z), E(z) on figure 3 to make it easier to understand.
21. line 153: Is «exponentially» appropriate? It seems linear.
22. line 162: The total increase in POC flux could be calculated by reconstructing the amount of remineralized POC from AOU and Redfield ratios.
23. Y-scales of 3g and 3h are making it confusing, maybe 0-700 m could be used.

Section 2.3
24. L 183: Here, the front is defined by the temperature gradient. Would it be possible to find out whether the front is dynamically active and carrying POC at depth or not?

25. L184/185: Can we really speak of degradation/intensification, since the float crosses the front but doesn't stay there?

26. L 186 / Fig. S4e: Why is the gradient lower than the background during the decaying stage? Why not equal since the front goes back to background?

27. L 188: Fig. S4g: How is the strain rate calculated and from which data product? You could physically define the front and add its position to your figures to make interpretation easier.

Methods

28. L 313: You could spell out bbp(700) and PAR again.

Reviewer #2 (Remarks to the Author):

This study presents an observation-based analysis of the relationship between particulate organic carbon (POC) and eddy and frontal dynamics. The authors have leveraged observations from floats with the objective to bring new insights into carbon export dynamics at fine scale. This is a valuable effort; yet there are major issues that need to be addressed before it can be published. The main concerns fall in 4 categories:

Gaps in context and references to prior work in introduction and discussion of the results, specifically the potential impact of frontal subduction (comment #1)
Missing or weak justification of the hypotheses used to interpret the data that account for the compounding effects of spatial and temporal variability, specifically the justification to use a “mostly spatial” or a “mostly temporal” framework depending on the eddy/front considered (comments #2-5)

Shortcomings with the conclusions and generalizations of the results that link fronts to POC export and attenuation coefficient b, which is the main point of the paper (comments # 6-10).
Imprecisions on the approach that need clarification, issues with text and figures. (comments #11-28).

Detailed comments and suggestions on how to address them are provided below. The two reviewers that contributed to this review believe the authors can improve the justification of their approach, as well as the interpretation and generalization so the manuscript can be acceptable for publication.

Gaps in context and references. The sentence of L71 is not true: "In this study, for the first time, we show the capability of using BGC-Argo floats equipped with optical sensors to estimate the carbon export flux and associated vertical attenuation in response to submesoscale fronts"

The paper is lacking key references, in particular prior studies identifying and quantifying the role of subduction by fronts, including with BGC-Argo floats. The manuscript leads us to believe that there were no attempts to quantify this subduction pump before. Yet observation based (using float AOU, spiciness) and high resolution modeling papers have been published on this topic (see list of reference below). They need to be included to provide the adequate context in the introduction but also in the discussion. Note that the process of export by subduction along fronts explained in this study can be called differently in the literature: eddy pump, eddy subduction pump etc. Although the term "eddy pump" was often used, it does not usually refer to "Ekman pumping" in eddies but the subduction by fast velocities associated with meso and submesoscale fronts, similarly to what is considered in this manuscript.

Sentences that critically need these references include L56-67 (almost all sentences in this paragraph are missing critical references), L71 which needs to integrate these prior work. In discussion, please consider how the impact of subduction would influence your results and discuss considering these prior works.

Review about export pumps including the physical injection pump or eddy pump:
Boyd, P.W., Claustre, H., Levy, M., Siegel, D.A., Weber, T., 2019. Multi-faceted particle pumps drive carbon sequestration in the ocean. Nature 568, 327. https://doi.org/10.1038/s41586-019-1098-2

Papers estimating the role the "eddy subduction pump" using BGC-Argo, cruises and models:
Erickson, Z.K., Thompson, A.F., 2018. The Seasonality of Physically Driven Export at Submesoscales in the Northeast Atlantic Ocean. Global Biogeochemical Cycles 32. https://doi.org/10.1029/2018GB005927
Lacour, L., Llort, J., Briggs, N., Strutton, P.G., Boyd, P.W., 2023. Seasonality of downward carbon export in the Pacific Southern Ocean revealed by multi-year robotic observations. Nat Commun 14, 1278. https://doi.org/10.1038/s41467-023-36954-7
Llort, J., Langlais C., Matear R., Moreau S., Lenton A., Strutton Peter G., 2018. Evaluating Southern Ocean Carbon Eddy-Pump From Biogeochemical-Argo Floats. Journal of Geophysical Research: Oceans 123,

971–984. https://doi.org/10.1002/2017JC012861
McGillicuddy, D.J., Resplandy, L., Lévy, M., 2019. Estimating particle export flux from satellite observations: Challenges associated with spatial and temporal decoupling of production and export. Journal of Marine Research 77, 247–258. https://doi.org/10.1357/002224019828474331
Omand, M.M., D'Asaro, E.A., Lee, C.M., Perry, M.J., Briggs, N., Cetinić, I., Mahadevan, A., 2015. Eddy-driven subduction exports particulate organic carbon from the spring bloom. Science 348, 222–225. https://doi.org/10.1126/science.1260062
Resplandy, L., Lévy, M., McGillicuddy Jr., D.J., 2019. Effects of Eddy-Driven Subduction on Ocean Biological Carbon Pump. Global Biogeochemical Cycles 33, 1071–1084. https://doi.org/10.1029/2018GB006125
Stukel, M.R., Song, H., Goericke, R., Miller, A.J., 2017. The role of subduction and gravitational sinking in particle export, carbon sequestration, and the remineralization length scale in the California Current Ecosystem: Subduction and sinking particle export in the CCE. Limnology and Oceanography 63, 363–383. https://doi.org/10.1002/lno.10636

Missing justification of the hypotheses, specifically the justification to use a "mostly spatial" or a "mostly temporal" framework to interpret the float data.
The authors alternatively interpret the observed changes along the float trajectories as "mostly spatial changes" (CE1 and ACE1, Figure 2), or "mostly temporal changes" i.e. pseudo lagrangian (CE2 and front, Figure 3). The hypothesis of "mostly temporal" is justified in the text for CE2 (L128 and 141). However, the justification to interpret as "mostly spatial" the changes along CE1 and ACE1 is missing, and the hypothesis to interpret as "mostly temporal" the changes attributed to frontal dynamics seems inadequate (see details in comments 3 and 4 below). We encourage the authors to explain clearly and early these different strategies to orient and prepare the reader.

The authors need to demonstrate why it is justified to ignore temporal variations when analyzing CE1 and ACE1. Figure 2 interprets all POC data as spatial changes (using radial structure of the eddies CE1 and ACE1). The approach is attractive, but the text and figures should clearly show that this is a valid hypothesis. Specifically, how much of the signal that is interpreted as spatial variability across the eddy is in fact introduced by the temporal evolution along the trajectory of the float? At least, the authors could indicate the day of sampling for each data point on Figure 2? [Colors could indicate the day of sampling while symbols could indicate the depth]. This would allow the reader to see how much of the variations attributed to the spatial structure of the eddy could be introduced by temporal changes.

The hypothesis of "mostly temporal" is highly disputable for the front. It is ok for eddy CE2 because the float seems to stay stuck close to the center of the eddy, and most changes can be interpreted as temporal changes. In the case of the front, however, the float seems to be sampling different parts of the front. The associated changes in SLA, POC, AOU etc. are therefore likely to come from spatial variations across the front and not temporal variations. The authors could demonstrate that it is indeed temporal, however it is unlikely to be successful and convincing given the complexity of the dynamics in the frontal structure. This shortcoming has major implications for the result interpretation and the conclusions of

the paper as the authors interpret these changes as temporal changes while they are likely spatial changes. This section of the results and associated conclusions needs to be revised.

The sampling context should be better presented. In Figure 1, eddies and float trajectories appear completely disconnected. This is likely because the black lines show the center of the eddy and not their span. Could you indicate the radius of the eddies around the trajectories so we can see that they were indeed in the eddies? Also we encourage the authors to include a figure similar to Figure S4a to show the float relative to the eddy/front. Maybe choose snapshots that show the overlap with the front as a function of time.

Generalization between front and carbon export. L214-222. “A strong correlation between the b value and GM was found …. This statistical relationship implies that a strong front is linked with high POC transfer efficiency to the mesopelagic layer, which could be used as a parameterization to account for the impact of fronts on carbon export in large-scale models, once the front intensity at the surface is known”. This conclusion seems not backed up by the results. More complete models seem to show that b can vary according to many other biophysical parameters, making this generalization highly debatable (see for instance Ngyuen et al. 2022: Microbes contribute to setting the ocean carbon flux by altering the fate of sinking particulates). This is one of the main points of the paper; the caveats need to be discussed properly.

Conclusion about seasonality and export. line 234: You did not take into account that the seasonality of mesoscale and frontal dynamics is different from the seasonality of POC content in the water column. Consequently, the amount of POC transported by the same front strength varies depending on the season. This could completely reverse your conclusion about the seasonal evolution of the parameter b. Please revise or strengthen these points.

Missing consideration of subduction #1. The POC export depth by fronts also depends on the penetration depth of the vertical velocities associated with the fronts (subduction, see comment #1 for a list of papers that describe this). Shallow fronts do not allow POC to be exported below the pycnocline and would limit the frontal effect on b, further weakening your conclusion and the link established between fronts and POC export/attenuation coefficient. Please consider this effect of subduction in introduction and importantly in your results and interpretation. This is one of the weakest point of the paper. See also comment 9 below.

Missing consideration of subduction #2. L118: “The POC distributions attenuated faster in the CE 1 center than at its edge, which is likely due to the higher remineralization of the newly produced and easily degradable organic matter inside the eddy core8,36,37.” Is this really what is happening here? Couldn’t it be that intense vertical velocities associated with the frontal dynamics at the boundary of the eddies are transporting/subducting material down?

Generalization to the subtropical Pacific. The generalization to the basin is very hand wavy and unconvincing, and probably not needed here. The results can stand alone without this. The author could

in fact point out that the impact at the basin scale and global scale is still an open question. The conclusion, abstract (and maybe title) should be modified to reflect the changes in conclusions.

Imprecisions on the approach that need clarification, issues with text and figures.

Section 2.1

Line 97 and onward. This paragraph about the position of the floats is a little tedious to read. Maybe the authors could either streamline or fold in some results in there as they describe the trajectory.

Line 107: "The euphotic depth (ZEu) was on average ~159 m within CE 1 and 161 m in ACE" is this difference really significant?

line 115: Correlations are reversed: change increasing to decreasing and vice versa.

Line 116: "This result suggested that nutrients in the euphotic zone were enhanced by the uplift of isotherms within this cyclonic eddy17," Don't you mean that uplift occurs at the edge of CE instead of "within"?

line 123: Trends are reversed: change positive to negative and vice versa.

Text on the figure was blurry, small and hard to read in my version.

Zscm is used to discuss the changes in the different properties measured. Could you explain why? How to separate the effect of the eddy dynamics and the change in sampling depth due to variations in Zscm? Is the assumption that Zscm follows a density layer? Maybe using density instead of depth would be more insightful?

Section 2.2

Please state clearly that you attribute change in integrated content over time to change in flux from the top, and detail and justify your assumptions.

More arguments could be provided to justify the reason for comparing the attenuation of a flux anomaly (FEI(z)) calculated with the float with the attenuation of the total POC flux from the literature.

Write P(z), E(z) on figure 3 to make it easier to understand.

line 153: Is «exponentially» appropriate? It seems linear.

line 162: The total increase in POC flux could be calculated by reconstructing the amount of remineralized POC from AOU and Redfield ratios.

Y-scales of 3g and 3h are making it confusing, maybe 0-700 m could be used.

Section 2.3

L 183: Here, the front is defined by the temperature gradient. Would it be possible to find out whether the front is dynamically active and carrying POC at depth or not?

L184/185: Can we really speak of degradation/intensification, since the float crosses the front but doesn't stay there?

L 186 / Fig. S4e: Why is the gradient lower than the background during the decaying stage? Why not equal since the front goes back to background?

L 188: Fig. S4g: How is the strain rate calculated and from which data product? You could physically define the front and add its position to your figures to make interpretation easier.

Methods

L 313: You could spell out bbp(700) and PAR again.

Reviewer #3 (Remarks to the Author):

Efficient biological carbon export to the mesopelagic ocean induced by submesoscale fronts in subtropical gyres
Guo, M., Xing, X., et al.
Nature Communications
5 June 2023

This study presents data from BGC-Argo floats along with interpolated satellite remote sensing data to investigate the role of processes at the submesoscale in shaping carbon export. The authors find that, in the North Pacific subtropical gyre, processes at the mesoscale and submesoscale may play a significant role in transporting carbon from the surface to the interior ocean. In particular, they note that the previous assumption of a relationship of carbon attenuation with temperature may be better considered as a relationship between attenuation and SST gradient magnitude. Their suggested relationship could help better constrain carbon cycling in the subtropical gyres in numerical models.

I would like to thank the authors for a clearly-written manuscript with many high-quality figures. I enjoyed reading this manuscript and felt it presented a compelling case for considering carbon export at the submesoscale in the subtropical ocean. Carbon export is an important and timely topic, and I appreciate the care with which the authors present the data. I have a few concerns that I hope the authors will address in order to make the work more accessible to the reader. In addition, while the manuscript generally does a nice job at describing limitations with the work, there are a few additional constraints to consider, as detailed below. My major concerns are documented below; there were also several minor concerns that I think the manuscript would benefit from addressing.

Major Concerns

Definition of the submesoscale front: The submesoscale front described in this manuscript is an interesting feature, but the manuscript does not yet convince me that I am seeing a density front and therefore that the resulting POC analysis is attributable to the feature. First, the front is diagnosed from SST and SST gradient. This ignores the potential contribution of salinity. To remedy this, I would like to

see section plots (time versus depth) of the float's temperature and salinity over this period (April 21-May 3) to visualize the depth of this front.

In addition, Figure S4 is stated to be of vertical buoyancy frequency, but that is not possible as in a stable water column, N2 must solely be positive. I imagine the authors may have instead calculated lateral buoyancy frequency (M2), but the magnitude of the values shown are extremely high for this property; M2 values of strong submesoscale fronts in the Southern Ocean are of O(10^-7) (Siegelman et al. 2020). I urge the authors to check what they are calculating here. I do appreciate the inclusion of strain rate, and the authors could also consider using Finite Size Lyapunov Exponents (AVISO+, https://www.aviso.altimetry.fr/en/data/products/value-added-products/fsle-finite-size-lyapunov-exponents.html) to demonstrate the presence of the frontal structure.

Sea level anomaly data: I greatly appreciate the authors' use of in situ data combined with remote sensing data. However, sea surface height data provided by AVISO typically has a footprint of approximately ¼ degree. While this can be manageable for mesoscale work, it is often too coarse for detection of submesoscale features. I am less familiar with satellite SST footprint, but I imagine it is of similar scale. I would like to see the authors discuss this potential limitation and how it might impact their results. I appreciate that there is some discussion of how the calculations from the SST gradient magnitude:attenuation rate relationship, and I think a discussion of how even remote sensing does not capture the submesoscale fully would fit in here. Addressing this concern, as well as my other concerns above and below, would help me feel more comfortable in the extrapolation of data from two eddies to the full Northern Hemisphere subtropical gyres.

Minor Concerns
Line 26: unnecessary "however"
Line 32: Is it clear that submesoscale motions would result in a net sink of CO2 in the subtropical gyres, or is there evidence that high DIC water from the mesopelagic could be brought up to the service and outgas?
Line 41: "relatively fast of remineralization": unnecessary "of," and can you state what region(s) you are comparing remineralization rate against?
Line 42: unnecessary and confusing "on the other hand"
Line 48: I suggest changing "leading to" to "resulting in"
Lines 49-52: Consider adding citations to Mahadevan (2016) (doi: 10.1146/annurev-marine-010814-015912) and Klein & Lapeyre (2009) (doi: 10.1146/annurev.marine.010908.163704).
Lines 54-55: Consider providing a short summary of why plankton size distribution impacts POC export rates (I imagine because of differing contributions by the gravitational pump?)
Line 56: Consider changing "However," to "Despite their potential importance to shape POC export," to make continuity stronger
Lines 71-73: Have the authors seen Llort et al. (2018) (doi: https://doi.org/10.1002/2017JC012861). While they use a different method, I suggest their work was among the first to estimate carbon flux from BGC-Argo. I do agree that this manuscript's work looking at vertical attenuation is novel, so I suggest rephrasing this sentence to recognize previous efforts and clarify the novel elements of this manuscript.

Somewhere in the introduction, it would enhance your paper's reach to introduce the terms "mesoscale" and "submesoscale" dynamically - potentially defining them with the Rossby number or some other scaling.
Line 90: Please point the reader to Figure 1b at this point - I got quite lost reading until I was able to match up with the figure.
Lines 90-92: I am confused by this sentence - are you saying that you look at the slope of the isopycnals to determine intensification?
Line 93: Again, please cite Figure 1c.
Lines 95-96: As someone who is used to working with BGC-Argo floats that are sampling at the traditional rate of 10 days, it would help to know earlier that the floats had more regular sampling. Please consider moving the content of this sentence to earlier in the paragraph.
Figure 1: Change blue and red dots to colors that are more visible on the chosen colorbar as the representations for NO3- and POC sampling stations.
Line 111: What was the average depth of the subsurface chlorophyll maximum?
Lines 116-118: I generally agree with the statement that there are higher POC concentrations in the CE center than the edge, but I would appreciate some commentary on the POC peaks at r/R =~ 1 and 1.3 at 200 m.
Line 122: Add a semicolon before "that is"
Lines 124-125: Perhaps I am missing something obvious, but Figure 2b illustrates to me that POC concentrations are higher at the ACE center than the edge at 200 m?
Figure 2: Why are standard deviations only shaded for some points?
Line 130: Specifically cite Figure 1d.
Line 142: Note that you are defining stable based on changes in r/R.
Lines 146-148: Can this linear least squares regression plot be included in the supplemental information to provide a sense of how different the calculated E(z) values are? Alternatively, one could include error bars on Figures 3c and 3d.
Comment only: I really like the inclusion of AOU/oxygen utilization rate to investigate biological activity!
Line 174: Consider mentioning that the Martin curve has long been used as the standard for understanding POC attenuation across the global ocean for those who are not familiar with the concept.
Section 2.4: The manuscript appears to argue that the relationship between SST gradient magnitude and the b value is consistent across both mesoscale eddies and submesoscale fronts (as implied in Figure 4). However, the authors do a nice job in the introduction explaining why there are different rates of export by mesoscale and submesoscale processes, so I am not so sure that the two can be compared in this way. I'd appreciate some explanation or breakdown of the limitations of the SST GM:b relationship, particularly with respect to spatiotemporal scales.
Figure S6: A different colorbar (perhaps cmocean's "algae" colorbar, https://matplotlib.org/cmocean/) would be a better choice for this figure.
Figure 5: Please consider using different colorbars for panels a and c versus b and d. At first glance, instinct is to compare a and b versus c and d. Different colorbars will make the story more clear and enhance understanding of your discussion.
Figure S7: Is it possible to put the eddies investigated in this study (CE 1 and 2) on panel b as a comparison? This could go a long way in solidifying the application of your parameterization.

Lines 304-307: While I agree that BGC-Argo floats can be used for this type of investigation, as evidenced by the nice work in this manuscript, I would include a caveat that the current 10-day sampling regime employed by the Argo programme runs the risk of not capturing the submesoscale and mesoscale processes described in this paper.

Reviewer #4 (Remarks to the Author):

Review comment

The manuscript by Wang et al., used two high-frequency sampling BGC-Argo deployed in the western Pacific to investigate the impact of the eddies on the POC export. The authors reported enhanced POC export in the regions affected by intensifying submesoscale fronts and further meta-analysis of a global compiled dataset revealed a prevailing relationship between transfer efficiency and the strength of submesoscale front (indicative of surface temperature gradient). Finally, the authors upscaled the contribution of eddies to the carbon export in the entire northern subtropical Pacific gyre by synthesizing the frequency of eddy occurrence.

However, I find several noteworthy loopholes in the BGC-Argo data analysis, specially related to the observed AOU rate, and its discrepancy with the POC rate. See my explicit comments below:

1) Unexpected, negative change of AOU and the discrepancy between AOU and POC rate:

Figure 3 shows the author derived negative values for the time rate of AOU in many cases, the implication being that twilight zone oxygen increases over time. This tendency is somewhat surprising to me, as we generally expect a declining trend of oxygen over time below the euphotic zone resulting from biological respiration, regardless of the relative change in upper POC flux.

Furthermore, the reported magnitude of AOU rate appears to be unrealistically high. The depth-integrated AOU rates between 150m (or 170m) and 900m fall within the range of 250-500 mmol m-2 d-1, equivalent to 90-180 mmol m-2 yr-1. For comparison, the global range of upper carbon export is only typically 1-4 mol C m-2 yr-1. These extremely high AOU rates also are sharply analogous relative to export flux solved from POC inventory, which falls within the range of 1-2 mmol C m-2 d-1.

I suspect there are two possible reasons for the discrepancies. It is well known that the evolution of eddy typically results in a significant displacement of water masses. Thereby, observed AOU and POC change rates along the isobars may reflect a confounding result of both biological and physical processes. Therefore, to better isolate the biologically-induced tracer change that the authors are interested in, it would be more appropriate to quantify the time rate change of AOU (and POC as well) along with the isopycncal surfaces.

The second possible reason is the float didn't exactly track the same water mass and may gradually move towards a region featured with a higher pre-formed oxygen concentration. To rule out this possibility, a more rigorous examination of the float hydrographic data and comparison with background spatial oxygen gradients from existing gridded products (e.g., WOA and GO-BAI oxygen product) and POC gradients (e.g., climatology of remotely sensed POC) is necessary.

2) Is it reasonable to interpret the decreasing trend of POC inventory as an enhanced POC export?

In principle, when there is an enhanced upper layer POC export event, we usually expect to observe an increase in the POC inventory in the mesopelagic zone, along with a simultaneous elevation of respiration (reflected by an increase in AOU).

Nevertheless, the author observed a decline in POC inventory. As the authors mentioned, the POC inventory change in the twilight zone reflects a net balance between the supply term from the upper layer and removal due to the local respiration (net POC inventory change= POC_sinking_upper-respiration). There are several possible scenarios that can explain the POC inventory decline, including:

- Enhanced respiration outpaces the concurrent elevated upper layer POC export (the scenario that authors have speculated).
- Elevated respiration occurs in the context of a constant upper layer POC export.
- A decline in the POC export accompanied by constant respiration).

It is challenging to rule out other possibilities unless the authors have the information on the respiration rates, which would enable to back-calculate the exact magnitude of upper layer POC export (POC_sinking_upper = respiration+ net POC inventory change).

Other comments:

Line 32: the eddy-induced upwelling also brings subsurface water rich in DIC into the surface, which may drive the CO2 outgains.

Line 55: it may be worth mentioning the potential impact of eddy-driven carbon export on helping reconcile the long-standing paradox regarding the imbalance between the upper layer carbon supply and deep ocean carbon consumption.

Line 86: a in the Chlorophyll-a should be italicized.
Line 131: What kind of threshold or criteria do you apply to define the start and end period of the intensifying phase of eddy (i.e., the amplitude continuously increased over more than one week as you mentioned in the SI). I tried to sort it out from Figure 1 but didn't find a very consistent criterion. It is very important information in your study, so it requires clear clarification in the main text of the manuscript.

Line 241: p should be lowercase and italicized.

.

Line 302: There is a need to discuss the caveats of the current study and provide a future roadmap to address these challenges.

Line 303: A more in-depth discussion is required regarding the potential implications of your work, such as the imbalance between carbon supply and demand. It would be also eye-opening to discuss the depth to which eddies can affect carbon export transfer, and its significance for long-term carbon sequestration, considering that carbon sequestration time is positive with the depth where carbon is eventually delivered.

Line 311: should provide the sampling information of temporal and vertical resolution.

Line 314: Why did you use the dark correction value collected during the period before the float deployment? In the standard Chl-a quality control procedure, the dark correction typically refers to in situ values at the depths around d 200-300m to account for the near-real-time background noise.

Line 323: add the sample number and relevant statistical information (i.e., R2 and p values). There should be a space between “mmol” and “C”.

Figure 3: Are there uncertainty estimates for AOU and POC rates? At the very least, it would take into account the error inherited from the linear fitting. What is unit of AOU rate (mmol C or mmol O2)?

Line 344: Although the authors have briefly described the method to derive the time rate of POC and AOU inventory in the result section, it is still worthwhile providing more detailed information in the method section. It would be more appropriate to quantify the POC and AOU rate alongside the isopycnal surface to reduce the impact of the tracer rate induced by water vertical displacement.

Figure 1: What is the temporal scale of background EKE? Should be clarified in the caption. All the acronyms shown in the figures should be spelled out in the caption to make the figure self-explanatory. Does the black line indicate the position of the core of the eddy? Should make it clear.

Responses to Reviewers

We thank the four reviewers for their constructive comments and suggestions, which we believe have considerably improved our manuscript now entitled ‘Efficient biological carbon export to the mesopelagic ocean induced by submesoscale fronts’. We have responded point-by-point to the reviewers’ questions and comments below. All page/line/reference/figure numbers refer to the tracked manuscript. Reviewers’ comments are in regular text, our responses are in blue and new text from the manuscript is in *blue italics.*

Reviewer #1 (Remarks to the Author):

This study presents an observation-based analysis of the relationship between particulate organic carbon (POC) and eddy and frontal dynamics. The authors have leveraged observations from floats with the objective to bring new insights into carbon export dynamics at fine scale. This is a valuable effort; yet there are major issues that need to be addressed before it can be published. The main concerns fall in 4 categories:

I. Gaps in context and references to prior work in introduction and discussion of the results, specifically the potential impact of frontal subduction (comment #1)

II. Missing or weak justification of the hypotheses used to interpret the data that account for the compounding effects of spatial and temporal variability, specifically the justification to use a “mostly spatial” or a “mostly temporal” framework depending on the eddy/front considered (comments #2-5)

III. Shortcomings with the conclusions and generalizations of the results that link fronts to POC export and attenuation coefficient b, which is the main point of the paper (comments # 6-10).

IV. Imprecisions on the approach that need clarification, issues with text and figures. (comments #11-28).

Detailed comments and suggestions on how to address them are provided below. The two reviewers that contributed to this review believe the authors can improve the justification of their approach, as well as the interpretation and generalization so the manuscript can be acceptable for publication.

Response: We thank the reviewer for the thoughtful and constructive suggestions. We find that the paper is significantly strengthened by addressing the four issues you are concerned with. We have responded to these questions and comments point-by-point below.

1. Gaps in context and references. The sentence of L71 is not true: “In this study, for the first time, we show the capability of using BGC-Argo floats equipped with optical sensors to estimate the carbon export flux and associated vertical attenuation in response to submesoscale fronts”

The paper is lacking key references, in particular prior studies identifying and quantifying the role of subduction by fronts, including with BGC-Argo floats. The manuscript leads us to believe that there were no attempts to quantify this subduction pump before. Yet observation based (using float AOU, spiciness) and high resolution modeling papers have been published on this topic (see list of reference below). They

need to be included to provide the adequate context in the introduction but also in the discussion. Note that the process of export by subduction along fronts explained in this study can be called differently in the literature: eddy pump, eddy subduction pump etc. Although the term “eddy pump” was often used, it does not usually refer to “Ekman pumping” in eddies but the subduction by fast velocities associated with meso and submesoscale fronts, similarly to what is considered in this manuscript.

Sentences that critically need these references include L56-67 (almost all sentences in this paragraph are missing critical references), L71 which needs to integrate these prior work. In discussion, please consider how the impact of subduction would influence your results and discuss considering these prior works.

Review about export pumps including the physical injection pump or eddy pump:
Boyd, P.W., Claustre, H., Levy, M., Siegel, D.A., Weber, T., 2019. Multi-faceted particle pumps drive carbon sequestration in the ocean. Nature 568, 327. https://doi.org/10.1038/s41586-019-1098-2

Papers estimating the role the “eddy subduction pump” using BGC-Argo, cruises and models:
Erickson, Z.K., Thompson, A.F., 2018. The Seasonality of Physically Driven Export at Submesoscales in the Northeast Atlantic Ocean. Global Biogeochemical Cycles 32. https://doi.org/10.1029/2018GB005927
Lacour, L., Llort, J., Briggs, N., Strutton, P.G., Boyd, P.W., 2023. Seasonality of downward carbon export in the Pacific Southern Ocean revealed by multi-year robotic observations. Nat Commun 14, 1278. https://doi.org/10.1038/s41467-023-36954-7
Llort, J., Langlais C., Matear R., Moreau S., Lenton A., Strutton Peter G., 2018. Evaluating Southern Ocean Carbon Eddy-Pump From Biogeochemical-Argo Floats. Journal of Geophysical Research: Oceans 123, 971–984. https://doi.org/10.1002/2017JC012861
McGillicuddy, D.J., Resplandy, L., Lévy, M., 2019. Estimating particle export flux from satellite observations: Challenges associated with spatial and temporal decoupling of production and export. Journal of Marine Research 77, 247–258. https://doi.org/10.1357/002224019828474331
Omand, M.M., D’Asaro, E.A., Lee, C.M., Perry, M.J., Briggs, N., Cetinić, I., Mahadevan, A., 2015. Eddy-driven subduction exports particulate organic carbon from the spring bloom. Science 348, 222–225. https://doi.org/10.1126/science.1260062
Resplandy, L., Lévy, M., McGillicuddy Jr., D.J., 2019. Effects of Eddy-Driven Subduction on Ocean Biological Carbon Pump. Global Biogeochemical Cycles 33, 1071–1084. https://doi.org/10.1029/2018GB006125
Stukel, M.R., Song, H., Goericke, R., Miller, A.J., 2017. The role of subduction and gravitational sinking in particle export, carbon sequestration, and the remineralization length scale in the California Current Ecosystem: Subduction and sinking particle export in the CCE. Limnology and Oceanography 63, 363–383. https://doi.org/10.1002/lno.10636

Response: Thank you for pointing this out. We agree that prior works on subduction by fronts should be included in the introduction, and its impacts on our results should be discussed, as subduction by fronts is one of the most important dynamics by which submesoscale processes affect local POC distributions. We deleted that inaccurate statement in this revision. The changes in the introduction and discussion sections are summarized below:

1. Changes in the introduction section

To provide a more adequate context in the introduction that the submesoscale process affects local POC export, we first added one sentence in Lines 57-60 to mention the eddy-subduction pump:
'*The vertical motions can inject high POC produced in the euphotic zone into the mesopelagic layer (referred to as eddy subduction pumps)*[17,19,22,24]*, resulting in long-term carbon sequestration*[17,19,20,23]*.*'

Second, prior works that attempted to identify and quantify eddy subduction pumps have been acknowledged in Lines 68-71:
'Prior studies have captured anomalous features of elevated POC at depth, which are interpreted as evidence of eddy subduction pump[19,22,23]*. Despite their potential importance in shaping POC export, direct observations of the downward POC flux and its attenuation in response to submesoscale fronts are scarce*[17,19-21,23,41]*.'*
and Lines 76-81 :
'Observational and high-resolution modeling studies have suggested that the eddy subduction pump contributes ~19% to 50% to the total export of POC. In contrast, Resplandy et al. (2019) explored the integrated effects of eddy subduction hot spots on the biological carbon pump and found that they contribute less than 5% to carbon export due to the strong compensation between upward and downward fluxes.'

Additionally, key references have been added in Lines 71 (*Omand et al., 2015; Llort et al., 2018; Erickson et al., 2018; Lacour et al., 2023; Boyd et al., 2019*) and Line 86 (*Llort et al., 2018; Lacour et al., 2023*).

Finally, we acknowledge that Llort et al. (2018) was the first to quantify the carbon export flux associated with submesoscale fronts by using BGC-Argo. Previous studies that quantified the carbon export flux at submesoscale using BGC-Argo have also been cited in this revision. In comparison, however, these previous studies didn't consider the vertical attenuation of the carbon flux at submesoscale fronts, which is the main contribution of our study. To integrate the previous efforts and clarify the novelty of our results, we have rephrased the sentence as shown below (Lines 91-96):
'The BGC-Argo has been used to identify elevated POC in the mesopelagic layer and estimate the carbon flux associated with submesoscale features[20,23]*. In this study, we further show the capability of using BGC-Argo floats to estimate the transfer efficiency*

of carbon export in response to submesoscale fronts.'

2. Discussion on the impacts of subduction on POC export

We agree with comments #8 and #9 that the impacts of subduction by front should be taken into account to interpret our results. More discussion has been added in this revision.

As you mentioned in comment #9, the high POC at a depth of 200 m at the edge of CE 1 could be due to subduction of POC from the productive layer by the front. After a detailed examination, we agree with this comment and have added additional text (Lines 376-382) to discuss this point. Please see our response to this comment #9.

On the other hand, we found that the influence depth of eddy subduction may not be deep enough to affect the lower mesopelagic layer. The elevated POC concentration and flux were only present at depths between Z_{Eu} and ~250 m. We tested different ways to fit the POC flux attenuation coefficient (*b*) with and without considering the POC flux in the subduction layer (Z_{Eu}~250 m). The *b* value was about 0.65 excluding the POC flux in the eddy subduction layer, which was higher than that considering the subduction layer (*b*=0.56), but was still lower than those estimated in eddies and normal conditions. This suggests that submesoscale dynamics affect POC export flux not only through physical subduction but also through modulating particle properties (e.g., size, sinking speed and composition). We have added a paragraph (Lines 373-393) to fully discuss the impact of eddy subduction on our results. Please find our detailed response to comment #8.

References:

Boyd, P.W., Claustre, H., Levy, M. et al. Multi-faceted particle pumps drive carbon sequestration in the ocean. *Nature* **568**, 327–335 (2019).

Resplandy, L., Lévy, M. & Mcgillicuddy, D. J. Effects of eddy-driven subduction on ocean biological carbon pump. *Glob. Biogeochem. Cycles* **33**, 1071–1084 (2019).

Omand, M. M. et al. Eddy-driven subduction exports particulate organic carbon from the spring bloom. *Science* **348**, 222–225 (2015).

Lacour, L., Llort, J., Briggs, N. et al. Seasonality of downward carbon export in the Pacific Southern Ocean revealed by multi-year robotic observations. *Nat. Commun.* **14**, 1278 (2023).

Erickson, Z. K. & Thompson, A. F. The seasonality of physically driven export at submesoscales in the Northeast Atlantic Ocean. *Global Biogeochem. Cycles* **32**, 1144–1162 (2018).

Stukel, M. R., Song, H., Goericke, R. & Miller, A. J. The role of subduction and gravitational sinking in particle export, carbon sequestration, and the remineralization length scale in the California Current Ecosystem. *Limnol. Oceanogr.* **63**, 363–383 (2018).

Llort, J. et al. Evaluating Southern Ocean carbon eddy-pump from biogeochemical-

argo floats. *J. Geophys. Res. Ocean.* **123**, 971–984 (2018).
McGillicuddy, D. J. et al. Estimating particle export flux from satellite observations: Challenges associated with spatial and temporal decoupling of production and export. *J. Mar. Res.* **77**, 247–258 (2019).
Lévy, M. et al. The Impact of Fine-Scale Currents on Biogeochemical Cycles in a Changing Ocean. *Annu. Rev. Mar. Sci.* **16**,1 (2024).

2. Missing justification of the hypotheses, specifically the justification to use a "mostly spatial" or a "mostly temporal" framework to interpret the float data.
The authors alternatively interpret the observed changes along the float trajectories as "mostly spatial changes" (CE1 and ACE1, Figure 2), or "mostly temporal changes" i.e. pseudo lagrangian (CE2 and front, Figure 3). The hypothesis of "mostly temporal" is justified in the text for CE2 (L128 and 141). However, the justification to interpret as "mostly spatial" the changes along CE1 and ACE1 is missing, and the hypothesis to interpret as "mostly temporal" the changes attributed to frontal dynamics seems inadequate (see details in comments 3 and 4 below). We encourage the authors to explain clearly and early these different strategies to orient and prepare the reader.

Response: Thank you for pointing this out. In this study, we performed different analyses based on different sampling scenarios of the floats to delineate temporal and spatial variabilities of the measured variable. For the hypothesis of 'most spatial changes' in eddies (CE1 and ACE1), we first showed a radial increase of the relative distance between the float and the eddy center with time. Then, we demonstrated that the change of surface Chl measured by the floats was consistent with the radial variation of remote-sensed surface Chl from the eddy core to the eddy edge. In addition, with remote sensing data, we can track the temporal evolution of Chl in eddies, and this temporal pattern was completely different from the change of float-measured surface Chl. Therefore, for this sampling period, we think the float-measured variability of POC can be used to represent the spatial variability of POC from the eddy core to the eddy edge.

For the hypothesis of 'most temporal changes' along a submesoscale front, we added more analysis about the float-measured characteristics of the water masses. We also conducted a numerical particle tracking experiment. These new results all suggest that the float continuously drifted along the warm side of the front on which homogeneous water masses were sampled. During this period, the front experienced intensifying and decaying stages, while the float was still sampling along its warm side. As such, the float observations at the front can be interpreted as a temporal signal due to the stage change of the front.

Following the suggestions, we now justify these two hypotheses early in Sections 2.1 and 2.3, respectively. Please see more details in our following responses to comments #3 and #4.

3. The authors need to demonstrate why it is justified to ignore temporal variations when analyzing CE1 and ACE1. Figure 2 interprets all POC data as spatial changes (using radial structure of the eddies CE1 and ACE1). The approach is attractive, but the text and figures should clearly show that this is a valid hypothesis. Specifically, how much of the signal that is interpreted as spatial variability across the eddy is in fact introduced by the temporal evolution along the trajectory of the float? At least, the authors could indicate the day of sampling for each data point on Figure 2? [Colors could indicate the day of sampling while symbols could indicate the depth]. This would allow the reader to see how much of the variations attributed to the spatial structure of the eddy could be introduced by temporal changes.

Response: Thank you for the constructive comment. For CE 1 and ACE 1, we interpreted the measured variability of POC as the radial structures of eddies based on the following considerations:

Firstly, there was an increasing relative distance between the float and the eddy center with time. The location of the float changed from inside the eddy to outside the eddy, with a relative distance of r/R between 0.4~2.0 for CE 1 and 0.23~1.2 for ACE 1.

Secondly, the spatial distribution of Chl in eddies was derived by compositing the remote-sensed sea surface Chl over the float sampling period. This satellite-derived Chl was then interpolated onto the relative locations of the float in eddies to compare with that measured by the float. In CE 1, both the satellite- and float-derived Chl decreased and then increased in the radial direction, reaching a peak at r/R=1~1.5 (Fig. S2). In ACE 1, both the satellite- and float-derived Chl showed a double peak pattern along the radial direction (Fig. S2a). The correlations between the satellite- and float-derived Chl were 0.39 (p=0.01) and 0.43 (p=0.09) for CE1 and ACE 1, respectively. The relatively consistent changing patterns of Chl between the float and remote sensing data suggest that the float captured the spatial variability of the eddies in the radial direction.

Lastly, it is likely that the temporal signal was also included in the float data as it exhibited larger 95% confidence intervals than those derived from satellite observations (Fig. S2a). With remote sensing data, we can track the temporal evolution of the Chl in eddies, and this temporal pattern was completely different from the change of float-measured surface Chl (Fig. S2b). The correlations between the satellite- and float-derived Chl over time were -0.18 (p=0.24) and -0.05 (p=0.82) for CE1 and ACE 1, respectively, suggesting that the variability explained by the temporal evolution of Chl of eddies was much small. Therefore, for this sampling period, we think the float-measured variability of variables can be used to represent the spatial variability of eddies.

To clarify this point, we added the following sentences in Lines 143-154 and Fig. S2 before we presented the radial POC distributions in the text:
‘The floats may capture the spatial variability of tracers for CE 1 and ACE 1 as the

location of the floats changed from inside the eddies to outside the eddies. The satellite-derived sea surface Chl was averaged over the float sampling period and then interpolated onto the relative locations of the float in eddies to compare with that measured by the float. The float-measured Chl was in good agreement with that derived from satellites in the radial direction (Fig. S2a), with correlations of 0.39 (p=0.01) for CE1 and 0.43 (p=0.09) for ACE 1. In contrast, the temporal evolution of satellite-derived Chl averaged inside eddies was completely different from that of float-measured Chl (Fig. S2b), indicated by low correlations (R= -0.18 (p=0.24) for CE 1 and R=-0.05 (p=0.82) for ACE 1). Therefore, the float-measured tracers during this period can be used to represent the spatial variability of the eddies along the radial direction.'

Another thing worth mentioning is that the high POC distribution at the edge of CE 1 was due to subduction by the front (please see our reply to comment #9). This further strengthens the point that the distributions of POC captured by the float reflect the spatial variability of the eddy. We have added the following sentence in Lines 169-170 to emphasize this point:
'This further supports that the radial distributions of POC measured by the float reflect the spatial variability of the eddy.'

Following the reviewer's suggestion, colors have been indicated in Fig. 2. For CE 1, as the float moved almost monotonically from the eddy core to the edge with time, it is still not easy to tell the influence of the temporal signal from this figure. Figure R1 shows the variations of the strength of the eddies. It is clear that although CE 1 was strengthened in early May while the float was at r/R=~1.3, the float-measured POC at SCM layer was still lower than that measured at the eddy core (Fig. 3), implying dominant variability caused by space instead of time.

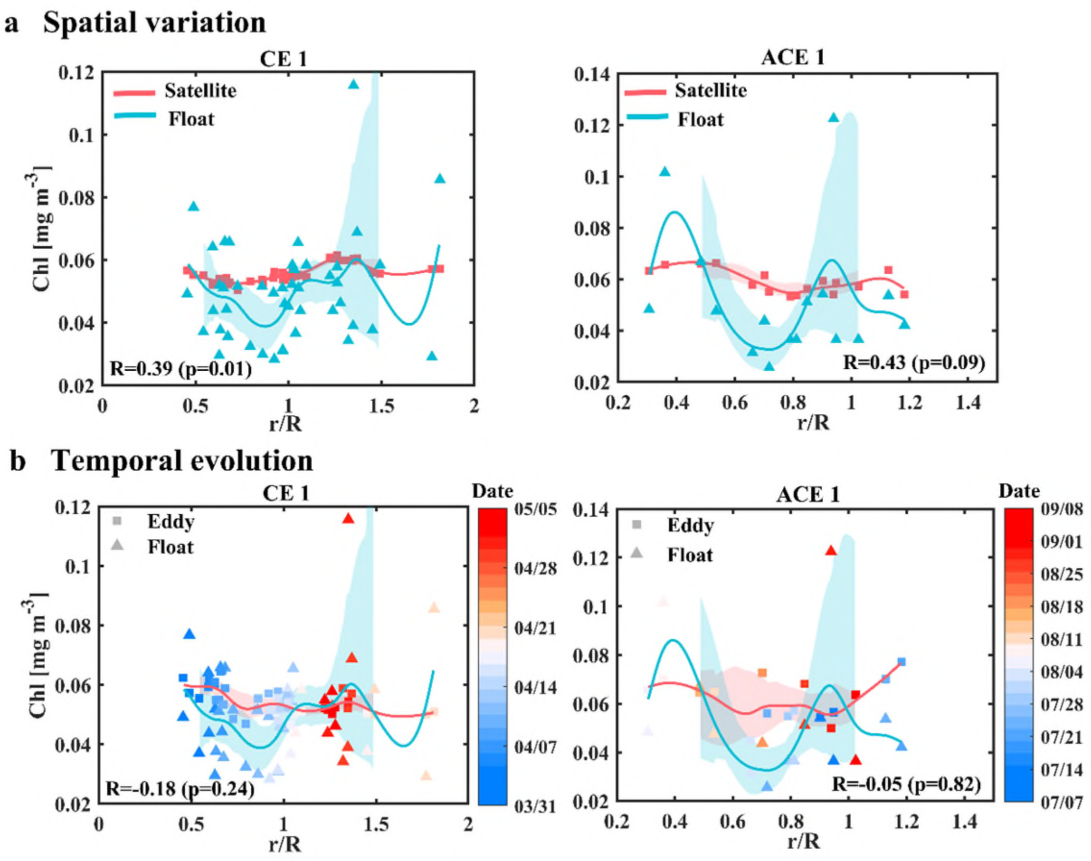

Figure S2. Comparison of sea surface Chl between satellite and BGC-Argo observations in both spatial and temporal variability. **a** Radial distribution of sea surface Chl at the float locations derived from both the BGC-Argo and satellite observations. The BGC-Argo results are directly obtained from the surface Chl measured by the float. The satellite-derived Chl values are calculated by averaging the Chl from OC-CCI over the float sampling period and then interpolating to the relative locations of the float in eddies. Because the eddies propagated over time, the location of the float in the eddies was constructed by calculating the azimuthal and radial positions of the float relative to the eddy center. **b** Satellite-derived Chl averaged inside eddies and sea surface Chl derived from BGC-Argo observations. The BGC-Argo results are directly obtained from the surface Chl measured by the float. The satellite-derived Chl values are calculated by averaging the satellite-derived Chl values inside the eddies at the time when the float was at the position of r/R.

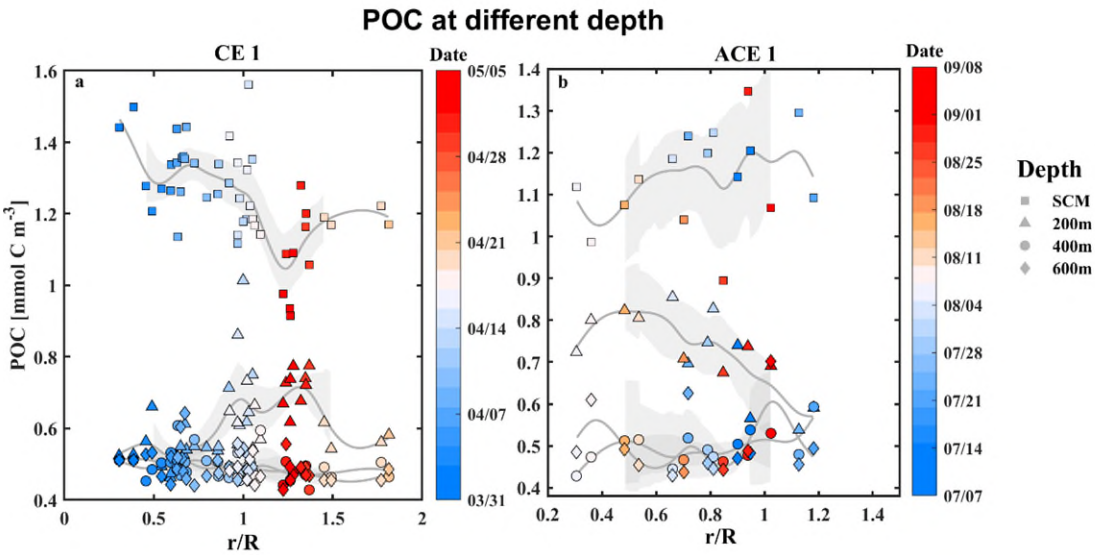

Figure 3. POC distributions at different depths within **a** CE 1 and **b** ACE 1.

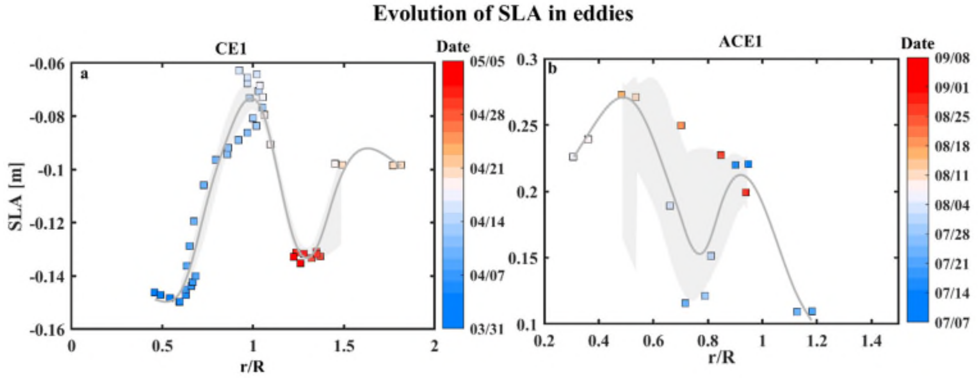

Figure R1. Evolution of SLA averaged inside eddies. The SLA values are calculated by averaging the SLA inside the eddies at the time when the float was at the position of r/R.

4. The hypothesis of "mostly temporal" is highly disputable for the front. It is ok for eddy CE2 because the float seems to stay stuck close to the center of the eddy, and most changes can be interpreted as temporal changes. In the case of the front, however, the float seems to be sampling different parts of the front. The associated changes in SLA, POC, AOU etc. are therefore likely to come from spatial variations across the front and not temporal variations. The authors could demonstrate that it is indeed temporal, however it is unlikely to be successful and convincing given the complexity of the dynamics in the frontal structure. This shortcoming has major implications for the result interpretation and the conclusions of the paper as the authors interpret these changes as temporal changes while they are likely spatial changes. This section of the results and associated conclusions needs to be revised.

Response: Thank you for the insightful comment. According to the frontogenesis

theory, when the front is enhanced, it will generate downwelling on the cold side of the front and upwelling on the warm side of the front. The hypothesis of ‘mostly temporal’ is that the float drifted along one side of the front so that relatively homogeneous water masses over time were sampled. During this period, the front experienced intensifying and decaying stages, while the float was still sampling along its one side. As such, the float observations at the front can be interpreted as a temporal signal due to the stage change of the front. We have provided more justifications in the revised text to interpret this hypothesis.

By investigating the time series of FSLE along the float trajectory, we found that the float traveled along the same filament with high FSLEs. This result, in combination with the Chl, SST and SST gradient distributions (already shown), demonstrated that the float continuously traveled along the warm side of a front during that period.

To further support that the float drifted along one front, a numerical particle tracking experiment was conducted at the frontal region using the TRACMASS code. The particle was released at the same location as the float on 21 April 2019 and it was moved purely by advection using the surface geostrophic current derived from AVISO data. The numerical tracking results showed that the float mainly followed the path of the particle (Fig. S14), suggesting that the float moved in a quasi-Lagrangian manner and sampled a similar water mass during the analysis period (supplementary information Text S3).

The homogeneous water masses sampled on one side of the front can be supported by the transect of temperature and salinity anomalies (Fig. S6). The temperature was higher in the upper 300 m and the salinity was lower in the upper 150 m depth compared to the CE 1 core. In addition, the T-S diagram presents a coherent physical characteristic from 21 April to 3 May 2019, suggesting that the float was moving along the front but not across the front.

Overall, these results support the use of the float data as a time series on the warm side of the front during the analysis period.

We have clarified this point in the text in Lines 247-250:
‘This is further supported by a Lagrangian particle tracking experiment, which suggests that the float drifted in a quasi-Lagrangian manner and sampled a similar water mass along one side of the front (Text S3).’
, and Lines 250-255:
‘The front exhibited a higher temperature in the upper 300 m and lower salinity in the upper 150 m compared to the CE 1 core (Fig. S6), suggesting homogeneous water masses sampled at the frontal region. The T-S diagram demonstrates a coherent physical characteristic from 21 April to 3 May 2019 (Fig. S6). All these results suggest that the float sampled homogeneous water masses along the warm side of the front.’

Text S3. Lagrangian particle tracking experiment on the front

To investigate whether the float drifted along the front, a numerical particle tracking experiment was conducted using the TRACMASS Lagrangian trajectory code (Döös, 1995; Blanke & Raynaud, 1997). The particle was released at the same location as the float on 21 April 2019 and transported purely by surface current. Here, we run the code with the current velocity derived from AVISO. The Lagrangian particle trajectory derived from the particle tracking model demonstrated that the virtual particle starting at the location of the float moved along the front (Fig S14). The float moved slower than the particle but mainly followed its path, suggesting that the float drifted in a quasi-Lagrangian manner during the analysis period. The difference in moving velocity is that the particle was only transported by surface current, whereas the motion of the float was driven by both the surface and deep currents.

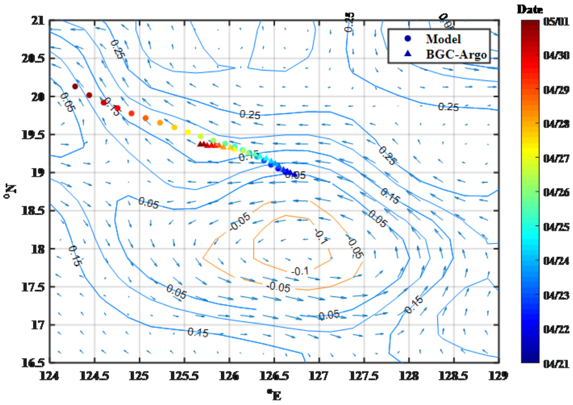

Figure S14. Trajectories of the BGC-Argo and the virtual particle over the period when the float was on the front (21 April - 3 May 2019). The particle was released at the same position as the float on 21 April 2019 and transported by the surface current velocity derived from AVISO. The particle trajectory was tracked using the TRACMASS code. Color indicates the dates. Blue and red lines are the contour lines of the sea level anomaly (SLA). Arrows represent the sea surface current velocity anomaly.

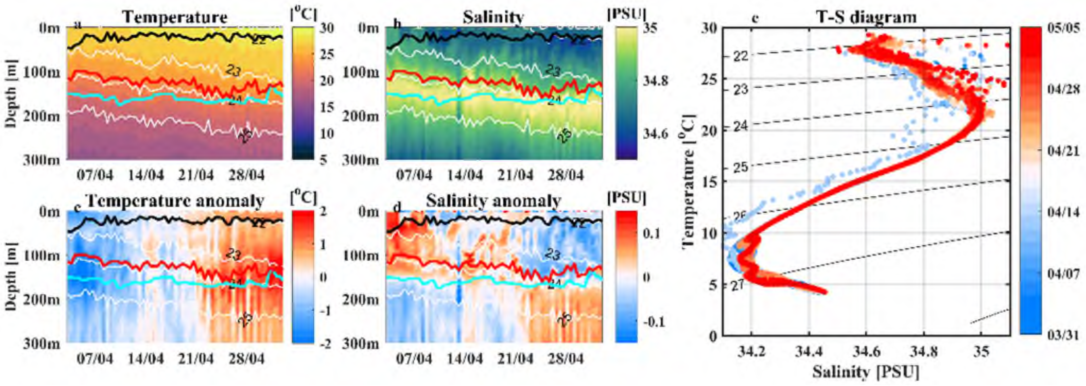

Figure S6. Physical characteristics of the float measurements. During 21 April - 3 May 2019, the float was on one side of the front. Time series of **a** temperature, **b** salinity, **c** temperature anomaly and **d** salinity anomaly measured by float 2902753 from 1 April

to 3 May 2019. The black, red and cyan lines indicate the mixed layer depth (MLD), the depth of subsurface chlorophyll maximum (Z_{SCM}) and the euphotic zone (Z_{Eu}), respectively. The white lines are the isopycnals of 23, 24 and 25 kg m^{-3}. The temperature and salinity anomalies were defined as the instantaneous values after subtracting the full time mean. **e** Temperature-salinity diagram with data points from float 2902753 from 1 April to 3 May 2019. The color indicates the sampling date.

5. The sampling context should be better presented. In Figure 1, eddies and float trajectories appear completely disconnected. This is likely because the black lines show the center of the eddy and not their span. Could you indicate the radius of the eddies around the trajectories so we can see that they were indeed in the eddies? Also we encourage the authors to include a figure similar to Figure S4a to show the float relative to the eddy/front. Maybe choose snapshots that show the overlap with the front as a function of time.

Response: Thank you for this suggestion. The gray lines indicating the eddy radius have been overlapped in Fig. 1a to show the connection of the eddy centers and the floats. Additionally, snapshots showing the locations of the floats relative to the eddies and the front have been included in the new Figure 2.

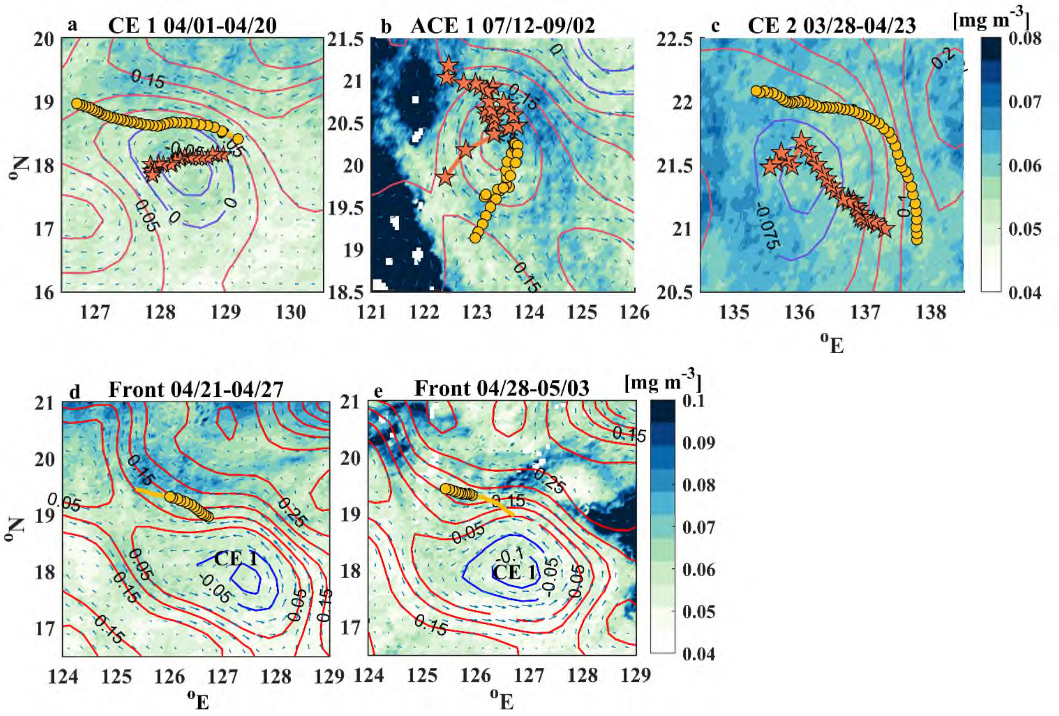

Figure 2. The BGC-Argo and eddy trajectories. **a** The locations of (yellow dots) the BGC-Argo float (WMO number 2902753) and (orange pentagrams) the center of cyclonic eddy 1 (CE 1) during 1-20 April 2019. **b** The locations of (yellow dots) the BGC-Argo float (WMO number 2902753) and (orange pentagrams) the center of anticyclonic eddy 1 (ACE 1) during 12 July - 2 September 2019. **c** The locations of (yellow dots) the BGC-Argo float (WMO number 2902756) and (orange pentagrams) the center of cyclonic eddy 2 (CE 2). **d** The locations of the BGC-Argo floats along the front during 21-27 April 2019. The yellow line is the float trajectory from 21 April to 3

May 2019. **e** The same as d but during 28 April-3 May 2019. Background color and isolines represent sea surface chlorophyll and sea level anomalies, respectively. The arrows are the geostrophic velocity anomaly.

6. Generalization between front and carbon export. L214-222. "A strong correlation between the b value and GM was found …. This statistical relationship implies that a strong front is linked with high POC transfer efficiency to the mesopelagic layer, which could be used as a parameterization to account for the impact of fronts on carbon export in large-scale models, once the front intensity at the surface is known". This conclusion seems not backed up by the results. More complete models seem to show that b can vary according to many other biophysical parameters, making this generalization highly debatable (see for instance Ngyuen et al. 2022: Microbes contribute to setting the ocean carbon flux by altering the fate of sinking particulates). This is one of the main points of the paper; the caveats need to be discussed properly.

Response: Thank you for pointing this out. We agree that a parametrization of the *b* value as a function of the front intensity is inadequate since the efficiency of POC transfer is modulated by complicated physical and biogeochemical processes. We have rephrased the sentence as follows to be clearer:
'This statistical relationship implies that a strong front is linked with high POC transfer efficiency to the mesopelagic layer, which could be used to estimate the impact of fronts on carbon export at the basin-scale oceans.'

We used this relationship to estimate the impacts of fronts on POC transfer efficiency. The underlying mechanism is that submesoscale dynamics play a role through their impact on the particle sinking velocity or particle lability. The following discussion has been added to the manuscript (Lines 405-413):
'The estimation suggests that submesoscale fronts are important in setting the spatial and temporal variations in POC transfer efficiency. Previous carbon cycle studies have suggested that the attenuation of the POC flux is determined by the rate of POC production in the euphotic zone, the sinking velocity of particles, and the rate of POC consumption in the mesopelagic layer[5-10,38,39,49-54]. Submesoscale dynamics may play a role in POC flux transfer through eddy subduction that directly enhances the particle sinking velocity or through its impacts on the plankton community structure in the euphotic zone that generates particles with large sinking velocities and/or low remineralization rates[9,14-23,32].'

The caveat of the estimation conducted in this study is that we did not explicitly include other physical and biogeochemical processes that affect POC flux attenuation. These processes include mixed layer pumping, bacterial colonization and consumption, zooplankton migration, consumption, and fragmentation. However, there are no clear data or previous studies showing why these processes could be particularly enhanced or weakened at fronts. We provide some discussions here. We think inclusion of these processes may allow a better constraint in estimating POC transfer efficiency, but it

definitely requires future work to examine how submesoscale dynamics affect these processes. To clarify this point, we have added the following sentences to the text (Lines 413-425):

'Our estimation suggests that submesoscale fronts increase the POC transport efficiency in the NPSG. However, it should be kept in mind that other processes that can potentially modulate the b value (e.g., microbial POC consumption[49,54], POC fragmentation by zooplankton[8]) were not explicitly included in the estimation. Quantifying the mesopelagic fragmentation processes revealed that roughly half of the observed flux attenuation was due to the fragmentation of large particles into small particles[8]. Ngyuen et al. showed that microbial dynamics may play a significant role in shaping the POC flux profiles observed in global oceans[54]. How these dynamics respond to submesoscale fronts, however, is still not clear. Future work including dynamics such as microbial consumption and zooplankton disaggregation of the particles is needed, and may allow for better constraints on the relative contributions of submesoscale fronts to the basin-scale carbon transport efficiency.'

7. Conclusion about seasonality and export. line 234: You did not take into account that the seasonality of mesoscale and frontal dynamics is different from the seasonality of POC content in the water column. Consequently, the amount of POC transported by the same front strength varies depending on the season. This could completely reverse your conclusion about the seasonal evolution of the parameter b. Please revise or strengthen these points.

Response: Thank you for pointing this out. The relationship between the *b* value and submesoscale dynamics was derived from multiple observations in subtropical gyres, which cover both winter and summer months. This suggests that submesoscale dynamics can significantly affect POC transport efficiency in different seasons. As the POC transport efficiency is largely determined by the *b* value, POC concentration usually won't affect the transport efficiency. Instead, the properties of the POC (e.g., size, lability) are important in changing the *b* value. Submesoscale dynamics have been suggested to affect the POC transfer efficiency by stimulating the growth of large plankton, which in turn generate large and rapidly sinking particles at the front. Regarding the same POC concentration with slow sinking in the background and with fast sinking at the front, the POC transfer efficiency will be higher at the front than in the background. Therefore, to derive a robust *b* value function, the properties of POC should also be considered. However, there are few POC property data available for now.

We examined the dataset from Station ALOHA, and found that the relationship between the *b* value and POC concentration was not clear at the seasonal timescale. At ALOHA, long-term records of the POC flux profiles suggest that the POC transfer efficiency is higher in winter than in summer (Fig. S16). On the other hand, the POC concentrations are generally higher in summer than in winter, which is indicated by the averaged profiles of POC at ALOHA (Fig. R2).

Overall, the result of a lower *b* value in winter than in summer derived from the relationships of both b_T and b_{GM} is in good agreement with the in situ result at ALOHA (Fig. S16), suggesting that the conclusion is likely robust. To support our results, the observational results of the seasonality of the *b* value (Fig. S16) have been included in the text.

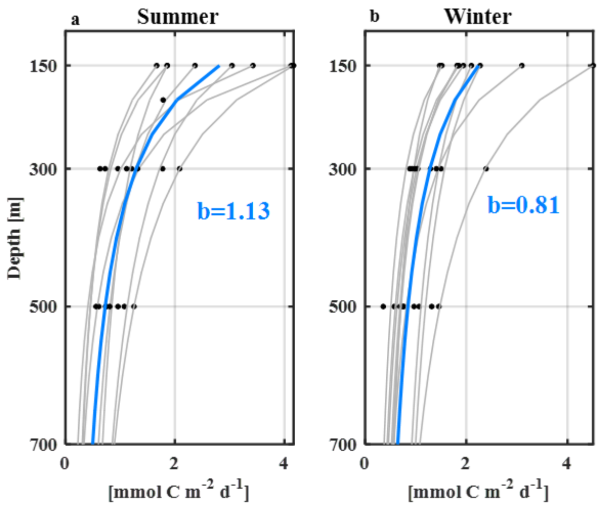

c

Summer	*b*	R^2	winter	*b*	R^2
1	1.08	0.93	1	0.93	0.98
2	1	1	2	0.93	1
3	0.83	1	3	0.94	1
4	1.63	0.99	4	0.61	0.96
5	0.66	0.95	5	0.81	1
6	1.17	0.98	6	0.91	0.93
7	0.89	0.96	7	0.53	0.99
8	1.77	0.94	8	1.11	0.98
			9	0.54	0.93
			10	0.78	1
Mean *b*	1.13			0.81	

Figure S16. The seasonal *b* value at the ALOHA station. **a** POC flux profiles and the corresponding Martin curves in summer. Black dots are observational profiles from sediment traps. Gray lines are the fitted Martin curves. The blue line is the Martin curve derived from the averaged *b* value and averaged POC flux at 150 m. **b** is the same as **a** but in winter. **c** The *b* value and R^2 for each observational POC flux profile in both summer and winter. We picked up all observational POC flux profiles with at least three vertical sampling points for Martin curve fitting. The *b* values in summer are significantly different from those in winter, as h=1 and p=0.036 in the two-sample t test result.

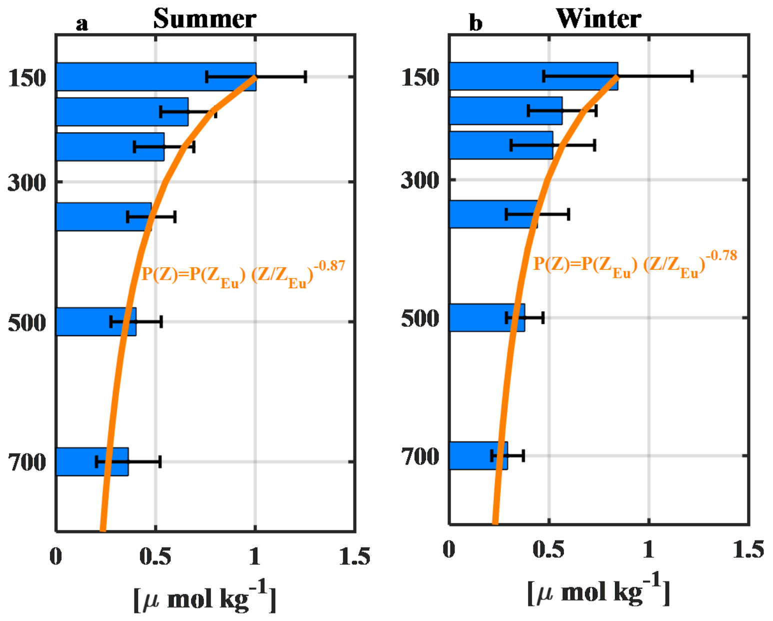

Figure R2. The seasonally averaged POC profiles at ALOHA. **a** The vertical

distributions of POC concentrations in summer averaged over 1998-2021. The bars show the averaged POC at depths of 150 m, 200 m, 250 m, 350 m, 500 m and 750 m. The orange line is the fitted attenuation curve of the averaged POC concentrations with depth. Error bars show one standard deviation for the averaging. **b** The same as **a** but in winter.

8. Missing consideration of subduction #1. The POC export depth by fronts also depends on the penetration depth of the vertical velocities associated with the fronts (subduction, see comment #1 for a list of papers that describe this). Shallow fronts do not allow POC to be exported below the pycnocline and would limit the frontal effect on b, further weakening your conclusion and the link established between fronts and POC export/attenuation coefficient. Please consider this effect of subduction in introduction and importantly in your results and interpretation. This is one of the weakest point of the paper. See also comment 9 below.

Response: Thank you for the constructive suggestion. In this study, we consider two mechanisms affecting POC export at fronts, which have been shown to be important in other regions in previous studies. The first mechanism is through biological carbon pump. Strong submesoscale flows can enhance the vertical nutrient supply, alter the phytoplankton community structure, and generate particles sinking to depth. Some of the particles generated through this mechanism, if not remineralized quickly, have the potential to penetrate the pycnocline and reach the mesopelagic layer via gravitational sinking. The second mechanism is through subduction, by which particles are carried downward with the water mass. For this mechanism, the vertical depth the particle can reach is largely determined by the subduction velocity and the ventilation depth. Of course, these two mechanisms could coexist in some cases. Following the reviewers' comments, we added more discussion about the effect of subduction in this revised manuscript.

From our data, two lines of evidence revealed that eddy subduction contributes to POC export at the front. First, a higher POC concentration between Z_{Eu} (bottom of the euphotic zone) and ~250 m depth at the front than that in CE 1 was captured by the float, which is likely due to subduction of POC from the productive layer by the front (see details in Reply #9). Second, the POC fluxes between Z_{Eu} and ~250 m depth were highest in the vertical direction, which is different from the classical biological carbon pump curve and implies POC supply from other mechanisms (Fig. S10). As the high POC signal was associated with tilting isopycnals, it was very likely induced by subduction at the front.

However, subduction appeared limited to depths between Z_{Eu} and ~250 m (Figs. S9 and S10). We tested different ways to fit the POC flux attenuation coefficient (*b*) with and without considering the POC flux in the subduction layer (Z_{Eu}~250 m). The *b* value was approximately 0.65 excluding the POC flux in the subduction layer, which was higher than that considering the subduction layer (*b*=0.56) but was still lower than those

estimated in eddies and normal conditions. This suggests that submesoscale dynamics affect POC export flux not only through physical subduction but also through modulating particle properties (e.g., size, sinking speed, and composition). Our results emphasize the complexity of submesoscale dynamics at play in enhancing POC transfer efficiency.

As we mentioned in the response to comment #1, the introduction of the effect of the eddy subduction pump has been included in the introduction section. Additionally, we added one paragraph in the discussion section to interpret and fully discuss the impact of the eddy subduction pump on our results (Lines 373-393):
'The high transfer efficiency of POC at the front could also result from the physical subduction of POC by the intense submesoscale vertical velocities. Two lines of evidence have revealed that the eddy subduction pump contributes to carbon exports at the front. First, frontal dynamics are responsible for the elevated POC at 200 m depth at the CE 1 edge of r/R=1 and 1.3 (Fig. 2a). At the positions of r/R=1 and 1.3, the float was right on the filaments of high FSLE accompanied by high M^2 and small R_i in the vertical direction, which is suggestive of ongoing intense submesoscale vertical motions (Fig S5). Concurrence with the physical dynamics was an elevated POC concentration between Z_{Eu} and ~250 m compared to those in CE 1 (Fig. S9), which is evidence of eddy subduction pumps suggested in previous studies[17,19,20,23]. Second, the POC fluxes between Z_{Eu} and ~250 m influenced by the eddy subduction pump were obviously higher than those above Z_{Eu} and below 250 m (Fig. S10). We tested different ways to fit the POC flux attenuation coefficient (b) with and without considering the POC flux in the subduction layer (Z_{Eu}~250 m). The b value was approximately 0.65 excluding the POC flux in the subduction layer, which was higher than that considering the subduction layer (b=0.56) but was still lower than those estimated in eddies and normal conditions. This suggests that submesoscale dynamics affect POC export flux not only through physical subduction but also through modulating particle properties (e.g., size, sinking speed, and composition). These results emphasize the complexity of submesoscale dynamics at play in enhancing POC transfer efficiency.'

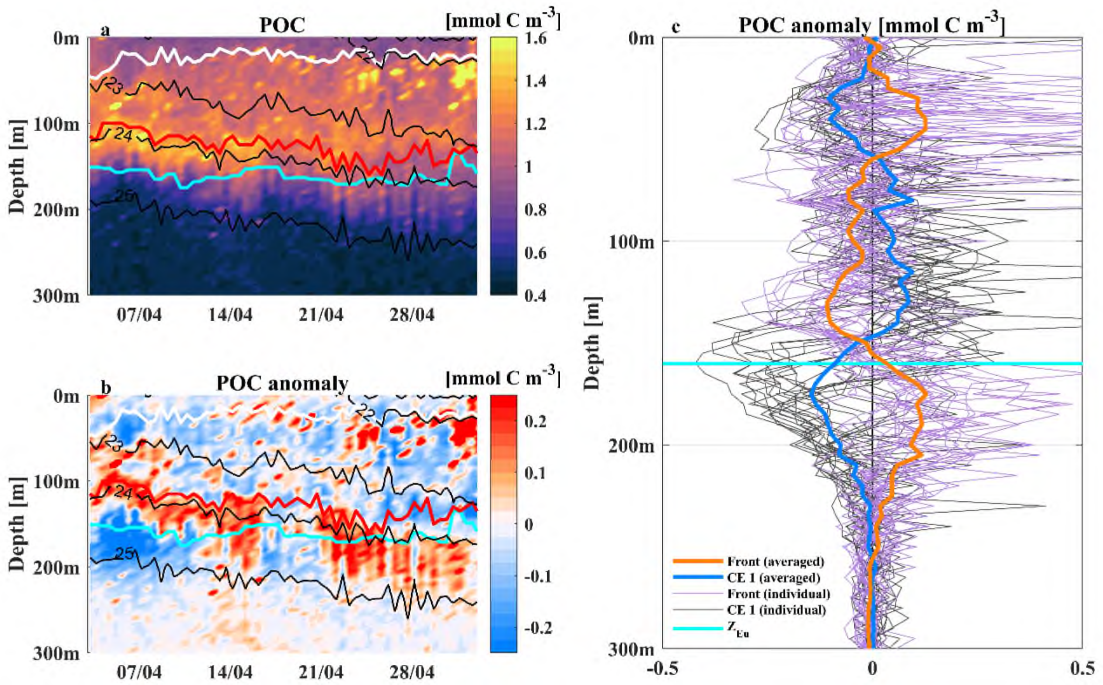

Figure S9. Vertical POC concentration distributions. **a-b** Time series of **a** POC distribution and **b** POC anomaly measured by float 2902753 from 3 April to 3 May 2019. The black, red and cyan lines indicate the mixed layer depth, depth of the subsurface chlorophyll maximum and euphotic zone, respectively. The POC anomalies were defined as the instantaneous values subtracting the full time mean. **c** Vertical distributions of the POC anomaly in CE 1 and at the front.

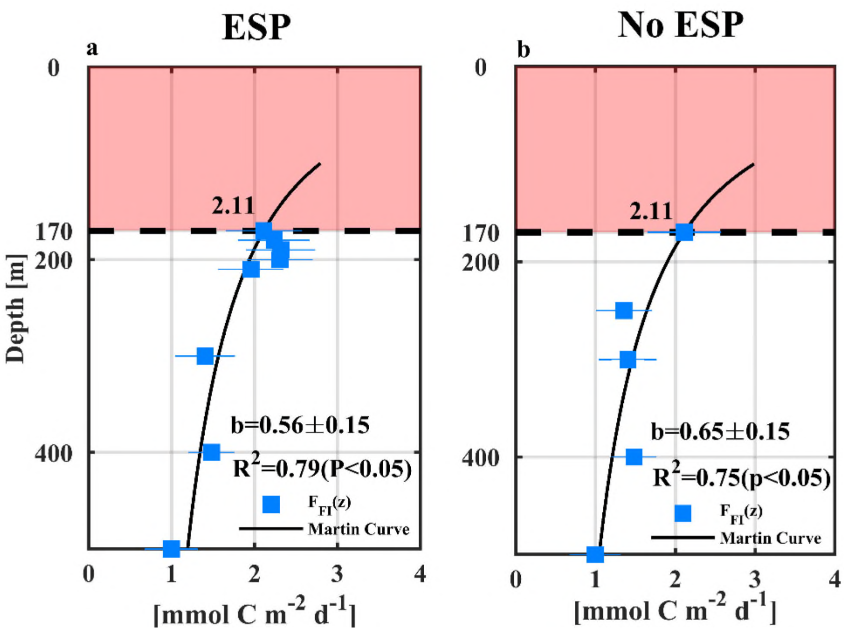

Figure S10. POC flux profiles. **a** The POC flux between Z_{Eu} and 250 m that is affected by eddy subduction pump (ESP) is accounted for when estimating the *b* value. **b** The ESP effect is not accounted for when estimating the *b* value. The black dashed lines and

error bars correspond to the euphotic zone depth and one standard deviation, respectively. The red areas represent the euphotic zone.

9. Missing consideration of subduction #2. L118: "The POC distributions attenuated faster in the CE 1 center than at its edge, which is likely due to the higher remineralization of the newly produced and easily degradable organic matter inside the eddy core8,36,37." Is this really what is happening here? Couldn't it be that intense vertical velocities associated with the frontal dynamics at the boundary of the eddies are transporting/subducting material down?

Response: As the reviewer suspected, the POC attenuated more slowly at the CE 1 edge than in its center because the subduction of POC by the front increased the subsurface POC at the edge. Specifically, the POC at 200 m presented a high value at r/R=~1 and 1.3, where the float was right on filaments undergoing intense submesoscale vertical motions. The proof to support this conclusion comes from the concurrence of high FSLE at the surface, large along-track M^2 and small R_i in the vertical direction (see Fig. S7). Meanwhile, the POC was higher between Z_{Eu} and ~250 m at the front compared to the eddy edge of other positions, likely due to subduction of POC from the productive layer by the front (Fig. S9). We have therefore rephrased this sentence as follows (Lines 165-169):
'The POC at 200 m reached its peaks at the edge of r/R=~1 and 1.3, which is likely due to subduction of POC from the productive layer by the front[17,19,20,23]'

More interpretations of the results have been added in the discussion section, specifically with the group of sentences shown below (Line 376-382):
'the frontal dynamics are responsible for the elevated POC at a depth of 200 m at the CE 1 edge of r/R=1 and 1.3 (Fig. 3a). At the positions of r/R=1 and 1.3, the float was right on the filaments of high FSLE accompanied by high M^2 and small R_i in the vertical direction, which is suggestive of ongoing intense submesoscale vertical motions (Fig S5). Concurrence with the physical dynamics was an elevated POC concentration between Z_{Eu} and ~250 m compared to those in CE 1 (Fig. S9), which is evidence of eddy subduction pump suggested in previous studies[17,19,20,23].'

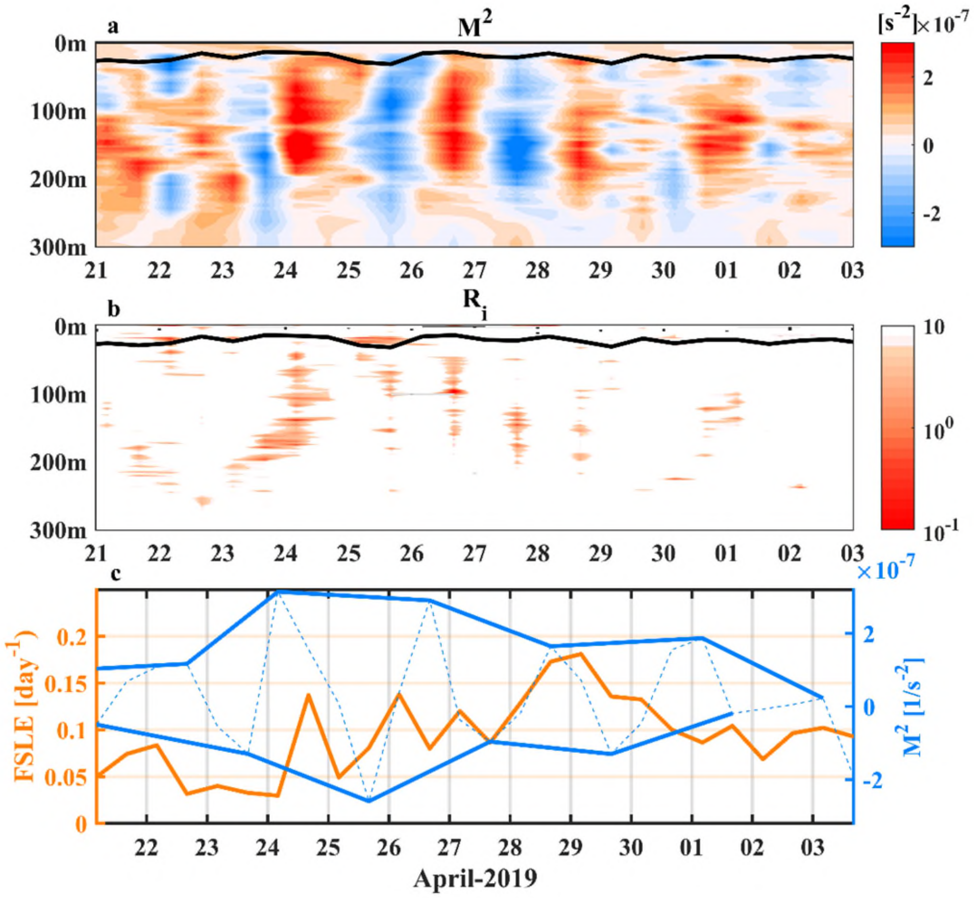

Figure S7. The lateral buoyancy frequency (M^2), Richardson number (R_i) and finite-size Lyapunov exponent (FSLE) at the front. **a** BGC-Argo-derived lateral buoyancy frequency during 21 April-3 May 2019. $M^2 = -\partial b/\partial s$, where s is the along-track direction. The black line represents the mixed layer depth. **b** BGC-Argo-derived Richardson number during 21 April-3 May 2019. $R_i = f^2 b_z / b_s^2$, where f and b are the Coriolis parameter and buoyancy, respectively. The black line represents the mixed layer depth. **c** Surface FSLE at the frontal region (red line) and the lateral buoyancy frequency (blue dashed line) at 100 m during 21 April-3 May 2019. Blue solid lines are the buoyancy frequency envelope.

10. Generalization to the subtropical Pacific. The generalization to the basin is very hand wavy and unconvincing, and probably not needed here. The results can stand alone without this. The author could in fact point out that the impact at the basin scale and global scale is still an open question. The conclusion, abstract (and maybe title) should be modified to reflect the changes in conclusions.

Response: Thank you for the helpful suggestion. We agree that the submesoscale impact at the basin scale is still an open question and have removed section 2.4 from the main text.

The generalization of our results to the Pacific gyre provides an applicable way to

estimate the effect of fronts on POC transfer efficiency based on satellite data, which definitely needs further studies to constrain uncertainties but also has the potential for future studies to explain POC variability in the mesopelagic ocean. In particular, we feel that the reviewers' comments #6 and #7 have been very helpful in improving this part. Based on these considerations, we modified our previous presentations and put this part as a discussion in the supplementary information. The title, abstract and conclusions have been modified accordingly. The details are listed below:

The title has been changed to '*Efficient biological carbon export to the mesopelagic ocean induced by submesoscale fronts*'.

In the abstract, the following sentences relative to the extrapolation results have been removed (Lines 27-31):
'Expanding the results to the subtropical gyre reveals a significant relationship between the Martin curve exponent coefficient (b) and front intensity. The average POC export flux at 1000 m may be underestimated by ~20-58% in the NPSG if the contribution of submesoscale fronts is ignored.'

For the previous section 2.4, the presentations have been modified. The section now has been moved to the supplementary information as Text S4.

In the discussion section, the following sentences have been added to link the observational results and Text S4 (Lines 396-405):
'The impact of fronts on POC transfer efficiency at the basin scale and global scale is still an open question. We combined previous studies that have estimated POC attenuation coefficients in the North Pacific and North Atlantic gyres (Table S4, Fig. S11). A strong correlation between the b value and GM was found (b_{GM}=-3.78·GM+1.44; R^2=0.52, p<0.01, Fig. 5). The derived relationship between the flux attenuation coefficient and the front intensity could be applied to large-scale applications. Estimations based on this relationship suggest that the average POC export flux at 1000 m may be underestimated by ~20-58% in the NPSG if the contribution of submesoscale fronts is ignored (Text S4).'

The following paragraph has been removed from the discussion section (Lines 330-341):

'The contribution of eddy intensification to the POC flux of the entire NPSG was estimated using eddy dataset. The intensifying cyclonic eddy occupied ~5.7% of the area of the NPSG (Fig. S7). This gives an additional POC flux of 0.12±0.03 mmolC m^{-2} d^{-1} by eddy intensifications in the NPSG in the euphotic zone. Considering the observed mean POC flux of 1.63 mmolC m^{-2} d^{-1} from previous studies in this region (Table S4), the eddy intensifications can increase the POC flux by ~7.4±1.8% in the NPSG. The contribution of front intensifications to the POC flux in the gyre is difficult to estimate, but an upper bound is attainable. Given that the fractional areas impacted by submesoscale structures are ~5.2% according to Liu et al.[42], the upper bound of the

POC flux that was increased by front intensification is 0.14±0.03 mmolC m^{-2} d^{-1}, which corresponds to ~9±1.9% of the POC flux at the base of the euphotic zone in the NPSG.'

In the Methods section, the following paragraph has been removed (Lines 561-568):
'Contribution of eddy intensification to the POC flux

The contribution of eddy intensification to the POC flux of the entire NPSG was estimated using the mesoscale eddy trajectory product from the Archiving, Validation and Interpretation of Satellite Oceanographic (AVISO) data. During 1993-2019, there were a total of 14539 cyclonic eddies, which, during the intensification stage, occupied ~5.7% of the area of the NPSG (Fig. S7). The POC export fluxes induced by eddy intensifications in the NPSG were calculated as $F_{EI}(Z_{Eu})\times A$, where A is the fractional area occupied by cyclonic eddies during the intensification stages.'

For figures, the original Figure 5 has been moved to the supplementary information, and the original Figure S7 has been removed.

Imprecisions on the approach that need clarification, issues with text and figures.
Section 2.1
11. Line 97 and onward. This paragraph about the position of the floats is a little tedious to read. Maybe the authors could either streamline or fold in some results in there as they describe the trajectory.

Response: Following the reviewer's suggestion, the descriptions of both the float position and float trajectory have been combined. In the new text, the paragraph you mentioned has been removed.

12. Line 107: "The euphotic depth (ZEu) was on average ~159 m within CE 1 and 161 m in ACE" is this difference really significant?

Response: We conducted a two-sample t test and found that the averaged Z_{Eu} for CE 1 did not statistically differ from that for ACE 1. We have mentioned this in the revised text (Lines 141-142):
'A two-sample t test suggests that the averaged Z_{Eu} for CE 1 was not statistically different from that for ACE 1.'

13. line 115: Correlations are reversed: change increasing to decreasing and vice versa.

Response: Done.

14. Line 116: "This result suggested that nutrients in the euphotic zone were enhanced by the uplift of isotherms within this cyclonic eddy17," Don't you mean that uplift occurs at the edge of CE instead of "within"?

Response: We mean that uplift occurs within the CE because the radial distribution of

nitrate concentration in CE 1 exhibited a higher level of nitrate in the CE 1 core than at its edge.

15. line 123: Trends are reversed: change positive to negative and vice versa.

Response: Done.

16. Text on the figure was blurry, small and hard to read in my version.

Response: We now use a larger font size for the text in Figure S1 and have updated all the figures with a higher resolution.

17. Zscm is used to discuss the changes in the different properties measured. Could you explain why? How to separate the effect of the eddy dynamics and the change in sampling depth due to variations in Zscm? Is the assumption that Zscm follows a density layer? Maybe using density instead of depth would be more insightful?

Response: We used Z_{scm} to discuss the biogeochemical effects of mesoscale eddies because mesoscale eddies have been demonstrated to significantly affect the subsurface chlorophyll maximum in the North Pacific subtropical gyre (Xiu & Chai, 2020). As shown in Fig. R3a and b, Z_{scm} mainly follows the density layer σ_θ=23.8 kg m^{-3} in CE 1 and σ_θ=23.3 kg m^{-3} in ACE 1. Due to the regulation of submesoscale frontal dynamics, the Z_{scm} at the CE 1 edge was shallower than the depth of σ_θ=23.8 kg m^{-3} from 28 April to 3 May 2019. Linear regressions along the density layer present analogous results to those along the Z_{scm} for Chl and POC. There are no significant correlations between the temperature and r/R because the along-isopycnal temperature was almost the same. The linear regression result for NO_3 along σ_θ=23.8 kg m^{-3} is opposite to that along Z_{scm} in CE 1 because the isopycnal reaches a deeper depth where the nitrate level is higher from 28 April to 3 May 2019. During this period, the isoline σ_θ=23.8 kg m^{-3} was below the euphotic zone depth where there is limited phytoplankton photosynthetic activity. In contrast, the Z_{SCM} was within the euphotic zone so that the impacts of the eddy dynamics on the biological processes can be well reflected. As such, we discussed the changes of different biogeochemical tracers using Z_{scm}.

Reference:

Xiu, P. & Chai, F. (2020). Eddies Affect Subsurface Phytoplankton and Oxygen Distributions in the North Pacific Subtropical Gyre. *Geophys. Res. Lett.* 47, e2020GL087037

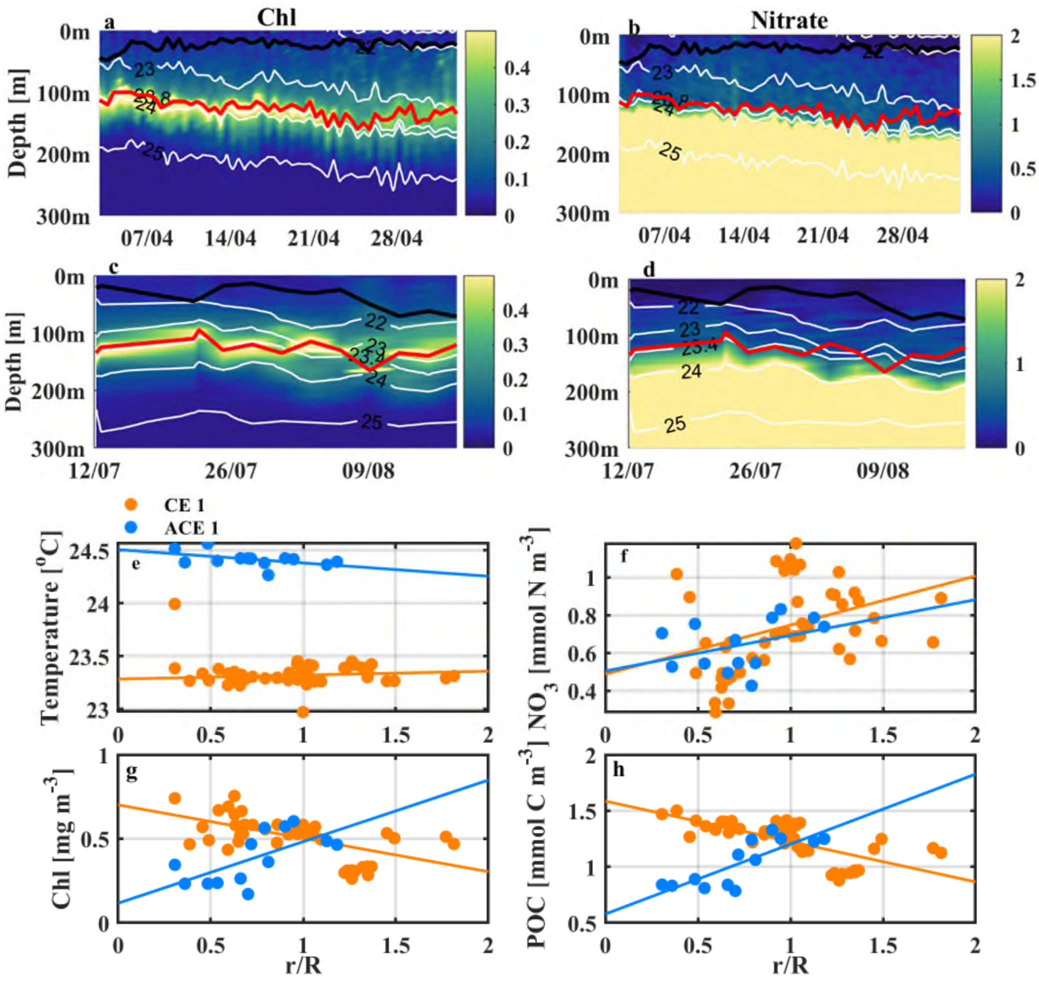

Figure R3. **a-b** Vertical distribution of (a) Chl and (b) NO_3 in CE 1. **c-d** Vertical distribution of (c) Chl and (d) NO_3 in ACE 1. Black, red and white lines in **a-d** represent the mixed layer depth, the depth of the subsurface chlorophyll maximum and the isopycnals, respectively. **e-h** Radial distributions of **c** temperature, **d** NO_3, **e** Chl and **f** POC along the density layer σ_θ=23.8 kg m^{-3} in (orange) CE 1 and σ_θ=23.4 kg m^{-3} in (blue) ACE 1. The solid lines indicate the linear regression lines.

Section 2.2

18. Please state clearly that you attribute change in integrated content over time to change in flux from the top, and detail and justify your assumptions.

Response: Thank you for the suggestion. In this study, the rate of change of the integrated POC from depth z to the mesopelagic layer is attributed to the net change between the POC flux supply from depth z and POC consumption between depth z and the base of the mesopelagic layer. Either changes in POC export or in POC consumption in the water column can affect this rate. The underlying assumption is that while the float drifted along the front, it sampled homogeneous water masses over time. We have clarified this point in the Methods section (Lines 495-507). Additionally, the assumption that the float sampled data as the time evolution of the front has been justified, as we replied to your comment #4.

19. More arguments could be provided to justify the reason for comparing the attenuation of a flux anomaly (FEI(z)) calculated with the float with the attenuation of the total POC flux from the literature.

Response: Thank you for the suggestion. For eddies, significant POC export is not sustained over their whole lifespan and is often associated with the physical dynamics of eddies (Zhou *et al*., 2020). The temporal evolution of biogeochemical processes in the eddy has been suggested to progress from nutrient injection, biomass buildup, and export enhancement during the eddy intensifying stage to biomass and export decline during the eddy decay stage (Sweeney *et al*., 2003; Zhou *et al*., 2020; Zhou *et al*., 2021). Observations have shown that intensifying eddies exhibit a large biological response, while eddies in the inactive stage do not show a significant biological response or high export flux (Sweeney *et al*., 2003; Buesseler *et al*., 2008). We assume that the magnitude of the export flux during the eddy decay stage is negligible, so that the flux anomaly approaches the total POC flux, and can be compared with those directly estimated from sediment traps or radioactive techniques recorded in the literature. Nevertheless, our estimated $F_{EI}(z)$ is a flux anomaly relative to the eddy decay stage, and it could underestimate the real POC export flux during the eddy intensifying stage. We have added this discussion to the text (Lines 227-235).

'For eddies, significant POC export is not sustained over their whole lifespan and is often associated with the physical dynamics of eddies[42,52]. Observations have shown that intensifying eddies exhibit a large biological response, while eddies in the inactive stage do not show a significant biological response or high export flux [29,31,42,52]. We assume that the magnitude of the export flux during the eddy decay stage is small, so that the enhanced net POC flux is considered as the approximation of the actual POC flux. The enhanced POC flux may underestimate the actual POC flux during the eddy intensifying stage as $F_{EI}(z)$ is a flux anomaly relative to the eddy decay stage.'

References:

Buesseler, K. O., Lamborg, C., Cai, P., Escoube, R., Johnson, R., Pike, S., Masque, P., McGillicuddy, D. & Verdeny, E. Particle fluxes associated with mesoscale eddies in the Sargasso Sea. Deep-Sea Res. II 55(10–13), 1426–1444 (2008).

Sweeney, E. N., McGillicuddy, D. J. & Buesseler, K. O. Biogeochemical impacts due to mesoscale eddy activity in the Sargasso Sea as measured at the Bermuda Time-Series Study (BATS). *Deep-Sea Res. II* **50**, 3017–3039 (2003).

Zhou, K., Dai, M. Xiu, P., Wang, L., Hu, J. & Benitez-Nelson, C. R. Transient enhancement and decoupling of carbon and opal export in cyclonic eddies. *J. Geophys. Res. Oceans* **125**, e2020JC016372 (2020).

Zhou, K., Benitez-Nelson, C. R., Huang, J., Xiu, P., Sun, Z. & Dai, M. Cyclonic eddies modulate temporal and spatial decoupling of particulate carbon, nitrogen, and biogenic silica export in the North Pacific Subtropical Gyre. *Limnol. Oceanogr.* **66**, 3508–3522 (2021).

20. Write P(z), E(z) on figure 3 to make it easier to understand.

Response: The symbols 'P(z)' and '$E(z)=\partial P(z)/\partial t$' have been added to the new Fig. 4a.

21. line 153: Is «exponentially» appropriate? It seems linear.

Response: We agree. It is not appropriate here. The word 'exponentially' has been replaced with 'gradually'.

22. line 162: The total increase in POC flux could be calculated by reconstructing the amount of remineralized POC from AOU and Redfield ratios.

Response: Thank you for the suggestion. We tried to quantify remineralized POC from AOU as suggested, but it may introduce more uncertainties to our results. The AOU multiplied by the Redfield ratios represents the heterotrophic respiration of the total organic carbon (TOC), 50-90% of which is dissolved organic carbon. Because the ratio of the POC to TOC and the Redfield ratios in the study region were not measured, we did not use the AOU to reconstruct the remineralized POC and only used AOU change to demonstrate possible changes in remineralization between different stages. We have added this discussion to the text (Lines 512-517):
'The AOU multiplied by the Redfield ratios represents the heterotrophic respiration of the total organic carbon (TOC), 50-90% of which is dissolved organic carbon[48,58,59]. Because the ratio of the POC to TOC and the Redfield ratios in the study region were not measured, we did not use the AOU to reconstruct the remineralized POC and only used AOU concentration change over time to demonstrate possible changes in remineralization between different stages.'

References:
Abell, J., Emerson, S., & Renaud, P. Distributions of TOP, TON and TOC in the North Pacific subtropical gyre: Implications for nutrient supply in the surface ocean and remineralization in the upper thermocline. *J. Mar. Res.* **58**(2), 203–222 (2000).
Hansell, D. A., & Carlson, C. A. Biogeochemistry of total organic carbon and nitrogen in the Sargasso Sea: Control by convective overturn. *Deep-Sea Res. Part II* **48**(8–9), 1649–1667 (2001).
Kheireddine, M. et al. Organic carbon export and loss rates in the Red Sea. *Global Biogeochem. Cycles* **34**, e2020GB006650 (2020).

23. Y-scales of 3g and 3h are making it confusing, maybe 0-700 m could be used.

Response: The Y-scales of 3g and h have been limited to the range 0-700 m.

Section 2.3
24. L 183: Here, the front is defined by the temperature gradient. Would it be possible to find out whether the front is dynamically active and carrying POC at depth or not?

Response: We diagnosed the frontal dynamics using more physical parameters, such as the finite-size Lyapunov exponent (FSLE), lateral buoyancy frequency (M^2) and Richardson number (R_i). The concurrence of SST gradients, high FSLE, M^2 and small R_i suggests that the front was dynamically active (Fig. S5 and S7). Below the euphotic zone depth, we found that the POC concentration was high, which indicated that the intense submesoscale vertical velocities subducted high POC downward (Fig. S9). These results have been incorporated in the main text in Lines 256-264.

25. L184/185: Can we really speak of degradation/intensification, since the float crosses the front but doesn't stay there?

Response: According to the frontogenesis theory, when the front is enhanced, it will generate upwelling on the warm side of the front and downwelling on the cold side of the front. During the sampling period, the front experienced intensifying and decaying stages, while the float was continuously sampled along its warm side (not across the front). We conducted an experiment of particle tracking and a T-S characteristic analysis of the water mass to justify that the float did drift along one side of the front, on which relatively homogeneous water masses were sampled. As such, the float observations can be interpreted as a temporal signal due to the stage change of the front. Our hypothesis is that when the front intensifies, the dynamic and biogeochemical changes on each side are generally coherent. Please see the details in our reply to comment #4.

26. L 186 / Fig. S4e: Why is the gradient lower than the background during the decaying stage? Why not equal since the front goes back to background?

Response: Thank you for the insightful question. Fig. S4e has been updated to exclude extremely high GM values when calculating the background GM. It appears reasonable now.

27. L 188: Fig. S4g: How is the strain rate calculated and from which data product? You could physically define the front and add its position to your figures to make interpretation easier.

Response: The strain rate was calculated using satellite-derived altimetry data (sea surface height data) at a resolution of 0.25°·0.25°. We realized that the spatial resolution of the strain rate is inadequate to identify the frontal structure and have removed it in this revision. We have instead used the finite-size Lyapunov exponent (FSLE) to identify the dynamic active regimes of the front calculated from the sea surface height data. The along-track time series of FSLE and lateral buoyancy frequency (M^2) presented the co-located pattern of high FSLE and M^2, suggesting that the frontal region was in active submesoscale motion. Please see Fig. S7.

Methods

28. L 313: You could spell out bbp(700) and PAR again.

Response: Following the suggestion, the words ‘bbp(700) and PAR’ have been replaced with ‘particulate backscattering coefficients at 700 nm (b_{bp}(700)) and photosynthetically available radiation (PAR)’ .

Reviewer #2 (Remarks to the Author):

This study presents an observation-based analysis of the relationship between particulate organic carbon (POC) and eddy and frontal dynamics. The authors have leveraged observations from floats with the objective to bring new insights into carbon export dynamics at fine scale. This is a valuable effort; yet there are major issues that need to be addressed before it can be published. The main concerns fall in 4 categories:

I. Gaps in context and references to prior work in introduction and discussion of the results, specifically the potential impact of frontal subduction (comment #1)

II. Missing or weak justification of the hypotheses used to interpret the data that account for the compounding effects of spatial and temporal variability, specifically the justification to use a “mostly spatial” or a “mostly temporal” framework depending on the eddy/front considered (comments #2-5)

III. Shortcomings with the conclusions and generalizations of the results that link fronts to POC export and attenuation coefficient b, which is the main point of the paper (comments # 6-10).

IV. Imprecisions on the approach that need clarification, issues with text and figures. (comments #11-28).

Detailed comments and suggestions on how to address them are provided below. The two reviewers that contributed to this review believe the authors can improve the justification of their approach, as well as the interpretation and generalization so the manuscript can be acceptable for publication.

Response: We thank the reviewer for the thoughtful and constructive suggestions. We find that the paper is significantly strengthened by addressing the four issues you are concerned with. We have responded to these questions and comments point-by-point below.

1. Gaps in context and references. The sentence of L71 is not true: “In this study, for the first time, we show the capability of using BGC-Argo floats equipped with optical sensors to estimate the carbon export flux and associated vertical attenuation in response to submesoscale fronts”

The paper is lacking key references, in particular prior studies identifying and quantifying the role of subduction by fronts, including with BGC-Argo floats. The manuscript leads us to believe that there were no attempts to quantify this subduction pump before. Yet observation based (using float AOU, spiciness) and high resolution modeling papers have been published on this topic (see list of reference below). They

need to be included to provide the adequate context in the introduction but also in the discussion. Note that the process of export by subduction along fronts explained in this study can be called differently in the literature: eddy pump, eddy subduction pump etc. Although the term “eddy pump” was often used, it does not usually refer to “Ekman pumping” in eddies but the subduction by fast velocities associated with meso and submesoscale fronts, similarly to what is considered in this manuscript.

Sentences that critically need these references include L56-67 (almost all sentences in this paragraph are missing critical references), L71 which needs to integrate these prior work. In discussion, please consider how the impact of subduction would influence your results and discuss considering these prior works.

Review about export pumps including the physical injection pump or eddy pump:
Boyd, P.W., Claustre, H., Levy, M., Siegel, D.A., Weber, T., 2019. Multi-faceted particle pumps drive carbon sequestration in the ocean. Nature 568, 327. https://doi.org/10.1038/s41586-019-1098-2

Papers estimating the role the “eddy subduction pump” using BGC-Argo, cruises and models:
Erickson, Z.K., Thompson, A.F., 2018. The Seasonality of Physically Driven Export at Submesoscales in the Northeast Atlantic Ocean. Global Biogeochemical Cycles 32. https://doi.org/10.1029/2018GB005927
Lacour, L., Llort, J., Briggs, N., Strutton, P.G., Boyd, P.W., 2023. Seasonality of downward carbon export in the Pacific Southern Ocean revealed by multi-year robotic observations. Nat Commun 14, 1278. https://doi.org/10.1038/s41467-023-36954-7
Llort, J., Langlais C., Matear R., Moreau S., Lenton A., Strutton Peter G., 2018. Evaluating Southern Ocean Carbon Eddy-Pump From Biogeochemical-Argo Floats. Journal of Geophysical Research: Oceans 123, 971–984. https://doi.org/10.1002/2017JC012861
McGillicuddy, D.J., Resplandy, L., Lévy, M., 2019. Estimating particle export flux from satellite observations: Challenges associated with spatial and temporal decoupling of production and export. Journal of Marine Research 77, 247–258. https://doi.org/10.1357/002224019828474331
Omand, M.M., D’Asaro, E.A., Lee, C.M., Perry, M.J., Briggs, N., Cetinić, I., Mahadevan, A., 2015. Eddy-driven subduction exports particulate organic carbon from the spring bloom. Science 348, 222–225. https://doi.org/10.1126/science.1260062
Resplandy, L., Lévy, M., McGillicuddy Jr., D.J., 2019. Effects of Eddy-Driven Subduction on Ocean Biological Carbon Pump. Global Biogeochemical Cycles 33, 1071–1084. https://doi.org/10.1029/2018GB006125
Stukel, M.R., Song, H., Goericke, R., Miller, A.J., 2017. The role of subduction and gravitational sinking in particle export, carbon sequestration, and the remineralization length scale in the California Current Ecosystem: Subduction and sinking particle export in the CCE. Limnology and Oceanography 63, 363–383. https://doi.org/10.1002/lno.10636

Response: Thank you for pointing this out. We agree that prior works on subduction by fronts should be included in the introduction, and its impacts on our results should be discussed, as subduction by fronts is one of the most important dynamics by which submesoscale processes affect local POC distributions. We deleted that inaccurate statement in this revision. The changes in the introduction and discussion sections are summarized below:

1. Changes in the introduction section

To provide a more adequate context in the introduction that the submesoscale process affects local POC export, we first added one sentence in Lines 57-60 to mention the eddy-subduction pump:
'*The vertical motions can inject high POC produced in the euphotic zone into the mesopelagic layer (referred to as eddy subduction pumps)*[17,19,22,24]*, resulting in long-term carbon sequestration*[17,19,20,23]*.*'

Second, prior works that attempted to identify and quantify eddy subduction pumps have been acknowledged in Lines 68-71:
'Prior studies have captured anomalous features of elevated POC at depth, which are interpreted as evidence of eddy subduction pump[19,22,23]*. Despite their potential importance in shaping POC export, direct observations of the downward POC flux and its attenuation in response to submesoscale fronts are scarce*[17,19-21,23,41]*.'*
and Lines 76-81 :
'Observational and high-resolution modeling studies have suggested that the eddy subduction pump contributes ~19% to 50% to the total export of POC. In contrast, Resplandy et al. (2019) explored the integrated effects of eddy subduction hot spots on the biological carbon pump and found that they contribute less than 5% to carbon export due to the strong compensation between upward and downward fluxes.'

Additionally, key references have been added in Lines 71 (*Omand et al., 2015; Llort et al., 2018; Erickson et al., 2018; Lacour et al., 2023; Boyd et al., 2019*) and Line 86 (*Llort et al., 2018; Lacour et al., 2023*).

Finally, we acknowledge that Llort et al. (2018) was the first to quantify the carbon export flux associated with submesoscale fronts by using BGC-Argo. Previous studies that quantified the carbon export flux at submesoscale using BGC-Argo have also been cited in this revision. In comparison, however, these previous studies didn't consider the vertical attenuation of the carbon flux at submesoscale fronts, which is the main contribution of our study. To integrate the previous efforts and clarify the novelty of our results, we have rephrased the sentence as shown below (Lines 91-96):
'The BGC-Argo has been used to identify elevated POC in the mesopelagic layer and estimate the carbon flux associated with submesoscale features[20,23]*. In this study, we further show the capability of using BGC-Argo floats to estimate the transfer efficiency*

of carbon export in response to submesoscale fronts.'

2. Discussion on the impacts of subduction on POC export

We agree with comments #8 and #9 that the impacts of subduction by front should be taken into account to interpret our results. More discussion has been added in this revision.

As you mentioned in comment #9, the high POC at a depth of 200 m at the edge of CE 1 could be due to subduction of POC from the productive layer by the front. After a detailed examination, we agree with this comment and have added additional text (Lines 376-382) to discuss this point. Please see our response to this comment #9.

On the other hand, we found that the influence depth of eddy subduction may not be deep enough to affect the lower mesopelagic layer. The elevated POC concentration and flux were only present at depths between Z_{Eu} and ~250 m. We tested different ways to fit the POC flux attenuation coefficient (*b*) with and without considering the POC flux in the subduction layer (Z_{Eu}~250 m). The *b* value was about 0.65 excluding the POC flux in the eddy subduction layer, which was higher than that considering the subduction layer (*b*=0.56), but was still lower than those estimated in eddies and normal conditions. This suggests that submesoscale dynamics affect POC export flux not only through physical subduction but also through modulating particle properties (e.g., size, sinking speed and composition). We have added a paragraph (Lines 373-393) to fully discuss the impact of eddy subduction on our results. Please find our detailed response to comment #8.

References:

Boyd, P.W., Claustre, H., Levy, M. et al. Multi-faceted particle pumps drive carbon sequestration in the ocean. *Nature* **568**, 327–335 (2019).
Resplandy, L., Lévy, M. & Mcgillicuddy, D. J. Effects of eddy-driven subduction on ocean biological carbon pump. *Glob. Biogeochem. Cycles* **33**, 1071–1084 (2019).
Omand, M. M. et al. Eddy-driven subduction exports particulate organic carbon from the spring bloom. *Science* **348**, 222–225 (2015).
Lacour, L., Llort, J., Briggs, N. et al. Seasonality of downward carbon export in the Pacific Southern Ocean revealed by multi-year robotic observations. *Nat. Commun.* **14**, 1278 (2023).
Erickson, Z. K. & Thompson, A. F. The seasonality of physically driven export at submesoscales in the Northeast Atlantic Ocean. *Global Biogeochem. Cycles* **32**, 1144–1162 (2018).
Stukel, M. R., Song, H., Goericke, R. & Miller, A. J. The role of subduction and gravitational sinking in particle export, carbon sequestration, and the remineralization length scale in the California Current Ecosystem. *Limnol. Oceanogr.* **63**, 363–383 (2018).
Llort, J. et al. Evaluating Southern Ocean carbon eddy-pump from biogeochemical-

argo floats. *J. Geophys. Res. Ocean.* **123**, 971–984 (2018).
McGillicuddy, D. J. et al. Estimating particle export flux from satellite observations: Challenges associated with spatial and temporal decoupling of production and export. *J. Mar. Res.* **77**, 247–258 (2019).
Lévy, M. et al. The Impact of Fine-Scale Currents on Biogeochemical Cycles in a Changing Ocean. *Annu. Rev. Mar. Sci.* **16**,1 (2024).

2. Missing justification of the hypotheses, specifically the justification to use a "mostly spatial" or a "mostly temporal" framework to interpret the float data.
The authors alternatively interpret the observed changes along the float trajectories as "mostly spatial changes" (CE1 and ACE1, Figure 2), or "mostly temporal changes" i.e. pseudo lagrangian (CE2 and front, Figure 3). The hypothesis of "mostly temporal" is justified in the text for CE2 (L128 and 141). However, the justification to interpret as "mostly spatial" the changes along CE1 and ACE1 is missing, and the hypothesis to interpret as "mostly temporal" the changes attributed to frontal dynamics seems inadequate (see details in comments 3 and 4 below). We encourage the authors to explain clearly and early these different strategies to orient and prepare the reader.

Response: Thank you for pointing this out. In this study, we performed different analyses based on different sampling scenarios of the floats to delineate temporal and spatial variabilities of the measured variable. For the hypothesis of 'most spatial changes' in eddies (CE1 and ACE1), we first showed a radial increase of the relative distance between the float and the eddy center with time. Then, we demonstrated that the change of surface Chl measured by the floats was consistent with the radial variation of remote-sensed surface Chl from the eddy core to the eddy edge. In addition, with remote sensing data, we can track the temporal evolution of Chl in eddies, and this temporal pattern was completely different from the change of float-measured surface Chl. Therefore, for this sampling period, we think the float-measured variability of POC can be used to represent the spatial variability of POC from the eddy core to the eddy edge.

For the hypothesis of 'most temporal changes' along a submesoscale front, we added more analysis about the float-measured characteristics of the water masses. We also conducted a numerical particle tracking experiment. These new results all suggest that the float continuously drifted along the warm side of the front on which homogeneous water masses were sampled. During this period, the front experienced intensifying and decaying stages, while the float was still sampling along its warm side. As such, the float observations at the front can be interpreted as a temporal signal due to the stage change of the front.

Following the suggestions, we now justify these two hypotheses early in Sections 2.1 and 2.3, respectively. Please see more details in our following responses to comments #3 and #4.

3. The authors need to demonstrate why it is justified to ignore temporal variations when analyzing CE1 and ACE1. Figure 2 interprets all POC data as spatial changes (using radial structure of the eddies CE1 and ACE1). The approach is attractive, but the text and figures should clearly show that this is a valid hypothesis. Specifically, how much of the signal that is interpreted as spatial variability across the eddy is in fact introduced by the temporal evolution along the trajectory of the float? At least, the authors could indicate the day of sampling for each data point on Figure 2? [Colors could indicate the day of sampling while symbols could indicate the depth]. This would allow the reader to see how much of the variations attributed to the spatial structure of the eddy could be introduced by temporal changes.

Response: Thank you for the constructive comment. For CE 1 and ACE 1, we interpreted the measured variability of POC as the radial structures of eddies based on the following considerations:

Firstly, there was an increasing relative distance between the float and the eddy center with time. The location of the float changed from inside the eddy to outside the eddy, with a relative distance of r/R between 0.4~2.0 for CE 1 and 0.23~1.2 for ACE 1.

Secondly, the spatial distribution of Chl in eddies was derived by compositing the remote-sensed sea surface Chl over the float sampling period. This satellite-derived Chl was then interpolated onto the relative locations of the float in eddies to compare with that measured by the float. In CE 1, both the satellite- and float-derived Chl decreased and then increased in the radial direction, reaching a peak at r/R=1~1.5 (Fig. S2). In ACE 1, both the satellite- and float-derived Chl showed a double peak pattern along the radial direction (Fig. S2a). The correlations between the satellite- and float-derived Chl were 0.39 (p=0.01) and 0.43 (p=0.09) for CE1 and ACE 1, respectively. The relatively consistent changing patterns of Chl between the float and remote sensing data suggest that the float captured the spatial variability of the eddies in the radial direction.

Lastly, it is likely that the temporal signal was also included in the float data as it exhibited larger 95% confidence intervals than those derived from satellite observations (Fig. S2a). With remote sensing data, we can track the temporal evolution of the Chl in eddies, and this temporal pattern was completely different from the change of float-measured surface Chl (Fig. S2b). The correlations between the satellite- and float-derived Chl over time were -0.18 (p=0.24) and -0.05 (p=0.82) for CE1 and ACE 1, respectively, suggesting that the variability explained by the temporal evolution of Chl of eddies was much small. Therefore, for this sampling period, we think the float-measured variability of variables can be used to represent the spatial variability of eddies.

To clarify this point, we added the following sentences in Lines 143-154 and Fig. S2 before we presented the radial POC distributions in the text:

'The floats may capture the spatial variability of tracers for CE 1 and ACE 1 as the

location of the floats changed from inside the eddies to outside the eddies. The satellite-derived sea surface Chl was averaged over the float sampling period and then interpolated onto the relative locations of the float in eddies to compare with that measured by the float. The float-measured Chl was in good agreement with that derived from satellites in the radial direction (Fig. S2a), with correlations of 0.39 (p=0.01) for CE1 and 0.43 (p=0.09) for ACE 1. In contrast, the temporal evolution of satellite-derived Chl averaged inside eddies was completely different from that of float-measured Chl (Fig. S2b), indicated by low correlations (R= -0.18 (p=0.24) for CE 1 and R=-0.05 (p=0.82) for ACE 1). Therefore, the float-measured tracers during this period can be used to represent the spatial variability of the eddies along the radial direction.'

Another thing worth mentioning is that the high POC distribution at the edge of CE 1 was due to subduction by the front (please see our reply to comment #9). This further strengthens the point that the distributions of POC captured by the float reflect the spatial variability of the eddy. We have added the following sentence in Lines 169-170 to emphasize this point:
'This further supports that the radial distributions of POC measured by the float reflect the spatial variability of the eddy.'

Following the reviewer's suggestion, colors have been indicated in Fig. 2. For CE 1, as the float moved almost monotonically from the eddy core to the edge with time, it is still not easy to tell the influence of the temporal signal from this figure. Figure R1 shows the variations of the strength of the eddies. It is clear that although CE 1 was strengthened in early May while the float was at r/R=~1.3, the float-measured POC at SCM layer was still lower than that measured at the eddy core (Fig. 3), implying dominant variability caused by space instead of time.

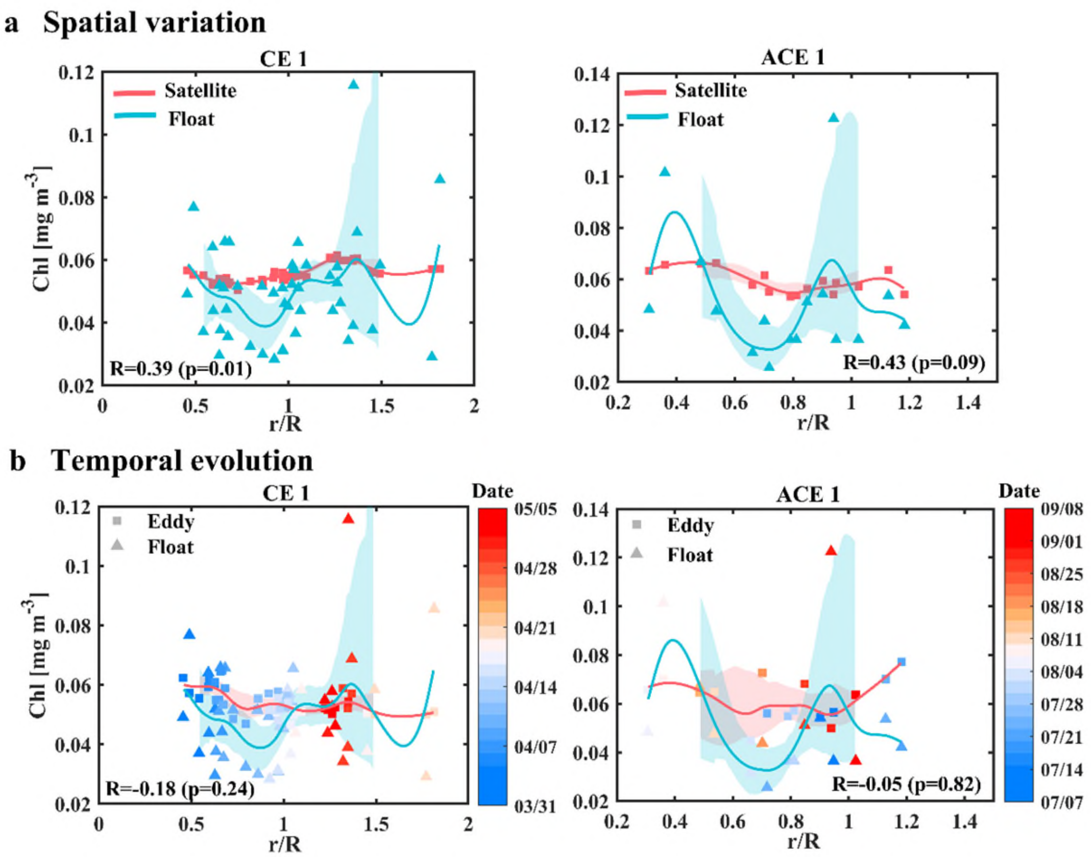

Figure S2. Comparison of sea surface Chl between satellite and BGC-Argo observations in both spatial and temporal variability. **a** Radial distribution of sea surface Chl at the float locations derived from both the BGC-Argo and satellite observations. The BGC-Argo results are directly obtained from the surface Chl measured by the float. The satellite-derived Chl values are calculated by averaging the Chl from OC-CCI over the float sampling period and then interpolating to the relative locations of the float in eddies. Because the eddies propagated over time, the location of the float in the eddies was constructed by calculating the azimuthal and radial positions of the float relative to the eddy center. **b** Satellite-derived Chl averaged inside eddies and sea surface Chl derived from BGC-Argo observations. The BGC-Argo results are directly obtained from the surface Chl measured by the float. The satellite-derived Chl values are calculated by averaging the satellite-derived Chl values inside the eddies at the time when the float was at the position of r/R.

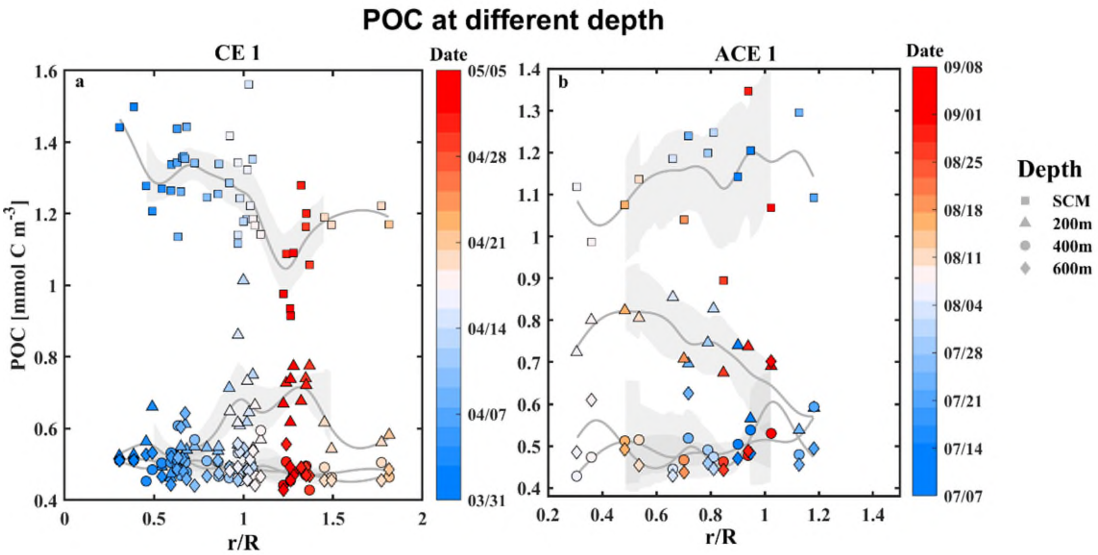

Figure 3. POC distributions at different depths within **a** CE 1 and **b** ACE 1.

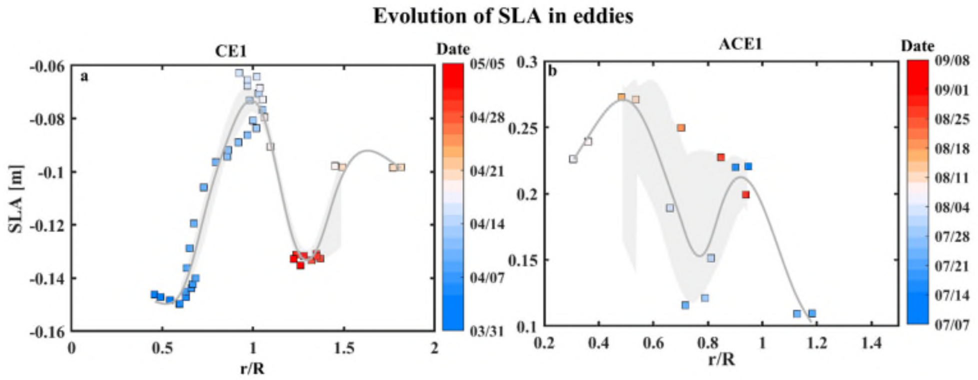

Figure R1. Evolution of SLA averaged inside eddies. The SLA values are calculated by averaging the SLA inside the eddies at the time when the float was at the position of r/R.

4. The hypothesis of "mostly temporal" is highly disputable for the front. It is ok for eddy CE2 because the float seems to stay stuck close to the center of the eddy, and most changes can be interpreted as temporal changes. In the case of the front, however, the float seems to be sampling different parts of the front. The associated changes in SLA, POC, AOU etc. are therefore likely to come from spatial variations across the front and not temporal variations. The authors could demonstrate that it is indeed temporal, however it is unlikely to be successful and convincing given the complexity of the dynamics in the frontal structure. This shortcoming has major implications for the result interpretation and the conclusions of the paper as the authors interpret these changes as temporal changes while they are likely spatial changes. This section of the results and associated conclusions needs to be revised.

Response: Thank you for the insightful comment. According to the frontogenesis

theory, when the front is enhanced, it will generate downwelling on the cold side of the front and upwelling on the warm side of the front. The hypothesis of 'mostly temporal' is that the float drifted along one side of the front so that relatively homogeneous water masses over time were sampled. During this period, the front experienced intensifying and decaying stages, while the float was still sampling along its one side. As such, the float observations at the front can be interpreted as a temporal signal due to the stage change of the front. We have provided more justifications in the revised text to interpret this hypothesis.

By investigating the time series of FSLE along the float trajectory, we found that the float traveled along the same filament with high FSLEs. This result, in combination with the Chl, SST and SST gradient distributions (already shown), demonstrated that the float continuously traveled along the warm side of a front during that period.

To further support that the float drifted along one front, a numerical particle tracking experiment was conducted at the frontal region using the TRACMASS code. The particle was released at the same location as the float on 21 April 2019 and it was moved purely by advection using the surface geostrophic current derived from AVISO data. The numerical tracking results showed that the float mainly followed the path of the particle (Fig. S14), suggesting that the float moved in a quasi-Lagrangian manner and sampled a similar water mass during the analysis period (supplementary information Text S3).

The homogeneous water masses sampled on one side of the front can be supported by the transect of temperature and salinity anomalies (Fig. S6). The temperature was higher in the upper 300 m and the salinity was lower in the upper 150 m depth compared to the CE 1 core. In addition, the T-S diagram presents a coherent physical characteristic from 21 April to 3 May 2019, suggesting that the float was moving along the front but not across the front.

Overall, these results support the use of the float data as a time series on the warm side of the front during the analysis period.

We have clarified this point in the text in Lines 247-250:
'This is further supported by a Lagrangian particle tracking experiment, which suggests that the float drifted in a quasi-Lagrangian manner and sampled a similar water mass along one side of the front (Text S3).'
, and Lines 250-255:
'The front exhibited a higher temperature in the upper 300 m and lower salinity in the upper 150 m compared to the CE 1 core (Fig. S6), suggesting homogeneous water masses sampled at the frontal region. The T-S diagram demonstrates a coherent physical characteristic from 21 April to 3 May 2019 (Fig. S6). All these results suggest that the float sampled homogeneous water masses along the warm side of the front.'

Text S3. Lagrangian particle tracking experiment on the front

To investigate whether the float drifted along the front, a numerical particle tracking experiment was conducted using the TRACMASS Lagrangian trajectory code (Döös, 1995; Blanke & Raynaud, 1997). The particle was released at the same location as the float on 21 April 2019 and transported purely by surface current. Here, we run the code with the current velocity derived from AVISO. The Lagrangian particle trajectory derived from the particle tracking model demonstrated that the virtual particle starting at the location of the float moved along the front (Fig S14). The float moved slower than the particle but mainly followed its path, suggesting that the float drifted in a quasi-Lagrangian manner during the analysis period. The difference in moving velocity is that the particle was only transported by surface current, whereas the motion of the float was driven by both the surface and deep currents.

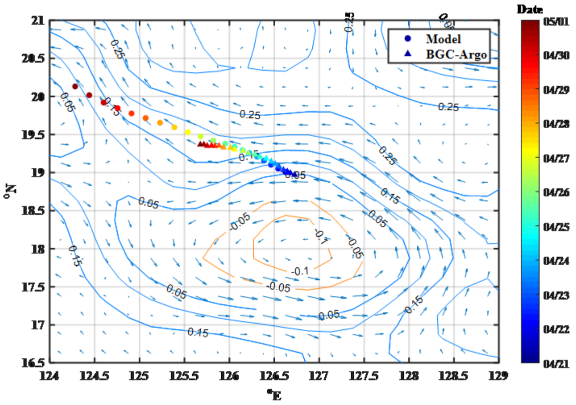

Figure S14. Trajectories of the BGC-Argo and the virtual particle over the period when the float was on the front (21 April - 3 May 2019). The particle was released at the same position as the float on 21 April 2019 and transported by the surface current velocity derived from AVISO. The particle trajectory was tracked using the TRACMASS code. Color indicates the dates. Blue and red lines are the contour lines of the sea level anomaly (SLA). Arrows represent the sea surface current velocity anomaly.

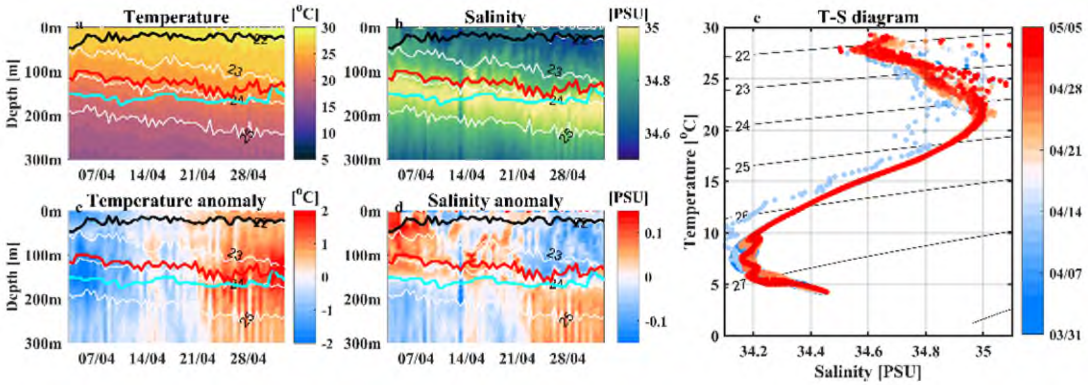

Figure S6. Physical characteristics of the float measurements. During 21 April - 3 May 2019, the float was on one side of the front. Time series of **a** temperature, **b** salinity, **c** temperature anomaly and **d** salinity anomaly measured by float 2902753 from 1 April

to 3 May 2019. The black, red and cyan lines indicate the mixed layer depth (MLD), the depth of subsurface chlorophyll maximum (Z_{SCM}) and the euphotic zone (Z_{Eu}), respectively. The white lines are the isopycnals of 23, 24 and 25 kg m^{-3}. The temperature and salinity anomalies were defined as the instantaneous values after subtracting the full time mean. **e** Temperature-salinity diagram with data points from float 2902753 from 1 April to 3 May 2019. The color indicates the sampling date.

5. The sampling context should be better presented. In Figure 1, eddies and float trajectories appear completely disconnected. This is likely because the black lines show the center of the eddy and not their span. Could you indicate the radius of the eddies around the trajectories so we can see that they were indeed in the eddies? Also we encourage the authors to include a figure similar to Figure S4a to show the float relative to the eddy/front. Maybe choose snapshots that show the overlap with the front as a function of time.

<u>**Response:**</u> Thank you for this suggestion. The gray lines indicating the eddy radius have been overlapped in Fig. 1a to show the connection of the eddy centers and the floats. Additionally, snapshots showing the locations of the floats relative to the eddies and the front have been included in the new Figure 2.

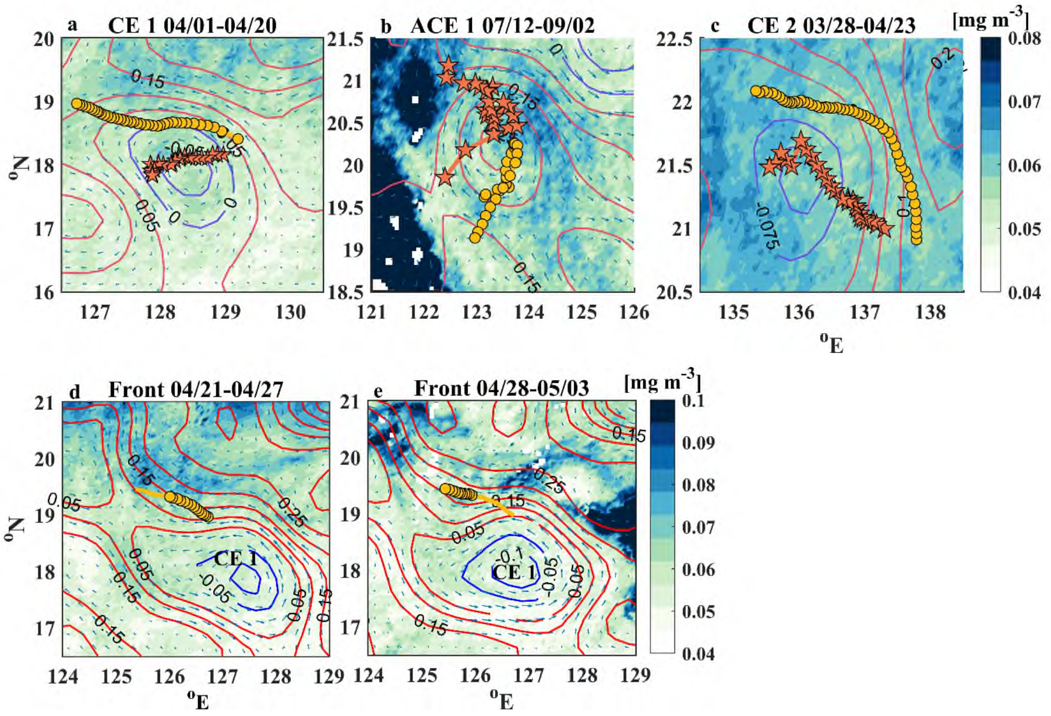

Figure 2. The BGC-Argo and eddy trajectories. **a** The locations of (yellow dots) the BGC-Argo float (WMO number 2902753) and (orange pentagrams) the center of cyclonic eddy 1 (CE 1) during 1-20 April 2019. **b** The locations of (yellow dots) the BGC-Argo float (WMO number 2902753) and (orange pentagrams) the center of anticyclonic eddy 1 (ACE 1) during 12 July - 2 September 2019. **c** The locations of (yellow dots) the BGC-Argo float (WMO number 2902756) and (orange pentagrams) the center of cyclonic eddy 2 (CE 2). **d** The locations of the BGC-Argo floats along the front during 21-27 April 2019. The yellow line is the float trajectory from 21 April to 3

May 2019. **e** The same as d but during 28 April-3 May 2019. Background color and isolines represent sea surface chlorophyll and sea level anomalies, respectively. The arrows are the geostrophic velocity anomaly.

6. Generalization between front and carbon export. L214-222. "A strong correlation between the b value and GM was found …. This statistical relationship implies that a strong front is linked with high POC transfer efficiency to the mesopelagic layer, which could be used as a parameterization to account for the impact of fronts on carbon export in large-scale models, once the front intensity at the surface is known". This conclusion seems not backed up by the results. More complete models seem to show that b can vary according to many other biophysical parameters, making this generalization highly debatable (see for instance Ngyuen et al. 2022: Microbes contribute to setting the ocean carbon flux by altering the fate of sinking particulates). This is one of the main points of the paper; the caveats need to be discussed properly.

Response: Thank you for pointing this out. We agree that a parametrization of the *b* value as a function of the front intensity is inadequate since the efficiency of POC transfer is modulated by complicated physical and biogeochemical processes. We have rephrased the sentence as follows to be clearer:
'This statistical relationship implies that a strong front is linked with high POC transfer efficiency to the mesopelagic layer, which could be used to estimate the impact of fronts on carbon export at the basin-scale oceans.'

We used this relationship to estimate the impacts of fronts on POC transfer efficiency. The underlying mechanism is that submesoscale dynamics play a role through their impact on the particle sinking velocity or particle lability. The following discussion has been added to the manuscript (Lines 405-413):
'The estimation suggests that submesoscale fronts are important in setting the spatial and temporal variations in POC transfer efficiency. Previous carbon cycle studies have suggested that the attenuation of the POC flux is determined by the rate of POC production in the euphotic zone, the sinking velocity of particles, and the rate of POC consumption in the mesopelagic layer[5-10,38,39,49-54]. Submesoscale dynamics may play a role in POC flux transfer through eddy subduction that directly enhances the particle sinking velocity or through its impacts on the plankton community structure in the euphotic zone that generates particles with large sinking velocities and/or low remineralization rates[9,14-23,32].'

The caveat of the estimation conducted in this study is that we did not explicitly include other physical and biogeochemical processes that affect POC flux attenuation. These processes include mixed layer pumping, bacterial colonization and consumption, zooplankton migration, consumption, and fragmentation. However, there are no clear data or previous studies showing why these processes could be particularly enhanced or weakened at fronts. We provide some discussions here. We think inclusion of these processes may allow a better constraint in estimating POC transfer efficiency, but it

definitely requires future work to examine how submesoscale dynamics affect these processes. To clarify this point, we have added the following sentences to the text (Lines 413-425):

‘Our estimation suggests that submesoscale fronts increase the POC transport efficiency in the NPSG. However, it should be kept in mind that other processes that can potentially modulate the b value (e.g., microbial POC consumption[49,54], POC fragmentation by zooplankton[8]) were not explicitly included in the estimation. Quantifying the mesopelagic fragmentation processes revealed that roughly half of the observed flux attenuation was due to the fragmentation of large particles into small particles[8]. Ngyuen et al. showed that microbial dynamics may play a significant role in shaping the POC flux profiles observed in global oceans[54]. How these dynamics respond to submesoscale fronts, however, is still not clear. Future work including dynamics such as microbial consumption and zooplankton disaggregation of the particles is needed, and may allow for better constraints on the relative contributions of submesoscale fronts to the basin-scale carbon transport efficiency.’

7. Conclusion about seasonality and export. line 234: You did not take into account that the seasonality of mesoscale and frontal dynamics is different from the seasonality of POC content in the water column. Consequently, the amount of POC transported by the same front strength varies depending on the season. This could completely reverse your conclusion about the seasonal evolution of the parameter b. Please revise or strengthen these points.

Response: Thank you for pointing this out. The relationship between the *b* value and submesoscale dynamics was derived from multiple observations in subtropical gyres, which cover both winter and summer months. This suggests that submesoscale dynamics can significantly affect POC transport efficiency in different seasons. As the POC transport efficiency is largely determined by the *b* value, POC concentration usually won’t affect the transport efficiency. Instead, the properties of the POC (e.g., size, lability) are important in changing the *b* value. Submesoscale dynamics have been suggested to affect the POC transfer efficiency by stimulating the growth of large plankton, which in turn generate large and rapidly sinking particles at the front. Regarding the same POC concentration with slow sinking in the background and with fast sinking at the front, the POC transfer efficiency will be higher at the front than in the background. Therefore, to derive a robust *b* value function, the properties of POC should also be considered. However, there are few POC property data available for now.

We examined the dataset from Station ALOHA, and found that the relationship between the *b* value and POC concentration was not clear at the seasonal timescale. At ALOHA, long-term records of the POC flux profiles suggest that the POC transfer efficiency is higher in winter than in summer (Fig. S16). On the other hand, the POC concentrations are generally higher in summer than in winter, which is indicated by the averaged profiles of POC at ALOHA (Fig. R2).

Overall, the result of a lower *b* value in winter than in summer derived from the relationships of both b_T and b_{GM} is in good agreement with the in situ result at ALOHA (Fig. S16), suggesting that the conclusion is likely robust. To support our results, the observational results of the seasonality of the *b* value (Fig. S16) have been included in the text.

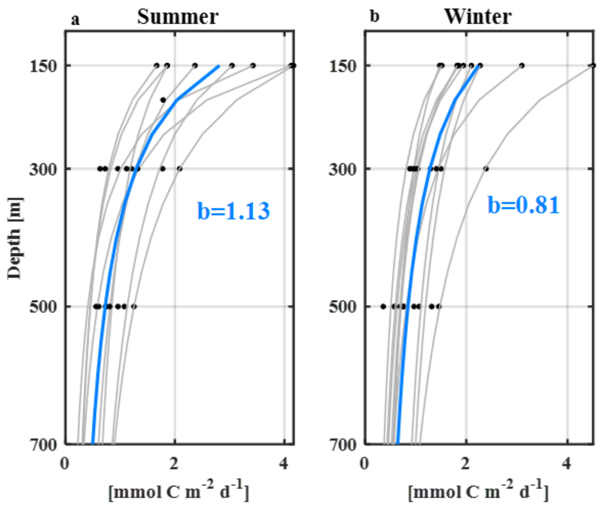

c

Summer	*b*	R^2	winter	*b*	R^2
1	1.08	0.93	1	0.93	0.98
2	1	1	2	0.93	1
3	0.83	1	3	0.94	1
4	1.63	0.99	4	0.61	0.96
5	0.66	0.95	5	0.81	1
6	1.17	0.98	6	0.91	0.93
7	0.89	0.96	7	0.53	0.99
8	1.77	0.94	8	1.11	0.98
			9	0.54	0.93
			10	0.78	1
Mean *b*	1.13			0.81	

Figure S16. The seasonal *b* value at the ALOHA station. **a** POC flux profiles and the corresponding Martin curves in summer. Black dots are observational profiles from sediment traps. Gray lines are the fitted Martin curves. The blue line is the Martin curve derived from the averaged *b* value and averaged POC flux at 150 m. **b** is the same as **a** but in winter. **c** The *b* value and R^2 for each observational POC flux profile in both summer and winter. We picked up all observational POC flux profiles with at least three vertical sampling points for Martin curve fitting. The *b* values in summer are significantly different from those in winter, as h=1 and p=0.036 in the two-sample t test result.

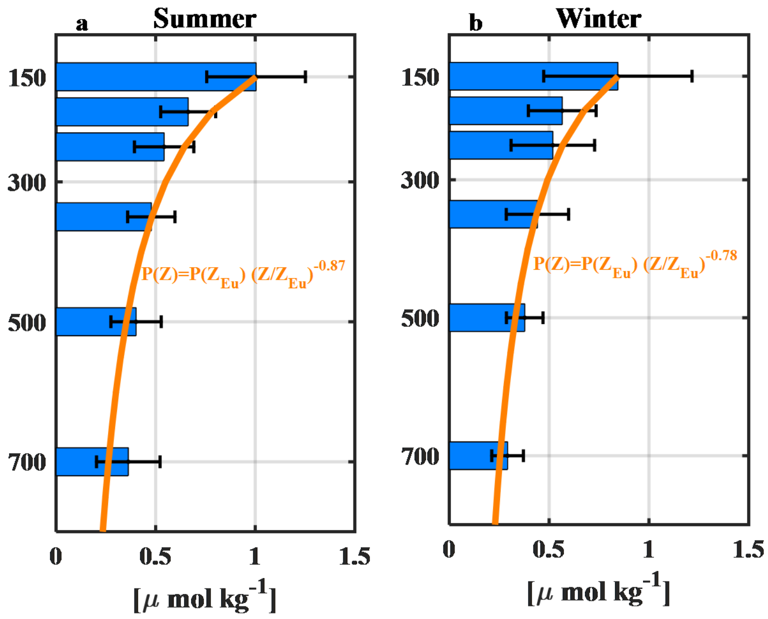

Figure R2. The seasonally averaged POC profiles at ALOHA. **a** The vertical

distributions of POC concentrations in summer averaged over 1998-2021. The bars show the averaged POC at depths of 150 m, 200 m, 250 m, 350 m, 500 m and 750 m. The orange line is the fitted attenuation curve of the averaged POC concentrations with depth. Error bars show one standard deviation for the averaging. **b** The same as **a** but in winter.

8. Missing consideration of subduction #1. The POC export depth by fronts also depends on the penetration depth of the vertical velocities associated with the fronts (subduction, see comment #1 for a list of papers that describe this). Shallow fronts do not allow POC to be exported below the pycnocline and would limit the frontal effect on b, further weakening your conclusion and the link established between fronts and POC export/attenuation coefficient. Please consider this effect of subduction in introduction and importantly in your results and interpretation. This is one of the weakest point of the paper. See also comment 9 below.

Response: Thank you for the constructive suggestion. In this study, we consider two mechanisms affecting POC export at fronts, which have been shown to be important in other regions in previous studies. The first mechanism is through biological carbon pump. Strong submesoscale flows can enhance the vertical nutrient supply, alter the phytoplankton community structure, and generate particles sinking to depth. Some of the particles generated through this mechanism, if not remineralized quickly, have the potential to penetrate the pycnocline and reach the mesopelagic layer via gravitational sinking. The second mechanism is through subduction, by which particles are carried downward with the water mass. For this mechanism, the vertical depth the particle can reach is largely determined by the subduction velocity and the ventilation depth. Of course, these two mechanisms could coexist in some cases. Following the reviewers' comments, we added more discussion about the effect of subduction in this revised manuscript.

From our data, two lines of evidence revealed that eddy subduction contributes to POC export at the front. First, a higher POC concentration between Z_{Eu} (bottom of the euphotic zone) and ~250 m depth at the front than that in CE 1 was captured by the float, which is likely due to subduction of POC from the productive layer by the front (see details in Reply #9). Second, the POC fluxes between Z_{Eu} and ~250 m depth were highest in the vertical direction, which is different from the classical biological carbon pump curve and implies POC supply from other mechanisms (Fig. S10). As the high POC signal was associated with tilting isopycnals, it was very likely induced by subduction at the front.

However, subduction appeared limited to depths between Z_{Eu} and ~250 m (Figs. S9 and S10). We tested different ways to fit the POC flux attenuation coefficient (*b*) with and without considering the POC flux in the subduction layer (Z_{Eu}~250 m). The *b* value was approximately 0.65 excluding the POC flux in the subduction layer, which was higher than that considering the subduction layer (*b*=0.56) but was still lower than those

estimated in eddies and normal conditions. This suggests that submesoscale dynamics affect POC export flux not only through physical subduction but also through modulating particle properties (e.g., size, sinking speed, and composition). Our results emphasize the complexity of submesoscale dynamics at play in enhancing POC transfer efficiency.

As we mentioned in the response to comment #1, the introduction of the effect of the eddy subduction pump has been included in the introduction section. Additionally, we added one paragraph in the discussion section to interpret and fully discuss the impact of the eddy subduction pump on our results (Lines 373-393):
'The high transfer efficiency of POC at the front could also result from the physical subduction of POC by the intense submesoscale vertical velocities. Two lines of evidence have revealed that the eddy subduction pump contributes to carbon exports at the front. First, frontal dynamics are responsible for the elevated POC at 200 m depth at the CE 1 edge of r/R=1 and 1.3 (Fig. 2a). At the positions of r/R=1 and 1.3, the float was right on the filaments of high FSLE accompanied by high M^2 and small R_i in the vertical direction, which is suggestive of ongoing intense submesoscale vertical motions (Fig S5). Concurrence with the physical dynamics was an elevated POC concentration between Z_{Eu} and ~250 m compared to those in CE 1 (Fig. S9), which is evidence of eddy subduction pumps suggested in previous studies[17,19,20,23]. Second, the POC fluxes between Z_{Eu} and ~250 m influenced by the eddy subduction pump were obviously higher than those above Z_{Eu} and below 250 m (Fig. S10). We tested different ways to fit the POC flux attenuation coefficient (b) with and without considering the POC flux in the subduction layer (Z_{Eu}~250 m). The b value was approximately 0.65 excluding the POC flux in the subduction layer, which was higher than that considering the subduction layer (b=0.56) but was still lower than those estimated in eddies and normal conditions. This suggests that submesoscale dynamics affect POC export flux not only through physical subduction but also through modulating particle properties (e.g., size, sinking speed, and composition). These results emphasize the complexity of submesoscale dynamics at play in enhancing POC transfer efficiency.'

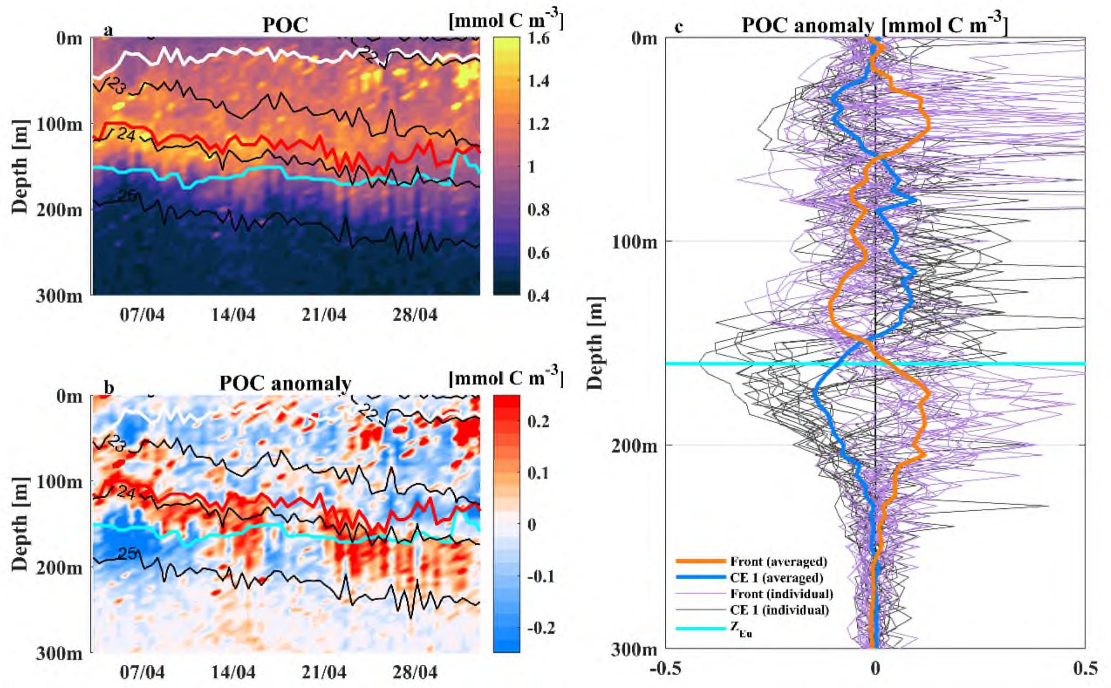

Figure S9. Vertical POC concentration distributions. **a-b** Time series of **a** POC distribution and **b** POC anomaly measured by float 2902753 from 3 April to 3 May 2019. The black, red and cyan lines indicate the mixed layer depth, depth of the subsurface chlorophyll maximum and euphotic zone, respectively. The POC anomalies were defined as the instantaneous values subtracting the full time mean. **c** Vertical distributions of the POC anomaly in CE 1 and at the front.

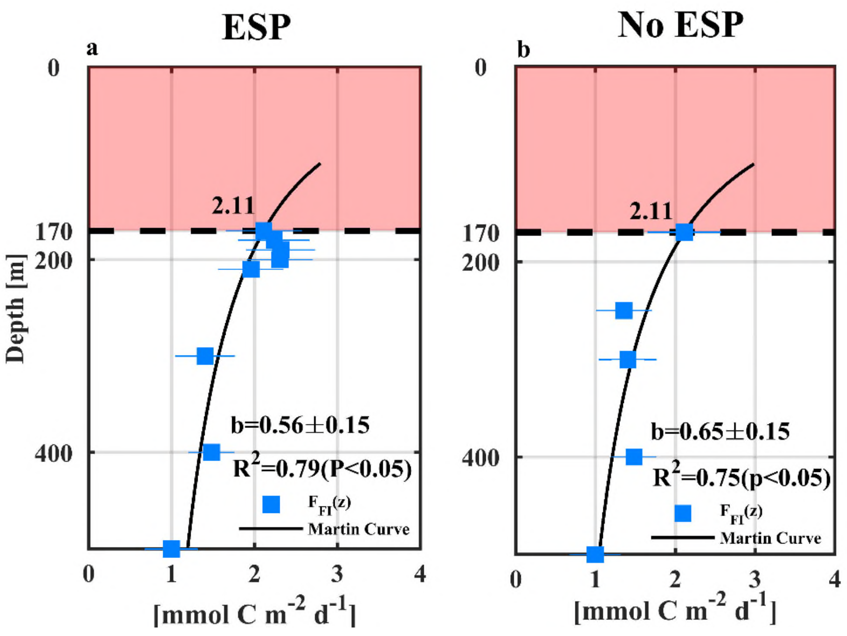

Figure S10. POC flux profiles. **a** The POC flux between Z_{Eu} and 250 m that is affected by eddy subduction pump (ESP) is accounted for when estimating the *b* value. **b** The ESP effect is not accounted for when estimating the *b* value. The black dashed lines and

error bars correspond to the euphotic zone depth and one standard deviation, respectively. The red areas represent the euphotic zone.

9. Missing consideration of subduction #2. L118: "The POC distributions attenuated faster in the CE 1 center than at its edge, which is likely due to the higher remineralization of the newly produced and easily degradable organic matter inside the eddy core8,36,37." Is this really what is happening here? Couldn't it be that intense vertical velocities associated with the frontal dynamics at the boundary of the eddies are transporting/subducting material down?

Response: As the reviewer suspected, the POC attenuated more slowly at the CE 1 edge than in its center because the subduction of POC by the front increased the subsurface POC at the edge. Specifically, the POC at 200 m presented a high value at r/R=~1 and 1.3, where the float was right on filaments undergoing intense submesoscale vertical motions. The proof to support this conclusion comes from the concurrence of high FSLE at the surface, large along-track M^2 and small R_i in the vertical direction (see Fig. S7). Meanwhile, the POC was higher between Z_{Eu} and ~250 m at the front compared to the eddy edge of other positions, likely due to subduction of POC from the productive layer by the front (Fig. S9). We have therefore rephrased this sentence as follows (Lines 165-169):

'The POC at 200 m reached its peaks at the edge of r/R=~1 and 1.3, which is likely due to subduction of POC from the productive layer by the front[17,19,20,23]'

More interpretations of the results have been added in the discussion section, specifically with the group of sentences shown below (Line 376-382):

'the frontal dynamics are responsible for the elevated POC at a depth of 200 m at the CE 1 edge of r/R=1 and 1.3 (Fig. 3a). At the positions of r/R=1 and 1.3, the float was right on the filaments of high FSLE accompanied by high M^2 and small R_i in the vertical direction, which is suggestive of ongoing intense submesoscale vertical motions (Fig S5). Concurrence with the physical dynamics was an elevated POC concentration between Z_{Eu} and ~250 m compared to those in CE 1 (Fig. S9), which is evidence of eddy subduction pump suggested in previous studies[17,19,20,23].'

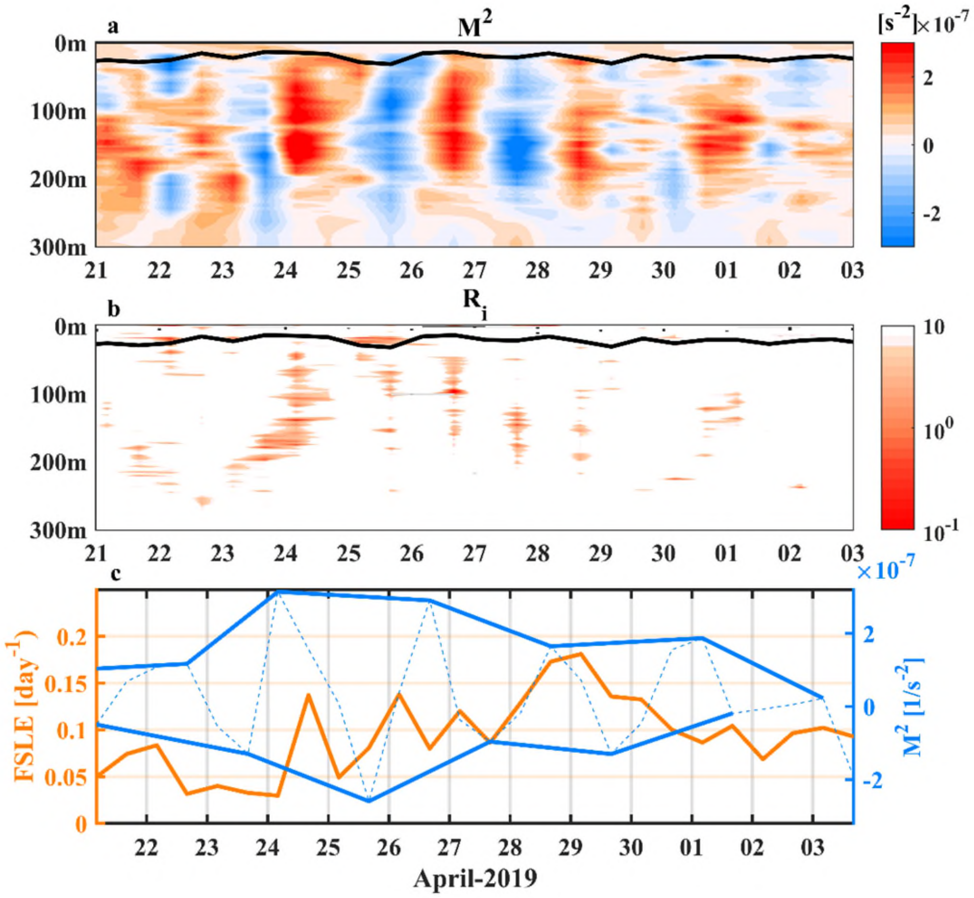

Figure S7. The lateral buoyancy frequency (M^2), Richardson number (R_i) and finite-size Lyapunov exponent (FSLE) at the front. **a** BGC-Argo-derived lateral buoyancy frequency during 21 April-3 May 2019. $M^2 = -\partial b/\partial s$, where s is the along-track direction. The black line represents the mixed layer depth. **b** BGC-Argo-derived Richardson number during 21 April-3 May 2019. $R_i = f^2 b_z / b_s^2$, where f and b are the Coriolis parameter and buoyancy, respectively. The black line represents the mixed layer depth. **c** Surface FSLE at the frontal region (red line) and the lateral buoyancy frequency (blue dashed line) at 100 m during 21 April-3 May 2019. Blue solid lines are the buoyancy frequency envelope.

10. Generalization to the subtropical Pacific. The generalization to the basin is very hand wavy and unconvincing, and probably not needed here. The results can stand alone without this. The author could in fact point out that the impact at the basin scale and global scale is still an open question. The conclusion, abstract (and maybe title) should be modified to reflect the changes in conclusions.

Response: Thank you for the helpful suggestion. We agree that the submesoscale impact at the basin scale is still an open question and have removed section 2.4 from the main text.

The generalization of our results to the Pacific gyre provides an applicable way to

estimate the effect of fronts on POC transfer efficiency based on satellite data, which definitely needs further studies to constrain uncertainties but also has the potential for future studies to explain POC variability in the mesopelagic ocean. In particular, we feel that the reviewers' comments #6 and #7 have been very helpful in improving this part. Based on these considerations, we modified our previous presentations and put this part as a discussion in the supplementary information. The title, abstract and conclusions have been modified accordingly. The details are listed below:

The title has been changed to '*Efficient biological carbon export to the mesopelagic ocean induced by submesoscale fronts*'.

In the abstract, the following sentences relative to the extrapolation results have been removed (Lines 27-31):

'Expanding the results to the subtropical gyre reveals a significant relationship between the Martin curve exponent coefficient (b) and front intensity. The average POC export flux at 1000 m may be underestimated by ~20-58% in the NPSG if the contribution of submesoscale fronts is ignored.'

For the previous section 2.4, the presentations have been modified. The section now has been moved to the supplementary information as Text S4.

In the discussion section, the following sentences have been added to link the observational results and Text S4 (Lines 396-405):

'The impact of fronts on POC transfer efficiency at the basin scale and global scale is still an open question. We combined previous studies that have estimated POC attenuation coefficients in the North Pacific and North Atlantic gyres (Table S4, Fig. S11). A strong correlation between the b value and GM was found ($b_{GM}=-3.78 \cdot GM+1.44$; $R^2=0.52$, $p<0.01$, Fig. 5). The derived relationship between the flux attenuation coefficient and the front intensity could be applied to large-scale applications. Estimations based on this relationship suggest that the average POC export flux at 1000 m may be underestimated by ~20-58% in the NPSG if the contribution of submesoscale fronts is ignored (Text S4).'

The following paragraph has been removed from the discussion section (Lines 330-341):

'The contribution of eddy intensification to the POC flux of the entire NPSG was estimated using eddy dataset. The intensifying cyclonic eddy occupied ~5.7% of the area of the NPSG (Fig. S7). This gives an additional POC flux of 0.12±0.03 mmolC m^{-2} d^{-1} by eddy intensifications in the NPSG in the euphotic zone. Considering the observed mean POC flux of 1.63 mmolC m^{-2} d^{-1} from previous studies in this region (Table S4), the eddy intensifications can increase the POC flux by ~7.4±1.8% in the NPSG. The contribution of front intensifications to the POC flux in the gyre is difficult to estimate, but an upper bound is attainable. Given that the fractional areas impacted by submesoscale structures are ~5.2% according to Liu et al.[42], the upper bound of the

POC flux that was increased by front intensification is 0.14±0.03 mmolC m^{-2} d^{-1}, which corresponds to ~9±1.9% of the POC flux at the base of the euphotic zone in the NPSG.'

In the Methods section, the following paragraph has been removed (Lines 561-568):
'Contribution of eddy intensification to the POC flux

The contribution of eddy intensification to the POC flux of the entire NPSG was estimated using the mesoscale eddy trajectory product from the Archiving, Validation and Interpretation of Satellite Oceanographic (AVISO) data. During 1993-2019, there were a total of 14539 cyclonic eddies, which, during the intensification stage, occupied ~5.7% of the area of the NPSG (Fig. S7). The POC export fluxes induced by eddy intensifications in the NPSG were calculated as $F_{EI}(Z_{Eu})\times A$, where A is the fractional area occupied by cyclonic eddies during the intensification stages.'

For figures, the original Figure 5 has been moved to the supplementary information, and the original Figure S7 has been removed.

Imprecisions on the approach that need clarification, issues with text and figures.
Section 2.1
11. Line 97 and onward. This paragraph about the position of the floats is a little tedious to read. Maybe the authors could either streamline or fold in some results in there as they describe the trajectory.

Response: Following the reviewer's suggestion, the descriptions of both the float position and float trajectory have been combined. In the new text, the paragraph you mentioned has been removed.

12. Line 107: "The euphotic depth (ZEu) was on average ~159 m within CE 1 and 161 m in ACE" is this difference really significant?

Response: We conducted a two-sample t test and found that the averaged Z_{Eu} for CE 1 did not statistically differ from that for ACE 1. We have mentioned this in the revised text (Lines 141-142):
'A two-sample t test suggests that the averaged Z_{Eu} for CE 1 was not statistically different from that for ACE 1.'

13. line 115: Correlations are reversed: change increasing to decreasing and vice versa.

Response: Done.

14. Line 116: "This result suggested that nutrients in the euphotic zone were enhanced by the uplift of isotherms within this cyclonic eddy17," Don't you mean that uplift occurs at the edge of CE instead of "within"?

Response: We mean that uplift occurs within the CE because the radial distribution of

nitrate concentration in CE 1 exhibited a higher level of nitrate in the CE 1 core than at its edge.

15. line 123: Trends are reversed: change positive to negative and vice versa.

Response: Done.

16. Text on the figure was blurry, small and hard to read in my version.

Response: We now use a larger font size for the text in Figure S1 and have updated all the figures with a higher resolution.

17. Zscm is used to discuss the changes in the different properties measured. Could you explain why? How to separate the effect of the eddy dynamics and the change in sampling depth due to variations in Zscm? Is the assumption that Zscm follows a density layer? Maybe using density instead of depth would be more insightful?

Response: We used Z_{scm} to discuss the biogeochemical effects of mesoscale eddies because mesoscale eddies have been demonstrated to significantly affect the subsurface chlorophyll maximum in the North Pacific subtropical gyre (Xiu & Chai, 2020). As shown in Fig. R3a and b, Z_{scm} mainly follows the density layer σ_θ=23.8 kg m^{-3} in CE 1 and σ_θ=23.3 kg m^{-3} in ACE 1. Due to the regulation of submesoscale frontal dynamics, the Z_{scm} at the CE 1 edge was shallower than the depth of σ_θ=23.8 kg m^{-3} from 28 April to 3 May 2019. Linear regressions along the density layer present analogous results to those along the Z_{scm} for Chl and POC. There are no significant correlations between the temperature and r/R because the along-isopycnal temperature was almost the same. The linear regression result for NO_3 along σ_θ=23.8 kg m^{-3} is opposite to that along Z_{scm} in CE 1 because the isopycnal reaches a deeper depth where the nitrate level is higher from 28 April to 3 May 2019. During this period, the isoline σ_θ=23.8 kg m^{-3} was below the euphotic zone depth where there is limited phytoplankton photosynthetic activity. In contrast, the Z_{SCM} was within the euphotic zone so that the impacts of the eddy dynamics on the biological processes can be well reflected. As such, we discussed the changes of different biogeochemical tracers using Z_{scm}.

Reference:

Xiu, P. & Chai, F. (2020). Eddies Affect Subsurface Phytoplankton and Oxygen Distributions in the North Pacific Subtropical Gyre. *Geophys. Res. Lett.* 47, e2020GL087037

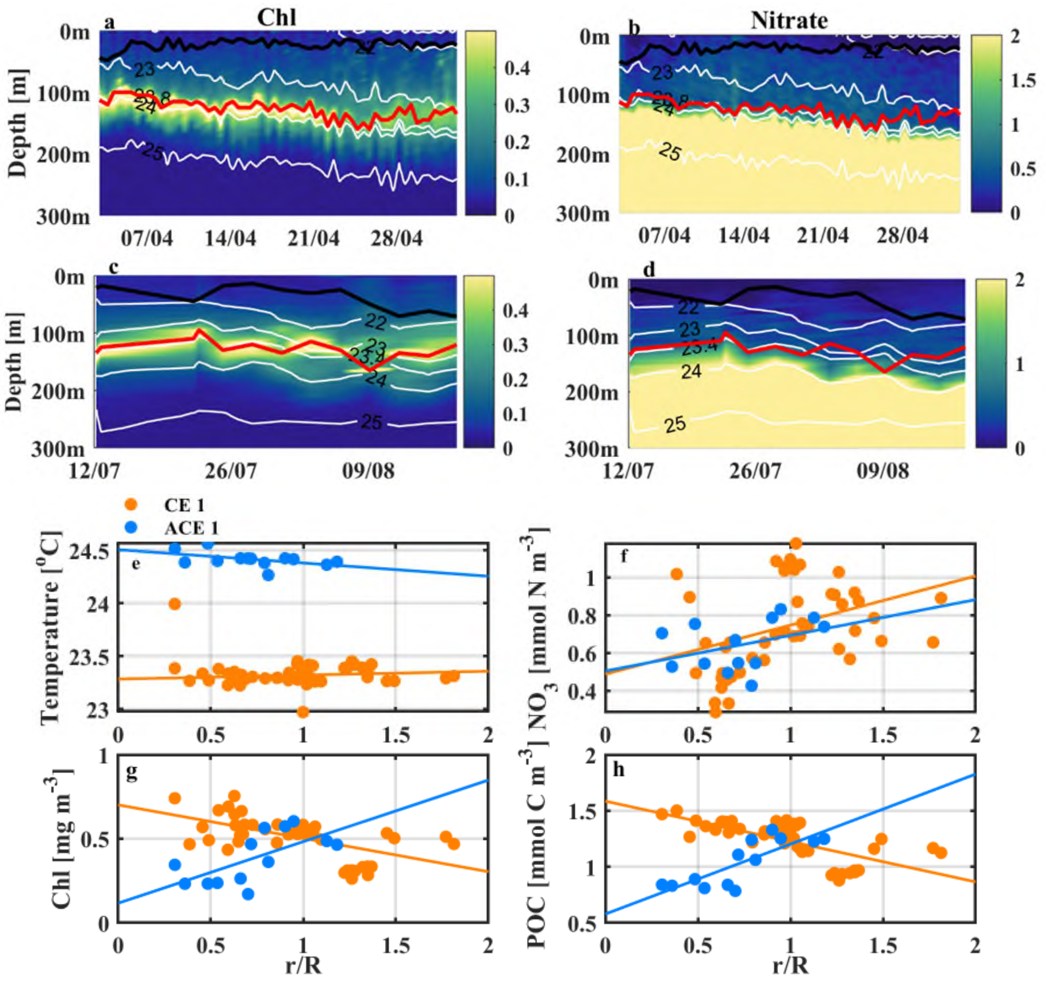

Figure R3. **a-b** Vertical distribution of (a) Chl and (b) NO_3 in CE 1. **c-d** Vertical distribution of (c) Chl and (d) NO_3 in ACE 1. Black, red and white lines in **a-d** represent the mixed layer depth, the depth of the subsurface chlorophyll maximum and the isopycnals, respectively. **e-h** Radial distributions of **c** temperature, **d** NO_3, **e** Chl and **f** POC along the density layer σ_θ=23.8 kg m^{-3} in (orange) CE 1 and σ_θ=23.4 kg m^{-3} in (blue) ACE 1. The solid lines indicate the linear regression lines.

Section 2.2

18. Please state clearly that you attribute change in integrated content over time to change in flux from the top, and detail and justify your assumptions.

Response: Thank you for the suggestion. In this study, the rate of change of the integrated POC from depth z to the mesopelagic layer is attributed to the net change between the POC flux supply from depth z and POC consumption between depth z and the base of the mesopelagic layer. Either changes in POC export or in POC consumption in the water column can affect this rate. The underlying assumption is that while the float drifted along the front, it sampled homogeneous water masses over time. We have clarified this point in the Methods section (Lines 495-507). Additionally, the assumption that the float sampled data as the time evolution of the front has been justified, as we replied to your comment #4.

19. More arguments could be provided to justify the reason for comparing the attenuation of a flux anomaly (FEI(z)) calculated with the float with the attenuation of the total POC flux from the literature.

Response: Thank you for the suggestion. For eddies, significant POC export is not sustained over their whole lifespan and is often associated with the physical dynamics of eddies (Zhou *et al*., 2020). The temporal evolution of biogeochemical processes in the eddy has been suggested to progress from nutrient injection, biomass buildup, and export enhancement during the eddy intensifying stage to biomass and export decline during the eddy decay stage (Sweeney *et al*., 2003; Zhou *et al*., 2020; Zhou *et al*., 2021). Observations have shown that intensifying eddies exhibit a large biological response, while eddies in the inactive stage do not show a significant biological response or high export flux (Sweeney *et al*., 2003; Buesseler *et al*., 2008). We assume that the magnitude of the export flux during the eddy decay stage is negligible, so that the flux anomaly approaches the total POC flux, and can be compared with those directly estimated from sediment traps or radioactive techniques recorded in the literature. Nevertheless, our estimated $F_{EI}(z)$ is a flux anomaly relative to the eddy decay stage, and it could underestimate the real POC export flux during the eddy intensifying stage. We have added this discussion to the text (Lines 227-235).

'For eddies, significant POC export is not sustained over their whole lifespan and is often associated with the physical dynamics of eddies[42,52]. Observations have shown that intensifying eddies exhibit a large biological response, while eddies in the inactive stage do not show a significant biological response or high export flux [29,31,42,52]. We assume that the magnitude of the export flux during the eddy decay stage is small, so that the enhanced net POC flux is considered as the approximation of the actual POC flux. The enhanced POC flux may underestimate the actual POC flux during the eddy intensifying stage as $F_{EI}(z)$ is a flux anomaly relative to the eddy decay stage.'

References:

Buesseler, K. O., Lamborg, C., Cai, P., Escoube, R., Johnson, R., Pike, S., Masque, P., McGillicuddy, D. & Verdeny, E. Particle fluxes associated with mesoscale eddies in the Sargasso Sea. Deep-Sea Res. II 55(10–13), 1426–1444 (2008).

Sweeney, E. N., McGillicuddy, D. J. & Buesseler, K. O. Biogeochemical impacts due to mesoscale eddy activity in the Sargasso Sea as measured at the Bermuda Time-Series Study (BATS). *Deep-Sea Res. II* **50**, 3017–3039 (2003).

Zhou, K., Dai, M. Xiu, P., Wang, L., Hu, J. & Benitez-Nelson, C. R. Transient enhancement and decoupling of carbon and opal export in cyclonic eddies. *J. Geophys. Res. Oceans* **125**, e2020JC016372 (2020).

Zhou, K., Benitez-Nelson, C. R., Huang, J., Xiu, P., Sun, Z. & Dai, M. Cyclonic eddies modulate temporal and spatial decoupling of particulate carbon, nitrogen, and biogenic silica export in the North Pacific Subtropical Gyre. *Limnol. Oceanogr.* **66**, 3508–3522 (2021).

20. Write P(z), E(z) on figure 3 to make it easier to understand.

Response: The symbols ‘P(z)’ and ‘$E(z)=\partial P(z)/\partial t$’ have been added to the new Fig. 4a.

21. line 153: Is «exponentially» appropriate? It seems linear.

Response: We agree. It is not appropriate here. The word ‘exponentially’ has been replaced with ‘gradually’.

22. line 162: The total increase in POC flux could be calculated by reconstructing the amount of remineralized POC from AOU and Redfield ratios.

Response: Thank you for the suggestion. We tried to quantify remineralized POC from AOU as suggested, but it may introduce more uncertainties to our results. The AOU multiplied by the Redfield ratios represents the heterotrophic respiration of the total organic carbon (TOC), 50-90% of which is dissolved organic carbon. Because the ratio of the POC to TOC and the Redfield ratios in the study region were not measured, we did not use the AOU to reconstruct the remineralized POC and only used AOU change to demonstrate possible changes in remineralization between different stages. We have added this discussion to the text (Lines 512-517):
‘The AOU multiplied by the Redfield ratios represents the heterotrophic respiration of the total organic carbon (TOC), 50-90% of which is dissolved organic carbon[48,58,59]. Because the ratio of the POC to TOC and the Redfield ratios in the study region were not measured, we did not use the AOU to reconstruct the remineralized POC and only used AOU concentration change over time to demonstrate possible changes in remineralization between different stages.’

References:

Abell, J., Emerson, S., & Renaud, P. Distributions of TOP, TON and TOC in the North Pacific subtropical gyre: Implications for nutrient supply in the surface ocean and remineralization in the upper thermocline. *J. Mar. Res.* **58**(2), 203–222 (2000).
Hansell, D. A., & Carlson, C. A. Biogeochemistry of total organic carbon and nitrogen in the Sargasso Sea: Control by convective overturn. *Deep-Sea Res. Part II* **48**(8–9), 1649–1667 (2001).
Kheireddine, M. et al. Organic carbon export and loss rates in the Red Sea. *Global Biogeochem. Cycles* **34**, e2020GB006650 (2020).

23. Y-scales of 3g and 3h are making it confusing, maybe 0-700 m could be used.

Response: The Y-scales of 3g and h have been limited to the range 0-700 m.

Section 2.3
24. L 183: Here, the front is defined by the temperature gradient. Would it be possible to find out whether the front is dynamically active and carrying POC at depth or not?

Response: We diagnosed the frontal dynamics using more physical parameters, such as the finite-size Lyapunov exponent (FSLE), lateral buoyancy frequency (M^2) and Richardson number (R_i). The concurrence of SST gradients, high FSLE, M^2 and small R_i suggests that the front was dynamically active (Fig. S5 and S7). Below the euphotic zone depth, we found that the POC concentration was high, which indicated that the intense submesoscale vertical velocities subducted high POC downward (Fig. S9). These results have been incorporated in the main text in Lines 256-264.

25. L184/185: Can we really speak of degradation/intensification, since the float crosses the front but doesn't stay there?

Response: According to the frontogenesis theory, when the front is enhanced, it will generate upwelling on the warm side of the front and downwelling on the cold side of the front. During the sampling period, the front experienced intensifying and decaying stages, while the float was continuously sampled along its warm side (not across the front). We conducted an experiment of particle tracking and a T-S characteristic analysis of the water mass to justify that the float did drift along one side of the front, on which relatively homogeneous water masses were sampled. As such, the float observations can be interpreted as a temporal signal due to the stage change of the front. Our hypothesis is that when the front intensifies, the dynamic and biogeochemical changes on each side are generally coherent. Please see the details in our reply to comment #4.

26. L 186 / Fig. S4e: Why is the gradient lower than the background during the decaying stage? Why not equal since the front goes back to background?

Response: Thank you for the insightful question. Fig. S4e has been updated to exclude extremely high GM values when calculating the background GM. It appears reasonable now.

27. L 188: Fig. S4g: How is the strain rate calculated and from which data product? You could physically define the front and add its position to your figures to make interpretation easier.

Response: The strain rate was calculated using satellite-derived altimetry data (sea surface height data) at a resolution of 0.25°·0.25°. We realized that the spatial resolution of the strain rate is inadequate to identify the frontal structure and have removed it in this revision. We have instead used the finite-size Lyapunov exponent (FSLE) to identify the dynamic active regimes of the front calculated from the sea surface height data. The along-track time series of FSLE and lateral buoyancy frequency (M^2) presented the co-located pattern of high FSLE and M^2, suggesting that the frontal region was in active submesoscale motion. Please see Fig. S7.

Methods

28. L 313: You could spell out bbp(700) and PAR again.

Response: Following the suggestion, the words ‘bbp(700) and PAR’ have been replaced with ‘particulate backscattering coefficients at 700 nm (b_{bp}(700)) and photosynthetically available radiation (PAR)’ .

Reviewer #3 (Remarks to the Author):

Efficient biological carbon export to the mesopelagic ocean induced by submesoscale fronts in subtropical gyres
Guo, M., Xing, X., et al.
Nature Communications
5 June 2023

This study presents data from BGC-Argo floats along with interpolated satellite remote sensing data to investigate the role of processes at the submesoscale in shaping carbon export. The authors find that, in the North Pacific subtropical gyre, processes at the mesoscale and submesoscale may play a significant role in transporting carbon from the surface to the interior ocean. In particular, they note that the previous assumption of a relationship of carbon attenuation with temperature may be better considered as a relationship between attenuation and SST gradient magnitude. Their suggested relationship could help better constrain carbon cycling in the subtropical gyres in numerical models.

I would like to thank the authors for a clearly-written manuscript with many high-quality figures. I enjoyed reading this manuscript and felt it presented a compelling case for considering carbon export at the submesoscale in the subtropical ocean. Carbon export is an important and timely topic, and I appreciate the care with which the authors present the data. I have a few concerns that I hope the authors will address in order to make the work more accessible to the reader. In addition, while the manuscript generally does a nice job at describing limitations with the work, there are a few additional constraints to consider, as detailed below. My major concerns are documented below; there were also several minor concerns that I think the manuscript would benefit from addressing.

Response: Thank you so much for the helpful comments and suggestions for this manuscript. We have addressed all your questions and concerns accordingly. We think this manuscript has been much improved with your constructive inputs. Please see the following details.

Major Concerns

Definition of the submesoscale front: The submesoscale front described in this manuscript is an interesting feature, but the manuscript does not yet convince me that I am seeing a density front and therefore that the resulting POC analysis is attributable to the feature. First, the front is diagnosed from SST and SST gradient. This ignores the potential contribution of salinity. To remedy this, I would like to see section plots (time versus depth) of the float's temperature and salinity over this period (April 21-May 3) to visualize the depth of this front.

Response: Thank you for the helpful suggestion. As the float did not cross the front but drifted along the front, we first plotted the vertical transects of temperature, salinity, and their anomalies obtained by subtracting the time averages from the instantaneous values (Fig. S6). As recorded by the float, the temperature was higher and salinity was lower at the frontal region than inside CE 1, suggesting a relatively light water mass consistent with the satellite-derived sea surface temperature. Because the float drifted along the front, the water mass sampled showed a coherent physical characteristic (Fig. S6e).

Secondly, the float was drifting on one side of a strong current (Fig. 2). According to the geostrophic balance, the float should be on the low-density side of the front, consistent with the observations.

Thirdly, we calculated the Richardson number R_i defined as $R_i = f^2 b_z / b_s^2$ (Fig. S7). Small R_i values ($R_i<2$) were found below the MLD to ~200 m during 21-28 April 2019, highlighting the ageostrophic character of the dynamical regime encountered by the float and the expected strong frontogenesis processes at play. As suggested by Siegelman et al. (2020), frontogenesis presented as a small R_i is prone to generate enhanced vertical velocities in the ocean interior below the mixed layer.

Lastly, we also showed the finite-size Lyapunov exponent (FSLE) and lateral buoyancy frequency (M^2) values (Fig. S7). All these dynamic variables suggest that the float was sampling on one side of the front and that the front experienced different stages during the sampling period.

The above information has been incorporated in the revised manuscript (Lines 247-261).

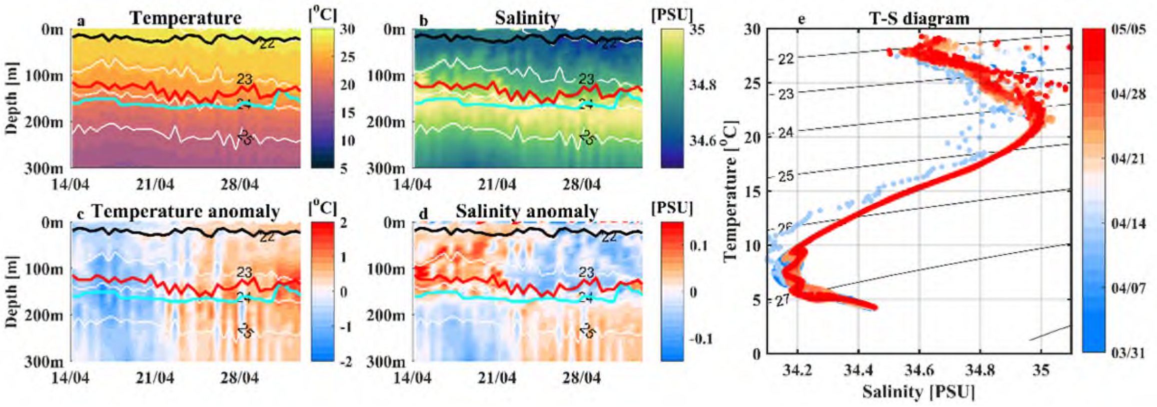

Figure S6. Physical characteristics of the front. Time series of **a** temperature, **b** salinity, **c** temperature anomaly and **d** salinity anomaly measured by float 2902753 from 1 April to 3 May 2019. The black, red and cyan lines indicate the mixed layer depth (MLD),

the depth of subsurface chlorophyll maximum (Z_{SCM}) and the euphotic zone (Z_{Eu}), respectively. The white lines are the isopycnals of 23, 24 and 25 kg m^{-3}. The temperature and salinity anomalies were defined as the instantaneous values subtracting the full time mean. **e** Temperature-salinity diagram with data points from float 2902753 from 1 April to 3 May 2019. Color indicates the sampling date.

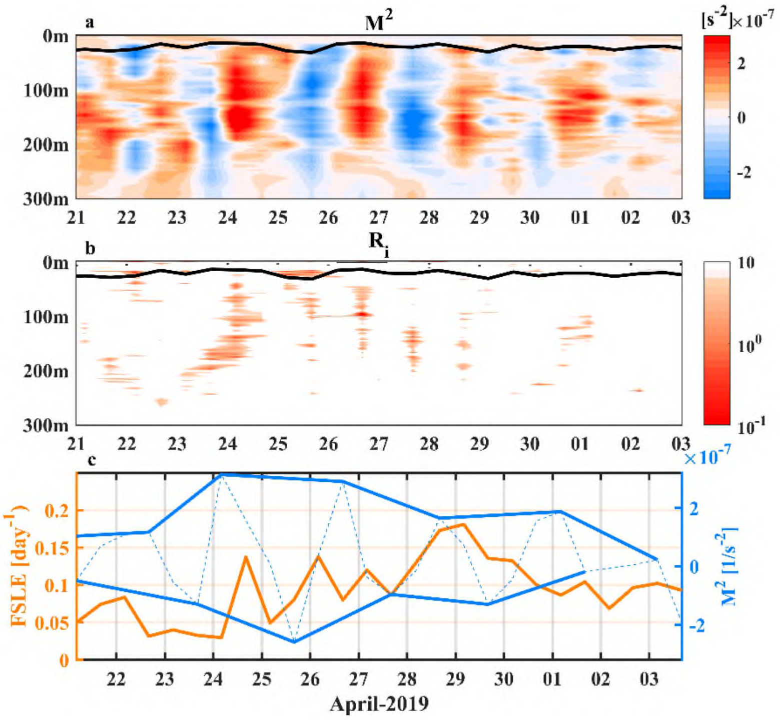

Figure S7. The lateral buoyancy frequency (M^2), finite-size Lyapunov exponent (FSLE) and Richardson number (R_i) at the front. **a** BGC-Argo-derived lateral buoyancy frequency during 21 April-3 May 2019. $M^2 = -\partial b/\partial s$, where s is the along-track direction. The black line represents the mixed layer depth. **b** BGC-Argo-derived Richardson number during 21 April-3 May 2019. $R_i = f^2 b_z / b_s^2$, where f and b are the Coriolis parameter and buoyancy, respectively. The black line represents the mixed layer depth. **c** Surface FSLE at the frontal region (red line) and the lateral buoyancy frequency (blue dashed line) at 100 m during 21 April-3 May 2019. Blue solid lines are the buoyancy frequency envelope.

In addition, Figure S4 is stated to be of vertical buoyancy frequency, but that is not possible as in a stable water column, N2 must solely be positive. I imagine the authors may have instead calculated lateral buoyancy frequency (M2), but the magnitude of the values shown are extremely high for this property; M2 values of strong submesoscale fronts in the Southern Ocean are of O(10^-7) (Siegelman et al. 2020). I urge the authors to check what they are calculating here. I do appreciate the inclusion of strain rate, and the authors could also consider using Finite Size Lyapunov Exponents (AVISO+, https://www.aviso.altimetry.fr/en/data/products/value-added-products/fsle-finite-size-lyapunov-exponents.html) to demonstrate the presence of the frontal structure.

Response: Thank you for pointing this out. Yes, we identified the frontal structure using the lateral buoyancy frequency but not the vertical buoyancy frequency. The lateral buoyancy frequency is calculated as $M^2=\partial b/\partial s$, where b is the buoyancy and s is the along-track direction. By converting the unit of s from km to m, we finally obtained a value of M^2 in a reasonable range, as reported in Siegelman et al. (2020). As such, Fig. S4 has been corrected. Please see the new Fig. S6.

The strain rate was calculated using satellite-derived altimetry data at a resolution of 0.25°·0.25°. We realized that the spatial resolution of the strain rate calculated based on satellite altimetry data is inadequate to identify the frontal structure and have removed it from Fig. S6.

Following the reviewer's suggestion, the FSLEs have been used to illustrate the frontal structure that float 2902753 encountered. As illustrated in Fig. S7, the float passed the regions with high FSLEs from 24 April to 1 May 2019. During this period, high FSLEs and M^2 were often collocated, which was consistent with Siegelman et al. (2020). Fig. S6 has been incorporated into the results, and the method using FSLEs to illustrate the frontal structure has been included in the Methods section with the following paragraph: '*The FSLE field*

We use the finite-size Lyapunov exponent (FSLE) to illustrate the frontal region encountered by the BGC-Argo. Daily FSLEs with a spatial resolution of 0.04° were obtained from the Archiving, Validation, and Interpretation of Satellite Oceanographic data (AVISO) service. The FSLE is defined as the inverse time of separation of two particles from their initial distance δ_0 to final distance δ_f (d'Ovidio et al., 2008). The particles were advected by altimetry velocities and their trajectories were computed by backward-time integration of the altimetry velocities. The FSLE has been suggested to be particularly suited to diagnose filamentary processes generated by mesoscale horizontal shear and strain deformation (d'Ovidio et al., 2008). Large backward FSLE values represent regions of strong submesoscale transport. (Calil et al., 2010; Siegelman et al., 2020). As such, the FSLE from AVISO has been employed to delineate submesoscale structures (Meunier & LaCasce, 2021; Guo et al., 2022). The FSLE time series was extracted along the path of float 2902753 to capture the strain field and was then compared to the lateral buoyancy frequency (M^2) sampled by the float.'

Sea level anomaly data: I greatly appreciate the authors' use of in situ data combined with remote sensing data. However, sea surface height data provided by AVISO typically has a footprint of approximately ¼ degree. While this can be manageable for mesoscale work, it is often too coarse for detection of submesoscale features. I am less familiar with satellite SST footprint, but I imagine it is of similar scale. I would like to see the authors discuss this potential limitation and how it might impact their results. I appreciate that there is some discussion of how the calculations from the SST gradient magnitude:attenuation rate relationship, and I think a discussion of how even remote sensing does not capture the submesoscale fully would fit in here. Addressing this concern, as well as my other concerns above and below, would help me feel more

comfortable in the extrapolation of data from two eddies to the full Northern Hemisphere subtropical gyres.

Response: We agree that the current spatial resolution of sea level anomaly data is insufficient for identifying submesoscale structures, so the SLA-calculated strain rate has been removed from frontal structure analysis. The SLA was only used to describe mesoscale eddies. The SST data we used have a 4 km resolution, which has the potential to demonstrate submesoscale structures.

The submesoscale fronts induced by frontogenesis are characterized by sharp density gradients. In much of the world's upper oceans, temperature variations govern the spatial gradients and seasonal variations of density, whereas salinity plays a secondary role. By analyzing a high-resolution dataset with similar horizontal resolutions for temperature and salinity, Rivière et al. (2019) suggested that over 90% of submesoscale density fronts can be identified using SST gradients. Therefore, the satellite-derived SST gradient as an indicator of submesoscale fronts has been suggested to be useful to investigate the spatiotemporal variation in submesoscale fronts. We have added the following sentences to clarify this point (Lines 80-85 in SI):
'In submesoscale frontal regions, frontogenesis within the developing stage leads to sharpening of the lateral density gradients[13,14,16,35]. In much of the world's ocean, temperature variations play a leading role in controlling the spatial near-surface density gradients, while salinity plays a secondary role[66]. Sea-surface temperature gradients derived from satellites at a 4-km resolution have been used to study fronts and filaments at scales of O(1 to 10) km[67,68].'

On the other hand, the SST gradients cannot cover all the density fronts. In regions that are strongly influenced by freshwater runoff and rain or have close-to-freezing surface temperatures, salinity variations govern the near-surface density gradients. Therefore, the spatial and temporal variation in the *b* value may be overestimated in this study. With more submesoscale structures identified in the region, a higher POC transfer efficiency is expected. We have added the following sentences to present the limitations of using SST gradients as an indicator of fronts and discussed their potential impacts on our results (Lines 105-108 in SI):
'Note that the GM cannot identify density fronts governed by salinity variations[70]. The spatial and temporal variation in the b value may be overestimated in this study. With more submesoscale structures identified in the region, a higher POC transfer efficiency is expected.'

References:

Rivière, P., Jaud, T., Siegelman, L., Klein, P., Cotté, C., Le Sommer, J., Dencausse, G. & Guinet, C. Sub-mesoscale fronts modify elephant seals foraging behavior. *Limnol. Oceanogr. Lett.* **4**, 193-204 (2019)
Johnson, G. C., Schmidtko, S., & Lyman, J. M. Relative contributions of temperature and salinity to seasonal mixed layer density changes and horizontal density gradients.

J. Geophys. Res. Oceans **117**, C04015 (2012).
Dong, J., & Zhong, Y. Submesoscale fronts observed by satellites over the northern South China Sea shelf. Dyn. *Atmospheres Oceans* **91**, 101161 (2020).
Gualtiero, S. J. & Mahadevan, A. Submesoscale-selective compensation of fronts in a salinity-stratified ocean. *Sci. Adv.* **4**,e1701504 (2018).

Minor Concerns
Line 26: unnecessary “however”

Response: We have deleted the word ‘however’.

Line 32: Is it clear that submesoscale motions would result in a net sink of CO2 in the subtropical gyres, or is there evidence that high DIC water from the mesopelagic could be brought up to the service and outgas?

Response: The carbon exported to the mesopelagic layer generally has a ventilation time scale of ~100 years. Previous studies have shown that deep water upwelling often occurs in regions with sharp topographic changes, such as the regions around sea mountains and islands. However, overall, the impacts of submesoscale processes on atmospheric carbon sinks are still an open question. Although some regional studies have shown strong carbon export associated with submesoscale processes, basin-scale studies are still lacking. Therefore, we have removed the phrase ‘that can potentially drive atmospheric CO_2 sinks’ in this sentence.

Line 41: “relatively fast of remineralization”: unnecessary “of,” and can you state what region(s) you are comparing remineralization rate against?

Response: The word ‘of’ has been deleted. We have added the phrase ‘compared to that at high latitudes’ at the end of this sentence.

Line 42: unnecessary and confusing “on the other hand”

Response: The phrase ‘on the other hand’ has been deleted.

Line 48: I suggest changing “leading to” to “resulting in”

Response: We have replaced ‘leading to’ with ‘resulting in’.

Lines 49-52: Consider adding citations to Mahadevan (2016) (doi: 10.1146/annurev-marine-010814-015912) and Klein & Lapeyre (2009) (doi: 10.1146/annurev.marine.010908.163704).

Response: The review papers by Mahadevan (2006) and Klein and Lapeyre (2009) have been cited.

References:

14 Mahadevan, A. The impact of submesoscale physics on primary productivity of plankton. *Annu. Rev. Mar. Sci.* 8, 161–184 (2016).
15 Klein, P. & Lapeyre, G. The oceanic vertical pump induced by mesoscale and submesoscale turbulence. *Ann. Rev. Mar. Sci.* 1, 351–375 (2009).

Lines 54-55: Consider providing a short summary of why plankton size distribution impacts POC export rates (I imagine because of differing contributions by the gravitational pump?)

Response: Both the gravitational pump by phytoplankton itself and marine food web dynamics can affect POC export rates. Serra-Pompei et al. (2022) used a global mechanistic size-spectrum model to investigate the relation between particulate export and plankton community metrics. They found that the size structure of the plankton community can affect export efficiency, as the size structure of the community can determine how efficiently energy and biomass reach larger organisms, and larger organisms tend to create particles (e.g., dense zooplankton fecal pellets) with faster sinking velocities. We have therefore added the sentences '*The vertical motions induced by the submesoscale front may also drive the vertical nutrient supply and stimulate the growth of large phytoplankton communities[32–36]; these large phytoplankton may sink fast and affect the carbon export efficiency by creating particles with fast sinking velocities through food web dynamics[16,36,37]*' in Line 62.

Reference:

Serra-Pompei, C., Ward, B. A., Pinti, J., Visser, A. W., Kiørboe, T., & Andersen, K. H. Linking plankton size spectra and community composition to carbon export and its efficiency. *Global Biogeochem. Cycles* **36**, e2021GB007275. (2022).

Line 56: Consider changing "However," to "Despite their potential importance to shape POC export," to make continuity stronger

Response: Done.

Lines 71-73: Have the authors seen Llort et al. (2018) (doi: https://doi.org/10.1002/2017JC012861). While they use a different method, I suggest their work was among the first to estimate carbon flux from BGC-Argo. I do agree that this manuscript's work looking at vertical attenuation is novel, so I suggest rephrasing this sentence to recognize previous efforts and clarify the novel elements of this manuscript.

Response: We thank the reviewer for this good suggestion. We acknowledge that Llort et al. (2018) identified subduction events and quantified the carbon export flux associated with submesoscale fronts by using BGC-Argo for the first time. We have

cited their paper and rephrased the sentence as follows (Lines 91-96):
'The BGC-Argo can be used to identify elevated POC in the mesopelagic layer and estimate the carbon flux associated with submesoscale features[20,23]. In this study, we further show the capability of using BGC-Argo floats to estimate the transfer efficiency of the carbon export flux in response to submesoscale fronts.'

Somewhere in the introduction, it would enhance your paper's reach to introduce the terms "mesoscale" and "submesoscale" dynamically - potentially defining them with the Rossby number or some other scaling.

Response: Thank you for the nice suggestion. We agree that the 'mesoscale' and 'submesoscale' terms should be introduced dynamically and have defined them using the Rossby number and Richardson number in the following sentence (Lines 44-47):
'Subtropical gyres have abundant mesoscale eddies that are characterized by a small Rossby number (R_0) and a large Richardson number (R_i), as well as submesoscale fronts that are characterized by O(1) R_0 and R_i[12-16]'.

Line 90: Please point the reader to Figure 1b at this point - I got quite lost reading until I was able to match up with the figure.

Response: We have cited Fig. 1b at the end of this sentence.

Lines 90-92: I am confused by this sentence - are you saying that you look at the slope of the isopycnals to determine intensification?

Response: We determined the intensification by comparing the SST gradient magnitude (GM) of the front with that of the background.

Line 93: Again, please cite Figure 1c.

Response: Done.

Lines 95-96: As someone who is used to working with BGC-Argo floats that are sampling at the traditional rate of 10 days, it would help to know earlier that the floats had more regular sampling. Please consider moving the content of this sentence to earlier in the paragraph.

Response: The content of this sentence has been moved to the introduction section in Lines 98-99.

Figure 1: Change blue and red dots to colors that are more visible on the chosen colorbar as the representations for NO3- and POC sampling stations.

Response: The blue and red dots have been replaced with black dots and yellow

pentagrams in Fig. 1a.

Line 111: What was the average depth of the subsurface chlorophyll maximum?

Response: The average depth of the subsurface chlorophyll maximum (Z_{scm}) is 117.0±9.9 m for CE 1 and 127.1±16.8 m for ACE 1, as shown in Fig. S1. We have specified the Z_{scm} values explicitly in the text. Please see line 157.

Lines 116-118: I generally agree with the statement that there are higher POC concentrations in the CE center than the edge, but I would appreciate some commentary on the POC peaks at r/R =~ 1 and 1.3 at 200 m.

Response: Thank you for pointing this out. The float at r/R = ~ 1 and 1.3 was right on filaments with active submesoscale dynamics. The high POC at the edge of CE 1 was likely due to the subduction of POC by intense submesoscale vertical motions. We have clarified this point in the main text in Lines 165-169:
'The POC at 200 m reached its peaks at the edge of r/R=~1 and 1.3, which is likely due to the subduction of POC by intense submesoscale vertical velocity at the edge[17,19,20,23]'

Line 122: Add a semicolon before "that is"

Response: Done.

Lines 124-125: Perhaps I am missing something obvious, but Figure 2b illustrates to me that POC concentrations are higher at the ACE center than the edge at 200 m?

Response: The original description is not correct. We have rephrased the sentence as follows:
'*The POC concentrations were lower at the Z_{SCM} and higher at 200 m in the ACE 1 center than at its edge, probably suggesting a slower vertical attenuation of POC in ACE1 center.*'

Figure 2: Why are standard deviations only shaded for some points?

Response: The shades are the 95% confidence intervals of the fitting results using a smoothing algorithm by Eilers (2003). The value of the confidence interval at both the left and right edges of the data is missing by using Eilers' algorithm. Therefore, the shades are not displayed at the boundaries of the data.

Reference:
Eilers, P. H. C. A Perfect Smoother. *Anal. Chem.* **75**(14), 3631?3636. (2003).

Line 130: Specifically cite Figure 1d.

Response: The citation 'Fig 1' has been changed to 'Fig. 1d'.

Line 142: Note that you are defining stable based on changes in r/R.

Response: The daily snapshots of BGC-Argo show that the float was likely trapped by the eddy (Fig. R1). During the observational period, the float drifted counterclockwise around the eddy center. By quantifying the position of the float relative to the eddy center, we found that the float stayed in a stable position relative to the eddy center. We therefore define the stable locations based on changes in r/R.

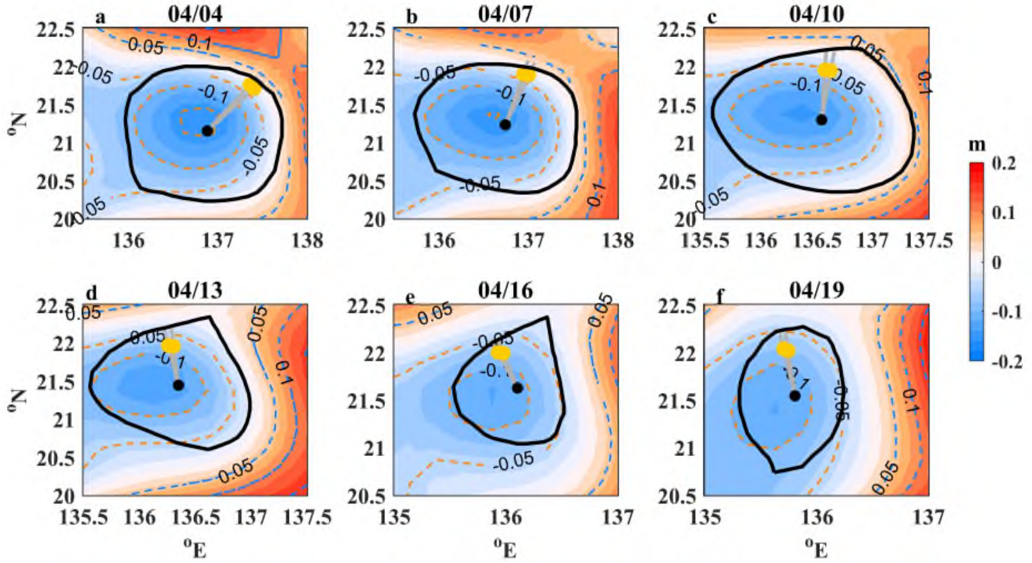

Figure R1. Daily snapshots of the float location in CE 2. Black and orange dots represent the eddy center and the float, respectively. Dashed contours are the isolines of SLA. Gray lines indicate the eddy radius. The black solid contour is the boundary of the eddy.

Lines 146-148: Can this linear least squares regression plot be included in the supplemental information to provide a sense of how different the calculated E(z) values are? Alternatively, one could include error bars on Figures 3c and 3d.

Response: Thank you for the suggestion. The linear least squares regression information has been included in the SI. Information on the rate of change of integrated POC, the standard error and the R^2 is shown in Table S6. Error bars were also added to this figure (now Fig. 4c, d).

Comment only: I really like the inclusion of AOU/oxygen utilization rate to investigate biological activity!

Response: Thank you for your positive comment!

Line 174: Consider mentioning that the Martin curve has long been used as the standard

for understanding POC attenuation across the global ocean for those who are not familiar with the concept.

Response: We have followed this suggestion to add the phrase '*Because the Martin curve has long been used as the standard for understanding POC attenuation across the global ocean*' in Lines 235-237.

Section 2.4: The manuscript appears to argue that the relationship between SST gradient magnitude and the b value is consistent across both mesoscale eddies and submesoscale fronts (as implied in Figure 4). However, the authors do a nice job in the introduction explaining why there are different rates of export by mesoscale and submesoscale processes, so I am not so sure that the two can be compared in this way. I'd appreciate some explanation or breakdown of the limitations of the SST GM:b relationship, particularly with respect to spatiotemporal scales.

Response: Thank you for the insightful comment. Yes, we put figure 4 in a broad perspective including all available data that were collected in different mesoscale and large-scale conditions. The assumption is that submesoscale processes are generally ubiquitous in the ocean and can occur in both mesoscale eddy core and edge regions. The submesoscale fronts at the eddy edge are often stronger than those at the eddy core, as demonstrated in Fig. 4. With the relationship in this figure, we can use one variable (GM) to estimate the *b* value, and we do not need to know whether the study region is in mesoscale and submesoscale conditions first.

Furthermore, because the spatial near-surface density gradients can be mainly explained by SST variations in much of the world's oceans, the satellite-derived SST GM is used to identify the spatiotemporal variations of submesoscale fronts in this study. However, submesoscale fronts formed by freshwater runoff, rain and in regions of near-freezing temperature cannot be identified by SST GM. Therefore, the POC transfer efficiency may be underestimated by extrapolating the SST GM: b relationship to the whole basin scale. As mentioned in our reply to main comment #3, we have acknowledged the limitations of this method in the revised text.

Figure S6: A different colorbar (perhaps cmocean's "algae" colorbar, https://matplotlib.org/cmocean/) would be a better choice for this figure.

Response: We thank the reviewer for the good suggestion. The cmoean's algae colormap works better for Fig. S6 (updated as Fig. S11) because the locations of the sample points are displayed more clearly compared to those in the previous Fig. S6:

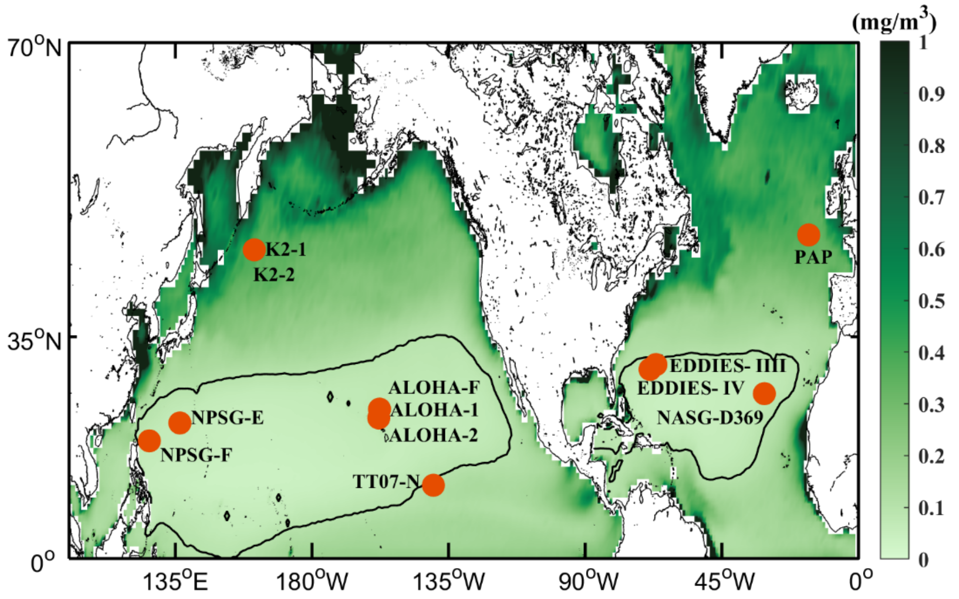

Figure S11. Locations of the POC flux data from previous studies (Fig. 5, Table S4) in subtropical gyres. The background color is the mean chlorophyll concentration from satellite data, and the black contour is the chlorophyll concentration of 0.1 mg m^{-3}.

Figure 5: Please consider using different colorbars for panels a and c versus b and d. At first glance, instinct is to compare a and b versus c and d. Different colorbars will make the story more clear and enhance understanding of your discussion.

Response: Done. The color bar for panels b and d is now different from that for panels a and c.

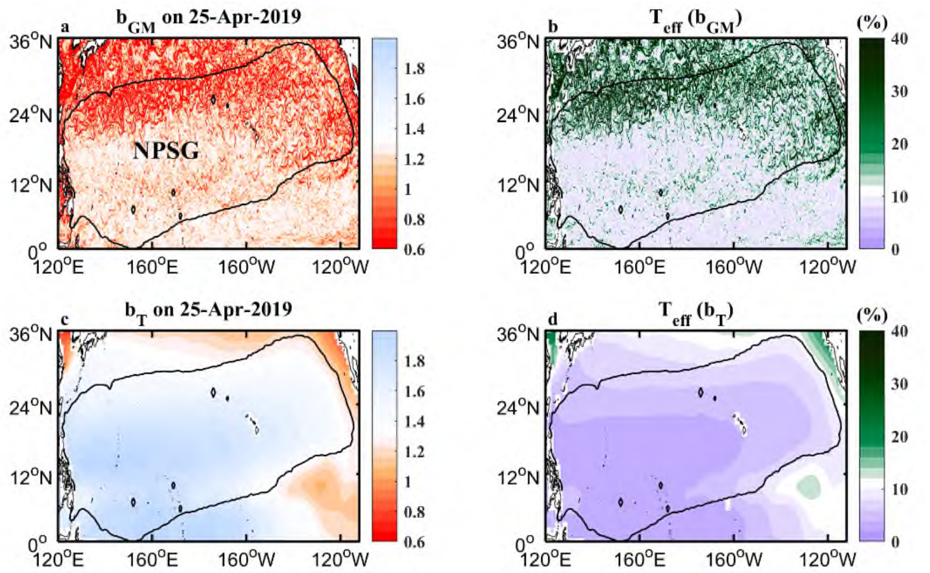

Figure S15. Transfer efficiency of POC flux at 1000 m estimated based on attenuation coefficient *b*. **a-b** Spatial distributions of attenuation coefficient *b* derived from GM (b_{GM}) on 25 April 2019 (**a**) and its transfer efficiency of POC flux at 1000 m (T_{eff}(b_{GM})) (**b**). **c-d** Spatial distributions of the attenuation coefficient *b* derived from the median

temperature of the upper 500 m (b_T) on 25 April 2019 (**c**) and its transfer efficiency of POC flux at 1000 m ($T_{eff}(b_T)$) (**d**).

Figure S7: Is it possible to put the eddies investigated in this study (CE 1 and 2) on panel b as a comparison? This could go a long way in solidifying the application of your parameterization.

Response: As suggested by reviewers 1 and 2, the generalization of the BGC-Argo results to the entire gyre and Figure S7 have been removed in this revision, as there may exist large uncertainties.

Lines 304-307: While I agree that BGC-Argo floats can be used for this type of investigation, as evidenced by the nice work in this manuscript, I would include a caveat that the current 10-day sampling regime employed by the Argo programme runs the risk of not capturing the submesoscale and mesoscale processes described in this paper.

Response: Thank you for the suggestion. We agree with the reviewer that the current 10-day sampling strategy of the BGC-Argo program is not feasible for capturing the effect of carbon export by fine-scale oceanic processes. We have included the caveat discussion in the text and highlighted the need for high-frequency profiling to address the challenges with the following sentences (Lines 431-438):
'The current 10-day sampling strategy employed by the BGC-Argo program runs the risk of not capturing carbon export induced by the fine-sale processes. This study demonstrated the unique application of BGC-Argo floats for studying these structures using a sampling strategy of twice a day for the operation of BGC-Argo. By deploying a large number of autonomous platforms sampling in high resolutions in the ocean, our understanding on ocean biological carbon pumps at different temporal and spatial scales will improve.'

Reviewer #4 (Remarks to the Author):

Review comment

The manuscript by Wang et al., used two high-frequency sampling BGC-Argo deployed in the western Pacific to investigate the impact of the eddies on the POC export. The authors reported enhanced POC export in the regions affected by intensifying submesoscale fronts and further meta-analysis of a global compiled dataset revealed a prevailing relationship between transfer efficiency and the strength of submesoscale front (indicative of surface temperature gradient). Finally, the authors upscaled the contribution of eddies to the carbon export in the entire northern subtropical Pacific gyre by synthesizing the frequency of eddy occurrence.

However, I find several noteworthy loopholes in the BGC-Argo data analysis, specially related to the observed AOU rate, and its discrepancy with the POC rate. See my explicit comments below:

Response: We thank the reviewer for providing helpful suggestions and pointing out the discrepancy between the AOU and POC results. We have responded to these questions and comments point-by-point below. We find that the paper is significantly strengthened by addressing these issues.

1) Unexpected, negative change of AOU and the discrepancy between AOU and POC rate:
Figure 3 shows the author derived negative values for the time rate of AOU in many cases, the implication being that twilight zone oxygen increases over time. This tendency is somewhat surprising to me, as we generally expect a declining trend of oxygen over time below the euphotic zone resulting from biological respiration, regardless of the relative change in upper POC flux.

Furthermore, the reported magnitude of AOU rate appears to be unrealistically high. The depth-integrated AOU rates between 150m (or 170m) and 900m fall within the range of 250-500 mmol m^{-2} d^{-1}, equivalent to 90-180 mol m^{-2} yr^{-1}. For comparison, the global range of upper carbon export is only typically 1-4 mol C m^{-2} yr^{-1}. These extremely high AOU rates also are sharply analogous relative to export flux solved from POC inventory, which falls within the range of 1-2 mmol C m^{-2} d^{-1}.

I suspect there are two possible reasons for the discrepancies. It is well known that the evolution of eddy typically results in a significant displacement of water masses. Thereby, observed AOU and POC change rates along the isobars may reflect a confounding result of both biological and physical processes. Therefore, to better isolate the biologically-induced tracer change that the authors are interested in, it would be more appropriate to quantify the time rate change of AOU (and POC as well) along

with the isopycncal surfaces.

The second possible reason is the float didn't exactly track the same water mass and may gradually move towards a region featured with a higher pre-formed oxygen concentration. To rule out this possibility, a more rigorous examination of the float hydrographic data and comparison with background spatial oxygen gradients from existing gridded products (e.g., WOA and GO-BAI oxygen product) and POC gradients (e.g., climatology of remotely sensed POC) is necessary.

Response: Thank you for pointing this out and providing helpful suggestions to examine this issue. For eddies, the changes of the POC inventory, AOU and O_2 during the intensifying and mature stages of CE 2 were actually in agreement with what the reviewer expected. As illustrated in Fig. R1, the mesopelagic POC inventories decrease and the AOU increases over time during both the intensifying and mature stages. Meanwhile, the O_2 decreased over time. All these results support the mechanism by which the POC inventories were consumed by heterotrophic respiration. There was elevated POC export during the intensifying stage of CE 2, as the rate of change of POC inventories was larger than that during the mature stage. As demonstrated by the larger rate of change of AOU in the intensifying stage than in the mature stage, biological respiration was also elevated during the intensifying stage.

In the frontal region, the AOU change rates are negative, as pointed out by the reviewer. The following have been conducted to examine this issue.

Firstly, we prove that the float sampled a similar water mass during the period. According to the frontogenesis theory, when the front is enhanced, it will generate upwelling on the warm side of the front and downwelling on the cold side of the front. During the sampling period, the front experienced intensifying and decaying stages, while the float was continuously sampling along its warm side (not across the front). As such, the float observations can be interpreted as a temporal signal due to the stage change of the front. Our hypothesis is that when the front intensifies, the dynamic and biogeochemical changes on each side are generally coherent. The spatial distributions of satellite temperature data have demonstrated that the float was actually on one side of the front. To justify that the float drifted along the front, we conducted a numerical particle tracking experiment by releasing particles at the beginning position of the float (Fig. S14). The particle tracking results suggest that the float drifted along the front in a quasi-Lagrangian manner (Text S3). In addition, the T-S diagram demonstrates a coherent physical characteristic of the water masses along the front, suggesting that homogeneous water masses have been sampled by the float (Fig. S6).

Secondly, we followed the reviewer's suggestion to compare the background O_2 gradients and sea surface POC (remote sensing product) with those derived from BGC-Argo (Fig. R2 and R3). The background O_2 from GOBAI did not change significantly compared with the float data. During the sampling period, the depth-averaged O_2 first

increased, then decreased, and then increased again without a clear trend. Satellite POC data also showed a variable pattern without a clear trend (Fig. R2 and R3). These results indicate that the background condition did not change largely in space during the float's movement.

We thus think there may be two possible reasons for the negative change of AOU over time along the front. First, there are errors in estimating the rate of change of both the AOU and POC inventories, as the float did not sample exactly the same water mass but similar ones. Due to atmosphere-ocean oxygen disequilibrium, AOU has been suggested to overestimate true oxygen utilization (Sulpis et al., 2023; DeVries & Holzer, 2019; Duteil et al., 2013; Holzer, 2022; Ito et al., 2004; Koeve & Kähler, 2016). The overestimation of O_2 utilization for each individual water mass brings larger uncertainties in the AOU rate estimation compared to the rate of POC inventory estimation (see larger standard deviations in Fig. R4b compared to Fig. R4a). Second, the negative AOU trend may reflect that biological respiration decreased with time in the study region. The sampled front was close to the mesoscale eddy CE 1, and the float was actually inside CE 1 on March 31-April 14, during which CE 1 weakened (Fig. R5). After that, the float moved from CE 1 to the frontal region during April 21- May 3; therefore, the condition in the frontal region may be connected with that inside CE 1. While the float was inside CE 1, the eddy weakened gradually with decreased POC export and decreased AOU, as similarly demonstrated in CE 2. The water mass associated with the float moved from CE 1 to the frontal region and may thus show a gradually decreasing AOU trend, which we call the mesoscale background. Superimposed on this mesoscale background, frontal dynamics further modified the POC and AOU in the water mass. Therefore, we think this negative AOU trend may result from the mesoscale background, as both stages of the front showed a similar negative trend. To identify the impact of front, we removed this background AOU trend for both front stages in this revision. By removing the background AOU trend, the rate of change of AOU shifted toward positive values. Overall, the net rate of change of AOU (defined as $E_{EI}(z)$-$E_{EM}(z)$ or $E_{FI}(z)$-$E_{FM}(z)$) is much larger for CE 2 than for the front (Fig. 4e and f), which is consistent with the result of high POC transfer efficiency along the front and low POC transfer efficiency in CE 2. Based on the analysis above, we present the net rate of change of AOU in Figure 4 in this revision.

Regarding the high AOU-based respiration rate, we checked our calculations and compared them with a previous study by Kheireddine et al. (2020). Comparing the depth-averaged AOU rate ($\frac{\partial(\frac{1}{Z_{base}-Z}\int_{Z_{base}}^{Z} AOU dz)}{\partial t}$) with that estimated in the Red Sea (Kheireddine et al., 2020), we find that the rates of change of the averaged AOU are in a similar range between the two studies (the maximum AOU rate is 0.72 mmol O_2 m^{-3} d^{-1} in this study and 0.24 mmol O_2 m^{-3} d^{-1} in the Red Sea). In Kheireddine et al. (2020), the mesopelagic zone was divided into 5 layers, and the averaged AOU rate was indicated to represent the average remineralization rate in each layer. By multiplying the Redfield value (ΔC: $\Delta O_2 \approx 0.688$) and the thickness of each layer, the AOU-based

remineralization rate in their study was calculated, i.e., 0.24 mmol O_2 m^{-3} d^{-1} * Redfield *66 m=10.8 mmol C m^{-2} d^{-1} (3.9 mol C m^{-2} y^{-1}). The AOU-based remineralization rates for the first layer of CE 2 and the front are 24.8 mmol C m^{-2} d^{-1} (9 mol C m^{-2} y^{-1}) and 7.8 mmol C m^{-2} d^{-1} (2.9 mol C m^{-2} y^{-1}), respectively, in this study (Table R1).

However, the AOU-based remineralization rate was still higher than the POC loss rate. In the Red Sea, the AOU-based remineralization rate was reported to be 25-fold higher than that estimated from the change of POC inventory (Kheireddine et al., 2020). The discrepancy is likely because the AOU reflects a consumption of total organic carbons, in which 50-90% have been reported to be dissolved organic carbon (Abell et al., 2000; Hansell & Carlson, 2001). In agreement with Kheireddine et al. (2020), we find that the POC loss rate accounts for a small proportion of organic carbon remineralization. Analyses of global and regional ocean carbon budgets have identified conspicuous imbalances in the mesopelagic zone by up to an order of magnitude between heterotrophic carbon demand and upper layer carbon supply (Burd et al., 2010; Steinberg et al., 2008; Giering et al., 2014; Boyd et al., 2019). Fully addressing this issue requires further field work and is beyond the scope of this study. Therefore, using the AOU rate to quantify the POC remineralization rate is not feasible here. We only used the difference of the AOU rate between the two stages to qualitatively demonstrate whether the remineralization rate was increased or decreased. To make this clear, we now present the averaged AOU rate instead of the integrated AOU rate in Figure 4.

To present the results of averaged AOU, we have added the following sentences in the main text (Lines 209-213 and 271-273):
'In addition, the net apparent oxygen utilization (AOU) changing rate was positive in the upper mesopelagic layer because the AOU rate was larger during the intensifying stage than the mature stage (Fig. 4e). This may imply enhanced POC consumption by microbial activity during the intensifying stage.'
'Different from the eddy, the net AOU changing rate was very small in the mesopelagic layer (Fig. 4f). The small net AOU changing rate implies relatively stable POC consumption rates during both the intensifying and decaying stages.'

To clarify why we used the averaged AOU rate instead of the AOU-based remineralization rate and how we calculated the averaged AOU rate, we have added the following paragraph to the Methods section (Lines 512-522):
The AOU multiplied by the Redfield ratios represents the heterotrophic respiration of the total organic carbon (TOC), 50-90% of which is dissolved organic carbon[48,58,59]. Because the ratio of the POC to TOC and the Redfield ratios in the study region were not measured, we did not use the AOU to reconstruct the remineralized POC and only used AOU concentration change over time to demonstrate possible changes in remineralization between different stages. The averaged AOU from the base of the mesopelagic layer (900 m) to an upper mesopelagic depth z was calculated as $\frac{1}{(900\ m - Z)}\int_{900\ m}^{z} AOU \cdot dz$ *(z= [Z_{Eu}, 200 m, 300 m, 400 m, 500 m]). The net rate of*

change of AOU defined as the difference of the time rate of AOU between the intensifying and mature (decaying) stages of the eddy (front) was thus estimated using the averaged AOU.

References:

Abell, J., Emerson, S., & Renaud, P. Distributions of TOP, TON and TOC in the North Pacific subtropical gyre: Implications for nutrient supply in the surface ocean and remineralization in the upper thermocline. *J. Mar. Res.* **58**(2), 203–222 (2000).

Burd, A. B. et al. Assessing the apparent imbalance between geochemical and biochemical indicators of meso- and bathypelagic biological activity: what the @$é! is wrong with present calculations of carbon budgets? *Deep-Sea Res. II* **57**, 1557–1571 (2010)

Boyd, P.W., Claustre, H., Levy, M. et al. Multifaceted particle pumps drive carbon sequestration in the ocean. *Nature* **568**, 327–335 (2019).

DeVries, T., & Holzer, M. Radiocarbon and helium isotope constraints on deep ocean ventilation and Mantle- 3He Sources. *J. Geophys. Res. Oceans* **124**(5), 3036–3057 (2019).

Duteil, O., Koeve, W., Oschlies, A., Bianchi, D., Galbraith, E., Kriest, I., & Matear, R. A novel estimate of ocean oxygen utilization points to a reduced rate of respiration in the ocean interior. *Biogeosciences* **10**(11), 7723–7738. (2013).

Giering, S., Sanders, R., Lampitt, R. et al. Reconciliation of the carbon budget in the ocean's twilight zone. *Nature* **507**, 480–483 (2014).

Hansell, D. A., & Carlson, C. A. Biogeochemistry of total organic carbon and nitrogen in the Sargasso Sea: Control by convective overturn. *Deep-Sea Res. Part II* **48**(8–9), 1649–1667 (2001).

Holzer, M. The fate of oxygen in the ocean and its sensitivity to local changes in biological production. *J. Geophys. Res. Oceans* **127**(8), e2022JC018802. (2022).

Ito, T., Follows, M. J., & Boyle, E. A. Is AOU a good measure of respiration in the oceans? *Geophys. Res. Lett.* **31**(17), L17305. (2004).

Koeve, W., & Kähler, P. Oxygen utilization rate (OUR) underestimates ocean respiration: A model study. *Global Biogeochem. Cycles* **30**(8), 1166–1182. (2016).

Kheireddine, M. et al. Organic carbon export and loss rates in the Red Sea. *Global Biogeochem. Cycles* **34**, e2020GB006650 (2020).

Steinberg, D. K., Van Mooy, B. A. S., Buesseler, K. O., Boyd, P. W., Kobari, T., & Karl, D. M. Bacterial vs. zooplankton control of sinking particle flux in the ocean's twilight zone. *Limnol. Oceanogr.* **53**(4), 1327–1338. (2008).

Sulpis, O., Trossman, D. S., Holzer, M., Jeansson, E., Lauvset, S. K., & Middelburg, J. J. Respiration patterns in the dark ocean. *Global Biogeochem. Cycles* **37**, e2023GB007747. (2023).

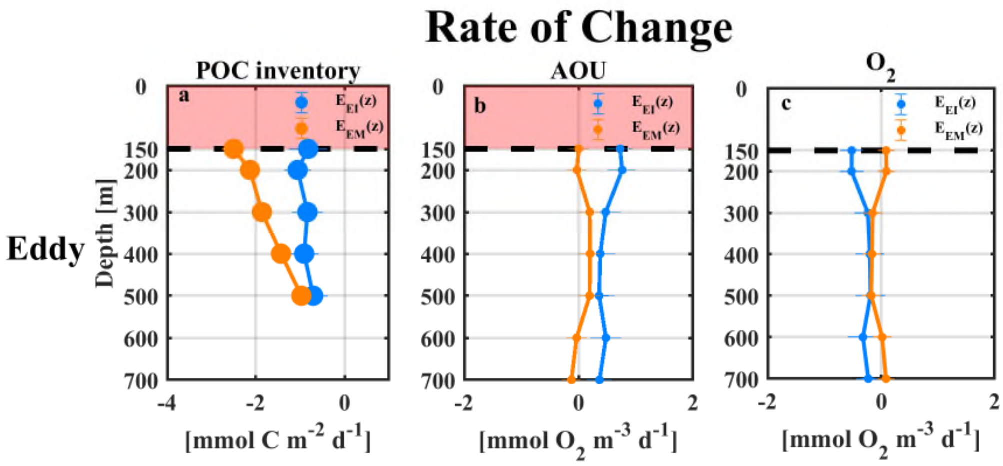

Figure R1. The rates of change of the POC inventory, AOU and O_2 during the intensifying and mature stages of CE 2.

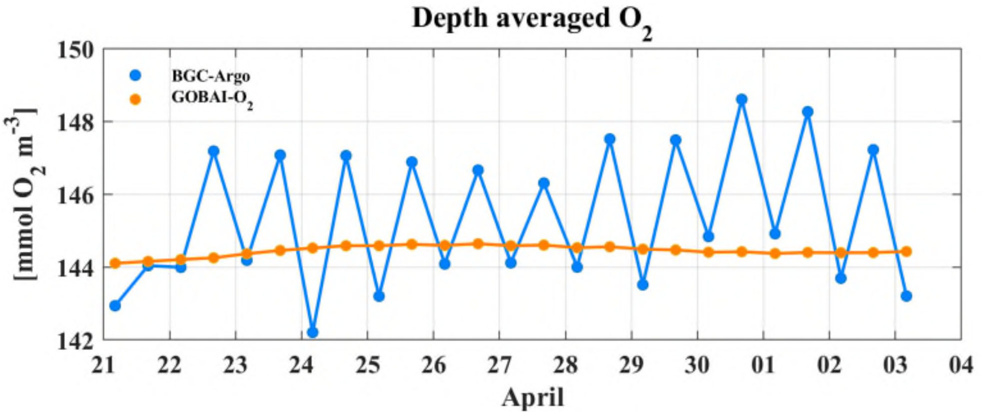

Figure R2. Comparison of the O_2 between GOBAI and BGC-Argo along the float trajectory. The O2 concentrations were averaged between 0-900 m for both the GOBAI and BGC-Argo data.

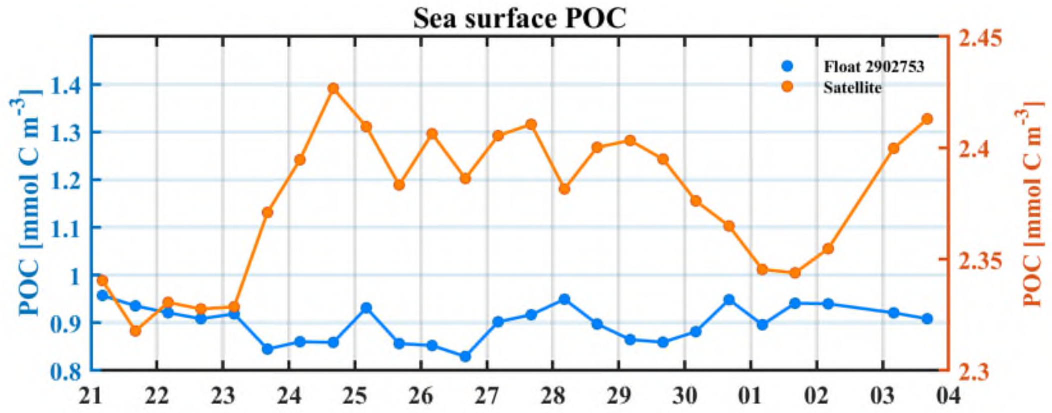

Figure R3. Comparison of the sea surface POC between the climatology of remote sensing and the BGC-Argo float measurement. The remote sensing POC data were derived from MODIS (https://oceancolor.gsfc. nasa.gov/l3/. References) and averaged from 2002 to 2022.

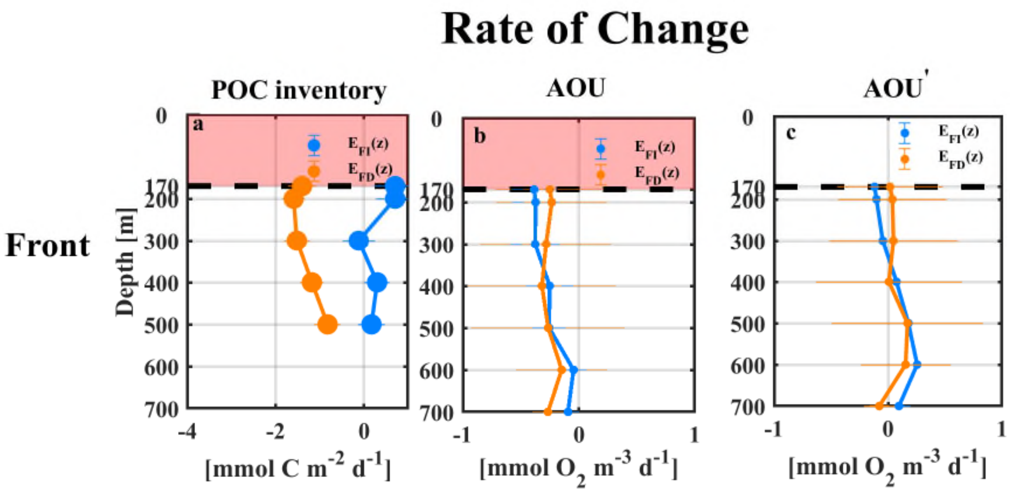

Figure R4. The rates of change of the POC inventory, AOU and AOU subtracting the background AOU trend (denoted as AOU') during the intensifying and decaying stages of the front.

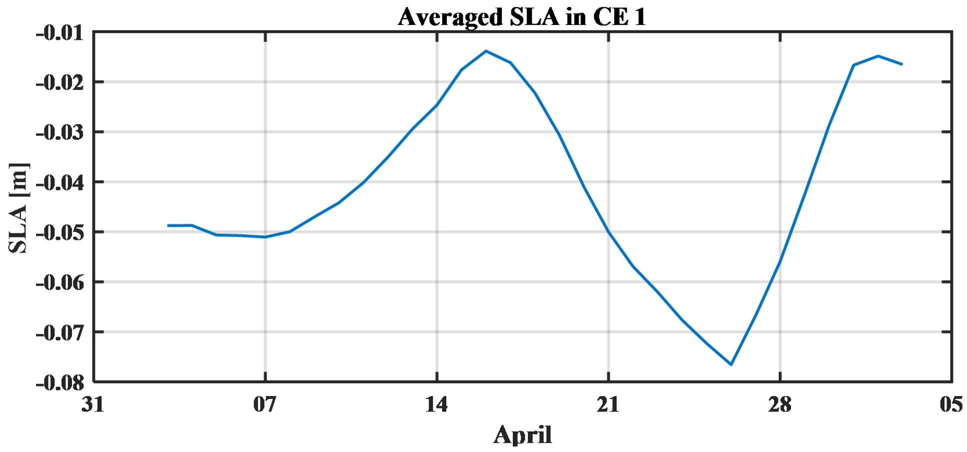

Figure R5. Time series of the averaged sea level anomaly (SLA) in CE 1.

Table R1. AOU-based remineralization rate

	Averaged AOU rate [mmol O_2 m^{-3} d^{-1}]	Redfield Ratio referred to Kheireddine et al.	Thickness [m]	Remineralization rate [mmol C m^{-2} d^{-1}]	Remineralization rate [mol C m^{-2} y^{-1}]	refs
Red Sea	0.24	0.688	66	10.8	3.9	Kheireddine et al. 2020
CE 2	0.72	0.688	50	24.8	9	This study
Front	0.38	0.688	30	7.8	2.9	This study

2) Is it reasonable to interpret the decreasing trend of POC inventory as an enhanced POC export?

In principle, when there is an enhanced upper layer POC export event, we usually expect to observe an increase in the POC inventory in the mesopelagic zone, along with a simultaneous elevation of respiration (reflected by an increase in AOU).

Nevertheless, the author observed a decline in POC inventory. As the authors mentioned, the POC inventory change in the twilight zone reflects a net balance between the supply term from the upper layer and removal due to the local respiration (net POC inventory change= POC_sinking_upper- respiration). There are several possible scenarios that can explain the POC inventory decline, including:

• Enhanced respiration outpaces the concurrent elevated upper layer POC export (the scenario that authors have speculated).
• Elevated respiration occurs in the context of a constant upper layer POC export.
• A decline in the POC export accompanied by constant respiration).

It is challenging to rule out other possibilities unless the authors have the information on the respiration rates, which would enable to back-calculate the exact magnitude of upper layer POC export (POC_sinking_upper = respiration+ net POC inventory change).

Response: Thank you for the constructive comment. In a stable environment, POC export would result in an increase in the mesopelagic POC inventory and an elevation in respiration. However, there are also studies showing different patterns between POC export, POC inventory, and AOU change. As reported in the Red Sea (Kheireddine et a., 2020), there is an increase in the mesopelagic POC inventory (due to the net POC supply) accompanied by a decrease in AOU (due to ventilation) during the winter phytoplankton bloom period. They also showed a decrease in the POC inventory along with an increase in AOU (due to enhanced bacterial remineralization) during the spring-summer period when restratification was established.

The mesoscale and submesoscale events investigated in this study occurred in late spring when the mixed layer became shallow. During this season, mesopelagic POC consumption may outpace the upper layer supply in the NPSG, resulting in a decreasing trend of the POC inventory analogous to that in the spring-summer period in the Red Sea (Kheireddine et al., 2020). To support this point, the seasonal variation of mesopelagic POC inventories in the study region is investigated using BGC-Argo 2902748, 29002753 and 2902756. As illustrated in Fig. R6, the mesopelagic POC inventories decreased during spring-winter. In particular, there was a fast POC loss from April to May. In addition, a climatological monthly POC inventory between 150-400 m was investigated at the ALOHA station. A decreasing trend of the POC inventory between 150 and 400 m was also found from April to June (Fig. R7). The consistency of the decreasing trend of the POC inventory between BGC-Argo and in situ observations suggests that heterotrophic remineralization outpaces the carbon supply in this season.

Nevertheless, we agree with the reviewer about the equation here (net POC inventory change= POC_sinking_flux - Respiration). As discussed in our reply to comment #1,

using AOU rate to quantify the POC remineralization rate is not feasible. So we only use the change of AOU rate between two stages to qualitatively demonstrate whether the respiration rate increased or decreased. By comparing two different stages of CE 2, we observed increased net POC inventory during the mature stage of the eddy. Meanwhile, we also showed that the AOU increased, probably indicating increased respiration during the CE 2 mature stage. Therefore, according to the equation (net POC inventory change= POC_sinking_flux - Respiration), the POC sinking flux from top should be enhanced during the mature stage of the CE 2.

For the front, we observed a larger POC inventory during the intensifying stage than that during the decaying stage, and we didn't see a significant change of AOU between the two stages. We thus expect enhanced POC export flux from top during the intensifying stage of the front.

It noteworthy that this kind of calculation indeed underestimated the actual POC export flux, as it only represented the enhanced export between the two stages. We have included the above discussion in the revised text (Lines 213-221):

'The POC inventory change in the water column is the combined result of the POC flux supplied from the top and the POC consumption in the water column. It is thus very likely that the POC export flux increased during the intensifying stage, given that both the change rate of POC and averaged AOU were larger during the intensifying stage than during the mature stage.

The enhanced net POC flux during the eddy intensifying stage was then calculated as $F_{EI}(z)=E_{EI}(z)-E_{EM}(z)$*. Note that this flux should be smaller than the actual POC export flux, as POC consumption also increased during the intensifying stage compared with the mature stage (presented as a positive net AOU rate) (Fig. 4e).'*

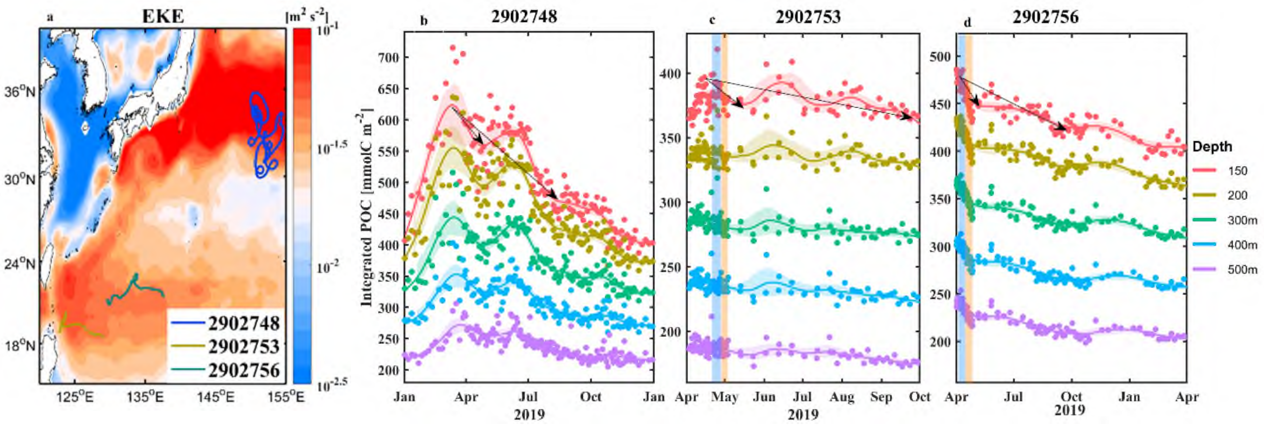

Figure R6. Integrated POC stocks estimated from BGC-Argos in the mesopelagic zone. **a** The trajectories of floats 2902748, 2902753 and 2902756. The background color is the eddy kinetic energy (EKE) averaged from 1993-2020. **b** Integrated POC stocks estimated from float 2902748. **c** The same as **b** but from float 2902753. **d** The same as **c** but from float 2902756. The POC stocks were integrated from the base of the mesopelagic zone to depth z, where z = 150 m, 200 m, 300 m, 400 m and 500 m.

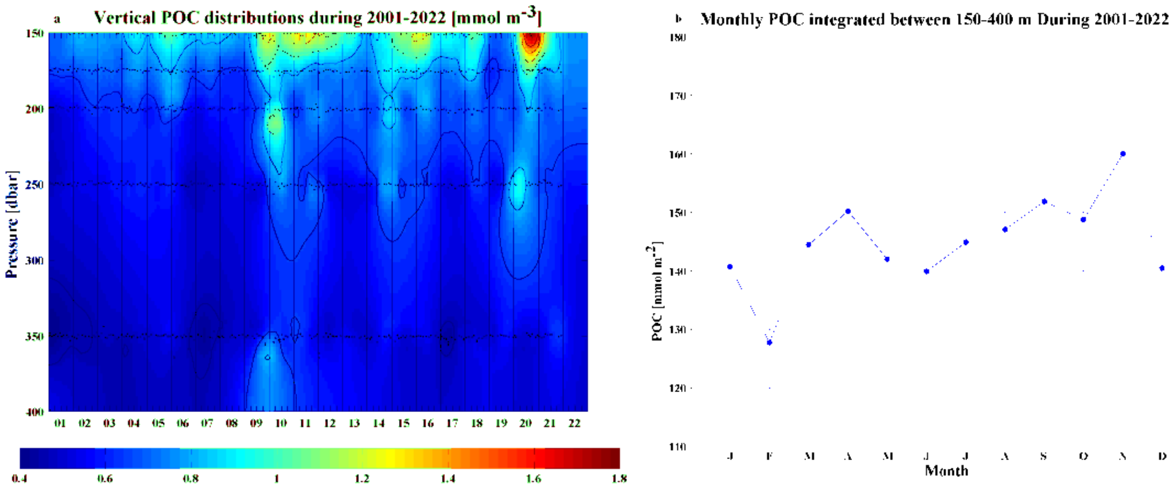

Figure R7. Climatological monthly POC stocks at the ALOHA station. **a** Vertical distribution of POC concentration during 2001-2022 at ALOHA. **b** Climatological monthly POC stocks integrated from 400 m to 150 m during 2001-2022. The figure was downloaded from https://hahana.soest.hawaii.edu/hot/hot-dogs/bcontour.html

Other comments:

Line 32: the eddy-induced upwelling also brings subsurface water rich in DIC into the surface, which may drive the CO2 outgains.

Response: Thank you for pointing this out. The impacts of submesoscale processes on atmospheric carbon sinks are still an open question. We have removed the phrase 'that can potentially drive atmospheric CO_2 sinks' in the revised manuscript.

Line 55: it may be worth mentioning the potential impact of eddy-driven carbon export on helping reconcile the long-standing paradox regarding the imbalance between the upper layer carbon supply and deep ocean carbon consumption.

Response: Thank you for this suggestion. We have included the following sentence to emphasize the potential impact of eddy-driven carbon export (Lines 64-67):
'*Over the global scale, the submesoscale dynamics may have the potential to increase the carbon export, which would help to reconcile the imbalance between the upper layer carbon supply and deep ocean carbon consumption.*'

Line 86: a in the Chlorophyll-a should be italicized.
Response: Done.

Line 131: What kind of threshold or criteria do you apply to define the start and end period of the intensifying phase of eddy (i.e., the amplitude continuously increased over more than one week as you mentioned in the SI). I tried to sort it out from Figure 1 but didn't find a very consistent criterion. It is very important information in your study, so

it requires clear clarification in the main text of the manuscript.

Response: Following the suggestions from reviewers 1 and 2, the generalization of the eddy results to the entire Pacific gyre has large uncertainties. We thus removed the eddy analysis for the entire Pacific gyre in this revision. For the specific eddy we studied, we defined the start and end periods of the intensifying phase of the eddy by finding the period with continuously decreasing SLA of the eddy's lifespan. The SLA near the float decreased from 4 to 13 April. The average SLA in the eddy decreased at the beginning of 4 April, then increased for two days, and finally decreased almost synchronously with the SLA of the float until 14 April (Fig. R8). We thus used the float measurements during April 4-13 to describe the eddy intensifying stage and measurements during April 14-21 to describe the eddy mature state.

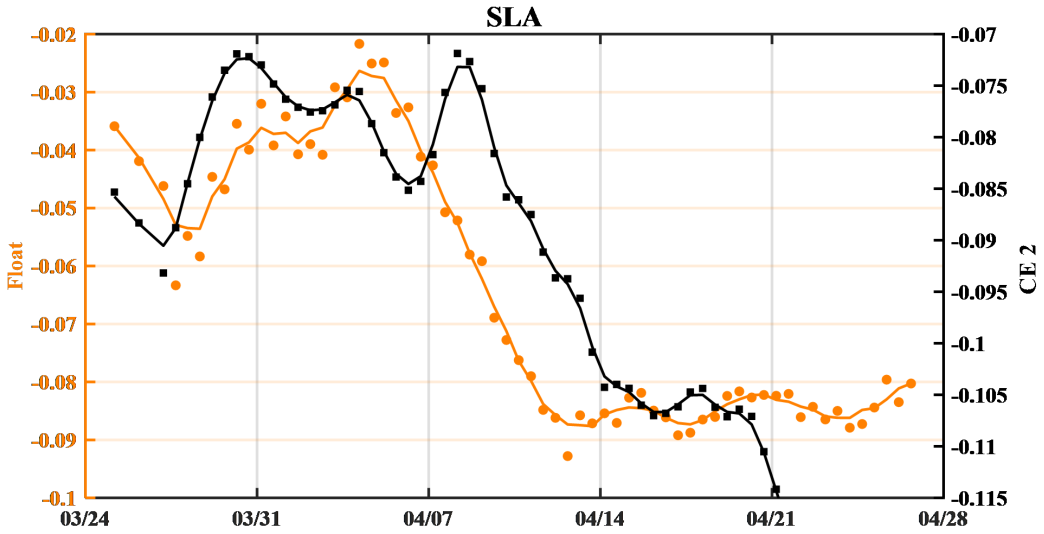

Figure R8. Time series of sea level anomaly (SLA) along (orange) the float trajectory and (black) averaged inside CE 2.

Line 214: p should be lowercase and italicized.

Response: Done.

Line 302: There is a need to discuss the caveats of the current study and provide a future roadmap to address these challenges.

Response: Thank you for the good suggestion. We have included some discussion about the caveat of the current study in the text and highlighted the need for high-frequency profiling to address the challenges with the following sentences (Lines 431-438):

'The current 10-day sampling strategy employed by the BGC-Argo program runs the risk of not capturing carbon export induced by the fine-sale processes. This study demonstrated the unique application of BGC-Argo floats for studying these structures

using a sampling strategy of twice a day for the operation of BGC-Argo. By deploying a large number of autonomous platforms sampling in high resolutions in the ocean, our understanding on ocean biological carbon pumps at different temporal and spatial scales will improve.'

Line 303: A more in-depth discussion is required regarding the potential implications of your work, such as the imbalance between carbon supply and demand. It would be also eye-opening to discuss the depth to which eddies can affect carbon export transfer, and its significance for long-term carbon sequestration, considering that carbon sequestration time is positive with the depth where carbon is eventually delivered.

Response: Thank you for the good suggestion. The following sentences to discuss the potential impact of submesoscale dynamics on carbon dynamics in the mesopelagic ocean have been added (Lines 438-446):

Although great efforts have been made to constrain the uncertainties in carbon sink and source estimations[9,40], the upper layer carbon export is still reported to be up to two to three times less than the mesopelagic carbon demand by heterotrophic metabolism[9,40], highlighting a requirement to reassess the unaccounted pathways that deliver carbon to deep waters[17]. This study demonstrated a higher carbon transport efficiency along the front compared to the climatological mean (T_{eff}=37% vs. T_{eff}=11%), suggesting that submesoscale dynamics could potentially result in closer agreement between sources and sinks of carbon and should be accounted for to address the imbalance in mesopelagic carbon budgets.

References:

Boyd, P.W., Claustre, H., Levy, M. et al. Multi-faceted particle pumps drive carbon sequestration in the ocean. *Nature* **568**, 327–335 (2019).

Burd, A. B. et al. Assessing the apparent imbalance between geochemical and biochemical indicators of meso- and bathypelagic biological activity: what the @$é! is wrong with present calculations of carbon budgets? *Deep-Sea Res. II* **57**, 1557–1571 (2010)

Giering, S., Sanders, R., Lampitt, R. et al. Reconciliation of the carbon budget in the ocean's twilight zone. *Nature* **507**, 480–483 (2014).

Line 311: should provide the sampling information of temporal and vertical resolution.

Response: The temporal and vertical resolution of the floats has been clarified in Lines 451-455:

'The floats profiled twice a day in CE 1 and CE 2 and along the frontal region, and then was set to profile once every three days in ACE 1. The floats sampled the first 30 m at a 0.2 m resolution, between 30-400 at a 1 m resolution and depths below 400 m at a 5 m resolution. All profile data were interpolated at a 5 m resolution.'

Line 314: Why did you use the dark correction value collected during the period before

the float deployment? In the standard Chl-a quality control procedure, the dark correction typically refers to in situ values at the depths around d 200-300m to account for the near-real-time background noise.

Response: Our description was revised as "*Before deployment, the dark currents of the chlorophyll fluorometer and backscattering sensor were corrected (reassigned) based on the dark-current measurement, by masking the optical windows with black tape.*" (Lines 458-462). Such a dark-current correction is more reliable than the deep-water correction method (i.e., using the deep-water Chla values assuming it is zero), and available to the backscattering sensor (the deep-water correction method cannot be conducted for backscattering, as the deep-water backscattering coefficient is not zero due to the presence of non algal particles).

Line 323: add the sample number and relevant statistical information (i.e., R2 and p values). There should be a space between “mmol” and “C”.

Response: The total number of data points for regression and the statistical information have been specified by adding the phrase ‘(A total of 32 data points for regression; R^2=0.39; p<0.01)’. The unit ‘mmolC’ has been corrected to ‘mmol C’.

Figure 3: Are there uncertainty estimates for AOU and POC rates? At the very least, it would take into account the error inherited from the linear fitting. What is unit of AOU rate (mmol C or mmol O2)?

Response: The uncertainties in the rates of change of POC and AOU are shown as error bars in Fig. 3c-f. This information has also been included in Table S7 in the text. The unit of the AOU rate was mmol O_2 m^{-2} d^{-1} in Fig. 3. Now we computed the net rate of averaged AOU instead, so the unit is mmol O_2 m^{-3} d^{-1}, which has been specified clearly in Fig. 4 e and f.

Line 344: Although the authors have briefly described the method to derive the time rate of POC and AOU inventory in the result section, it is still worthwhile providing more detailed information in the method section. It would be more appropriate to quantify the POC and AOU rate alongside the isopycnal surface to reduce the impact of the tracer rate induced by water vertical displacement.

Response: Thank you for the suggestion. We have revised the Methods section to clarify the method used to calculate the rate of change in the POC inventories and averaged AOU. We also tried to use the calculations along isopycnals. As we eventually calculated the change of integrated values (i.e., POC inventory, integrated or depth-averaged AOU), our main conclusions were not affected by the results either using isopycnal calculation or using depth calculation.

Figure 1: What is the temporal scale of background EKE? Should be clarified in the

caption. All the acronyms shown in the figures should be spelled out in the caption to make the figure self-explanatory. Does the black line indicate the position of the core of the eddy? Should make it clear.

Response: The background EKE was averaged from 1993 to 2020, which has been clarified in the caption of Fig. 1. The acronyms including CE 1, ACE 1, CE 2, NO_3, POC, EKE and r/R have been spelled out in the caption following the reviewer's suggestion. The black line is the center of the mesoscale eddies. We have clarified this in the caption.

REVIEWER COMMENTS

Reviewer #1 (Remarks to the Author):

The authors have made significant improvements to the manuscript and addressed the major concerns raised in the prior version.

In particular, the authors did a great job at improving and justifying the quantification of spatial and temporal variability. They included the use of satellite to justify that most of the signal associated with CE1 and ACE1 were spatial and not temporal. They also included material to back up that the front was intensifying (and not just sampling something else), which was a major shortcoming of the prior version. They combined Lagrangian methods - particles, Finite Size Laypunov Exponents – with temperature and salinity anomalies and now convincingly show that the float was indeed measuring the front while it was intensifying. Again, I stress the great efforts the authors have put in to address this issue!

The authors have also integrated the necessary literature in the introduction, in particular on fine scale subduction events and their impact on the biological carbon pump.

Finally, the extrapolation of the results to the basin-scale which was very speculative was removed from the results and is now mentioned in the discussion to motivate future studies in other regions. The message of the present version is more focused on the region of study and less general, but it is also much more robust and more convincingly convey the message about the potential role of fronts in increasing the vertical carbon export.

We still have a few minor suggestions to further strengthen the paper:
- Answer #7: Adding an uncertainty range on the mean values of b in summer and winter would clearly indicate how robust the difference is between the two seasons, and strengthen the robustness of the results.
- Answer #8: To back up the fact that the increase in POC between the base of the euphotic zone and 250m depth is linked to fine sacle subduction, the aithors could show spiciness anomalies which should reflect a surface water signature (e.g., Omand et al Science 2015; Llort et al, JGR 2018; Johnson and Omand, JGR 2021; Lacour et al, Nature Comms 2023). Note that the sign of the spiciness anomaly will depend on the surface to depth gradient and therefore on the region.

Once these minor points have been addressed, we are confident that the paper can be suitable for publication in Nature Communications.

References -
Johnson, A.R., Omand, M.M., 2021. Evolution of a Subducted Carbon-Rich Filament on the Edge of the

North Atlantic Gyre. Journal of Geophysical Research: Oceans 126, e2020JC016685. https://doi.org/10.1029/2020JC016685

Omand, M.M., D'Asaro, E.A., Lee, C.M., Perry, M.J., Briggs, N., Cetinić, I., Mahadevan, A., 2015. Eddy-driven subduction exports particulate organic carbon from the spring bloom. Science 348, 222–225. https://doi.org/10.1126/science.1260062

Lacour, L., Llort, J., Briggs, N., Strutton, P.G., Boyd, P.W., 2023. Seasonality of downward carbon export in the Pacific Southern Ocean revealed by multi-year robotic observations. Nat Commun 14, 1278. https://doi.org/10.1038/s41467-023-36954-7

Llort, J., Langlais C., Matear R., Moreau S., Lenton A., Strutton Peter G., 2018. Evaluating Southern Ocean Carbon Eddy-Pump From Biogeochemical-Argo Floats. Journal of Geophysical Research: Oceans 123, 971–984. https://doi.org/10.1002/2017JC012861

Reviewer #2 (Remarks to the Author):

The authors have made significant improvements to the manuscript and addressed the major concerns raised in the prior version.

In particular, the authors did a great job at improving and justifying the quantification of spatial and temporal variability. They included the use of satellite to justify that most of the signal associated with CE1 and ACE1 were spatial and not temporal. They also included material to back up that the front was intensifying (and not just sampling something else), which was a major shortcoming of the prior version. They combined Lagrangian methods - particles, Finite Size Laypunov Exponents – with temperature and salinity anomalies and now convincingly show that the float was indeed measuring the front while it was intensifying. Again, I stress the great efforts the authors have put in to address this issue!

The authors have also integrated the necessary literature in the introduction, in particular on fine scale subduction events and their impact on the biological carbon pump.

Finally, the extrapolation of the results to the basin-scale which was very speculative was removed from the results and is now mentioned in the discussion to motivate future studies in other regions. The message of the present version is more focused on the region of study and less general, but it is also much more robust and more convincingly convey the message about the potential role of fronts in increasing the vertical carbon export.

We still have a few minor suggestions to further strengthen the paper:

- Answer #7: Adding an uncertainty range on the mean values of b in summer and winter would

clearly indicate how robust the difference is between the two seasons, and strengthen the robustness of the results.

- Answer #8: To back up the fact that the increase in POC between the base of the euphotic zone and 250m depth is linked to fine sacle subduction, the aithors could show spiciness anomalies which should reflect a surface water signature (e.g., Omand et al Science 2015; Llort et al, JGR 2018; Johnson and Omand, JGR 2021; Lacour et al, Nature Comms 2023). Note that the sign of the spiciness anomaly will depend on the surface to depth gradient and therefore on the region.

Once these minor points have been addressed, we are confident that the paper can be suitable for publication in Nature Communications.

References -

Johnson, A.R., Omand, M.M., 2021. Evolution of a Subducted Carbon-Rich Filament on the Edge of the North Atlantic Gyre. Journal of Geophysical Research: Oceans 126, e2020JC016685. https://doi.org/10.1029/2020JC016685

Omand, M.M., D'Asaro, E.A., Lee, C.M., Perry, M.J., Briggs, N., Cetinić, I., Mahadevan, A., 2015. Eddy-driven subduction exports particulate organic carbon from the spring bloom. Science 348, 222–225. https://doi.org/10.1126/science.1260062

Lacour, L., Llort, J., Briggs, N., Strutton, P.G., Boyd, P.W., 2023. Seasonality of downward carbon export in the Pacific Southern Ocean revealed by multi-year robotic observations. Nat Commun 14, 1278. https://doi.org/10.1038/s41467-023-36954-7

Llort, J., Langlais C., Matear R., Moreau S., Lenton A., Strutton Peter G., 2018. Evaluating Southern Ocean Carbon Eddy-Pump From Biogeochemical-Argo Floats. Journal of Geophysical Research: Oceans 123, 971–984. https://doi.org/10.1002/2017JC012861

Reviewer #5 (Remarks to the Author):

Please see attached PDF

Responses to Reviewers (second round)

We sincerely thank all the reviewers for the constructive comments on the manuscript entitled ‘Efficient biological carbon export to the mesopelagic ocean induced by submesoscale fronts’. We have responded point-by-point to the reviewers’ questions and comments below. All page/line/reference/figure numbers refer to the tracked version of the manuscript. Reviewers’ comments are in regular text, our responses are in blue and new text from the manuscript is in *blue italics.*

Reviewer #1 and #2

(**Reviewer #1 and #2 coreviewed and the comments are the same. Please see our replies below**):

The authors have made significant improvements to the manuscript and addressed the major concerns raised in the prior version.

In particular, the authors did a great job at improving and justifying the quantification of spatial and temporal variability. They included the use of satellite to justify that most of the signal associated with CE1 and ACE1 were spatial and not temporal. They also included material to back up that the front was intensifying (and not just sampling something else), which was a major shortcoming of the prior version. They combined Lagrangian methods - particles, Finite Size Laypunov Exponents – with temperature and salinity anomalies and now convincingly show that the float was indeed measuring the front while it was intensifying. Again, I stress the great efforts the authors have put in to address this issue!

The authors have also integrated the necessary literature in the introduction, in particular on fine scale subduction events and their impact on the biological carbon pump.

Finally, the extrapolation of the results to the basin-scale which was very speculative was removed from the results and is now mentioned in the discussion to motivate future studies in other regions. The message of the present version is more focused on the region of study and less general, but it is also much more robust and more convincingly convey the message about the potential role of fronts in increasing the vertical carbon export.

We still have a few minor suggestions to further strengthen the paper:

Response: We would like to express our gratitude for your support to the last round of revision. The manuscript has been much improved with your suggestions. We also appreciate your constructive comments on how to further improve this manuscript and have revised the text accordingly, as detailed below.

- Answer #7: Adding an uncertainty range on the mean values of b in summer and winter would clearly indicate how robust the difference is between the two seasons, and strengthen the robustness of the results.

Response: Thank you for the good suggestion. The standard deviations (std) of the *b* value at ALOHA in both the summer and winter are calculated and added to the manuscript. As shown in Fig. S17, b=1.13±0.39 in summer and b=0.87±0.19 in winter.

The standard deviation is small in winter (std=0.19) and large in summer (std=0.39), and both are significantly smaller than the mean values (i.e., 1.13 and 0.87). The combination of the two-sample t test results and the standard deviation results suggests that the difference of the average *b* value between the two seasons is robust. The standard deviations have been included in Line 98 in the supporting information and Fig. S17.

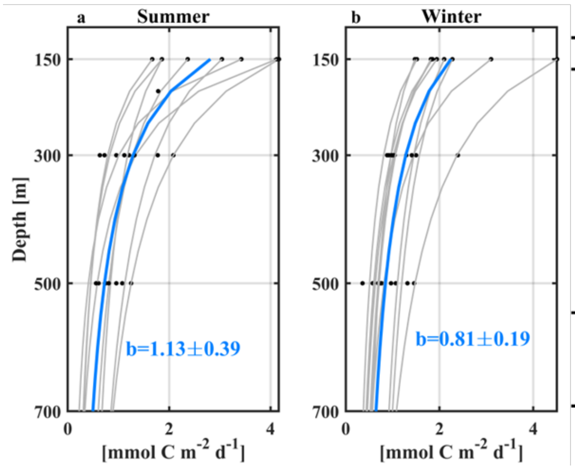

c

Summer	*b*	R^2	winter	*b*	R^2
1	1.08	0.93	1	0.93	0.98
2	1	1	2	0.93	1
3	0.83	1	3	0.94	1
4	1.63	0.99	4	0.61	0.96
5	0.66	0.95	5	0.81	1
6	1.17	0.98	6	0.91	0.93
7	0.89	0.96	7	0.53	0.99
8	1.77	0.94	8	1.11	0.98
			9	0.54	0.93
			10	0.78	1
Mean *b*	1.13±0.39			0.81±0.19	

Figure S17. The seasonal *b* value at the ALOHA station. **a** POC flux profiles and the corresponding Martin curves in summer. Black dots are observational profiles from sediment traps. Gray lines are the fitted Martin curves. The blue line is the Martin curve derived from the averaged *b* value and averaged POC flux at 150 m. **b** is the same as **a** but in winter. **c** The *b* value and R^2 for each observational POC flux profile in both summer and winter. We picked up all observational POC flux profiles with at least three vertical sampling points for Martin curve fitting. The *b* values in summer are significantly different from those in winter, as h=1 and p=0.036 in the two-sample t test result.

- Answer #8: To back up the fact that the increase in POC between the base of the euphotic zone and 250m depth is linked to fine scale subduction, the authors could show spiciness anomalies which should reflect a surface water signature (e.g., Omand et al Science 2015; Llort et al, JGR 2018; Johnson and Omand, JGR 2021; Lacour et al, Nature Comms 2023). Note that the sign of the spiciness anomaly will depend on the surface to depth gradient and therefore on the region.

Response: We thank the reviewer for this constructive suggestion and have estimated the spiciness distributions (Fig. S10) accordingly. Omand et al. (2015) showed that when a surface water parcel is injected into the subsurface, it carries positive anomalous values of spiciness and POC into the mesopelagic zone. The positive spiciness anomaly is evidence of sea surface water with high spiciness. In this study, the spiciness distributions decreased with depth in a way that is similar to those observed by Omand et al. (2015). Consistent with Omand et al. (2015), we present the cooccurrence of positive spiciness and POC anomalies below the euphotic zone (Fig. S10), suggesting that water masses rich in POC were subducted into the mesopelagic zone by

submesoscale processes (Omand et al., 2015; Llort et al., 2018; Johnson and Omand, 2021; Lacour et al., 2023). Both the positive spiciness and POC anomalies were stronger in the front intensifying stage than in the decaying stage. However, overall, the patches of positive spiciness anomalies were limited at depths between the euphotic zone and 250 m, and the patches of POC anomalies can also be seen at depths deeper than 250 m, suggesting that processes other than physical subduction also contribute to the POC anomalies in this region. To back up the conclusion that the elevated POC is linked to a subduction pump, we have included Fig. S10 in the supporting information and the following text in Lines 355-359, 373-375, and 462-466:

'Concurrent with the physical dynamics, there were patches of positive spiciness and POC anomalies between Z_{Eu} and ~250 m (Fig. S10), which is the evidence of subduction pump as suggested in previous studies[17,19,20,23,55]. The patches of positive spiciness and POC anomalies were stronger in the front intensifying stage than in the decaying stage (Fig. S10).'

'Overall, we show that physical subduction was mostly limited to depths of < 250 m. Below 250 m, POC export in the region could be largely attributable to the gravitational sinking of particles.'

'To identify subsurface features resulting from subduction pumps, spiciness that describes thermohaline perturbations not included in potential density was estimated using the BGC-Argo-derived temperature and salinity. As suggested by Omand et al. (2015), subsurface water masses originating from the euphotic zone were identified as subsurface anomalies in vertical POC and spiciness distributions.'

Additionally, the references listed by the reviewer have all been cited in this revision.

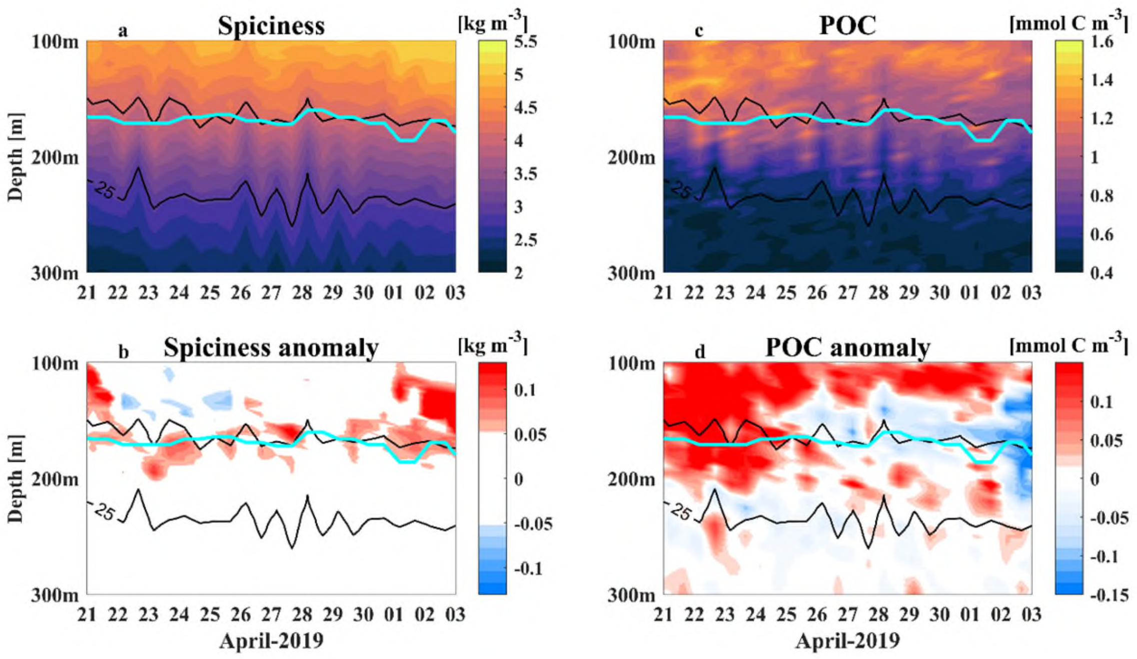

Figure S10. Vertical distribution of spiciness and POC. Time series of **a** spiciness and

c POC distributions measured by float 2902753 from 21 April to 3 May 2019. Time series of **b** spiciness anomalies and **d** POC anomalies. The cyan lines indicate the euphotic zone depth. The black lines are the isopycnals of 24 and 25 kg m^{-3}. The spiciness and POC anomalies were defined as the instantaneous values subtracting the annual time mean at the same isopycnal layer.

Once these minor points have been addressed, we are confident that the paper can be suitable for publication in Nature Communications.

Response: We appreciate your helpful suggestions to improve this manuscript.

References -

Johnson, A.R., Omand, M.M., 2021. Evolution of a Subducted Carbon-Rich Filament on the Edge of the North Atlantic Gyre. Journal of Geophysical Research: Oceans 126, e2020JC016685. https://doi.org/10.1029/2020JC016685

Omand, M.M., D'Asaro, E.A., Lee, C.M., Perry, M.J., Briggs, N., Cetini?, I., Mahadevan, A., 2015. Eddy-driven subduction exports particulate organic carbon from the spring bloom. Science 348, 222–225. https://doi.org/10.1126/science.1260062

Lacour, L., Llort, J., Briggs, N., Strutton, P.G., Boyd, P.W., 2023. Seasonality of downward carbon export in the Pacific Southern Ocean revealed by multi-year robotic observations. Nat Commun 14, 1278. https://doi.org/10.1038/s41467-023-36954-7

Llort, J., Langlais C., Matear R., Moreau S., Lenton A., Strutton Peter G., 2018. Evaluating Southern Ocean Carbon Eddy-Pump From Biogeochemical-Argo Floats. Journal of Geophysical Research: Oceans 123, 971–984. https://doi.org/10.1002/2017JC012861

Editorial Note: Parts of this Peer Review File have been redacted as indicated to remove third-party material where no permission to publish could be obtained.

Reviewer #5

The authors have done a nice job of addressing my original concerns. At this point, I have a few additional concerns for the authors and editors to consider in the evaluation of this work. Each of the two major points here has follow-up in the "specific revisions" section below. I once again thank the authors for a meticulously pursued research question using instrumentation that is becoming more accessible in a global context.

Response: We sincerely thank the reviewer for providing constructive and helpful suggestions to improve this manuscript. In the following point-by-point responses, we have addressed all the comments, including (1) the definitions of eddy and front intensification, maturity, and decay have been added in the text; (2) decisive statements summarizing our main findings regarding the two mechanisms by which submesoscale dynamics modulate POC transfer efficiency have been added in the discussion section; and (3) the discussion on the anticyclonic eddy has been removed from the text, following the reviewer's suggestion. Please see our detailed responses below.

1. **Remembering the audience and making the point clear:** This paper strives to fit a lot of work into a relatively short format journal. I think it can be done, but I think the paper would strongly benefit from the authors considering what can be removed for the sake of making the main points clear. As Nature Communications has a wide audience, sharpening the focus of the manuscript will increase readership and comprehension. Suggestions are detailed below but include removing discussion of the anticyclone and having a relatively decisive statement in the discussion about what the authors would like the audience to take away regarding carbon export in the subtropical gyres.

Response: Thank you for the good suggestion. The objective of this study is to investigate the POC transfer efficiency in response to episodic mesoscale and submesoscale processes using BGC-Argo high-frequency observations. The POC distributions in the anticyclonic eddy (ACE 1) presented in the previous version of the manuscript do not fit the objective and have been removed from the text following the reviewer's suggestion. We agree that this has made the paper more focused. Please find details in response to the specific comment #6 shown below.

The main point we would like the audience to take away from this study is that submesoscale processes during their intensifying stage can enhance POC vertical transfer efficiency in subtropical gyres. It is generally performed through the mechanisms of the biological carbon pump and/or the physical subduction pump. Regarding the biological carbon pump, strong submesoscale flows can enhance the vertical nutrient supply, alter the phytoplankton community structure, and generate particles sinking to depth. Some of the particles generated through this pathway, if not remineralized quickly, have the potential to penetrate the pycnocline and reach the

mesopelagic layer via gravitational sinking. The second mechanism is through physical subduction, by which particles are carried downward with the water mass. For this mechanism, the vertical depth the particle can reach is determined by the subduction velocity and the ventilation depth. From the data, we show that physical subduction was mostly limited to depths of < 250 m. Below 250 m, POC export in the region could be largely attributable to the gravitational sinking of particles.

To clearly deliver the message of this study, we have included these statements in the revised manuscript following the reviewer's suggestion in Lines 318-327 and 373-375:

'Two mechanisms affecting POC export at fronts have been shown to be important in previous studies[14-24,35]. The first mechanism is through biological carbon pump. Strong submesoscale flows can enhance the vertical nutrient supply[14,15,33], alter the phytoplankton community structure[32,35], and generate particles sinking to depth[26,41]. Some of the particles generated through this mechanism have the potential to penetrate the pycnocline and reach the mesopelagic layer via gravitational sinking[16,36,37]. The second mechanism is through physical subduction, by which particles are carried downward with the water mass[17-23]. For this mechanism, the vertical depth the particle can reach is largely determined by the subduction velocity and the ventilation depth[17-23].'

'Overall, we show that physical subduction was mostly limited to depths of < 250 m. Below 250 m, POC export in the region could be largely attributable to the gravitational sinking of particles.'

2. Definition of eddy and front intensification, maturity, and decay: I remain confused as to how the front and eddy are classified as "intensifying", "mature", or "decaying". For the front, I expect this definition is hiding in Figure S7 and perhaps Figure S8, but I am struggling to pick it out. For the eddies, I am unclear how the authors make these designations, which is important because it shapes the way they define additional export (Lines 197-198). Please define early and clearly how these classifications are made and also make the point at which you see a shift in the frontal expression clear.

Response: Thank you for pointing this out. The following have been added and/or discussed in the revised manuscript.

For the front, we define different stages of the front by examining changes of GM at the float locations shown in Fig. S6e. From 21 April to 27 April, the GM was elevated and larger than the background value, indicating that the float was sampling the front intensifying stage. From 28 April to 3 May, the GM decreased to the background value, indicative of the front decaying stage sampled by the float. These statements have been clearly added in the revised manuscript in Lines 261-265. Please also see details in our response to the specific comment #7.

Additionally, the shift of the two stages at the front was supported by the changes of R_i and M^2 in Fig. S8. As exhibited in Fig. S8b, R_i from the MLD to approximately 250 m became small during the intensifying stage and large during the decaying stage, suggesting that submesoscale motions were more active during the intensifying stage than during the decaying stage. Moreover, the magnitude of M^2 increased from 21 to 27 April and then decreased from 28 April to 3 May, reflecting the shift from the intensifying stage to the decaying stage. We have added more explanation to clarify this point in Lines 265-275. Please also see our response to the specific comment #13.

The large M^2 magnitude measured during the intensifying stage was associated with enhanced submesoscale dynamics. As strong secondary circulations across the front were generated during the intensifying stage, the temperature and salinity distributions along the front were consequently perturbed. Due to the heterogeneous evolution and distribution of the front over time, it also results in an increase of along-front buoyancy gradients as sampled by the float. However, note that the frontal dynamics were mainly driven by the change of the cross-front buoyancy gradient, which was much larger than the along-front buoyancy gradient. Thus, the enhanced along-front buoyancy gradient was only a manifestation of front intensification but not the driving force, as the float drifted along the front and the sampled physical characteristics of the water masses were generally similar (Fig. S6 and S7). These discussions have been added in Lines 269-273 to make them clear. Please also see our response to the specific comment #14.

Fig. S9 shows that the POC inventory was elevated during the intensifying stage. Meanwhile, large particles with fast sinking speeds, as indicated by the bbp spike signal, were detected in the mesopelagic layer (Fig. S9c). To explain this clearly, we have added the following sentences in Lines 277-281:

'During the intensifying stage, the POC inventory in the euphotic zone was elevated (Fig. S9b). Meanwhile, large particles were detected by spikes of backscattering coefficients in the mesopelagic layer[8] (Fig. S9c).'

For the eddy, we define the intensifying and mature stages of CE 2 based on the temporal change of SLA averaged inside CE 2, as generally used in previous studies. We then classified float measurements into intensifying-stage and mature-stage measurements by comparing the temporal changes of SLA near the float with those averaged inside CE 2. From 6 April to 14 April 2019, the CE 2 intensified with gradually decreasing SLA values (Fig. S4). The SLA near the float also decreased gradually from -0.03 m to -0.09 m, almost synchronously with the SLA decease inside CE 2 until 14 April (Fig. S4). After that, the SLA near the float and that averaged inside CE 2 both remained at extremely low values until 22 April, indicating that the eddy was in a mature stage during this period. The float was not located exactly at the eddy center, but it maintained a relatively constant distance to the eddy center (r/R=~0.7) and experienced different stages of the eddy. We thus used the float measurements during 6-14 April to describe the eddy intensifying stage and measurements during 15-22 April

to describe the eddy mature stage. Based on the two stages identified from the SLA changes, the carbon export was then estimated. We clarify the definition of the intensifying and mature stages of the eddy with the following sentences in Lines 167-180:

'We define the intensifying and mature stages of CE 2 based on the temporal change of SLA averaged inside CE 2. The float 2902756 measurements were then classified into intensifying-stage and mature-stage measurements by comparing the change of SLA near the float with that averaged inside CE 2. From 6 April to 14 April 2019, the CE 2 intensified with gradually decreasing SLA values (Fig. S4). The SLA near the float also decreased gradually from -0.03 m to -0.09 m, almost synchronously with the SLA decease inside CE 2 until 14 April (Fig. S4). After that, the SLA near the float and that averaged inside CE 2 both remained at extremely low values until 22 April, indicating that the eddy was in a mature stage during this period. Except for a fluctuation in the relative position (r/R) during the first few days, the float remained at a r/R of approximately 0.7 inside CE 2 until 22 April 2019 (Fig. 1c). We thus used the float measurements during 6-14 April to describe the eddy intensifying stage and measurements during 15-22 April to describe the eddy mature stage.'

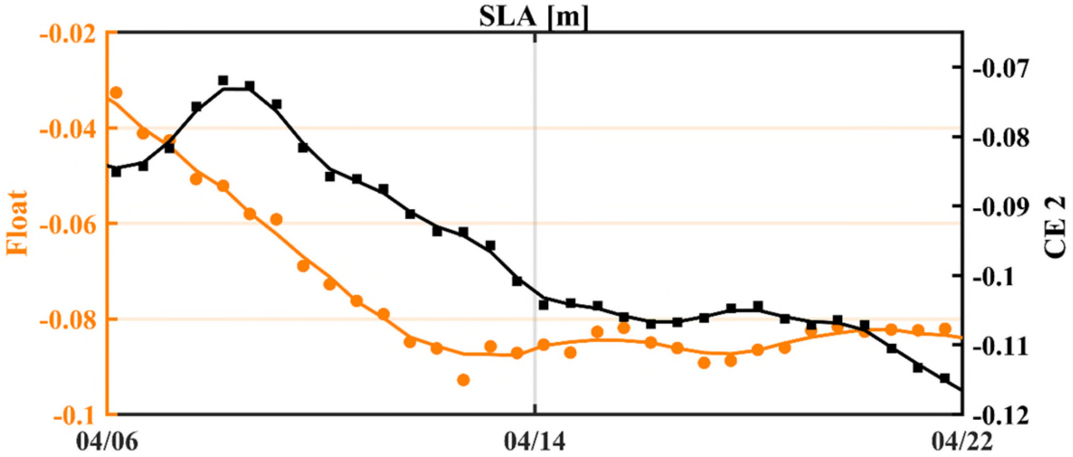

Figure S4. Time series of sea level anomaly (SLA) along (orange) the float trajectory and (black) averaged inside CE 2.

Specific revisions:

1. Line 34: To make this paragraph flow better, add a phrase at the start of the sentence currently starting "Weak vertical nutrient supply…" Perhaps "In these regions, weak vertical nutrient supply…"

Response: Done. The phrase 'In these regions, ' has been added to the beginning of the sentence. (Line 34)

2. Line 60: Rather than "increase" export, I suggest "contribute to"

Response: Done. The word 'increase' has been replaced with 'contribute to'. (Line 59)

3. Line 70: I suggest "whereas the effect of submesoscale features are still unclear"

Response: Done. We have replaced 'the submesoscale features' with 'the effects of submesoscale features'. (Line 70)

4. Line 71-72: This feels somewhat unrepresentative of what the literature actually shows. Omand et al. (2015) shows only the estimated springtime export, and the 50%+ number comes from regions like the Southern Ocean and North Atlantic. Llort et al. (2018) found that as little as 1% of export (in spring) could be from submesoscale motions. I encourage the authors to be careful with how they represent these numbers. I think it actually adds more motivation to the work that the range is so large (and so spring/summer focused).

Response: Thank you for pointing this out and providing suggestions to improve it. We did not make this clear in the last manuscript. In terms of the impact of submesoscale subduction on carbon export, previous studies indeed demonstrated a large range. Using high-resolution simulations, Omand et al. (2015) showed that the subduction pump contributes up to 50% of the spring POC flux in the North Atlantic and the Southern Ocean. However, Llort et al. (2018) showed that the contribution ranged between 1% in spring and 14-19% in summer in the Southern Ocean. By using a model covering a subpolar and a subtropical gyre, Resplandy et al. (2019) suggested that the subduction pump contributes less than 5% to the annual carbon export due to the strong compensation between upward and downward fluxes at the basin scale. Given the large uncertainty among the estimations from different studies, more field observations are required to evaluate the impact of submesoscale features on POC export flux. We have rephrased the sentences to summarize what the literature actually shows in Lines 70-82:

'Using high-resolution simulations, Omand et al. (2015) showed that the subduction pump contributes up to 50% of the spring POC flux in the North Atlantic and the Southern Ocean[19]. However, Llort et al. (2018) showed that the contribution ranged between 1% in spring and 14-19% in summer in the Southern Ocean[23]. By using a model covering a subpolar and a subtropical gyre, Resplandy et al. (2019) suggested that the subduction pump contributes less than 5% to the annual carbon export due to the strong compensation between upward and downward fluxes at the basin scale[18]. Given the large uncertainty among the estimations from different studies, more high-resolution field observations are required.'

5. Suggest renaming section 2.1 "Site characterization and tracer distributions" or something to that effect.

Response: Done. Section 2.1 has been renamed as 'Site characterization and tracer distributions'.

6. (related to major point 1) I am not convinced that including ACE 1 is necessary for

this paper, because it is not included in the key results or discussion beyond section 2.1. I think removing the discussion of this feature from the paper could tighten the results and figures and additionally make the focus on carbon export more clear. I do know that a lot of effort went into this data collection and analysis, but in this kind of journal, you need to be concise and focused in your results.

Response: Thank you for this constructive suggestion. To focus on carbon exports more clearly, the results and figures related to ACE 1 have been removed from this revision. Details of the revision are listed below:

Text: The introduction of ACE 1 and description of Chl and POC distributions in ACE 1 have been removed throughout the text, specifically in Lines 97, 120-125, 128-131, 137, 140, 142, 145-148, 157-161 and 441.

Fig. 1: The trajectory of ACE 1 in Fig. 1a has been removed. The original Fig. 1c showing the r/R and SLA in ACE 1 has been removed.
Fig. 2: The original panel b showing the locations of the float in ACE 1 has been removed.
Fig. 3: The original panel b showing the POC distributions in ACE 1 has been removed.
Fig. S1: The original panel b showing the Z_{eu}, MLD and Z_{SCM} in ACE 1 has been removed.
Fig. S2: Panels showing the spatial and temporal variabilities of Chl in ACE 1 have been removed.
Fig. S3: The radial distributions of temperature, NO_3, Chl and POC in ACE 1 have been removed.

Table S1: The sampling information when the BGC-Argo was in AC 1 has been removed.
Table S2: Information on the characteristics of ACE 1 has been removed.
Table S3: The correlation coefficients for ACE 1 have been removed.

7. (related to major point 2) Lines 112-113: "Here, we define the intensifying stage of the front as the front associated with enhanced submesoscale dynamics compared with the background condition." This sentence confuses me in this location because the authors have not yet demonstrated that this is a region of enhanced submesoscale motions. I suggest reframing this section or moving Figure S7 up if this is where you want to have that discussion.

Response: Thank you for pointing this out. We agree and have moved this sentence about the definition of submesoscale front enhancement from section 2.1 to section 2.3, where we discuss POC flux induced by an intensifying submesoscale front.

For the front, we define different stages of the front by examining changes of GM at the float locations shown in Fig. S6e. From 21 April to 27 April, the GM was elevated

and larger than the background value, indicating that the float was sampling the front intensifying stage. From 28 April to 3 May, the GM decreased to the background value, indicative of the front decaying stage sampled by the float. These statements have been clearly added in the revised manuscript in Lines 261-265 as shown below:

'We define different stages of the front by examining changes of GM at the float locations. From 21 April to 27 April, the GM was elevated and larger than the background value, indicating that the float was sampling the front intensifying stage. From 28 April to 3 May, the GM decreased to the background value, indicative of the front decaying stage sampled by the float (Fig. S6e).'

Support for the definition of the two stages comes from the temporal changes of R_i and M^2 as well (Fig. S8b and S8c). The submesoscale motions are more active during the intensifying stages as the distributions of R_i from the MLD to approximately 250 m became small during the intensifying stage and large during the decaying stage, suggesting that submesoscale motions were more active during the intensifying stage than during the decaying stage. Moreover, the magnitude of M^2 increased from 21 to 27 April and then decreased from 28 April to 3 May, reflecting the shift from the intensifying stage to the decaying stage. To further support the definition of intensifying and decaying stages, we have added the following sentences to the text in Lines 265-275:

'In addition, the R_i between the MLD and 250 m depth was smaller during the intensifying stage than during the decaying stage (Fig. S8b). Consistent with the change of R_i, the magnitude of the lateral buoyancy frequency (M^2) increased during the intensifying stage and decreased during the decaying stage (see the M^2 envelope in Fig. S8c). During the intensifying stage, submesoscale dynamics are prone to generate secondary circulations across the front, which in turn change the temperature and salinity distributions along the front. Due to the heterogeneous evolution and distribution of the front over time, it also results in the increase of M^2 magnitude along the front. The temporal changes in R_i and M^2 suggest that submesoscale motions were more active during the intensifying stage than during the decaying stage.'

8. Line 131: The term "completely different" is a little casual - perhaps just say it was different, or significantly different.

Response: Done. We have followed the reviewer's suggestion to remove the word 'completely'.

9. Line 162: Perhaps instead of saying a "relatively stable" location, a better term is "the float was traveling in a quasi-Lagrangian manner inside the eddy"?

Response: Done. The phrase 'the float was in a relatively stable location in the eddy' has been replaced with 'the float was traveling in a quasi-Lagrangian manner inside the eddy'.

10. (related to major point 2) Line 166: “the eddy was in a mature stage” - how is this determined? Sea surface height?

Response: We define the intensifying and mature stages of CE 2 based on the temporal change of SLA averaged inside CE 2, as generally used in previous studies. We then classified float measurements into intensifying-stage and mature-stage measurements by comparing the temporal changes of SLA near the float with those averaged inside CE 2. From 6 April to 14 April 2019, the CE 2 intensified with gradually decreasing SLA values (Fig. S4). The SLA near the float also decreased gradually from -0.03 m to -0.09 m, almost synchronously with the SLA decease inside CE 2 until 14 April (Fig. S4). After that, the SLA near the float and that averaged inside CE 2 both remained at extremely low values until 22 April, indicating that the eddy was in a mature stage during this period. The float was not located exactly at the eddy center, but it maintained a relatively constant distance to the eddy center (r/R=~0.7) and experienced different stages of the eddy. We thus used the float measurements during 6-14 April to describe the eddy intensifying stage and measurements during 15-22 April to describe the eddy mature stage. We clarify the definition of the intensifying and mature stages of the eddy with the following sentences in Lines 167-180:

‘We define the intensifying and mature stages of CE 2 based on the temporal change of SLA averaged inside CE 2. The float 2902756 measurements were then classified into intensifying-stage and mature-stage measurements by comparing the change of SLA near the float with that averaged inside CE 2. From 6 April to 14 April 2019, the CE 2 intensified with gradually decreasing SLA values (Fig. S4). The SLA near the float also decreased gradually from -0.03 m to -0.09 m, almost synchronously with the SLA decease inside CE 2 until 14 April (Fig. S4). After that, the SLA near the float and that averaged inside CE 2 both remained at extremely low values until 22 April, indicating that the eddy was in a mature stage during this period. Except for a fluctuation in the relative position (r/R) during the first few days, the float remained at a r/R of approximately 0.7 inside CE 2 until 22 April 2019 (Fig. 1c). We thus used the float measurements during 6-14 April to describe the eddy intensifying stage and measurements during 15-22 April to describe the eddy mature stage.’

11. Line 185: “appeared to” - can you just say the difference decreased to remove the hedging language?

Response: Thank you for the suggestion. The phrase ‘the difference appeared to decrease’ has been replaced with ‘the difference decreased’. Additionally, the whole manuscript has been checked to avoid such hedging language.

12. Line 189-190: This is a personal preference, but I think rate of change of AOU is a more clear term than “AOU change rate” or “AOU rate”.

Response: We have followed the reviewer’s suggestion to replace the term ‘AOU change rate’ or ‘AOU rate’ with ‘the rate of change of AOU’ throughout the text.

Specifically, in Lines 210-212, 218, 223, 288, and 841.

13. (related to major point 2) Line 239-241: "These two stages were also reflected by temporal changes in the lateral buoyancy frequency (Fig. S7a and c), FSLE(Fig. S7c)...". I am not sure what the authors are pointing at here. I struggle to see two stages (intensifying and decaying) represented in S7a or S7c. Perhaps putting a line on the figures where they see the change would help?

Response: Thank you for the good suggestion. We have added a dashed line in this figure to better show the two stages. The definitions of the two stages were actually based on the temporal change of GM shown in Fig. S6e. From 21 April to 27 April, the GM was elevated and larger than the background value, indicating that the float was sampling the front intensifying stage. From 28 April to 3 May, the GM decreased to the background value, indicative of the front decaying stage sampled by the float. These statements have been clearly added in the revised manuscript in Lines 261-265.

Additionally, the shift of the two stages at the front was supported by the changes of R_i and M^2 in Fig. S8. As exhibited in Fig. S8b, R_i from the MLD to approximately 250 m became small during the intensifying stage and large during the decaying stage, suggesting that submesoscale motions were more active during the intensifying stage than during the decaying stage. The magnitude of M^2 increased from 21 to 27 April and then decreased from 28 April to 3 May, reflecting the shift from the intensifying stage to the decaying stage. The FSLEs increased from 21 to 27 April and then decreased from 28 April to 3 May, likely supporting the two-stage statement as well.

We have added more explanation to clarify this point in Lines 265-275 and Lines 259-261:

'In addition, the R_i between the MLD and 250 m depth was smaller during the intensifying stage than during the decaying stage (Fig. S8b). Consistent with the change of R_i, the magnitude of the lateral buoyancy frequency (M^2) increased during the intensifying stage and decreased during the decaying stage (see the M^2 envelope in Fig. S8c). During the intensifying stage, submesoscale dynamics are prone to generate secondary circulations across the front, which in turn change the temperature and salinity distributions along the front. Due to the heterogeneous evolution and distribution of the front over time, it also results in the increase of M^2 magnitude along the front. The temporal changes in R_i and M^2 suggest that submesoscale motions were more active during the intensifying stage than during the decaying stage.'

'The ageostrophic motions at the front can also be indicated by the increased finite-size Lyapunov exponent (FSLE) distributions at the front (Fig. S8c).'

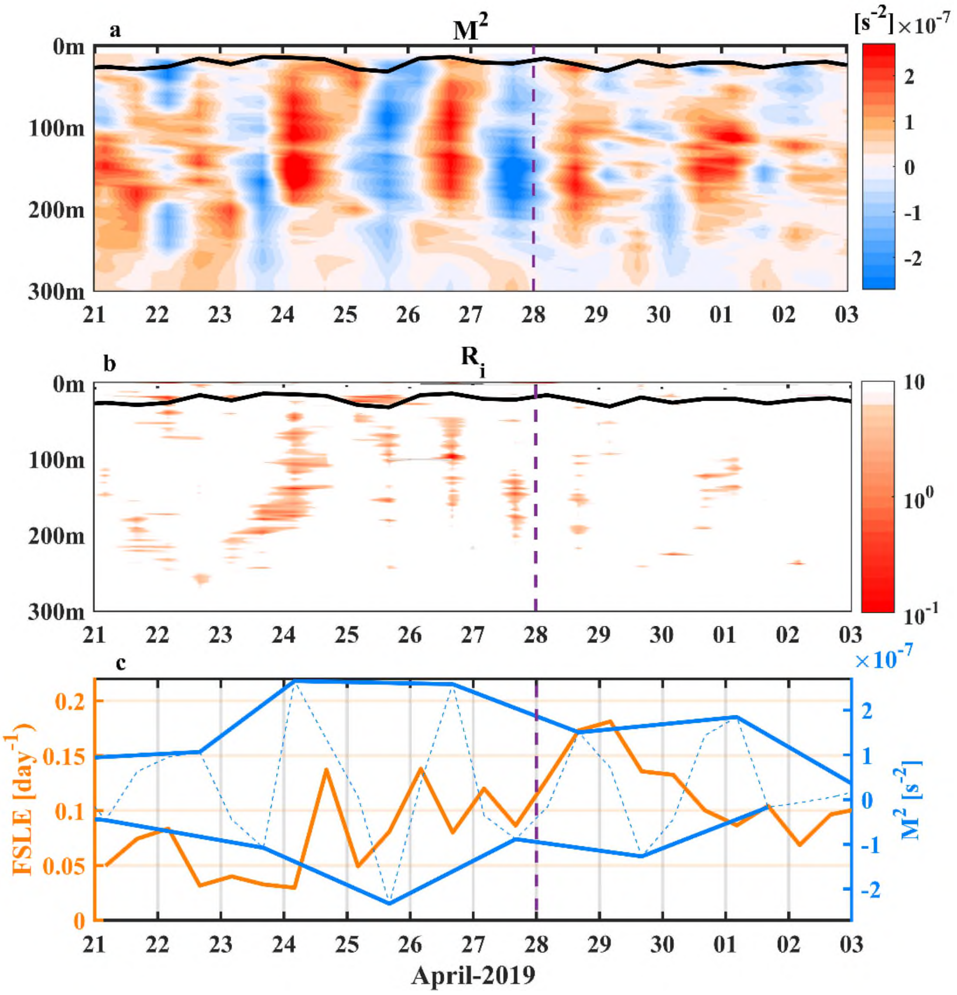

Figure S8. The lateral buoyancy frequency (M^2), Richardson number (R_i) and finite-size Lyapunov exponent (FSLE) at the front. **a** BGC-Argo-derived lateral buoyancy frequency during 21 April-3 May 2019. $M^2 = -\partial b/\partial s$, where s is the along-track direction. The black line represents the mixed layer depth. **b** BGC-Argo-derived Richardson number during 21 April-3 May 2019. $R_i = f^2 b_z / b_s^2$, where f and b are the Coriolis parameter and buoyancy, respectively. The black line represents the mixed layer depth. **c** Surface FSLE in the frontal region (orange line) and the lateral buoyancy frequency (blue dashed line) at 100 m during 21 April-3 May 2019. Blue solid lines are the buoyancy frequency envelope.

14. Related to the above, I'm unclear how if the float was in a homogenous water mass along the submesoscale front from April 21-May 3 (as described in lines 231-234 and figure S6) how there are such strong lateral gradients of buoyancy (M^2, figure S7a). Do the authors have a clear explanation for this?

Response: Thank you for pointing this out. In this study, we have demonstrated that the float drifted along one side of the front by conducting a numerical particle tracking experiment. We also showed that the water masses were generally similar along this

side of the front by analyzing the float-sampled temperature and salinity distributions. In this revision, we have replaced ‘homogenous water mass’ with ‘similar water masses’ throughout the text.

The large M^2 magnitude measured during the intensifying stage was associated with enhanced submesoscale dynamics. As strong secondary circulations across the front were generated during the intensifying stage, the temperature and salinity distributions along the front were consequently perturbed. Due to the heterogeneous evolution and distribution of the front over time, it also results in an increase of along-front buoyancy gradients as sampled by the float. However, note that the frontal dynamics were mainly driven by the change of the cross-front buoyancy gradient, which was much larger than the along-front buoyancy gradient. Thus, the enhanced along-front buoyancy gradient was only a manifestation of front intensification but not the driving force, as the float drifted along the front and the sampled physical characteristics of the water masses were generally similar (Fig. S6 and S7).

Here, we compared the magnitude of M^2 with those reported in previous studies, such as glider observations in the southern Drake Passage (Fig. R1b; Vigligone et al., 2018) and near the Porcupine Abyssal Plain (PAP) site (Fig. R1c; Thompson et al. 2016), as well as seal observations in the Southern Ocean (Siegelman et al., 2020). Estimation of the buoyancy gradient ($|M^2|$) in the southern Drake Passage showed a range of 10^{-8} to 10^{-5} s^{-2} during the four-month observations (Fig. R1b; Vigligone et al., 2018). Large variability of $|M^2|$ ranging from 10^{-6} to 10^{-5} s^{-2} was also exhibited by full seasonal observations near the PAP (Fig. R1c; Thompson et al. 2016). As suggested by Siegelman et al. (2020), the glider trajectory can be considered to be at submesoscale active regions only when $|M^2|$ is larger than $2.5x10^{-7}$ s^{-2}. Only a small proportion of the glider trajectories reported in Vigligone et al. (2018) and Thompson et al. (2016) were in submesoscale active regions since only ~10% of the $|M^2|$ was in the range of $2.5x10^{-7}$ to 10^{-5} s^{-2}. Consistent with Vigligone et al. (2018) and Thompson et al. (2016), over 90% of the $|M^2|$ estimated in our study was between $5.0x10^{-9}$ and $1.0x10^{-7}$ s^{-2} (Fig. R1a); however, less than 1% of $|M^2|$ was larger than $2.5x10^{-7}$ s^{-2}. The reason that the magnitude of $|M^2|$ is smaller than that expected by Siegelman et al. (2020) is that the float drifted along the front rather than across the front.

To explain the reason for the large lateral buoyancy gradients observed along the front during its intensifying stage, we have added the following sentences to the text (Lines 269-273:

‘*During the intensifying stage, submesoscale dynamics are prone to generate secondary circulations across the front, which in turn change the temperature and salinity distributions along the front. Due to the heterogeneous evolution and distribution of the front over time, it also results in the increase of M^2 magnitude along the front.*’

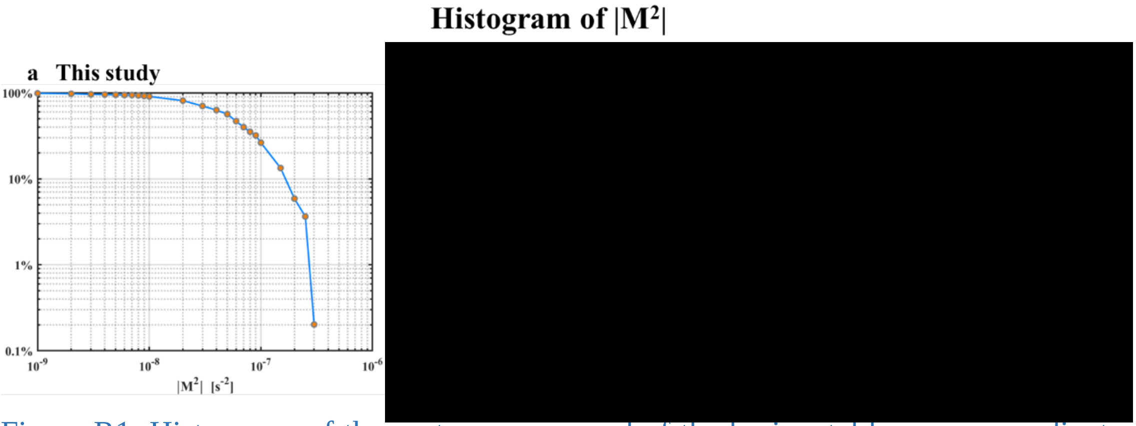

[redacted]

Figure R1. Histograms of the root mean squared of the horizontal buoyancy gradient (|M^2|) from **a** this study, **b** Viglione et al. (2018) and **c** Thompson et al. (2016). Here, |M^2| in the upper 100 m at the front is used for statistical analysis.

References:
Thompson, A. F., Lazar, A., Buckingham, C., Naveira Garabato, A. C., Damerell, G. M., & Heywood, K. J. Open-Ocean Submesoscale Motions: A Full Seasonal Cycle of Mixed Layer Instabilities from Gliders. *J. Phys. Oceanogr.*, **46**, 1285–1307. (2016).
Viglione, G. A., Thompson, A. F., Flexas, M. M., Sprintall, J., & Swart, S. Abrupt Transitions in Submesoscale Structure in Southern Drake Passage: Glider Observations and Model Results. *J. Phys. Oceanogr.*, **48**, 2011–2027. (2018).
Siegelman, L., Klein, P., Rivière, P. et al. Enhanced upward heat transport at deep submesoscale ocean fronts. *Nat. Geosci.* **13**, 50–55 (2020).

15. Paragraph starting at line 321: Please consider including the limitation that SST gradients alone may not account for changes in density due to salinity, the importance of which is detailed in Jaeger & Mahadevan (2018). [Jaeger, Gualtiero Spiro, and Amala Mahadevan. 2018. “Submesoscale-Selective Compensation of Fronts in a Salinity-Stratified Ocean.” Science Advances 4 (2): e1701504. https://doi.org/10.1126/sciadv.1701504.]

Response: Thank you for this suggestion. As explained in Jaeger and Mahadevan (2018), temperature variations play a leading role, while salinity variations play a secondary role in governing the density gradients in much of the world’s upper oceans. Therefore, the satellite-derived SST gradient as an indicator of submesoscale fronts has been suggested to be useful to investigate the spatiotemporal variation in submesoscale fronts. However, in regions that are strongly influenced by freshwater runoff and rain

or have close-to-freezing surface temperatures, salinity variations rather than temperature variations govern the near-surface density gradients. It may thus introduce uncertainties in the identified front and the estimated *b* value when using SST only to indicate the front. As high-resolution sea surface salinity data are currently not available from satellites, we have added the following sentences to present the limitations of using the SST gradient as an indicator of fronts (Lines 390-399):

'Note that the estimation in this study is based on the relationship between the b value and GM. In much of the world's ocean, temperature variations play a leading role in controlling the spatial near-surface density gradients, while salinity plays a secondary role[56]. Therefore, the satellite-derived SST gradient as an indicator of submesoscale fronts has been suggested to be useful to investigate the spatiotemporal variation in submesoscale fronts. However, in regions that are strongly influenced by freshwater runoff and rain or have close-to-freezing surface temperatures, salinity variations govern the near-surface density gradients. The GM method cannot identify density fronts governed by salinity variations[57]. It may thus introduce uncertainties in the identified front and the estimated b value.'

16. (related to major point 1) In response to reviewer 1, the authors stated: "The first mechanism [investigated in this study] is through a biological carbon pump. Strong submesoscale flows can enhance the vertical nutrient supply, alter the phytoplankton community structure, and generate particles sinking to depth. Some of the particles generated through this mechanism, if not remineralized quickly, have the potential to penetrate the pycnocline and reach the mesopelagic layer via gravitational sinking. The second mechanism is through subduction, by which particles are carried downward with the water mass. For this mechanism, the vertical depth the particle can reach is largely determined by the subduction velocity and the ventilation depth." Such a concise and clear explanation for the topics you are investigating is somewhat lacking from the paper as it stands, although the individual components are scattered throughout. I would highly encourage the authors to include such a statement at the start of the discussion or somewhere similar.

Response: We appreciate the reviewer's comment and agree that it is important to provide a clear explanation of the two mechanisms at the beginning of the discussion. The statement mentioned by the reviewer has been incorporated in the discussion as a separate paragraph in Lines 318-327, following which we then discussed the mechanisms of the biological carbon pump and eddy subduction pump one by one from Line 328 to 375.

'Two mechanisms affecting POC export at fronts have been shown to be important in previous studies[14-24,35]. The first mechanism is through biological carbon pump. Strong submesoscale flows can enhance the vertical nutrient supply[14,15,33], alter the phytoplankton community structure[32,35], and generate particles sinking to depth[26,41]. Some of the particles generated through this mechanism have the potential to penetrate the pycnocline and reach the mesopelagic layer via gravitational sinking[16,36,37]. The second mechanism is through physical subduction, by which particles are carried

downward with the water mass[17-23]*. For this mechanism, the vertical depth the particle can reach is largely determined by the subduction velocity and the ventilation depth*[17-23]*.'*

'Overall, we show that physical subduction was mostly limited to depths of < 250 m. Below 250 m, POC export in the region could be largely attributable to the gravitational sinking of particles.'

17. Figure 2: A very small thing, but check that the font sizes for your axes are the same (e.g. x axis in panel d).

Response: Thank you for pointing this out. The font sizes for all panels are now the same.

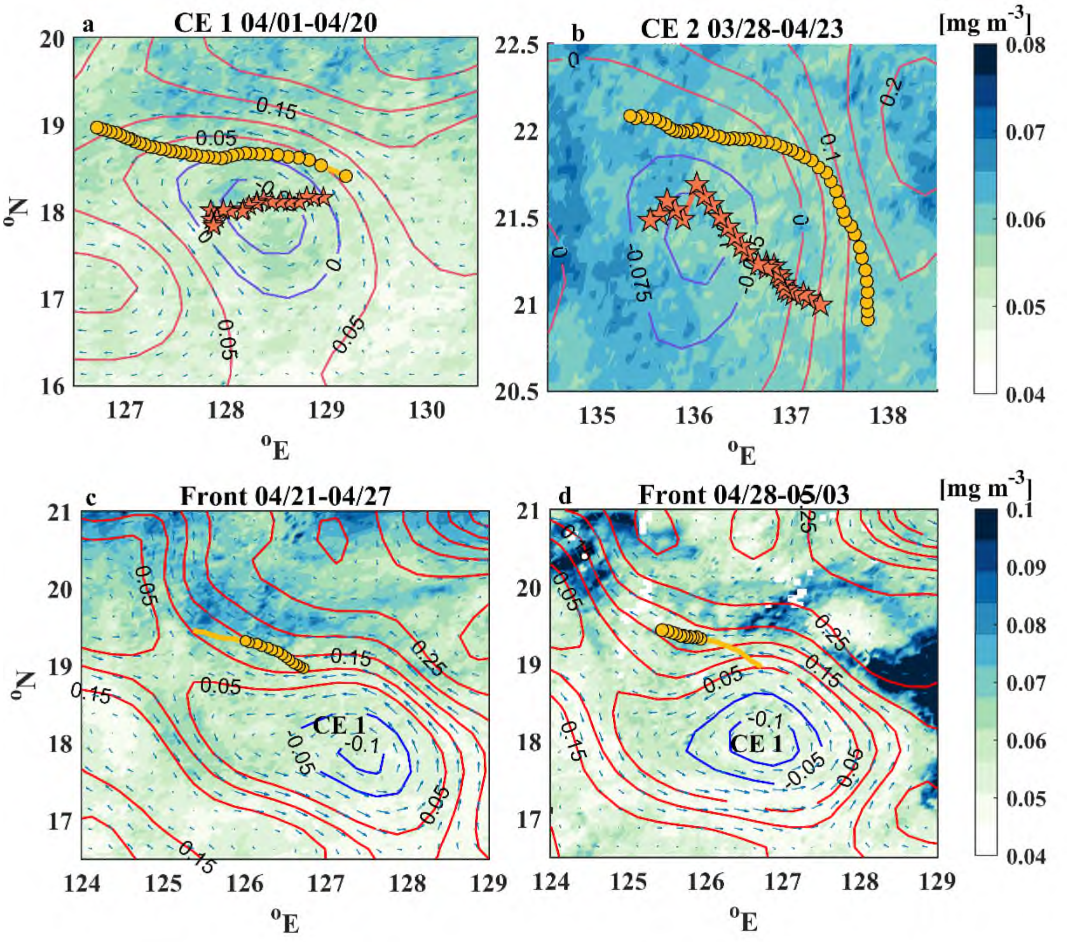

Figure 2. The BGC-Argo and eddy trajectories. **a** The locations of (yellow dots) the BGC-Argo float (WMO number 2902753) and (orange pentagrams) the center of cyclonic eddy 1 (CE 1) during 1-20 April 2019. **b** The locations of (yellow dots) the BGC-Argo float (WMO number 2902756) and (orange pentagrams) the center of cyclonic eddy 2 (CE 2). **c** The locations of the BGC-Argo floats along the front during 21-27 April 2019. The yellow line is the float trajectory from 21 April to 3 May 2019. **d** The same as d but during 28 April-3 May 2019. Background color and isolines

represent sea surface chlorophyll and sea level anomalies, respectively. The arrows are the geostrophic velocity anomalies.
